# Formation of a hydride containing amido-zincate using pinacolborane†

**DOI:** 10.1039/d1dt02580e

**Published:** 2021-09-15

**Authors:** Marina Uzelac, Kang Yuan, Gary S. Nichol, Michael J. Ingleson

**Affiliations:** EaStCHEM School of Chemistry, The University of Edinburgh David Brewster Road Edinburgh EH9 3FJ UK michael.ingleson@edinburgh.ac.uk

## Abstract

Amido-zincates containing hydrides are underexplored yet potentially useful complexes. Attempts to access this type of zincate through combining amido-organo zincates and pinacolborane (HBPin) *via* Zn–C/H–BPin exchange led instead to preferential formation of amide–BPin and/or [amide–BPin(Y)]^−^ (Y = Ph, amide, H), when the amide is hexamethyldisilazide or 2,2,6,6-tetramethylpiperidide and the hydrocarbyl group was phenyl or ethyl. In contrast, the use of a dipyridylamide (dpa) based arylzinc complex led to Zn–C/H–BPin metathesis being the major outcome. Independent synthesis and full characterisation of two L_*n*_Li[(dpa)ZnPh_2_] (L = THF, *n* = 3; L = PMDETA, *n* = 1) complexes, **1** and **3**, respectively, enabled reactivity studies that demonstrated that these species display zincate type reactivity (by comparison to the lower reactivity of the neutral complex (Me-dpa)ZnPh_2_, **4**, Me-dpa = 2,2′-dipyridyl-*N*-methylamine). This included **1** performing the rapid deprotonation of 4-ethynyltoluene and also phenyl transfer to α,α,α-trifluoroacetophenone in contrast to neutral complex **4**. Complex **1** reacted with one equivalent of HBPin to give predominantly PhBPin (*ca.* 90%) and a lithium amidophenylzincate containing a hydride unit, complex **7-A**, as the major zinc containing product. Complex **7-A** transfers hydride to an electrophile preferentially over phenyl, indicating it reacts as a hydridozincate. Attempts to react **1** with >1 equivalent of HBPin or with catecholborane led to more complex outcomes, which included significant borane and dpaZn substituent scrambling, two examples of which were crystallographically characterised. While this work provides proof of principle for Zn–C/H–BPin exchange as a route to form an amido-zincate containing a hydride, amido-organozincates that undergo more selective Zn–C/H–BPin exchange still are required.

## Abstract 2

Careful tuning of the nature of the amide ligand in amido-zincates allows for selective Zn–C over Zn–N exchange with HBPin affording a hydride containing amido-zincate. The mixed hydrido-phenyl zincate preferentially transfers hydride over phenyl.

## Graphical abstract

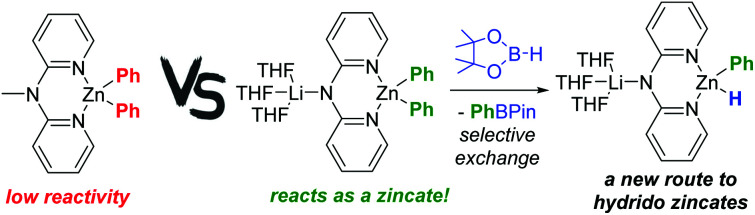

## Introduction

**Chart 1:**
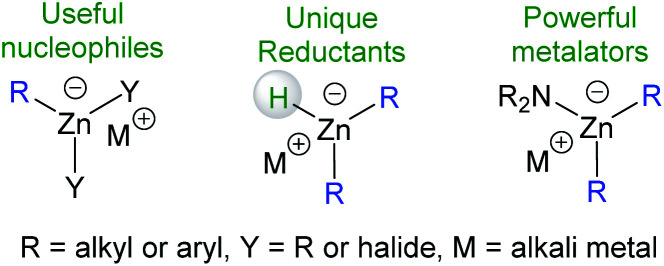
Select classes of important zincates.

Zincates, such as monoanionic three coordinate zinc complexes, are receiving increasing attention due to their unique reactivity.^1^ For example, organozincates ([R_*n*_ZnX_3−*n*_]^−^*n* ≥ 1, X = halide, R = hydrocarbyl group) are important nucleophiles in metal-halogen exchange and transmetalation reactions,^2^ thus can be key species in Negishi cross coupling reactions.^3^ Another notable class of zincates are the mixed amido-containing zincates that are powerful Brønsted bases able to effect C–H metalations,^4^ including of challenging substrates such as benzene (Chart 1).^5^ Detailed studies into (hetero)arene C–H metalation with these zincates demonstrated that all the constituent parts of the zincate (the amide, the group one metal and the hydrocarbyl group) are vital for enabling (hetero)arene metalations. Furthermore, the different roles the amide and alkyl groups fulfil during C–H metalation using amido-alkyl-zincates have been elucidated, specifically the amide is the kinetic and the hydrocarbyl the thermodynamic deprotonation positions, respectively.^6^ Due to the significant contribution of all components that make up metal-“ate” complexes to controlling reactivity the formation of novel “ate” complexes containing underexplored substituents is important as it has the potential to uncover new reactivity.

One rarely explored class of zincates are those containing a hydride substituent.^1*b*^ The limited reports in this area have focused principally on determining the solution and solid state structures of the hydrido-zincates formed on combining MH or M with R_2_Zn (M = group one metal).^7^ Furthermore, there is only one amido-hydrido zincate^8^ and only one alkoxy-hydrido zincate^9^ well characterised to date to the best of our knowledge. The use of hydrido-zincates in organic transformations is also limited, and in all cases the hydrido-zincates are formed and used *in situ*.^1*b*^ Nevertheless, notable reports from Uchiyama and co-workers using R_2_Zn/MH combinations revealed highly selective reductions with no competing Brønsted basic reactivity.^10^ Since this work hydrido-zincates have remained largely overlooked in organic transformations. Changing the latter would be facilitated by the development of alternative routes to access hydrido-zincates, such as methods that use bench stable hydride sources in place of MH or M (M = group 1 metal).

**Scheme 1:**
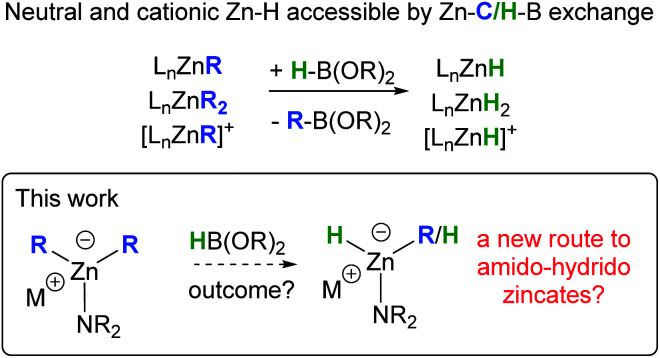
Zn–C/H–B exchange as a route to form zinc-hydrides.

In contrast to anionic zinc hydrides, the chemistry of neutral and cationic molecular zinc hydrides has experienced significant progress, particularly in the past decade.^11^ This includes the development of multiple synthetic routes to access these zinc hydrides, some of the most common being: complexation of metastable ZnH_2_ with ligand(s); substituent exchange reactions between L_*n*_Zn–X and MH_*n*_, or between L_*n*_Zn–OR (or L_*n*_Zn–NR_2_) and R_4−*x*_Si–H_*x*_ or L_*n*_Zn–NR_2_ and R_2_HNBH_3_.^11^ More recently, we reported that a metathesis type reaction between neutral and cationic L_*n*_Zn–R complexes (R = alkyl, aryl, alkenyl or alkynyl) and pinacolborane, HBPin, provides an alternative route to neutral and cationic molecular zinc hydrides (Scheme 1).^12^ While this approach is related to the formation of zinc-hydrides by reaction of zinc alkyls with LiAlH_4_ (reported for both neutral organozinc and alkylzincate complexes),^13^ HBPin is a bench stable hydride source, and the products from Zn–C/H–B exchange, R–BPin, are ubiquitous in synthesis (in contrast to the by-products using LiAlH_4_, *e.g.* [R_4−*X*_AlH_*x*_]^−^). The formal metathesis of organozincates with HBPin represents an unexplored route to form hydrido-zincate complexes concomitant with organoboranes that could underpin future catalytic developments mediated by zincates. Herein we report our studies into assessing the reactivity of amido-organozincates with HBPin as a potential route to form hydrido-zincates.

## Results and discussion

### Towards selective Zn–C/H–BPin exchange

We targeted a heteroleptic alkali–metal amido-organo zincate that would undergo selective Zn–C/H–BPin exchange to form mixed amido-hydrido-zincates in preference to Zn–N/H–BPin exchange. Selective exchange would help avoid formation of insoluble [ZnH_3_]^−^ salts which would form in the presence of excess HBPin if both amido and hydrocarbyl ligands rapidly underwent exchange with HBPin. To expedite the search for such systems, a series of amido-organozincates were made *in situ* following the well-established co-complexation methodology of mixing appropriate monometallic precursors: ZnR′_2_ and a MNR_2_ salt.^1^ Initial studies focused on two sterically demanding, secondary amides, namely hexamethyldisilazide (HMDS) and 2,2,6,6-tetramethylpiperidide (TMP),^14^ which have progressed the chemistry of alkali–metal zincates significantly. The hypothesis was that sufficient amide steric bulk would retard Zn–N/H–BPin exchange. Combining equimolar amounts of ZnEt_2_ (1 M in hexanes) and LiHMDS in benzene followed by the addition of one equivalent of HBPin (Table 1, entry 1) led to effectively complete formation of (HMDS)BPin (by ^11^B NMR spectroscopy, *δ*_11B_ ≈ 26),^15,16^ with effectively no (<2%) EtBPin formation (*δ*_11B_ ≈ 34).^16^ Changing the organozinc reagent from ZnEt_2_ to ZnPh_2_ (entry 2) still led to formation of (HMDS)BPin as the major product. However, PhBPin (*δ*_11B_ ≈ 31)^17^ was present in a greater amount relative to EtBPin in the previous reaction (compare Fig. S2 and S4†) alongside broad resonances between +6 to +8 ppm in the ^11^B NMR spectrum, consistent with [Y_2_BPin]^−^ (Y = combinations of H, Ph or NR_2_).^18^ The observation of more of the desired organoboron product PhBPin (relative to EtBPin) led us to focus further efforts on the reactivity of zincates derived from ZnPh_2_. To explore the effect of varying the group one metal component of the zincate, LiHMDS was replaced with its potassium counterpart (entry 3), but this did not effect a significant change in site of reactivity as the major products remained (HMDS)BPin along with a broad resonance centered at *δ*_11B_ = 6 ppm (a chemical shift closely comparable with previously isolated and well characterised [Y(NR_2_)BPin]^−^ salts).^18^ It should be noted that in none of these reactions with HMDS (or TMP, *vide infra*) are any Zn–H resonances observed by ^1^H NMR spectroscopy indicating that no molecular zinc hydrides are formed.

**Table 1:** Outcomes from initial screening reactions

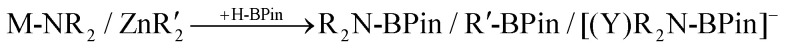			
Entry	MNR_2_	ZnR_2_	Major product^a^
1	LiHMDS	ZnEt_2_	R_2_NBPin
2^b^	LiHMDS	ZnPh_2_	R_2_NBPin
3^c^	KHMDS	ZnPh_2_	R_2_NBPin
4^c^	LiTMP	ZnPh_2_	R_2_NBPin/PhBpin/[R_2_NBPin(Y)]^−^
5^b^^,^^d^	LiTMP	ZnPh_2_	R_2_NBPin/PhBpin
6^c^	KTMP	ZnPh_2_	[R_2_NBPin(Y)]^−^/PhBPin
7	(dpa)Li	ZnPh_2_	PhBPin

a Identity of major product(s) determined by ^11^B NMR spectroscopy.

b 1 eq. of DME added.

c THF was added dropwise until a solution was obtained.

d 2 eq. HBPin used. For more details see ESI.†

The more sterically hindered amide TMP was next explored, with equimolar combinations of LiTMP and ZnPh_2_ addition of one equivalent of HBPin afforded mixtures containing approximately 1 : 1 : 1 ratios of TMPBPin, PhBPin (*δ*_11B_ ≈ 25 and 31, respectively) and a broad resonance centered at 6 ppm (presumably [(NR_2_)YBPin]^−^ anion(s) (Y = H, Ph or NR_2_)) in contrast to outcomes with HMDS/Ph-zincates (where PhBPin is only observed at *ca.* 10%). These findings indicate that increasing the amide steric bulk is beneficial for improving selectivity towards Zn–C/H-BPin exchange, but even with TMP this still does not lead to high selectivity. Addition of further HBPin (now two equivalents in total relative to LiTMP/ZnPh_2_) led to complete consumption of HBPin and formation of an approximately 1 : 1 mix of PhBPin and TMPBPin as the major products (by ^11^B NMR spectroscopy) with minor quantities of borate salts (*e.g.* Li[Ph_*x*_BH_*y*_], *x* + *y* = 4) also observed. This indicates that both Zn–C and Zn–NR_2_ units undergo complete exchange with HBPin, indicating that the undesired formation of insoluble homoleptic hydridozincates will occur with these systems. Similar results were also observed replacing LiTMP with KTMP.

We next turned our attention to replacing monofunctional amides (TMP/HMDS) with multifunctional amides: it was hypothesised that by preparing zincates containing less nucleophilic amides (*e.g.* aniline derived) incorporated into a chelating group could preclude Zn–N/H–BPin exchange. 2,2′-Dipyridylamide (dpa) was selected as it has three Lewis basic N-atoms incorporated within a flexible scaffold, favouring chelation to zinc,^19^ and a significantly lower nucleophilicity amide (potentially disfavouring Zn–N/H–B exchange). Mixing Lidpa with ZnPh_2_ and reacting this with one equivalent of HBPin afforded PhBPin as the major product (entry 7 and Fig. S14†) with no PinB-NR_2_ observed (minor borate resonances at *δ*_11B_ 6–8 ppm were observed consistent with formation of [R_2_N(Y)BPin]^−^ anions, but these were only 20% the intensity of the PhBPin resonance in the ^11^B NMR spectra). Importantly, a new sharp resonance in the region 4.5–5.7 ppm (depending on the solvent) consistent with a Zn–H was observed in the ^1^H NMR spectra suggesting successful formation of a soluble and stable Zn–H species derived from Zn–C/H–BPin exchange. Encouraged by this observation, we set out to identify the constitution of the relevant dpa-based zincates formed prior to addition of HBPin to enable more controlled studies and more insight into the putative hydrido-dpa-zincate.

### Zinc-dpa complexes

**Scheme 2:**
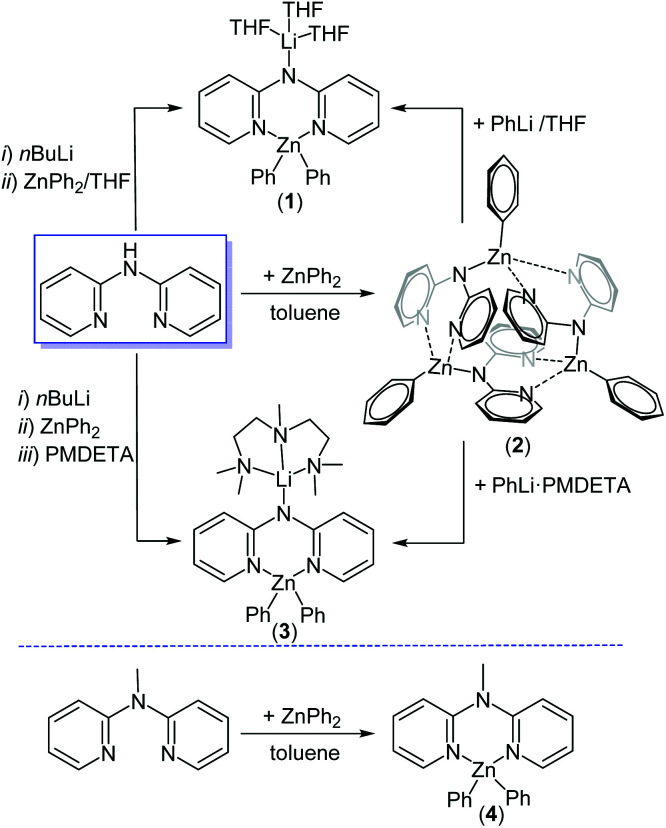
Formation of dpa-Zn complexes **1–4**.

Deprotonation of 2,2′-dipyridylamine with *n*BuLi in hexane followed by addition of a THF-solution of ZnPh_2_ afforded [(THF)_3_Li(μ-dpa)ZnPh_2_] (**1**) in 70% isolated yield (Scheme 2).

Deprotonation of dpaH with ZnPh_2_ in toluene afforded on crystallisation trimeric [(dpaZnPh)_3_] (**2**) in 77% isolated yield, which could be converted into mono-zinc complex **1** by addition of one equivalent of PhLi in THF. Addition of the chelating ligand PMDETA (*N*,*N*,*N*′,*N*′′,*N*′′-pentamethyldiethylenetriamine) during zincate synthesis afforded the PMDETA analogue of **1**, [(PMDETA)Li(μ-dpa)ZnPh_2_] (**3**) in 65% isolated yield. In addition, a neutral monomeric Zn-complex incorporating the dpa scaffold was prepared for reactivity comparison studies (*vide infra*). Adding an equivalent of 2,2′-dipyridyl-*N*-methylamine (Me-dpa) to a solution of ZnPh_2_ afforded adduct [(Me-dpa)·ZnPh_2_] (**4**). Complexes **1–4** were all fully characterised in solution (comparing well with the data previously reported for other dpa-zincates (*e.g.* [(TMEDA)_2_Na_2_(μ-dpa)_2_Zn(^*t*^Bu)_2_], **A**, and [Na(THF)_6_{Zn(^*t*^Bu)_2_(dpa)Zn(^*t*^Bu)_2_}], **B**)^19^ and L_2_ZnPh_2_ systems, *e.g.* TMEDA·ZnPh_2_)^20^ and in the solid state by single crystal X-ray diffraction studies. It should be noted that ^1^H DOSY NMR studies are consistent with **2** persisting as an oligomer in solution by comparison to parameters derived from ^1^H DOSY studies on monomeric **4** (see ESI, Fig. S38 and S39†).

**Fig. 1:**
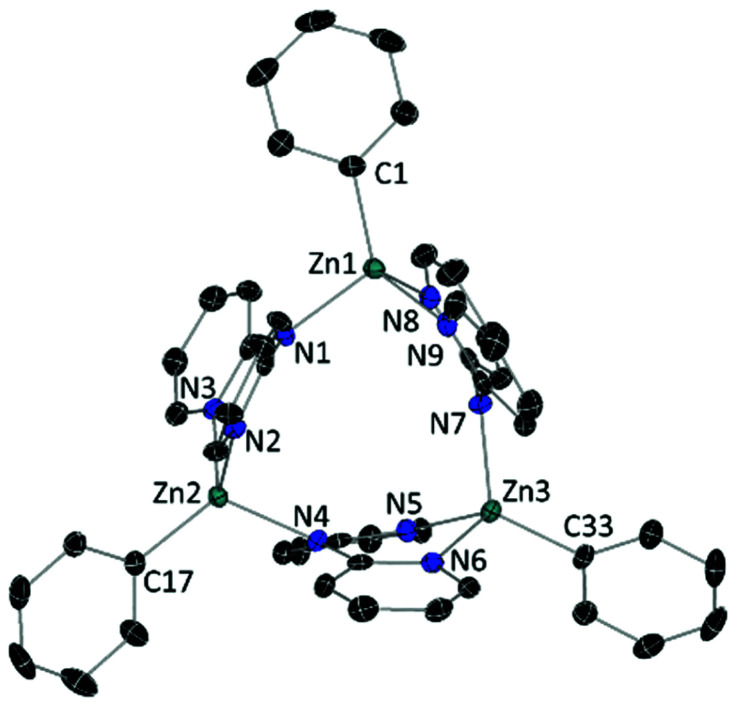
ORTEP-representation of [(dpaZnPh)_3_] (**2**) with ellipsoids at 50% probability level. The disorder component, hydrogen atoms and crystallisation solvent have been omitted for clarity.

In the solid state (Fig. 1) **2** is a trimer in which each dpa assumes an *anti*/*anti* conformation to act as a bridge between neighbouring Zn atoms. Each Zn atom is in a distorted tetrahedral environment, bonded to the N_amido_ of one dpa unit and chelated within the dipyridyl pocket of another dpa, with its coordination completed with a terminal Ph ligand. Within the trimer, the three Zn-atoms define a plane, to which all three dpa ligands are effectively perpendicular, while the Ph rings are 7.20°, 22.40° and 34.87° tilted to the Zn_3_ plane. The Zn–C bond distances are in close agreement with that reported for the dimer [(dpaZn^*t*^Bu)_2_], while the Zn–N bonds are only slightly shortened in **2** (*cf.* for [(dpaZn^*t*^Bu)_2_] Zn–N = 2.116(2) Å; 2.079(2) Å; 2.070(2) Å).^19^

**Fig. 2:**
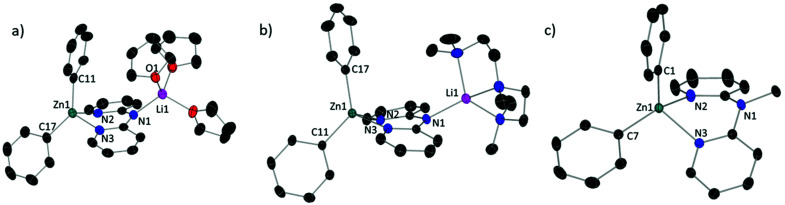
ORTEP representation of (a) [(THF)_3_Li(μ-dpa)ZnPh_2_] (**1**); (b) [(PMDETA)Li(μ-dpa)ZnPh_2_] (**3**) and (c) [(Me-dpa)·ZnPh_2_] (**4**). Ellipsoids are drawn at 50% probability level. Hydrogen atoms omitted for clarity.

In both **1** and **3** (Fig. 2a and b), the anionic dpa ligand is also in an *anti*/*anti* conformation, but now bridges between a donor-capped Li^+^ cation coordinated to the N_amido_ with a ZnPh_2_ unit chelated within the dipyridyl pocket, similar to that observed in zincates **A** and **B**.^19^ Close comparison of **1** and **3** (Table 2) reveal that the tetrahedral environment of Li in **3** is more distorted (*τ*_4_ = 0.7) from ideal than in **1** (*τ*_4_ = 0.9) but the average angles and Li–N_amido_ bond lengths are identical within error. In both complexes Zn is in a distorted N_2_C_2_-tetrahedral environment, displaying essentially identical bond distances, average angles and the extent of distortion from an ideal tetrahedral environment (*τ*_4_). However, the C–Zn–C angle is significantly different at 137.17(8)° for **1** and 128.07(4)° for **3**, the former angle is even greater than the Zn^*t*^Bu_2_ species, **A** (130.31(7)°) and **B** (131.9(1)°),^19^ where a larger steric effect from the ^*t*^Bu groups would be expected. Previous studies have shown that the narrowing of the C–Zn–C bond angle in diorganylzinc complexes with C_2_ZnN_2_ coordination environments increases the Lewis acidity of Zn-centre.^21^ However, based on reactivity studies and DFT calculations (*vide infra*) this C–Zn–C angle disparity does not indicate a significantly different electronic structure for **1** and **3**. Finally, the tetrahedrally coordinated Zn centre in **4** (Fig. 2c) exhibits very similar parameters to those found in **3**, with a C–Zn–C angle of 126.20(7)°.

**Table 2:** Selected bond distances and angles for complexes **1–5**

	**1**	**2**	**3**	**4**	**5**
Zn–C (Å)	2.004(2)	1.987(2)–1.992(2)	2.0088(11)	1.9988(18)	1.973(4)
	2.010(2)		2.0018(10)	1.9964(19)	1.971(5)
Zn–N_py_ (Å)	2.0952(16)	2.035(2)–2.056(2)	2.0966(9)	2.1032(15)	2.059(4)
	2.0985(17)		2.1071(9)	2.1309(15)	2.064(4)
Zn–N_amide_ (Å)	—	2.047(2)–2.063(2)	—	—	—
C–Zn–C (°)	137.17(8)	—	128.07(4)	126.20(7)	130.60(19)
Li–N_amide_ (Å)	2.047(4)	—	2.028(2)	—	2.028(9)

**Fig. 3:**
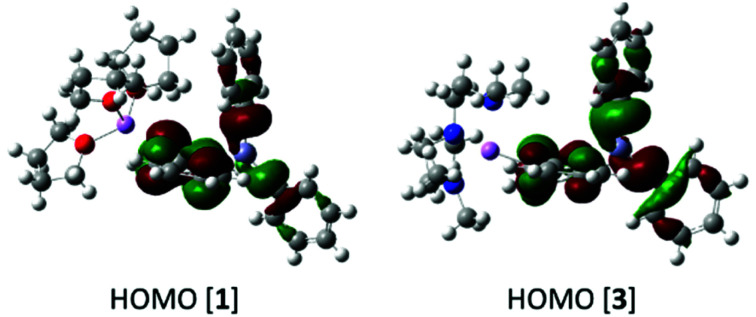
HOMO of **1** and **3**.

The solid state geometries of complexes **1** and **3** were used for single point calculations at the B3PW91/6-311G(d,p) (H, Li, C, N, O)/lanl2dz (Zn)//PCM(THF) level (PCM = polarisable continuum model). Notably, these calculations show that despite the difference in the C–Zn–C bond angle between **1** and **3** the two structures possess effectively identical NBO charge distributions (*e.g.* axial *ipso*_Ph_C̲ charge = −0.499*e* and −0.506*e* for **1** and **3**, respectively) and frontier orbital energies and character. For example, the HOMO of **1** and **3** (Fig. 3) both contain Zn–C σ bonding character and are effectively identical in energy (−5.02 eV and −5.06 eV), while the charges at Zn are within 0.006*e* in **1** and **3** (+1.169 and +1.163*e*, respectively). The most notable difference is in the NBO charge at lithium which is +0.875*e* for **1** and +0.846*e* for **3**; this small difference is presumably due to a more electrostatic dominated interaction between _(THF)_O_3_–Li (in **1**) than between _(PMDETA)_N_3_–Li in **3**.

### Reactivity studies of Zn-dpa complexes

**Fig. 4:**
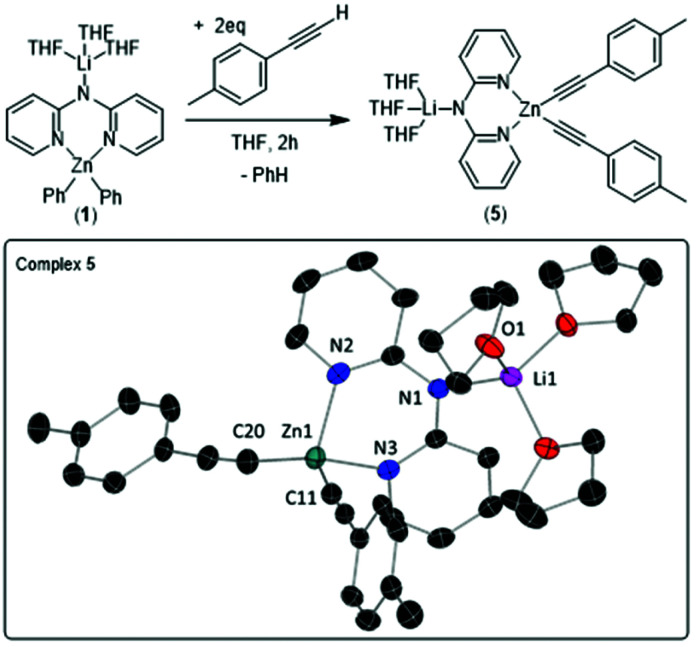
Formation of [(THF)_3_Li(μ-dpa)Zn(C≡CC_6_H_4_Me)_2_] (**5**). ORTEP representation of **5** with ellipsoids at 30% probability level and hydrogen atoms omitted for clarity.

To determine their relative reactivity, the various zinc complexes were applied in selected transformations. We started by investigating the deprotonation of two equivalents of the terminal alkyne 4-ethynyltoluene. This was slow using neutral zinc complex **4**, only proceeding to 41% after 24 h at room temperature. Contrastingly, **1** completely consumed all two equivalents of the terminal alkyne within two hours at room temperature affording the new bimetallic complex [(THF)_3_Li(μ-dpa)Zn(C≡CC_6_H_4_Me)_2_] (**5**, Fig. 4) as the major product. The disparity between the reactivity of **1** and **4** is an indicator of zincate character in **1**.

Single crystal X-ray diffraction analysis of **5** revealed it to have an essentially isostructural Li-dpa-Zn core to **1** and **3** (Table 2). The most notable metric is the C–Zn–C angle which is different between **1** and **5**, with **5** having a compressed C–Zn–C angle (130.60(19)°) relative to that in **1** (137.17(8)°), despite both having an identical lithium environment. Solution studies on **5** were precluded due to the poor solubility of crystals of **5**, even in THF. Furthermore, during reactions to form **5** small amounts of two other alkynyl-containing species also were observed which could correspond to different dpaZn structural motif(s) or to products obtained by alkyne deprotonation partially proceeding through Li–N_amido_ fragment. The same minor species are observed even when **5** was prepared by reacting dialkynylzinc with Lidpa. Finally, compound **3** reacts with two equivalents of 4-ethynyltoluene in a comparable manner to **1**, with all alkyne consumed within 2 hours using **3**, indicating similar reactivity between **1** and **3** in metalation reactions supporting the comparable electronic structures in the Ph_2_Zn-dpa units of **1** and **3** found by DFT calculations.

**Scheme 3:**
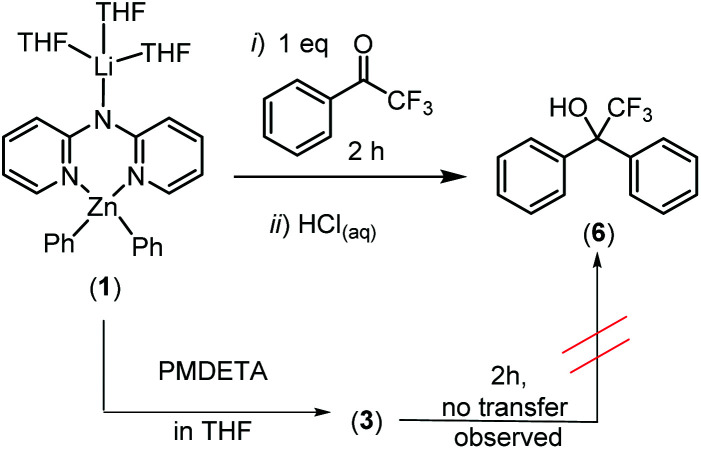
Phenyl transfer with zincates **1** and **3**.

We next turned our attention to phenyl transfer to α,α,α-trifluoroacetophenone (to facilitate reaction monitoring by ^19^F NMR spectroscopy). While neutral complexes **2** and **4** afforded no transfer of a phenyl group (by ^19^F NMR spectroscopy) even after days, complex **1** displayed significant reactivity and afforded 2,2,2-trifluoro-1,1-diphenylethanol (**6**) in 71% yield after only 2 h reaction time at room temperature (Scheme 3). This observation is in line with previous reports on the sluggish reactivity of neutral diorganozinc reagents towards aldehydes and ketones that can be enhanced by formation of zincates.^22^ This further indicates that **1** displays zincate type reactivity. Monitoring the reaction of **1**/α,α,α-trifluoroacetophenone *in situ* (prior to work up) revealed formation of resonances consistent with **2** as the only observed zinc containing by-product from phenyl transfer. In contrast to fast phenyl transfer with **1**, compound **3** led to no phenyl transfer to α,α,α-trifluoroacetophenone after 2 h by ^19^F NMR spectroscopy (though slow phenyl transfer does occur at longer reaction times). Given the similar calculated electronic structures and comparable reactivity towards terminal alkynes observed for **1** and **3** this disparity was surprising. It is attributed to the lithium centre in **1** being more accessible for coordination of a carbonyl and that this Lewis acid activation of the ketone accelerates phenyl transfer (consistent with observations from reported DFT calculations on lithium-zincates reacting with carbonyls).^10^ In contrast, PMDETA would bind lithium more strongly and disfavour coordination of the ketone to lithium. A similar observation has been previously reported where TMEDA addition retards nucleophile transfer from a lithium zincate to carbonyl containing electrophiles.^23^ This hypothesis is supported further by the addition of PMDETA to **1** resulting in formation of **3**, confirming the stronger binding affinity that PMDETA has towards lithium in these lithium zincates relative to three molecules of THF.

With confirmation of zincate type reactivity exhibited by **1** in hand we explored next the reactivity of **1** towards HBPin targeting an amido-hydrido-zincate by Zn–C/HBPin exchange.

### Reactivity of **1** towards pinacolborane

#### Equimolar **1**/HBPin reactivity

**Fig. 5:**
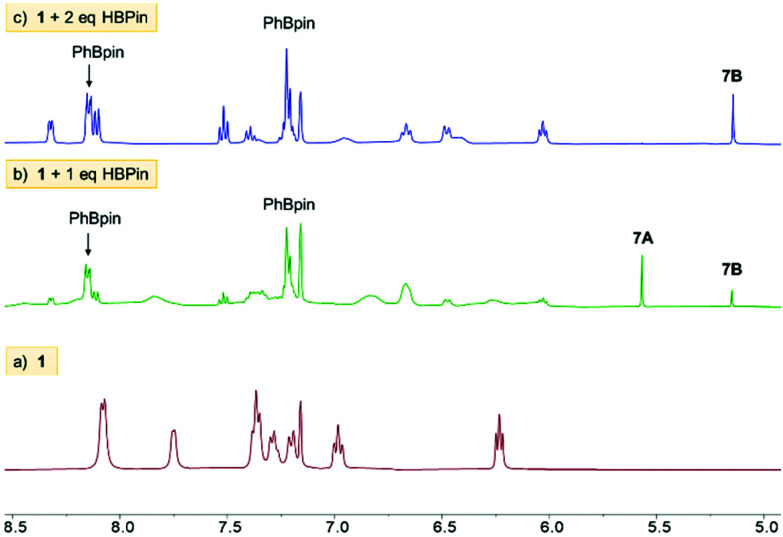
Stacked ^1^H NMR spectra in C_6_D_6_ at room temperature (5.0–8.5 ppm region) comparing outcomes of reaction with HBPin: (a) starting zincate **1**; (b) **1** reacted with 1 eq. of HBPin; (c) **1** reacted with 2 eq. of HBPin.

**Fig. 6:**
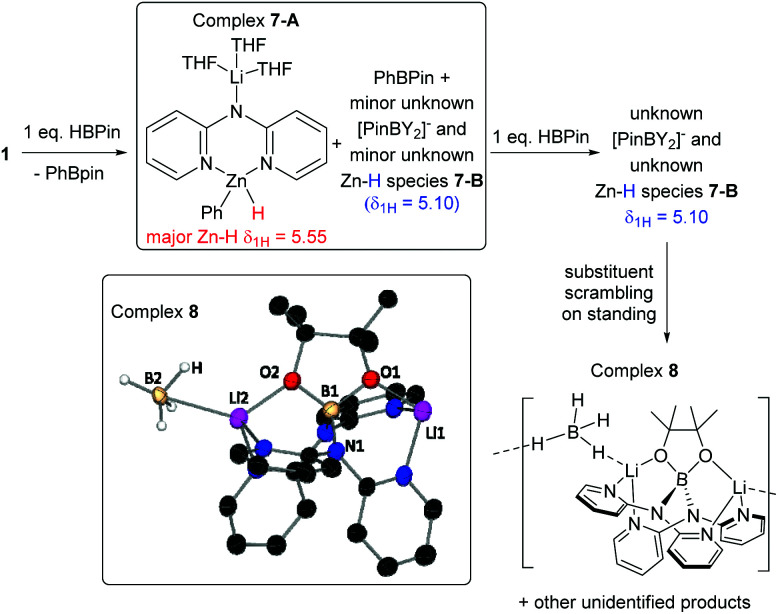
Observed products formed on reaction of **1** with one and two equivalents of HBPin. ORTEP representation of decomposition product **8** with ellipsoids at 20% probability level and with hydrogen atoms (except B–H) omitted for clarity.

The combination of **1** with one equivalent of HBPin at ambient (in d_6_-benzene) or low (in toluene) temperature led to reaction mixtures containing two sharp singlets at *δ*_1H_ 5.55 (major) and 5.10 (minor) assigned as Zn–*H̲* resonances (they do not correspond to C–H moieties based on HSQC NMR experiments and are not observed in reactions using DBPin in place of HBPin). Along with these the ^1^H NMR spectra contained broad aromatic resonances for dpa and Zn–Ph, sharper aromatic resonances corresponding to PhBPin, and THF resonances that remain shifted relative to free THF consistent with THF coordination to Li^+^ as observed in **1**. The ^11^B NMR spectra showed PhBPin as the major product and a minor product associated with a very broad resonance centered at 5 ppm. This very broad ^11^B resonance is in the region expected for [PinB(Y)_2_]^−^, Y = Ph and/or NR_2_.^18,25^ The observation of PhBPin as the major boron containing product and new Zn–H species formed from equimolar **1**/HBPin suggests successful formation of a mixed amido-phenyl-hydrido-zincate derived from **1**, tentatively assigned as [(THF)_3_Li(μ-dpa)ZnPh(H)] (**7-A**) (Fig. 5 and 6). ^7^Li NMR spectra for **7-A** do not show any Li–H scalar coupling in contrast to the only (to our knowledge) other previously reported lithium amido-hydrido zincate.^8^ Repeated attempts to isolate or crystallise any Zn–H species from these reaction mixtures were unsuccessful in our hands.

Mass spectrometry on equimolar **1**/HBPin reactions only showed fragmentation products such as [dpaZnPh]˙^+^. It should be noted that combining HBPin/**1** in aromatic solvents also formed some insoluble material, however performing these reactions in more polar solvents, *e.g.* THF, led to more complex mixtures by NMR spectroscopy, (though PhBPin is still the dominant boron containing species when reactions are performed in THF), thus aromatic solvents were the preferred reaction media.

On standing in benzene **1**/HBPin mixtures slowly produced more insoluble material over several days, with the only significant species ultimately left in solution being PhBPin. The amount of PhBPin formed was quantified *versus* an internal standard which revealed >90% of HBPin had converted into PhBPin indicating that Zn–Ph/H–BPin exchange is the dominant outcome on combining equimolar **1** and HBPin. Finally, attempts to access the putative amido-hydrido-zincate **7-A** by other routes were unsuccessful (see ESI† for more details).

#### 1 : 2 combinations of **1** and HBPin

**Fig. 7:**
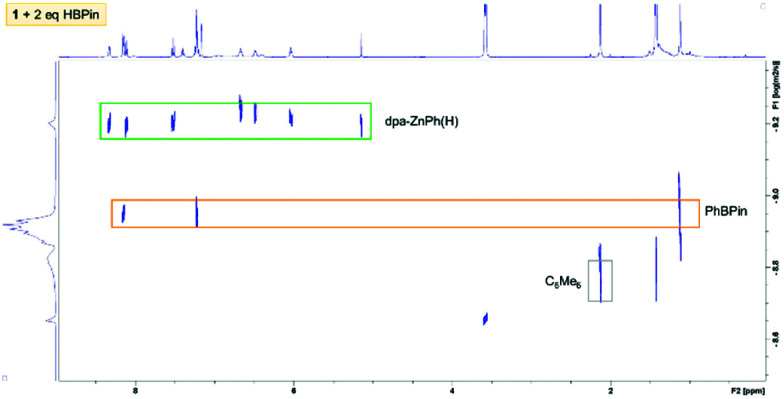
^1^H DOSY NMR spectrum of reaction mixture **1** with 2 eq. of HBPin in C_6_D_6_ at room temperature after 45 min.

Complex **1** was combined with two equivalents of HBPin in benzene, with arene solvents again essential as the use of THF leads to more complex outcomes (including rapid formation of [BH_4_]^−^, [PhBH_3_]^−^ and other new B–H species). Combining in d_6_-benzene a 1 : 2 ratio of **1** : HBPin led to complete consumption of HBPin with the ^1^H NMR spectrum containing resonances consistent with a single zinc-dpa containing complex, termed **7-B**, with key resonances including: a 1H integral (relative to dpa) Zn–H̲ at 5.10 ppm, and resonances for a single Zn–Ph unit, along with one set of well-defined dpa resonance (Fig. 5). A HMBC NMR experiment contained a cross peak between the *ipso* Zn–C_Ph_ and the Zn–H confirming they are bound to the same zinc centre. It should be noted that dpa also remains bound to the Zn(Ph)H moiety in this complex based on ^1^H DOSY NMR studies (Fig. 7). An internal standard was added and integration revealed that the dpaZn(Ph)H species **7-B** corresponded to 70% of the amount of **1** originally present, confirming it is the major product, but that some dpa containing material had precipitated. The addition of three equivalents of HBPin to **1** led to the same soluble zinc complex **7-B**, with one equivalent of HBPin left unreacted (by ^11^B NMR spectroscopy). This confirms that dpa ligation of zinc disfavours formation of insoluble homoleptic hydridozincates and that a 2 : 1 reaction stoichiometry between HBPin and **1** leads to the **7-B** species. The ^11^B NMR spectrum of the 1 : 2 **1** /HBPin reaction showed only PhBPin and a very broad resonance centered at 5 ppm. As **7-B** could not be isolated in our hands, it can only be tentatively assigned as the analogue of **7-A** where a YBPin molecule is bound to the anilido N in place of [Li(THF)_3_]^+^.

On standing the *δ*_1H_ 5.10 resonance slowly decreases in intensity, concomitant with the very broad *δ*_11B_ 5 ppm resonance being replaced with several other boron resonances. The most intense new resonances correspond to [BH_4_]^−^ species (*δ*_11B_ = −40 ppm quintet) and a sharper *δ*_11B_ resonance at +5 ppm (consistent with [diamido–BPin]^−^).^18^ This decomposition is consistent with previous work on the reactivity of anionic nucleophiles with HBPin, that form metastable [HBPin(Y)]^−^ anions that undergo substituent scrambling, this indicates that the 1 : 2 **1**/HBpin reaction mixture contains significant [(Y)HBPin]^−^.^24,25^ Attempts to crystallise these 1 : 2 reaction mixtures to isolate the Zn–H species **7-B** also were unsuccessful, with the only species isolated being a small quantity of the redistribution product, **8**, containing both [BH_4_]^−^ and [(R_2_N)_2_BPin]^−^ moieties consistent with the +5 and −40 ppm resonances observed in the ^11^B NMR spectra. The PMDETA analogue, **3**, displayed comparable outcomes to combinations of **1** with HBPin, undergoing Zn–Ph/H–BPin metathesis (albeit more slowly than **1**) to give PhBPin and Zn–H **7-B** (*δ*_1H_ 5.1 ppm), with scrambling to form BH_*x*_ species (*x* > 1) also observed on standing. Finally, attempts to use catecholborane, HBCat, in place of HBPin led to much more complex outcomes, rapidly forming new boron containing compounds including: [BCat_2_]^−^, species containing BH_2_ and BH_3_ units and [BH_4_]^−^. Single crystals of a substituent scrambled product, [dpa_3_Zn_2_][BCat_2_] (**9**), were isolated from these mixtures (see ESI, Fig. S132†). These observations indicate that both the borane and dpaZn fragments can undergo exchange processes, and that these occur much more rapidly with BCat derived species.

#### Reactivity studies of the Zn–H complexes

**Scheme 4:**
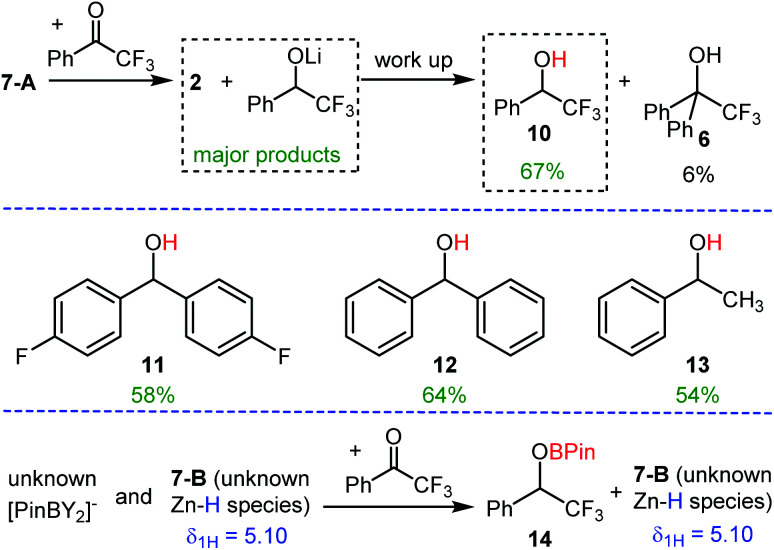
Top and bottom, disparate outcomes on reaction of Zn–H species **7-A** and **7-B** derived from **1**/HBPin with α,α,α-trifluoroacetophenone. Middle, alcohols formed by carbonyl reduction using **7-A**.

As the reaction of **1** with one eq. of HBPin gave two different (dpa)Zn–H containing species under a range of conditions that frustrated isolation the identity of these was probed in reactivity studies. The initial reaction was the hydrofunctionalisation of α,α,α-trifluoroacetophenone. In these reactions the presence of [HBPin(Y)]^−^ would lead to rapid hydroboration as previously reported,^24^ in contrast with a hydrido-zincate (such as **7-A**) hydrometalation of the ketone would dominate, leading to formation of the lithium or zinc alkoxide (which have distinct *δ*_19F_ relative to the hydroboration product).^10^ The reaction mixture derived from 1 : 1 **1**/HBPin on addition of α,α,α-trifluoroacetophenone led to consumption of the *δ*_1H_ 5.55 ppm Zn–H species and formation of **2** as the major zinc containing species. After work up 2,2,2-trifluoro-1-phenylethanol, **10**, derived from hydride transfer from zinc was the major product with minimal product observed derived from phenyl transfer, **6** (Scheme 4). This is consistent with previous studies using hydrido zincates that demonstrated preferential hydride transfer over hydrocarbyl.^10^ Monitoring the reaction *in situ* revealed no significant ketone hydroboration (product **14**, Scheme 4) had formed by ^19^F NMR spectroscopy, precluding the presence of any significant amount of [H(Y)BPin]^−^ anion in the 1 : 1 reaction mixture. This further supports hydrido-zincate **7-A** being the major product derived from a 1 : 1 **1**/HBPin combination, and that **7-A** can react selectively as a source of hydride. **7-A** was also effective for the reduction of a number of other ketones, including an enolisable ketone, affording alcohols **11–13** in reasonable yield.

In contrast, the combination of the reaction mixture derived from 1 : 2 **1**/HBPin with one equivalent of α,α,α-trifluoroacetophenone led to formation of a significant quantity of the hydroboration product **14** (by ^19^F NMR spectroscopy), indicating the presence of significant [(Y)HBPin]^−^ species. Furthermore, the Zn–H resonance at 5.10 ppm is not consumed on addition of this ketone, indicating that ketone reduction is achieved by a B–H species not a Zn–H complex. Note, in a control reaction using HBPin under identical conditions no hydroboration of α,α,α-trifluoroacetophenone is observed. The formation of significant hydroboration product **14**, combined with the spectroscopic data, suggests that on using two equivalents of HBPin (with respect to **1**) the outcome involves a single Zn–C/H–BPin exchange followed by formation of a [H(Y)BPin]^−^ species that is metastable (towards substituent redistribution).

## Conclusions

In summary, controlling selectivity on the addition of pinacolborane to amido-containing organozincates is challenging, with three outcomes occurring, often in competition: (i) Zn–N/H–BPin exchange; (ii) Zn–C/H–BPin exchange; (iii) transfer of an anionic group from the zincate to HBPin to form [(Y)HBPin]^−^ species. The use of dipyridylamide (dpa) to ligate zinc produces zincates where the two pyridyl nitrogens bind Zn while the anilido nitrogen binds Li. The (donor)Li(dpa)ZnPh_2_ complexes exhibit zincate type reactivity, and in reaction with pinacolborane lead to improved selectivity for Zn–C/H–BPin exchange and no over-reaction (to from [ZnH_3_]^−^) on addition of excess HBPin, presumably due to the lower nucleophilicity of the anilido N and the chelate effect. This enables the *in situ* synthesis of a mixed amido-hydrido zincate **7-A** as the major product, with initial reactivity studies using **7-A** demonstrating it functions as a selective hydride transfer agent. However, selectivity during the Zn–C/H–BPin exchange step still needs to be improved for catalytic applications. Furthermore, the addition of excess pinacolborane results in formation of both zinc hydride **7-B** and [(Y)HBpin]^−^, the latter which are unstable with respect to substituent scrambling.

## Conflicts of interest

There are no conflicts to declare.

## Supplementary Material

DT-050-D1DT02580E-s001

DT-050-D1DT02580E-s002
