# Supplementary material for: Formation of a hydride containing amido-zincate using pinacolborane
Source: Dalton Trans. 2021 Sep 15;50(39):14018–26. doi: 10.1039/d1dt02580e (PMC8507400; doi:10.1039/d1dt02580e)
Supplement: DT-050-D1DT02580E-s001 [file DT-050-D1DT02580E-s001.pdf]

Electronic Supplementary Material (ESI) for Dalton Transactions.
This journal is © The Royal Society of Chemistry 2021

Supporting information for

Formation of a hydride containing amido-zincate using pinacolborane

Marina Uzelac, Kang Yuan, Gary S. Nichol, Michael J. Ingleson*

School of Chemistry, University of Edinburgh, Edinburgh, EH9 3 FJ, UK

Contents

1. General considerations 2
2. Scoping alkali-metal zincates towards Zn-C/H-B metathesis 2
2.1 Reaction of LiHMDS and Et_2Zn with HBPin 2
2.2 Reaction of LiHMDS , $ZnPh_2$ and DME with HBPin 3
2.3 Reaction of KHMDS and $ZnPh_2$ with HBPin 5
2.4 Reaction of LiTMP, $ZnPh_2$ and THF with HBPin 6
2.5 Reaction of LiTMP, $ZnPh_2$ and DME with 2 equivalents of HBPin 8
2.6 Reaction of KTMP and $ZnPh_2$ with HBPin 9
2.7 Reaction of Lidpa and $ZnPh_2$ with HBPin 11
3. Synthesis and characterisation of complexes **1** – **4** 12
3.1 [$(THF)_3$Li(μ-dpa)$ZnPh_2$] (**1**) 12
3.2 [$(dpaZnPh)_3$] (**2**) 19
3.3 [(PMDETA)Li(μ-dpa)$ZnPh_2$] (**3**) 21
3.4 [(Me-dpa)·$ZnPh_2$] (**4**) 24
3.5 ^{1}H DOSY NMR comparison of **2** and **4** 26
3.6 Conversion of **1** to **3** by addition of PMDETA in THF solution 27
4. Reactivity studies 30
4.1 Deprotonation of terminal alkyne and isolation of **5** 30
4.2 Arylation of α,α,α-trifluoroacetophenone and synthesis of **6** 33
5. Reactivity studies on formation of hydride species 40
5.1 Reactivity of **1** towards HBPin 40
5.2 Reactivity of **1** towards HBCat 56
5.3 Reactivity of **1** towards silane 58
5.4 Reactivity of **3** towards HBPin 60
5.5 Reactivity of **2** towards HBPin 65
5.6 Reactivity of **4** towards HBPin 68
5.7 Reactivity of hydride species towards ketones 70
6. X-Ray Crystallography 82
7. Computational details 85

8. References..85

1. General considerations

Unless otherwise indicated all manipulations were conducted under inert conditions either using standard Schlenk techniques or in a MBraun UniLab glovebox (< 0.1 ppm H_2O / O_2). D_6-benzene and d_8-THF were dried over CaH_2 and Na respectively, and distilled prior to storage over 3 Å molecular sieves. Toluene was dried using an Innovative Technology SPS system and stored over activated molecular sieves. LiTMP,[1] KTMP[2] and N-methyl-2,2'-dipyridylamine[3] were prepared according to literature procedures. LiHMDS was prepared by deprotonation of hexamethyldisilazane (HMDS(H)), and KHMDS was prepared by metathesis from LiHMDS and KO*t*Bu in hexanes. Unless otherwise stated all other compounds were purchased from commercial sources and used as received. NMR spectra were recorded on Bruker Avance III 400 MHz and 500 MHz spectrometers. Chemical shifts are reported as dimensionless δ values and are frequency referenced relative to residual protio- impurities in the NMR solvents for ^{1}H and $^{13}C\{^{1}H\}$ respectively, while ^{11}B, ^{19}F and ^{7}Li shifts are referenced relative to external $BF_3{\cdot}Et_2O$, hexafluorobenzene, and LiCl in D_2O, respectively. Coupling constants J are given in Hertz (Hz) as positive values regardless of their real individual signs. The multiplicity of the signals are indicated as "s", "d", "t" "q" "pent", "sept" or "m" for singlet, doublet, triplet, quartet, pentet, septet or multiplet, respectively. ^{1}H-^{7}Li HOESY spectra were recorded using a Bruker PRO 500 spectrometer equipped with RT BB probe. 48 scans were recorded with mixing time of 150 ms.

Electrospray ionization (ESI) measurements were performed at the Scottish Instrumentation and Resource Centre for Advanced Mass Spectrometry (SIRCAMS) based in the School of Chemistry at the University of Edinburgh. High resolution mass spectra were recorded on a Bruker Daltonics 12T SolariX Fourier Transform Ion Cyclotron Resonance Mass Spectrometer (FT-ICR-MS). Elemental analyses were carried out by Elemental Analysis Ltd., measured in duplicate.

2. Scoping alkali-metal zincates towards Zn-C/H-B metathesis

2.1 Reaction of LiHMDS and Et_2Zn with HBPin

Lithium bis(trimethylsilyl)amide (33 mg, 0.2 mmol, 1 eq) was loaded into a J Young's NMR tube and dissolved in C_6D_6 (0.5 mL). Diethylzinc (1 M solution in hexanes, 0.2 mL, 0.2 mmol, 1 eq) followed by addition of pinacolborane (29 µL, 0.2 mmol, 1 eq). After 1 h stirring at room temperature, the reaction mixture was analysed by ^{1}H and ^{11}B NMR spectroscopies which showed predominant formation of PinB-N($SiMe_3$)$_2$ species (Figures S1-S2).

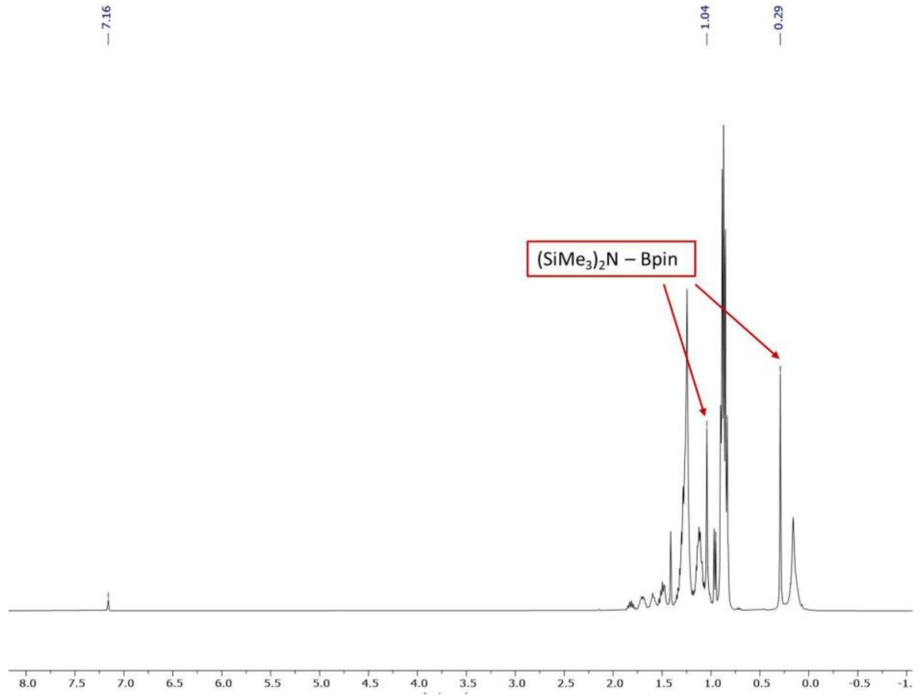

Figure S1. ^{1}H NMR spectrum of reaction mixture in C_6D_6 / hexanes at room temperature.

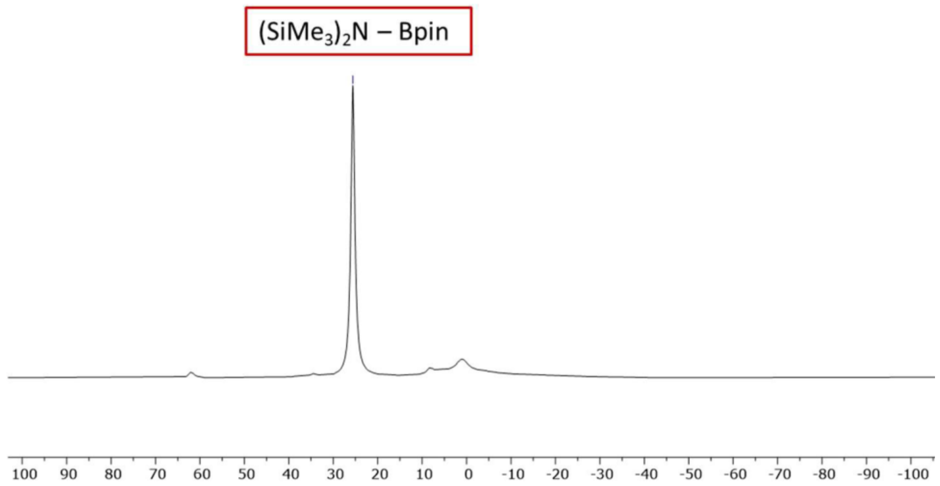

Figure S2. ^{11}B NMR spectrum of reaction mixture in C_6D_6 / hexanes at room temperature.

2.2 Reaction of LiHMDS , $ZnPh_2$ and DME with HBPin

Lithium bis(trimethylsilyl)amide (33 mg, 0.2 mmol, 1 eq) was loaded into a J Young's NMR tube and dissolved in C_6D_6 (0.5 mL). Diphenylzinc (44 mg, 0.2 mmol, 1 eq) was added resulting in the formation of a white precipitate. After 15 min 1,2-dimethoxyethane (DME) was added as a co-solvent (21 µL, 0.2 mmol, 1 eq) affording a homogenous solution. Pinacolborane (29 µL, 0.2 mmol, 1 eq) was added and after 1 h stirring at room temperature, the reaction mixture was analysed by ^{1}H and ^{11}B NMR spectroscopies (Figure S3-S4) which showed predominant formation of pinB-N$(SiMe_3)_2$ species.

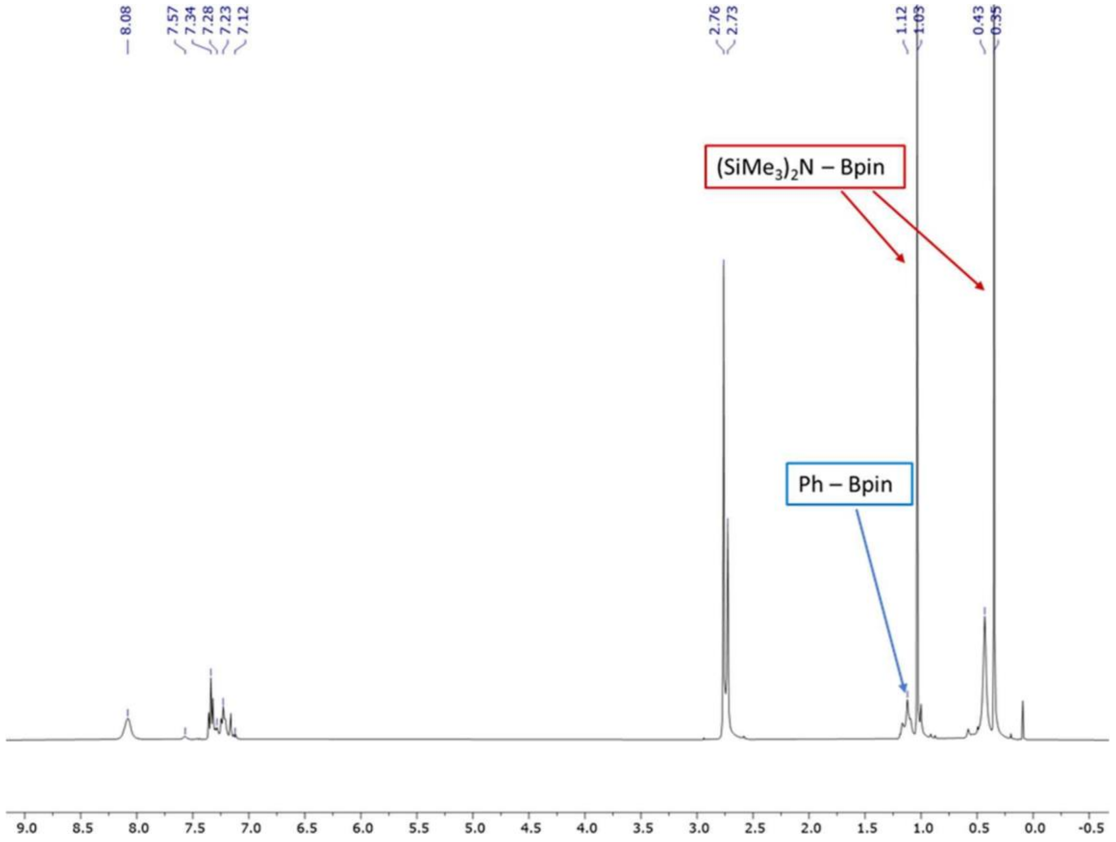

Figure S3. ^{1}H NMR spectrum of reaction mixture in C_6D_6 (+ 21 μl DME) at room temperature.

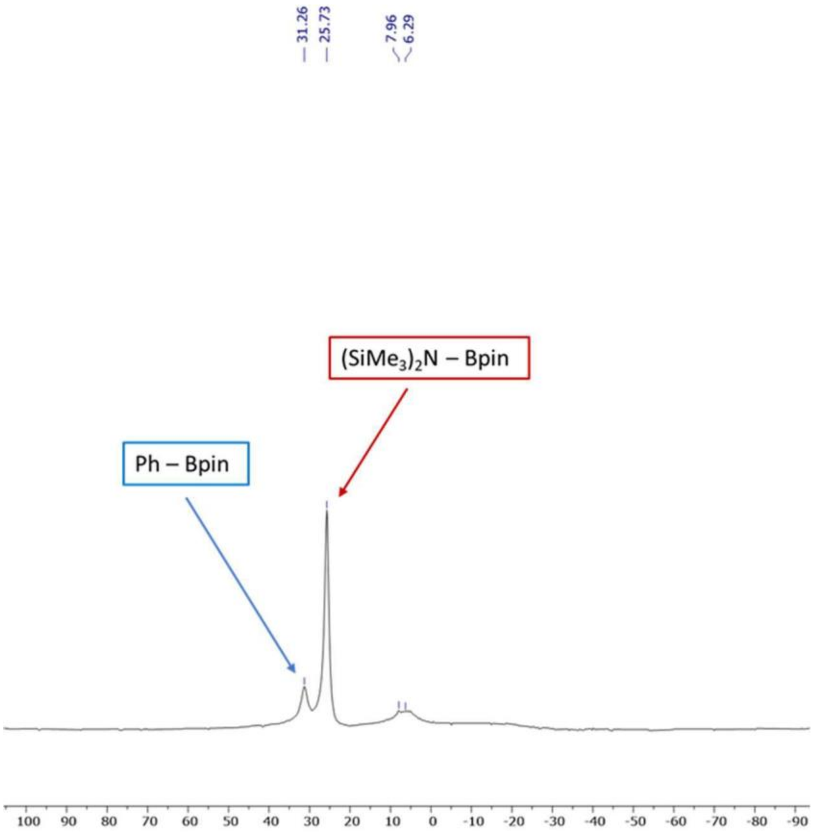

Figure S4. ^{11}B NMR spectrum of reaction mixture in C_6D_6 (+ 21 μl DME) at room temperature.

2.3 Reaction of KHMDS and $ZnPh_2$ with HBPin

Potassium bis(trimethylsilyl)amide (20 mg, 0.1 mmol, 1 eq) and diphenylzinc (22 mg, 0.1 mmol, 1 eq) were loaded into a J Young's NMR tube and suspended in C_6D_6 (0.5 mL). THF was dropwise added until a homogeneous solution was obtained. Pinacolborane (14.5 µL, 0.1 mmol, 1 eq) was added and after 2 h stirring at room temperature, the reaction mixture was analysed by ^1H and ^{11}B NMR spectroscopies which showed formation of both PhBPin and pinB-N($SiMe_3$)$_2$ species, but with pinB-N($SiMe_3$)$_2$ in greater amount (Figure S5-S6).

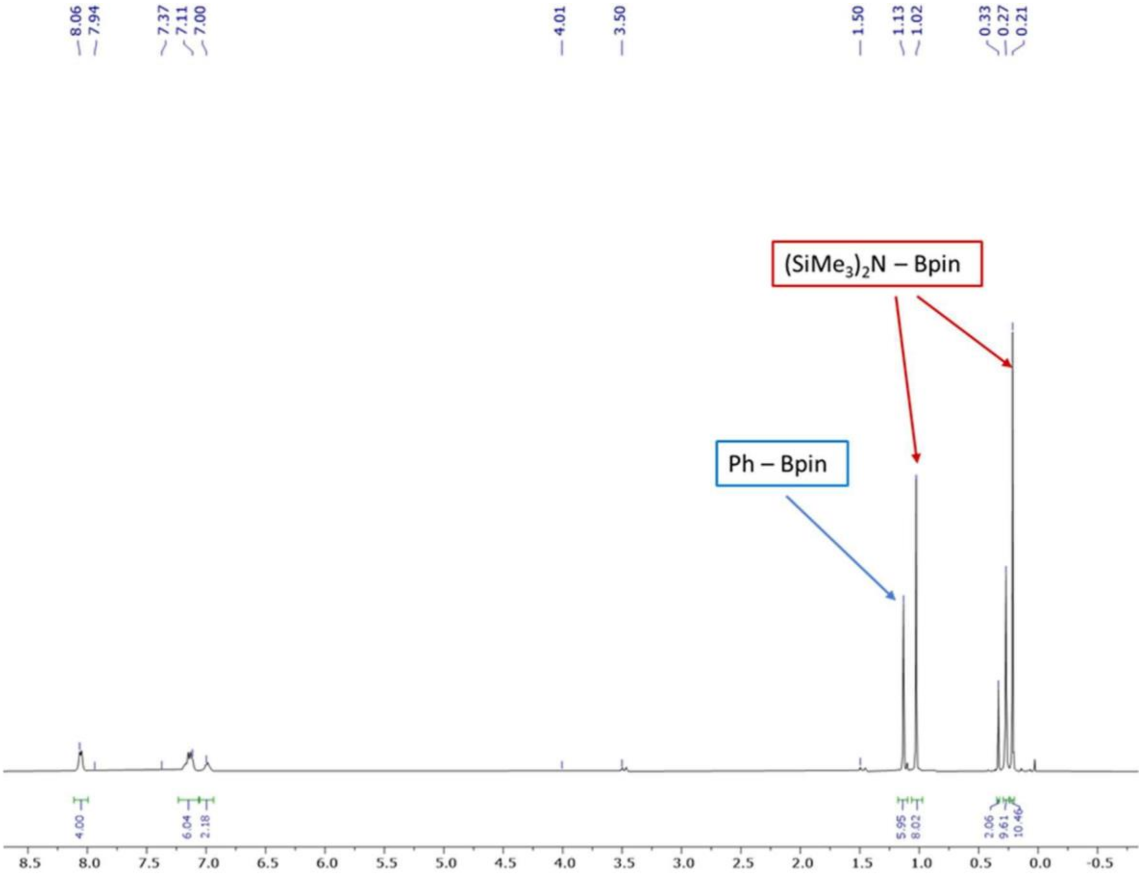

Figure S5. ^1H NMR spectrum of reaction mixture in C_6D_6 (with THF) at room temperature.

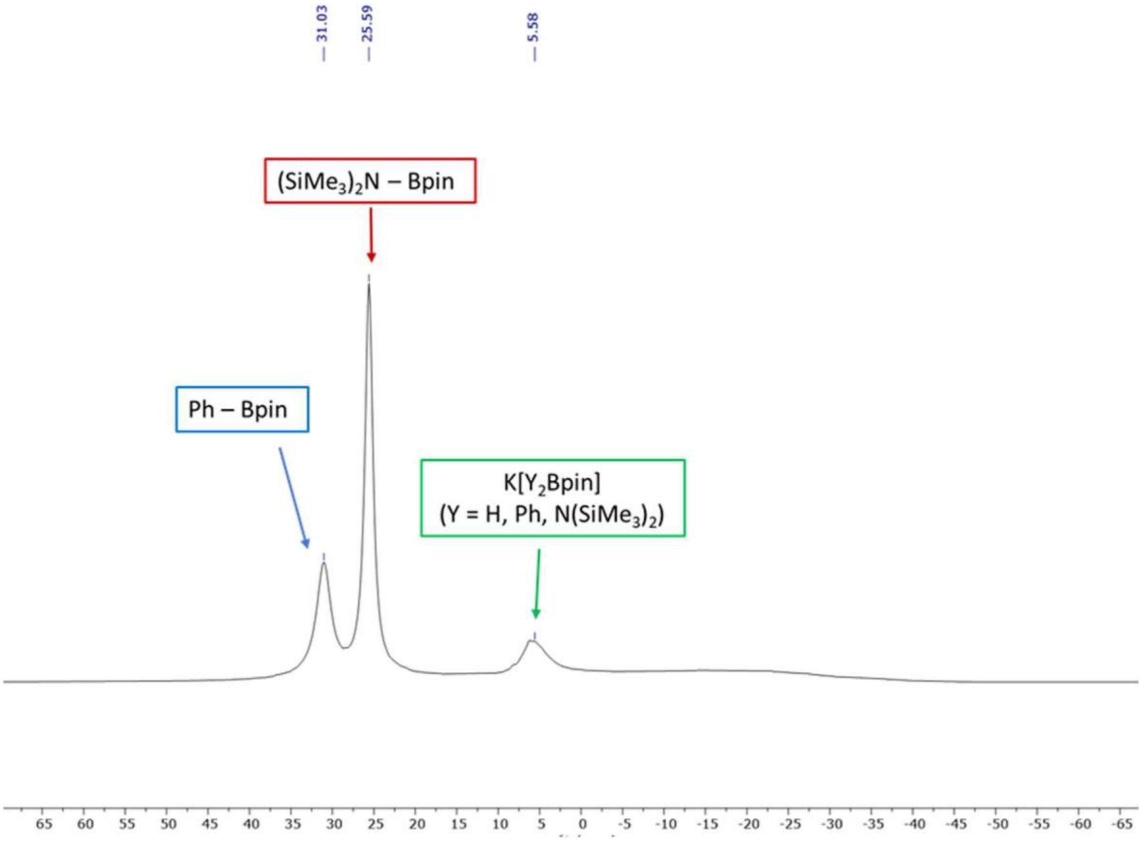

Figure S6. ^{11}B NMR spectrum of reaction mixture in C_6D_6 (with THF) at room temperature.

2.4 Reaction of LiTMP, $ZnPh_2$ and THF with HBPin

Lithium 2,2,6,6-tetramethylpiperidide (29 mg, 0.2 mmol, 1 eq) and diphenylzinc (44 mg, 0.2 mmol, 1 eq) were loaded into a J Young's NMR tube and suspended in C_6D_6 (0.5 mL). After 15 min THF was added as a co-solvent (0.1 mL) affording a solution. Pinacolborane (29 µL, 0.2 mmol, 1 eq) was added and after 1 h stirring at room temperature, the reaction mixture was analysed by ^1H and ^{11}B NMR spectroscopies which showed formation of both pinB-TMP, pinB-Ph and Li[Y_2BPin] type species in approximately equal amounts (Figures S7-S8).

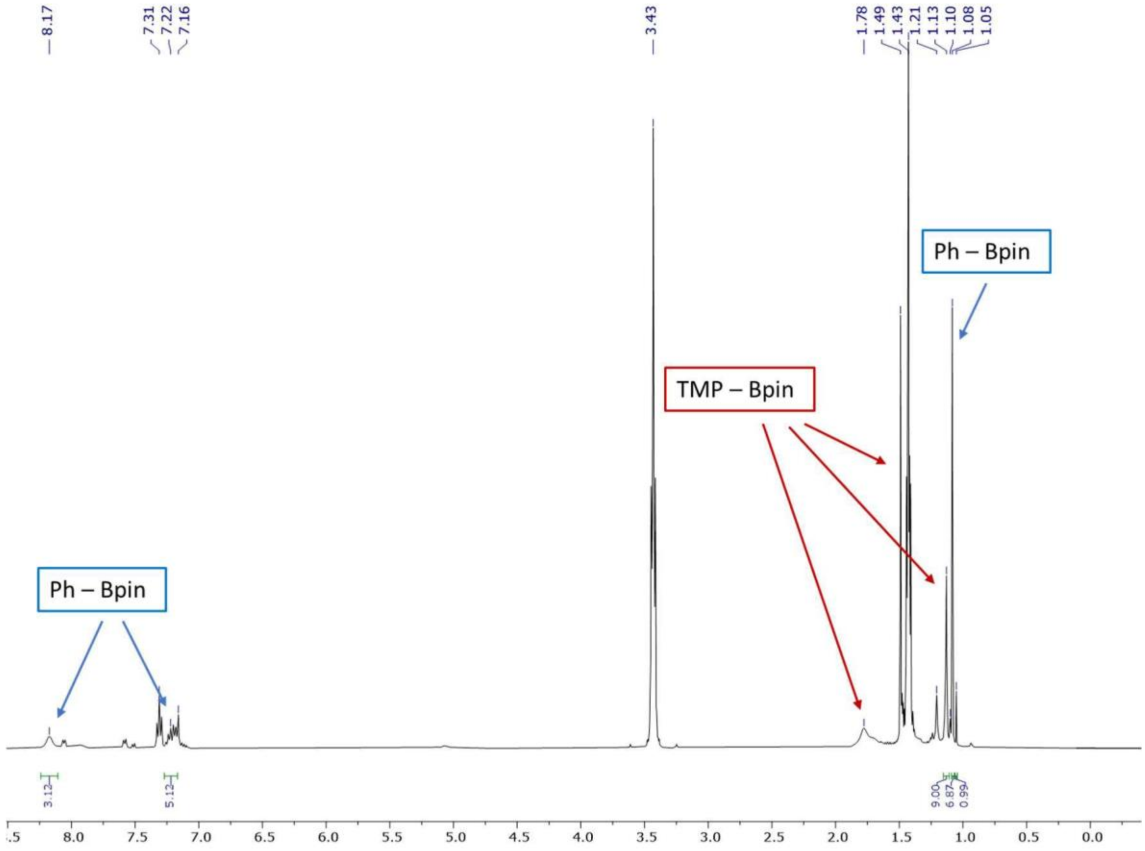

Figure S7. ^{1}H NMR spectrum of reaction mixture in C_6D_6 (+ 0.1 mL THF) at room temperature.

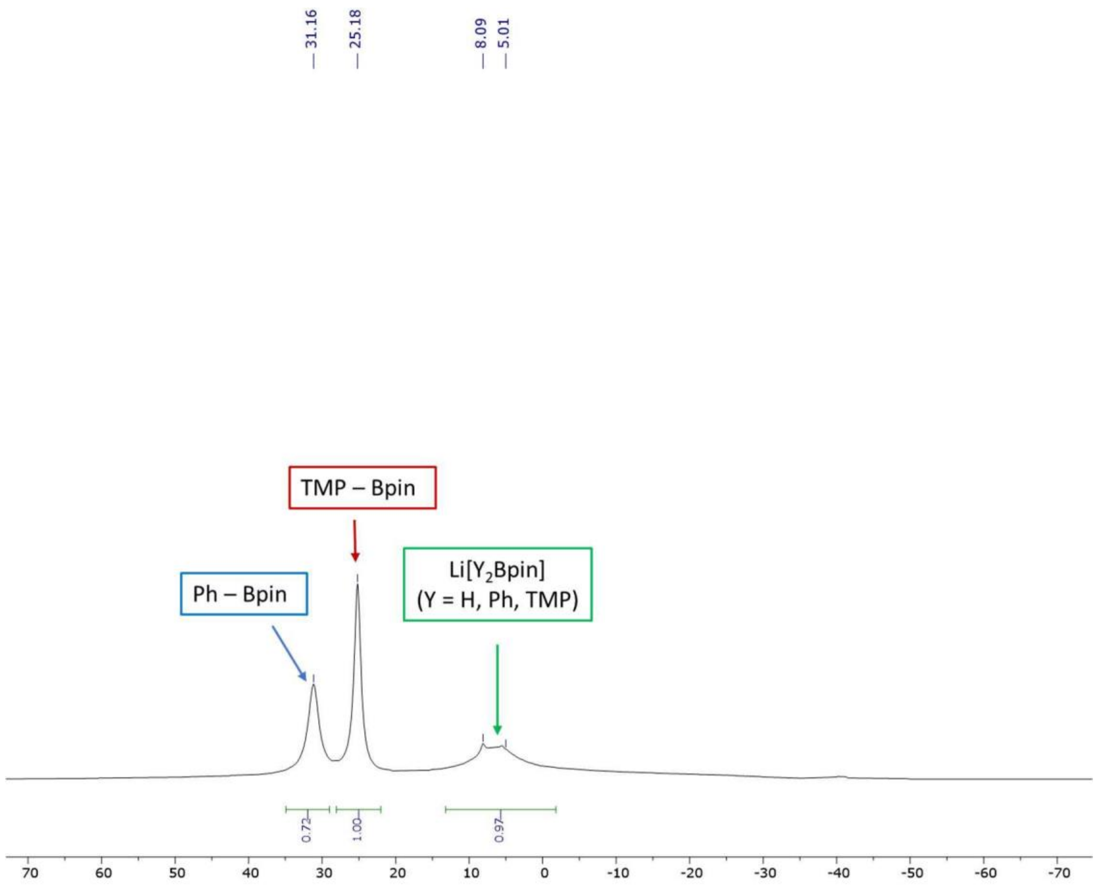

Figure S8. ^{11}B NMR spectrum of reaction mixture in C_6D_6 (+ 0.1 mL THF) at room temperature.

2.5 Reaction of LiTMP, $ZnPh_2$ and DME with 2 equivalents of HBPin

Lithium 2,2,6,6-tetramethylpiperidide (29 mg, 0.2 mmol, 1 eq) and diphenylzinc (44 mg, 0.2 mmol, 1 eq) were loaded into a J Young's NMR tube and suspended in C_6D_6 (0.5 mL). After 15 min 1,2-dimethoxyethane (DME) was added as a co-solvent (21 µL, 0.2 mmol, 1 eq) affording a homogenous solution. Pinacolborane (58 µL, 0.4 mmol, 2 eq) was added and after 1 h stirring at room temperature, the reaction mixture was analysed by ^{1}H and ^{11}B NMR spectroscopies which showed formation of both pinB-TMP and pinB-Ph species in approximately equal amounts (Figures S9-S10).

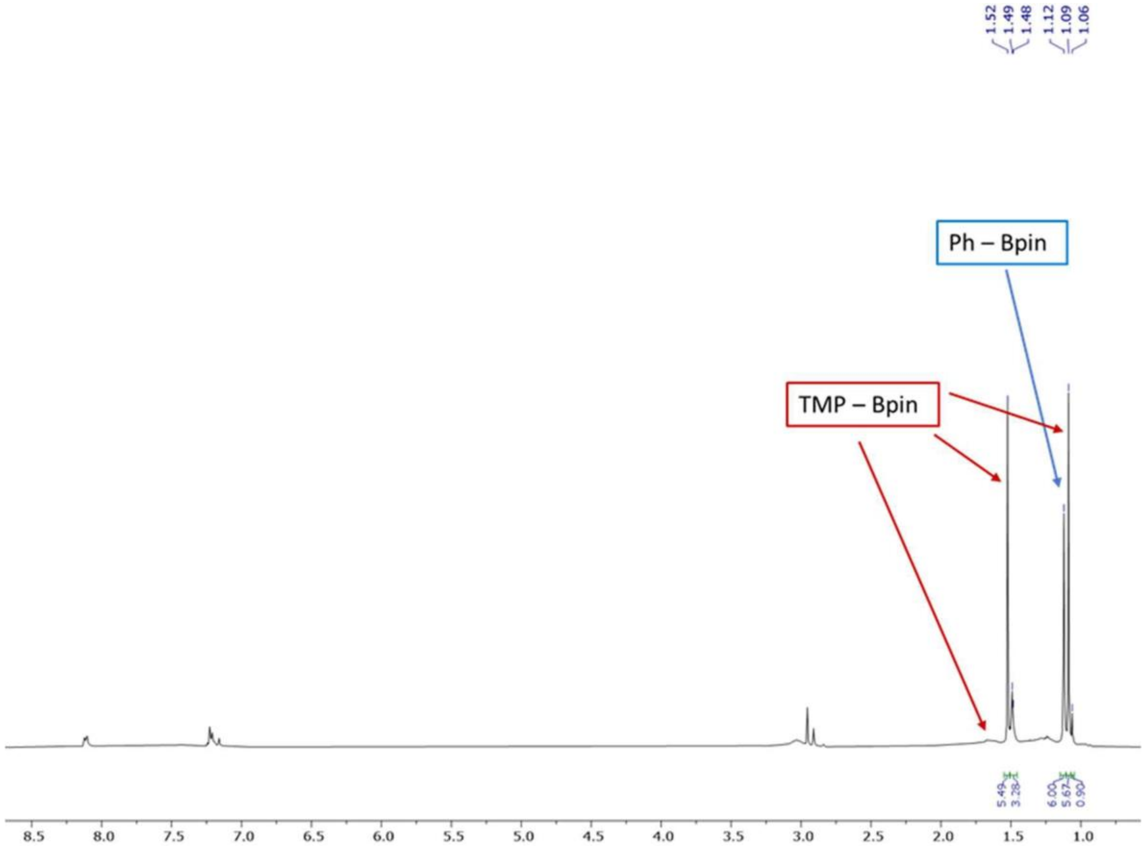

Figure S9. ^{1}H NMR spectrum of reaction mixture in C_6D_6 (+ 21 µL DME) at room temperature.

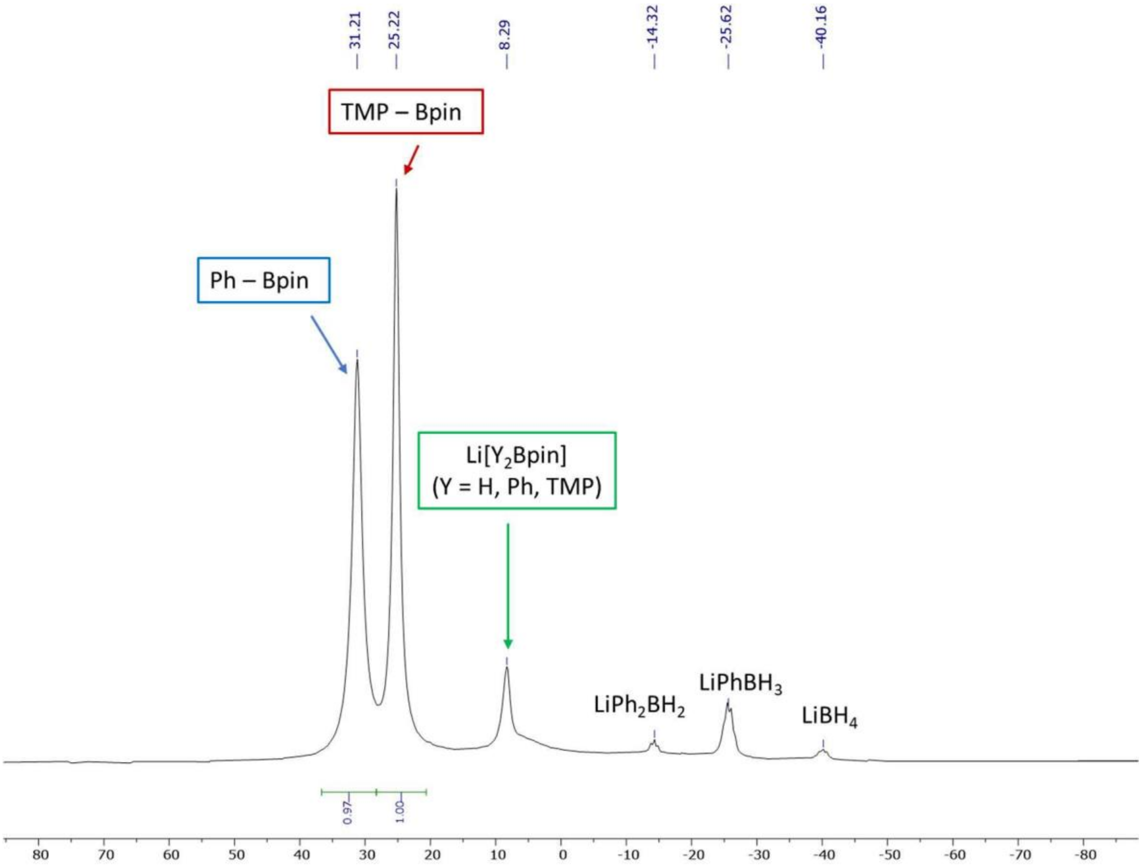

Figure S10. ^{11}B NMR spectrum of reaction mixture in C_6D_6 (+ 21 µL DME) at room temperature.

2.6 Reaction of KTMP and $ZnPh_2$ with HBPin

Potassium 2,2,6,6-tetramethylpiperidide (18 mg, 0.1 mmol, 1 eq) and diphenylzinc (22 mg, 0.1 mmol, 1 eq) were loaded into a J Young's NMR tube and suspended in C_6D_6 (0.5 mL). THF was added dropwise until a homogeneous solution was obtained. Pinacolborane (14.5 µL, 0.1 mmol, 1 eq) was added and after 1 h stirring at room temperature, the reaction mixture was analysed by ^{1}H and ^{11}B NMR spectroscopies which showed formation of pinB-Ph, pinB-TMP and K[Y_2BPin] type species (Figures S11-S12).

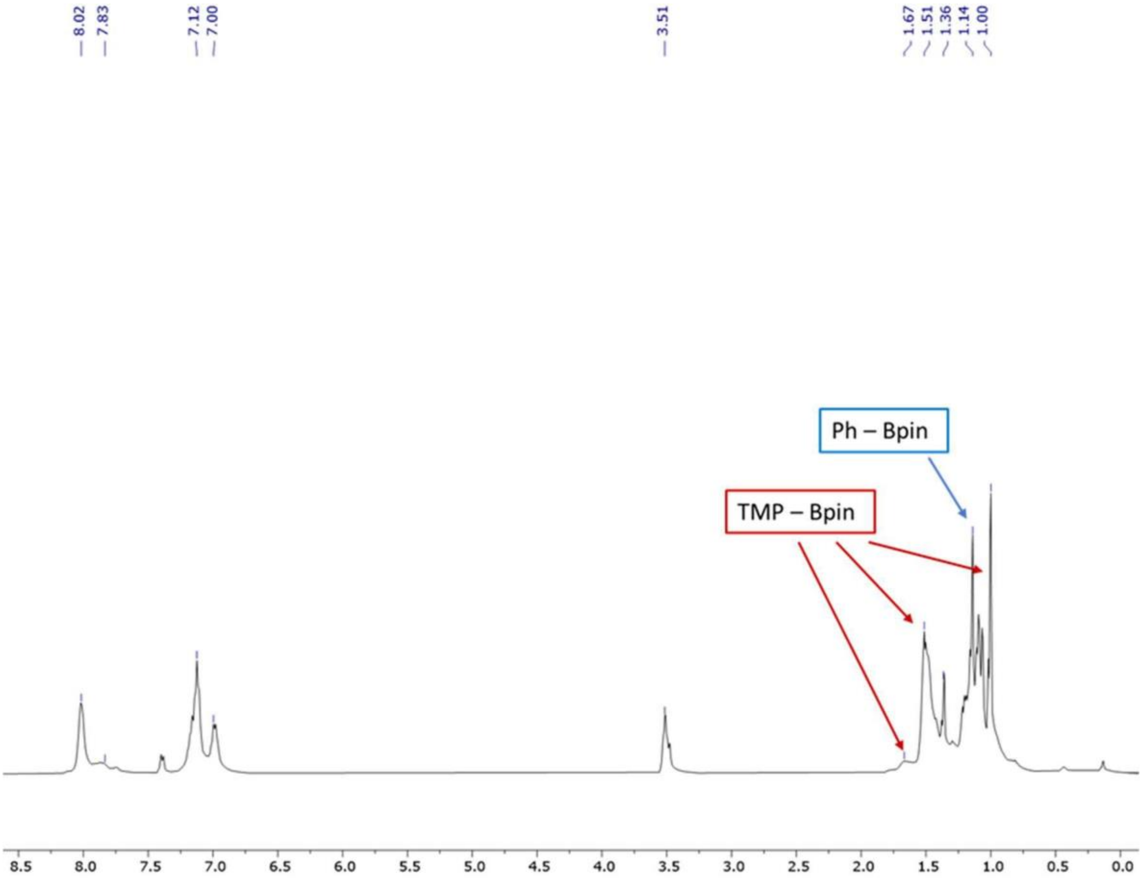

Figure S11. ^{1}H NMR spectrum of reaction mixture in C_6D_6 (with THF) at room temperature.

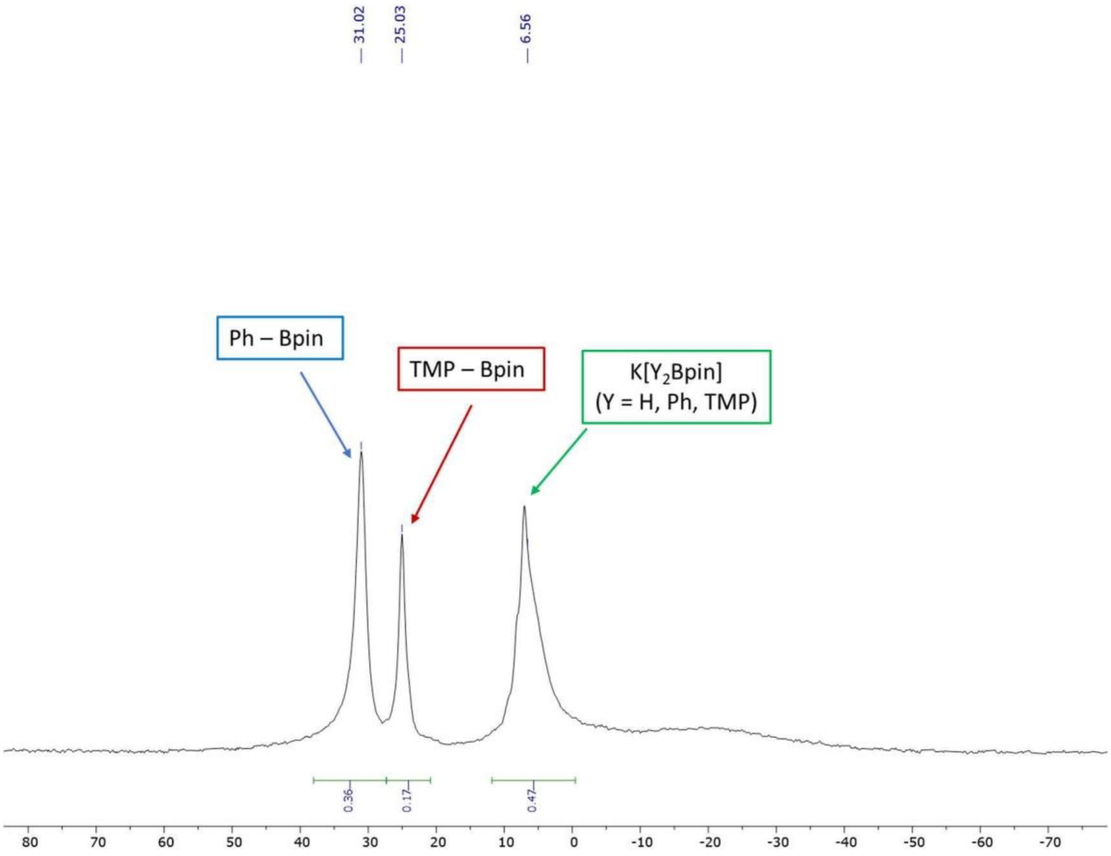

Figure S12. ^{11}B NMR spectrum of reaction mixture in C_6D_6 (with THF) at room temperature.

2.7 Reaction of Lidpa and $ZnPh_2$ with HBPin

Lithium dipyridylamide (18 mg, 0.1 mmol, 1 eq) was loaded into a J Young's NMR tube, followed by Ph_2Zn (22 mg, 0.1 mmol, 1 eq) and THF (0.5 mL). To this light yellow solution, pinacolborane (14 µL, 0.1 mmol, 1 eq) was added and after 2 h stirring at room temperature, the reaction mixture was analysed by ^{1}H and ^{11}B NMR spectroscopy which showed predominant formation of PhBPin and several other side-products in low quantities (Figure S13-S14).

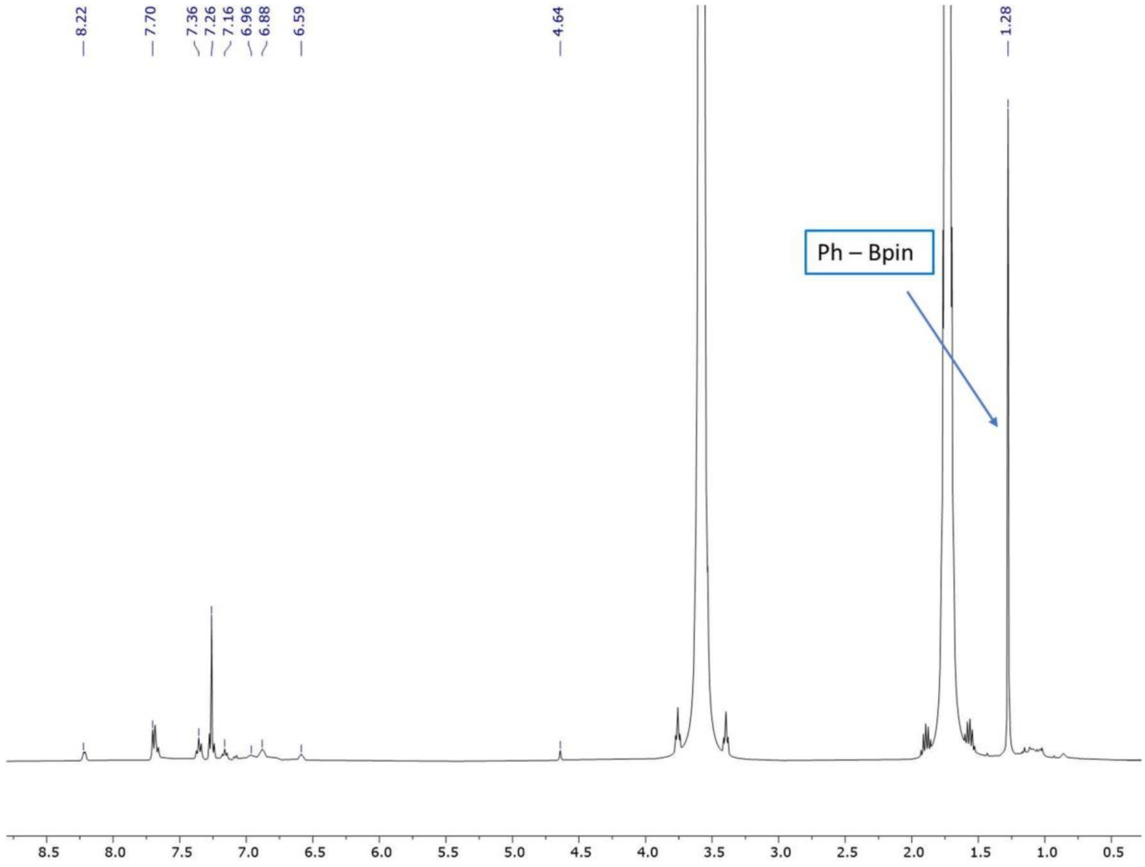

Figure S13. ^{1}H NMR spectrum of reaction mixture in THF at room temperature.

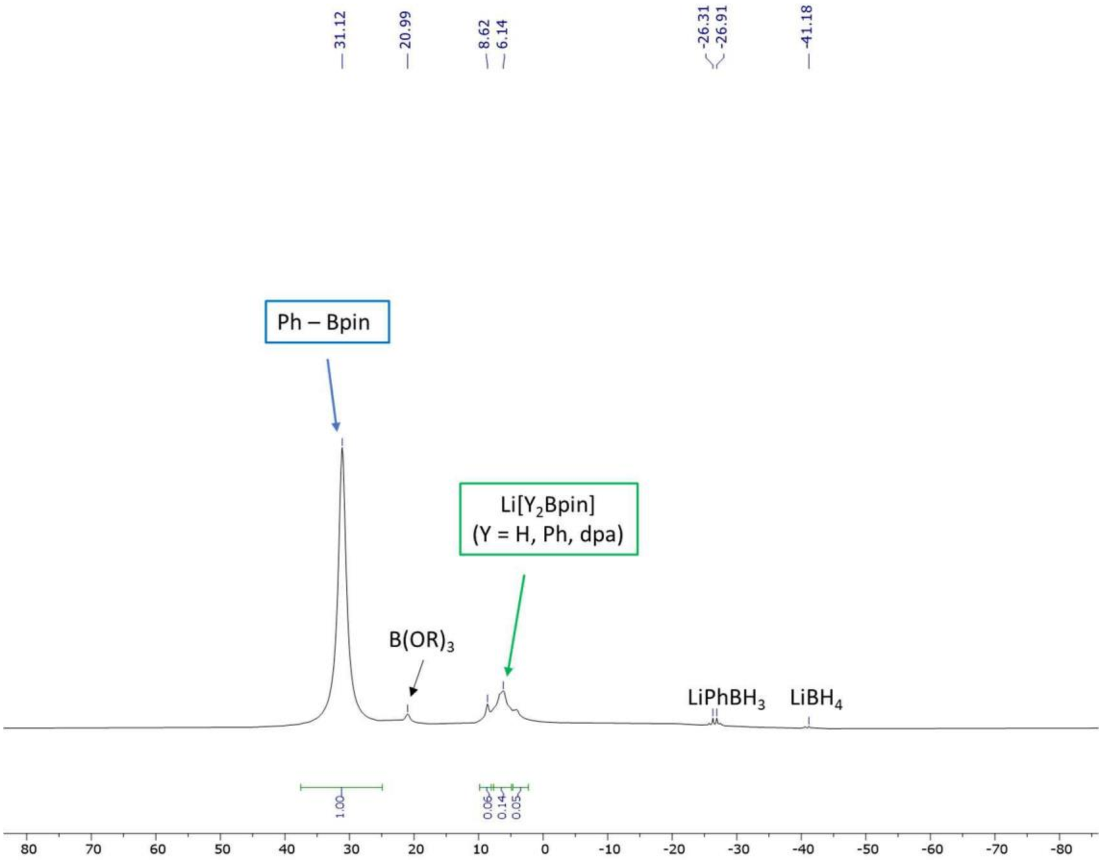

Figure S14. ^{11}B NMR spectrum of reaction mixture in THF at room temperature.

3. Synthesis and characterisation of complexes 1 – 4

3.1 [(THF)$_3$Li(μ-dpa)ZnPh$_2$] (1)

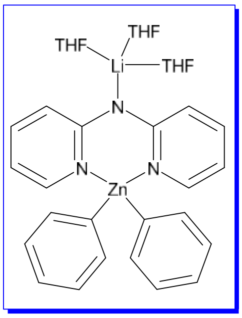

Method A: 2,2'-dipyridylamine (0.171 g, 1 mmol, 1 eq.) was loaded into a Schlenk tube and suspended in hexane (5 mL). To this, *n*BuLi (1.6 M in hexanes, 0.62 mL, 1 mmol, 1 eq) was added and the suspension was stirred for 2 h at room temperature. In another Schlenk tube, diphenylzinc (0.22 g, 1 mmol, 1 eq) was dissolved in THF (3 mL) and this solution was added to the suspension of lithium amide. The obtained yellow suspension was stirred overnight. The product was isolated by filtration (0.43 g, 70.5%).

Method B: Diphenylzinc (0.11 g, 0.5 mmol, 1 eq) and phenyllithium (0.047 g, 0.5 mmol, 1 eq) were loaded into a Schlenk tube and suspended in hexane (5 mL) affording a white suspension which was stirred for 15 min. The obtained suspension was solubilised by adding THF (5 mL) and stirred for 10 min. The obtained solution was added to another Schlenk tube containing 2,2'-dipyridylamine (0.085 g, 0.5 mmol, 1 eq) and the solution was stirred for 2 h. After 40 min stirring the product started crashing out, but the suspension was stirred for 2 h, then placed in freezer to crystallise more product which was isolated by filtration (0.255 g, 83.3 %)

Crystals suitable for X-ray diffraction were grown from a solution in THF layered with pentane after several days at -30 °C. Anal. Calc. for $C_{34}H_{42}LiN_3O_3Zn$: C, 66.61; H, 6.91; N, 6.85; found: C, 66.33; H, 6.80; N, 7.03.

^{1}H NMR (500 MHz, C_6D_6, 298 K): δ 8.08 (d, *J*=7.0 Hz, 4 H, *o*-C*H*$_{Ph}$), 7.75 (d, *J*=4.7 Hz, 2H, C*H*$_{dpa}$), 7.37 (t, *J*=7.2, Hz, 4 H, *m*-C*H*$_{Ph}$), 7.28 (t, *J*=7.3 Hz, 2 H, *p*-C*H*$_{Ph}$), 7.20 (dd, *J*=8.6, 2H, C*H*$_{dpa}$), 6.98 (ddd, *J*=8.9, 6.9, 2.0 Hz, 2 H, C*H*$_{dpa}$), 6.23 (m, 2 H, C*H*$_{dpa}$), 3.34 (m, 12H, α-THF), 1.28 (m, 12H, β-THF). ^{13}C{^{1}H} NMR (126 MHz, C_6D_6, 298 K): δ 163.7(*C*-N$_{dpa}$), 159.0 (Zn-*C*$_{Ph}$), 146.6 (*C*H$_{dpa}$), 140.9 (*o*-*C*H$_{Ph}$), 138.1 (*C*H$_{dpa}$), 127.1 (*m*-*C*H$_{Ph}$), 126.2 (*p*-*C*H$_{Ph}$), 115.2 (*C*H$_{dpa}$), 112.5 (*C*H$_{dpa}$) ppm. ^{7}Li NMR (155.5 MHz, C_6D_6, 298 K): 1.14 (s).

^{1}H NMR (500 MHz, THF-*d*$_8$, 298 K): δ 7.92 (dd, *J*=5.6, 2.1 Hz, 2H, C*H*$_{dpa}$), 7.53 (d, *J*=6.7 Hz, 4 H, *o*-C*H*$_{Ph}$), 7.25 (ddd, *J*=8.8, 6.8, 2.1 Hz, 2 H, C*H*$_{dpa}$), 6.90 (t, *J*=7.2, Hz, 4 H, *m*-C*H*$_{dpa}$), 6.83 (t, *J*=7.4 Hz, 2 H, *p*-C*H*$_{dpa}$), 6.70 (m, 2H, C*H*$_{dpa}$), 6.38 (m, 2 H, C*H*$_{dpa}$), 3.61 (m, 12H, α-THF), 1.77 (m, 12H, β-THF). ^{13}C{^{1}H} NMR (126 MHz, THF-*d*$_8$, 298 K): δ 165.6 (Zn-*C*$_{Ph}$), 162.8 (*C*-N$_{dpa}$), 147.2 (*C*H$_{dpa}$), 140.4 (*o*-*C*H$_{Ph}$), 136.7 (*C*H$_{dpa}$), , 126.0(*m*-*C*H$_{Ph}$), 124.0 (*p*-*C*H$_{Ph}$) 119.4 (*C*H$_{dpa}$), 111.7(*C*H$_{dpa}$) 68.0 (α-THF); 26.2 (β-THF) ppm. ^{7}Li NMR (155.5 MHz, THF-*d*$_8$, 298 K): 0.34 (s).

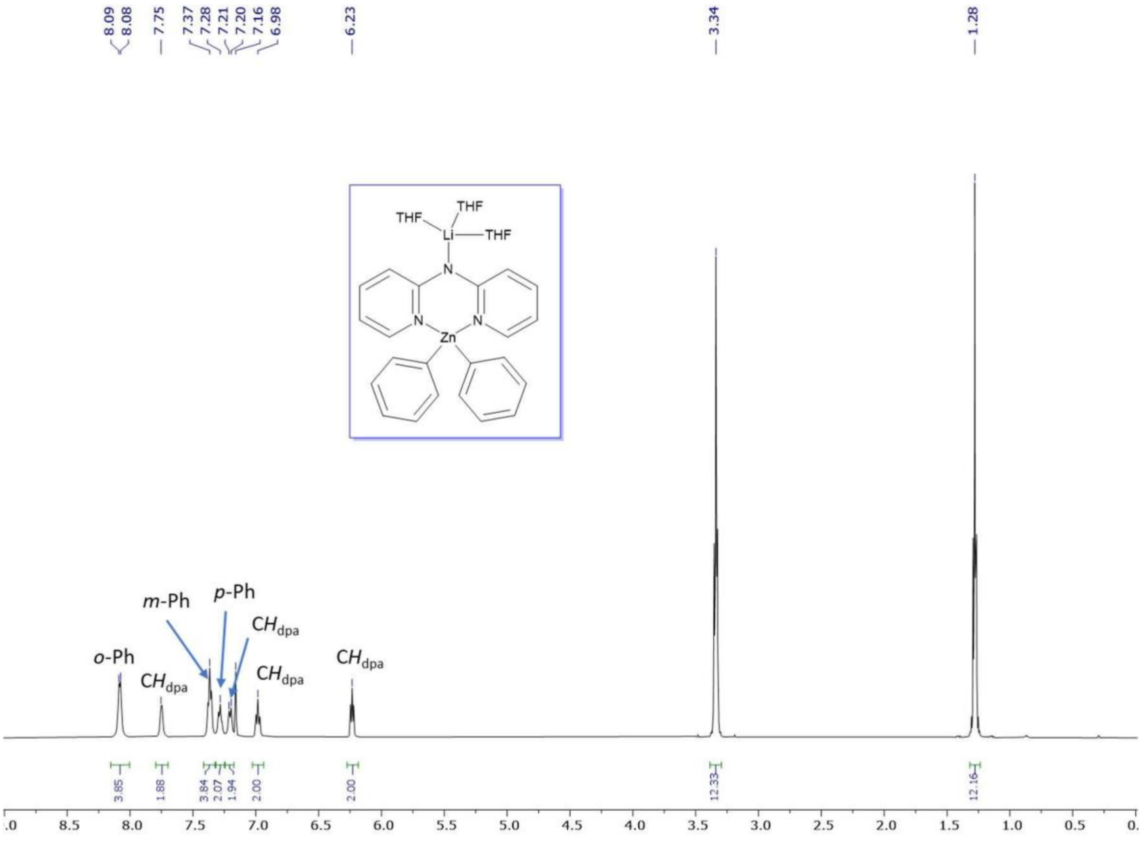

Figure S15. ^{1}H NMR spectrum of **1** in C_6D_6 at room temperature.

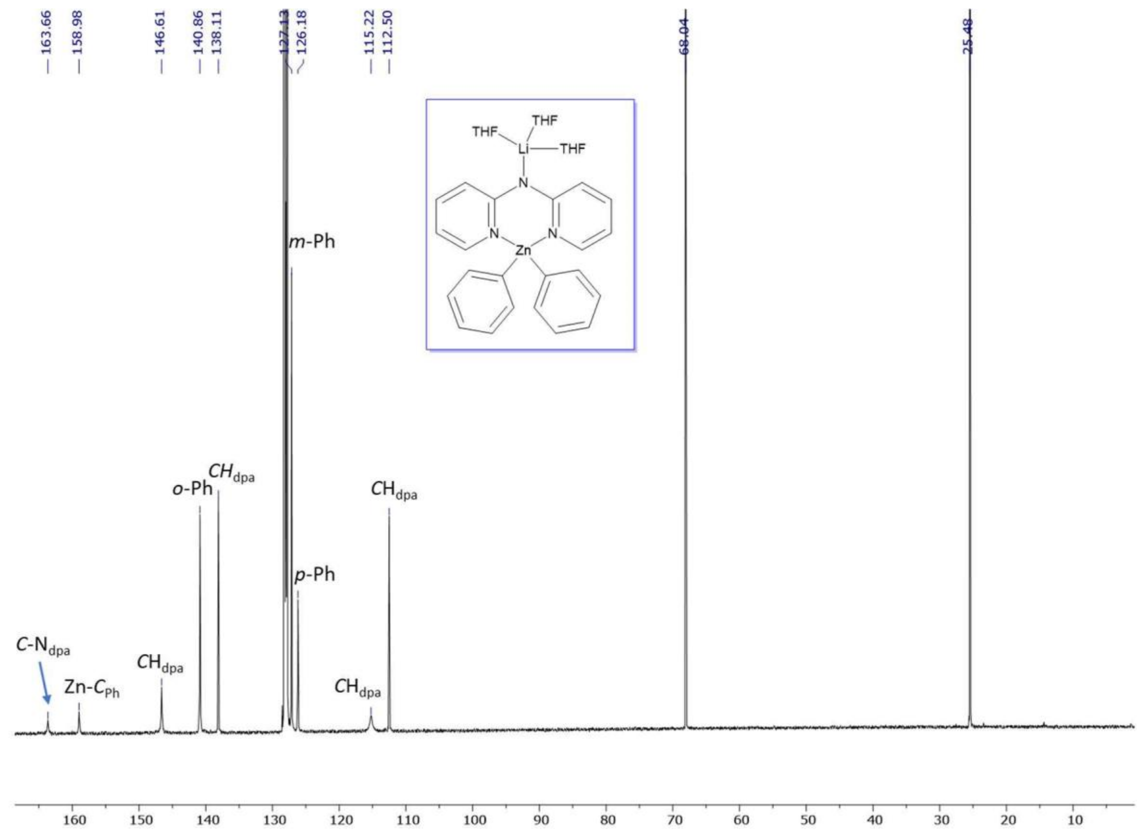

Figure S16. $^{13}C\{^{1}H\}$ NMR spectrum of **1** in C_6D_6 at room temperature.

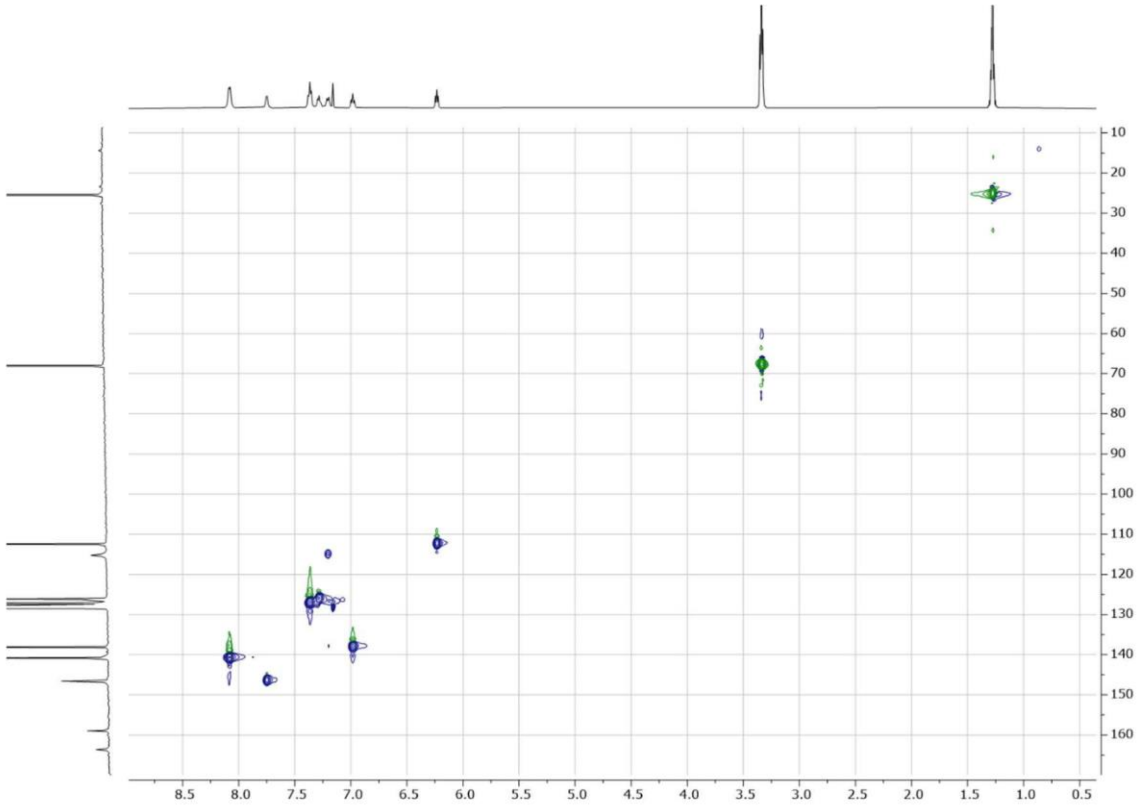

Figure S17. ^{1}H – $^{13}C\{^{1}H\}$ HSQC NMR spectrum of **1** in C_6D_6 at room temperature.

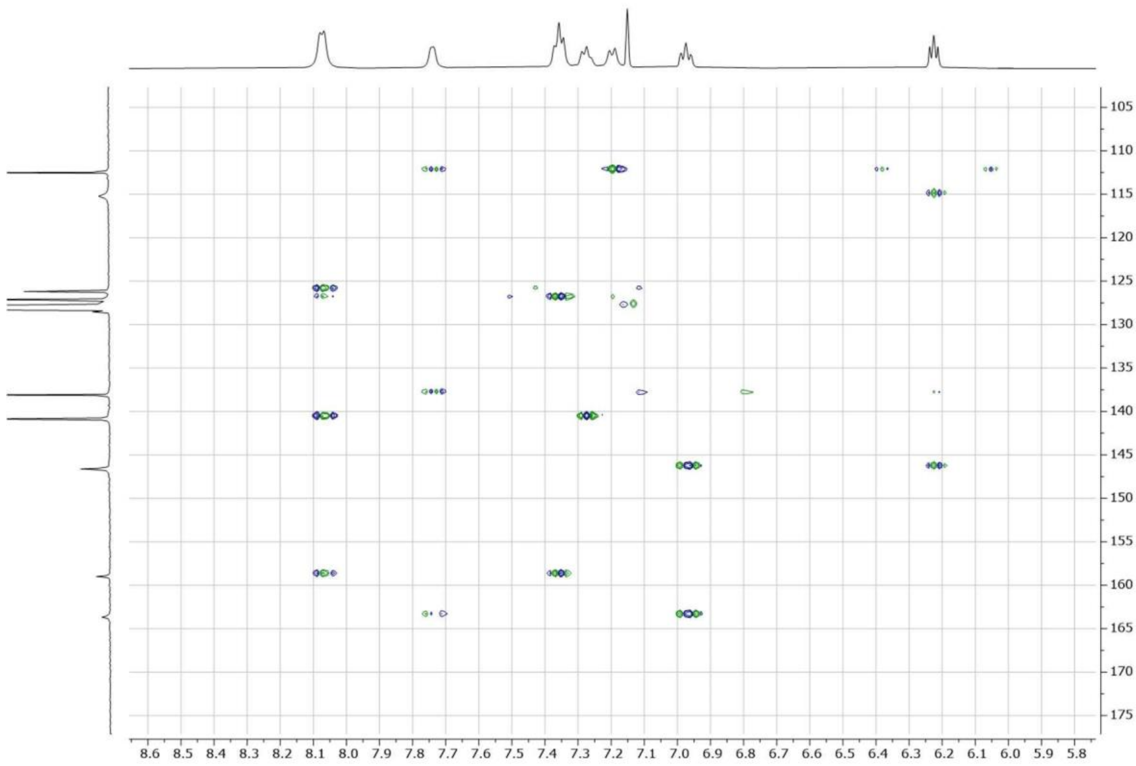

Figure S18. ^{1}H – $^{13}C\{^{1}H\}$ HMBC NMR spectrum of **1** in C_6D_6 at room temperature.

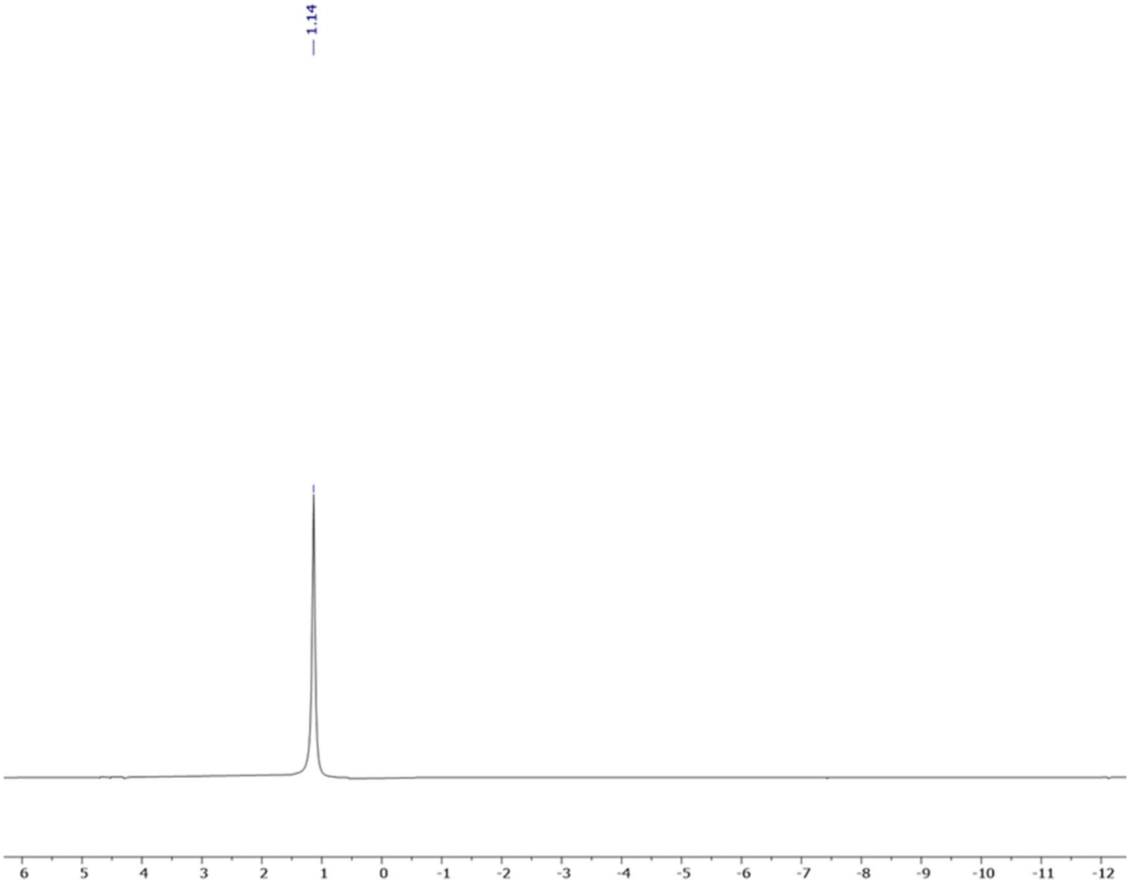

Figure S19. ^{7}Li NMR spectrum of **1** in C_6D_6 at room temperature.

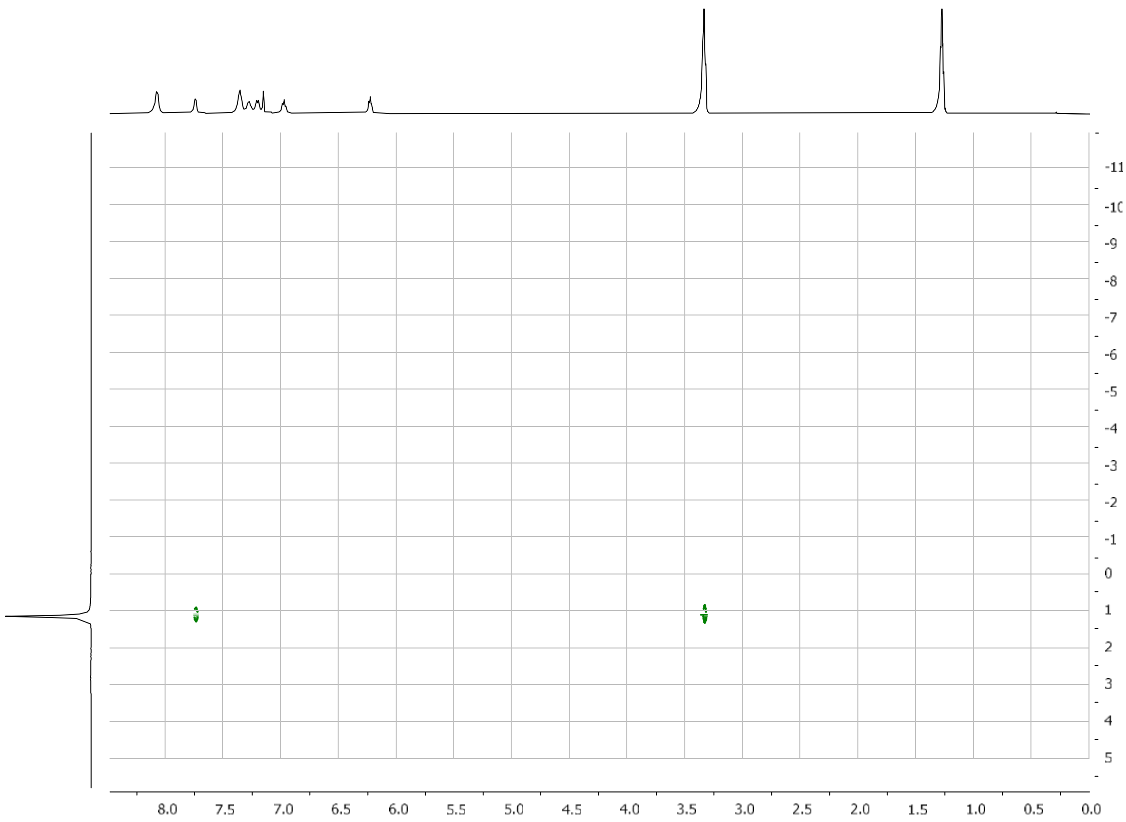

Figure 20. ^{1}H – ^{7}Li HOESY NMR spectrum of **1** in C_6D_6 at room temperature.

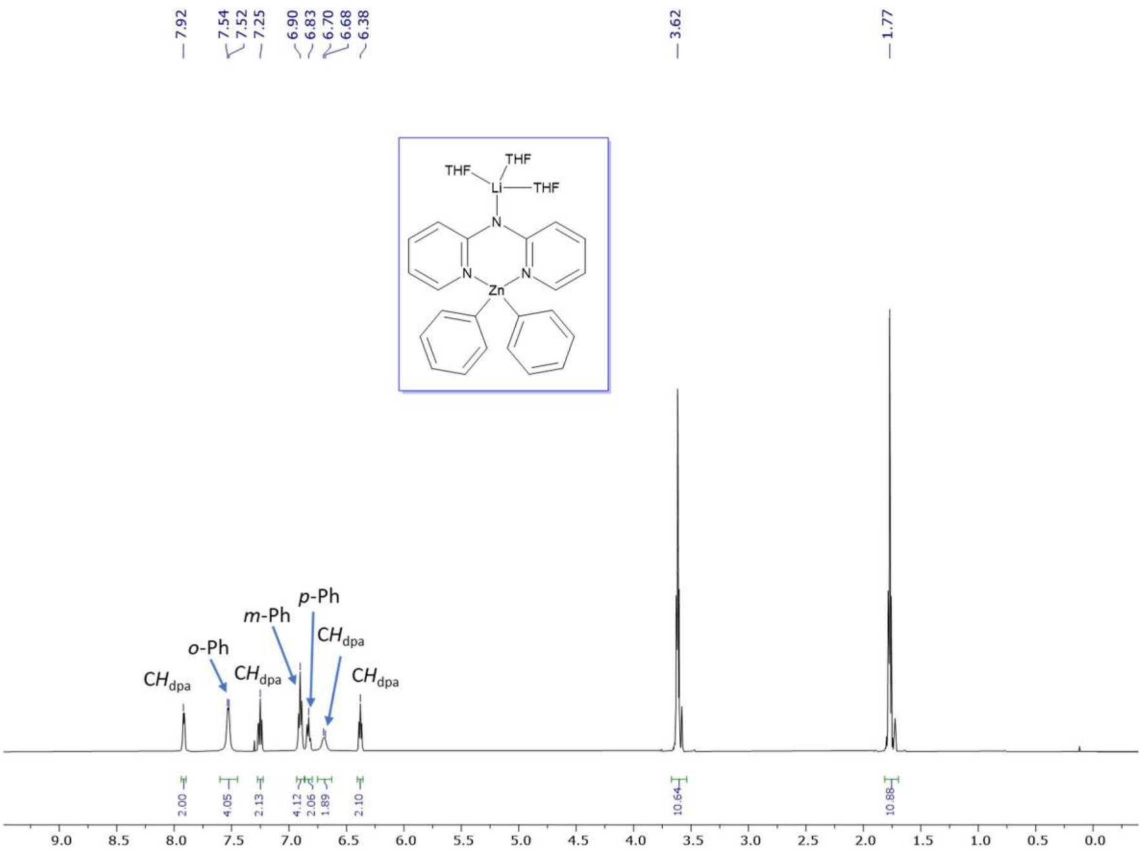

Figure S21. ^{1}H NMR spectrum of **1** in d_8-THF at room temperature.

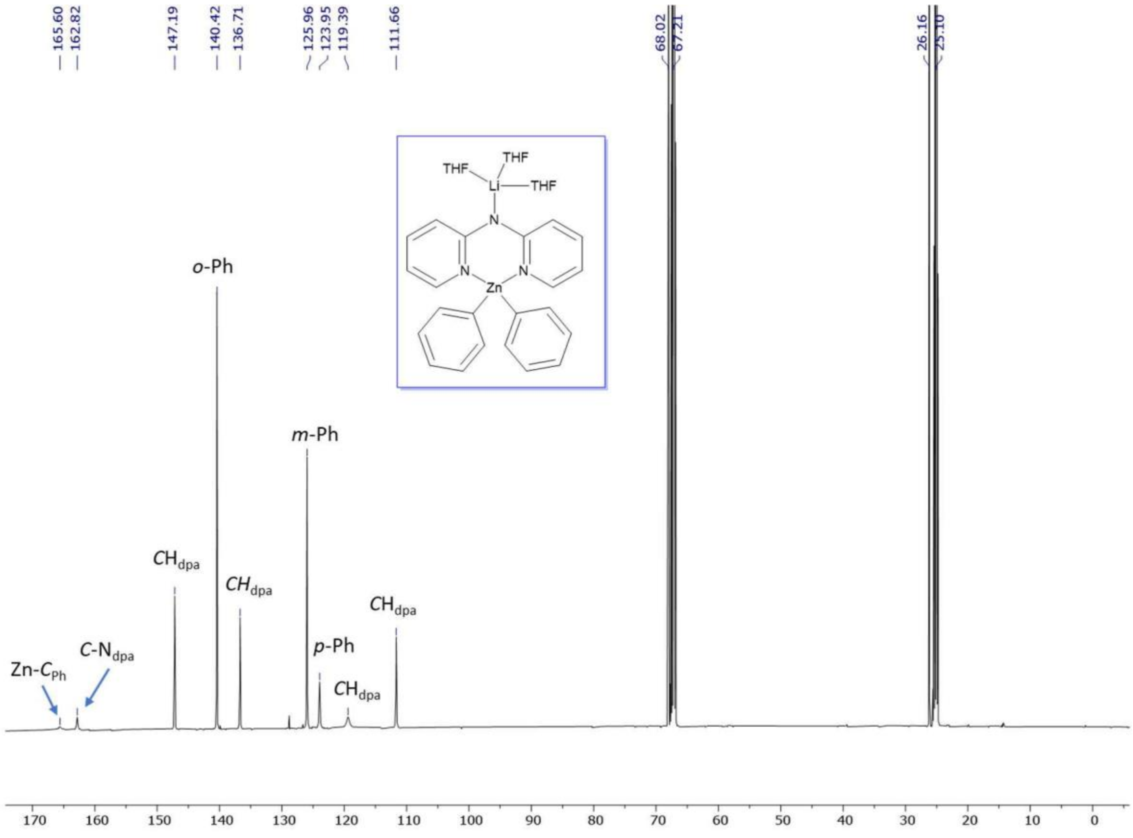

Figure S22. $^{13}C\{^1H\}$ NMR spectrum of **1** in d_8-THF at room temperature.

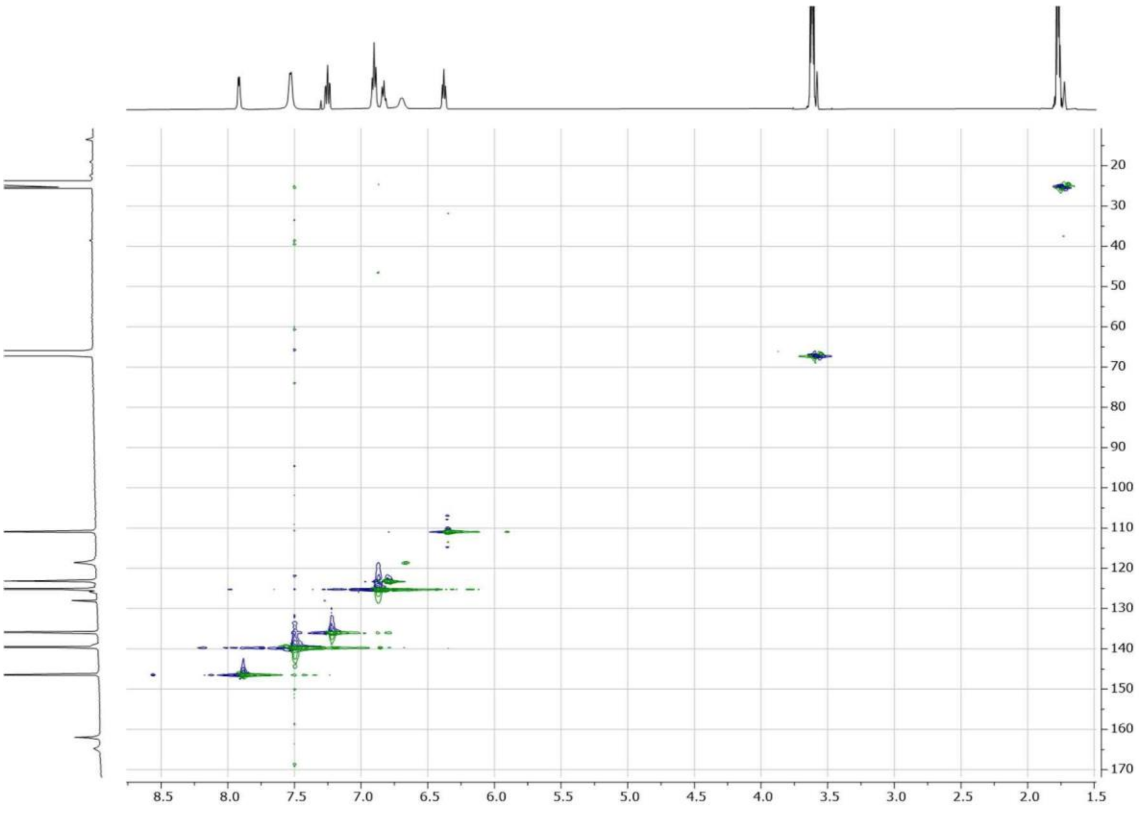

Figure S23. ^1H – $^{13}C\{^1H\}$ HSQC NMR spectrum of **1** in d_8-THF at room temperature.

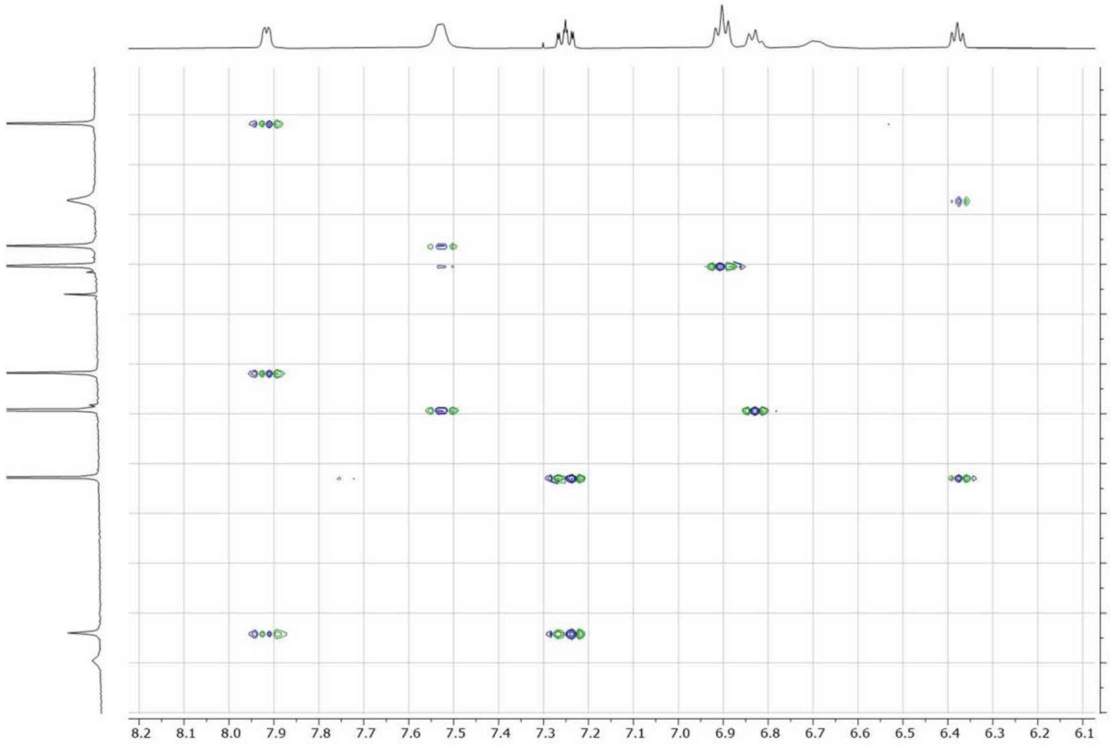

Figure S24. ^1H – $^{13}C\{^1H\}$ HMBC NMR spectrum of **1** in d_8-THF at room temperature.

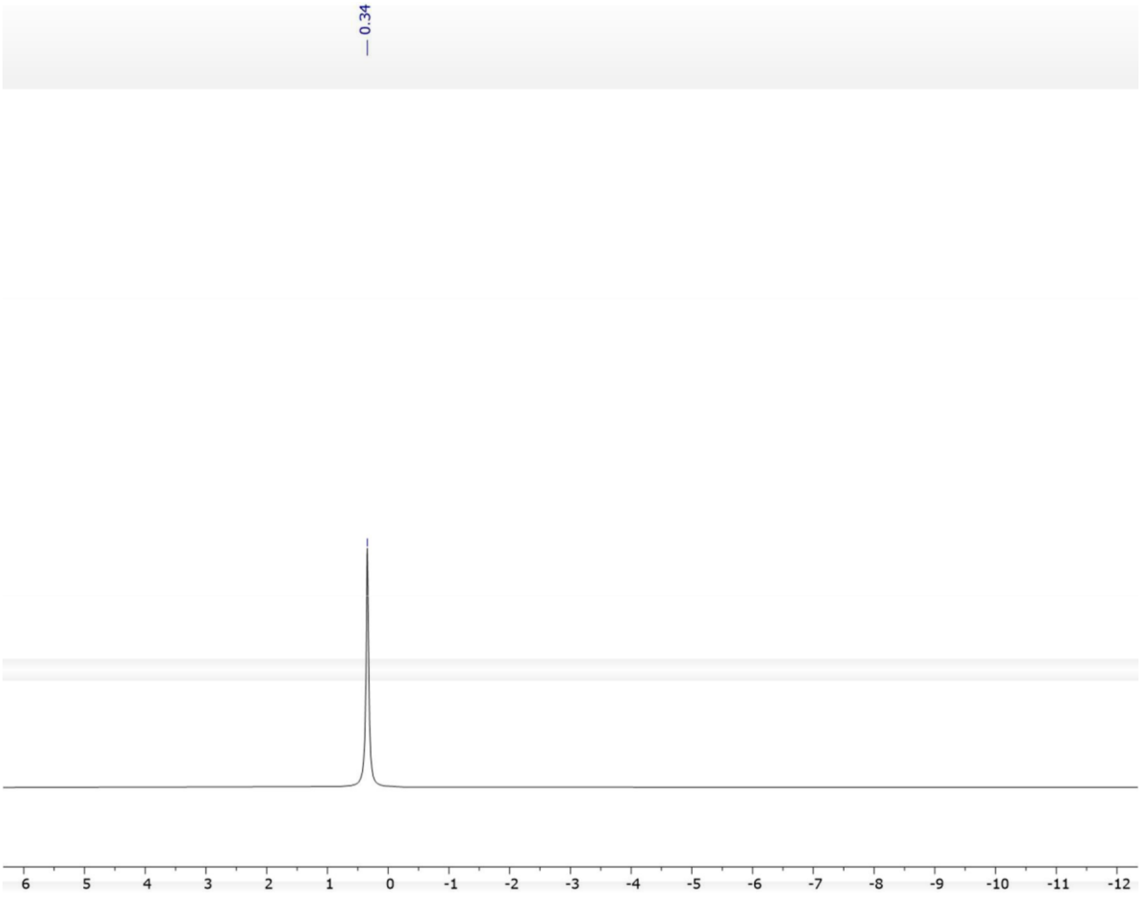

Figure S25. ^7Li NMR spectrum of **1** in d_8-THF at room temperature.

3.2 [(dpaZnPh)$_3$] (2)

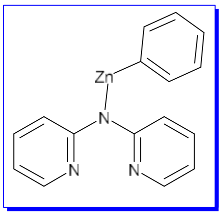

Diphenylzinc (0.11 g, 0.5 mmol, 1 eq) was loaded into a Schlenk tube and dissolved in toluene (5 mL). The solution was added to another Schlenk tube containing neat 2,2'-dipyridylamine (85 mg, 0.5 mmol, 1 eq) and stirred for one hour affording an off-white suspension. The product was isolated by filtration (240 mg, 77%). Crystals suitable for analysis by X-ray diffraction were grown from a concentrated C_6D_6 solution of **2** standing at ambient temperature over a period of several days. Anal. Calc. for $C_{16}H_{13}N_3Zn$: C, 61.46; H, 4.19; N, 13.44; found: C, 61.79; H, 4.25; N, 12.73.

^{1}H NMR (500 MHz, C_6D_6, 298 K): δ 8.13 (m, 2 H, *o*-CH_{Ph}), 7.68 (ddd, *J*=5.6, 2.1, 0.7 Hz, 2H, CH_{dpa}), 7.60 (t, *J*=7.5, Hz, 2 H, *m*-CH_{Ph}), 7.48 (m, 1 H, *p*-CH_{Ph}), 7.11 (dd, *J*=8.6, 0.9 Hz, 2H, CH_{dpa}), 6.58 (ddd, *J*=8.8, 7.0, 2.0 Hz, 2 H, CH_{dpa}), 5.90 (ddd, *J*=6.8, 5.6, 1.1 Hz, 2 H, CH_{dpa}). ^{13}C{^{1}H} NMR (126 MHz, C_6D_6, 298 K): δ 160.6 (C-N$_{dpa}$), 155.5 (Zn-C_{Ph}), 146.4 (CH$_{dpa}$), 140.7 (*o*-CH$_{Ph}$), 137.7 (CH$_{dpa}$), 127.9 (*m*-CH$_{Ph}$), 126.8 (*p*-CH$_{Ph}$), 121.5 (CH$_{dpa}$), 113.5 (CH$_{dpa}$) ppm.

MS: While the molecular ion was not observed several fragments containing multiple Zn/dpa units were, including: (MS-EI$^+$) found: 405.08711 [$C_{20}H_{17}N_6Zn$ = dpa-Zn-dpa(H)], simulated 405.08006
642.08569 [$C_{30}H_{24}N_9Zn_2$ = dpa$_3$Zn$_2$], simulated 642.06905

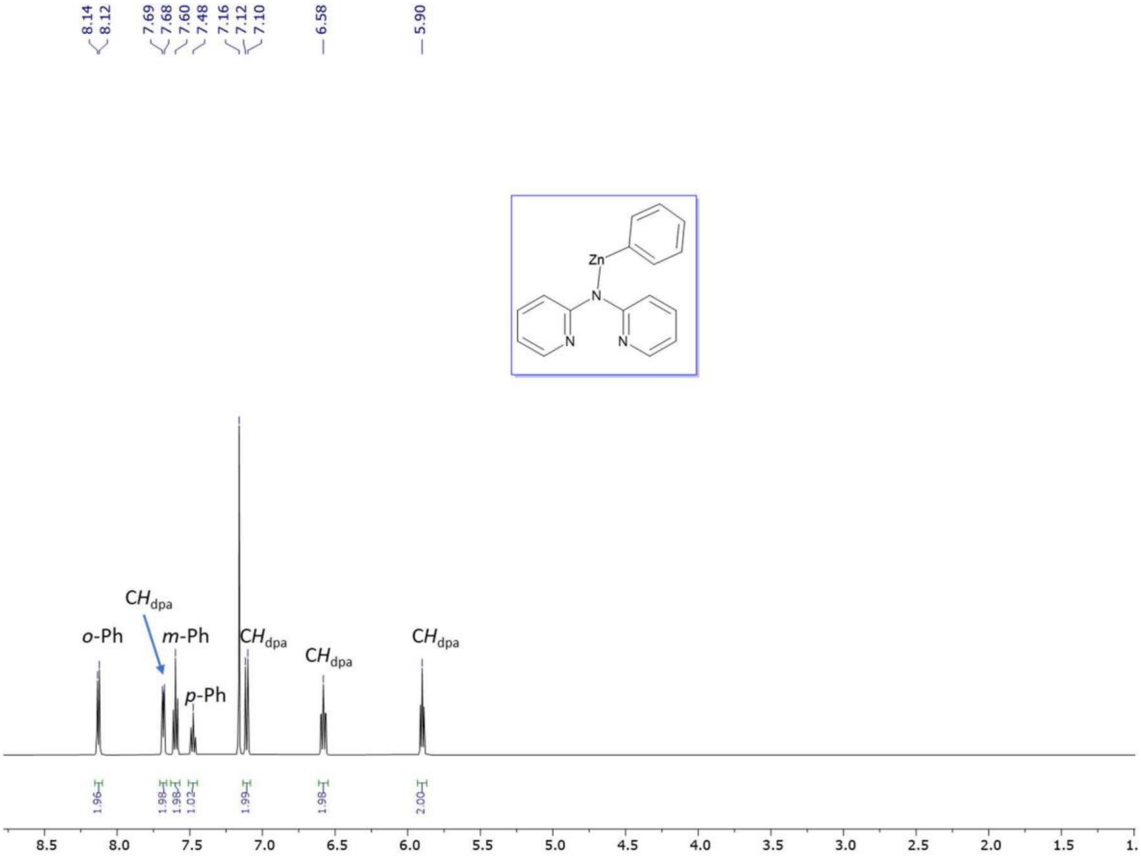

Figure S26. ^{1}H NMR spectrum of **2** in C_6D_6 at room temperature.

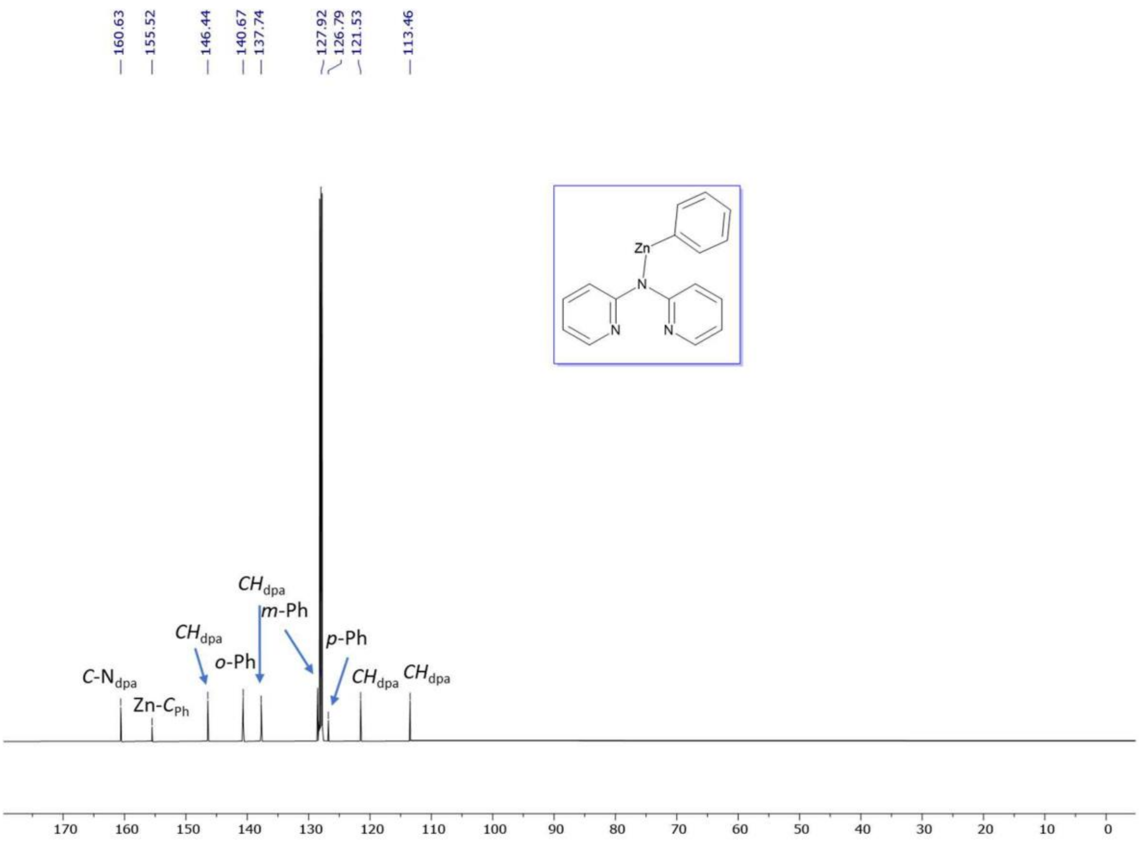

Figure S27. $^{13}C\{^{1}H\}$ NMR spectrum of **2** in C_6D_6 at room temperature.

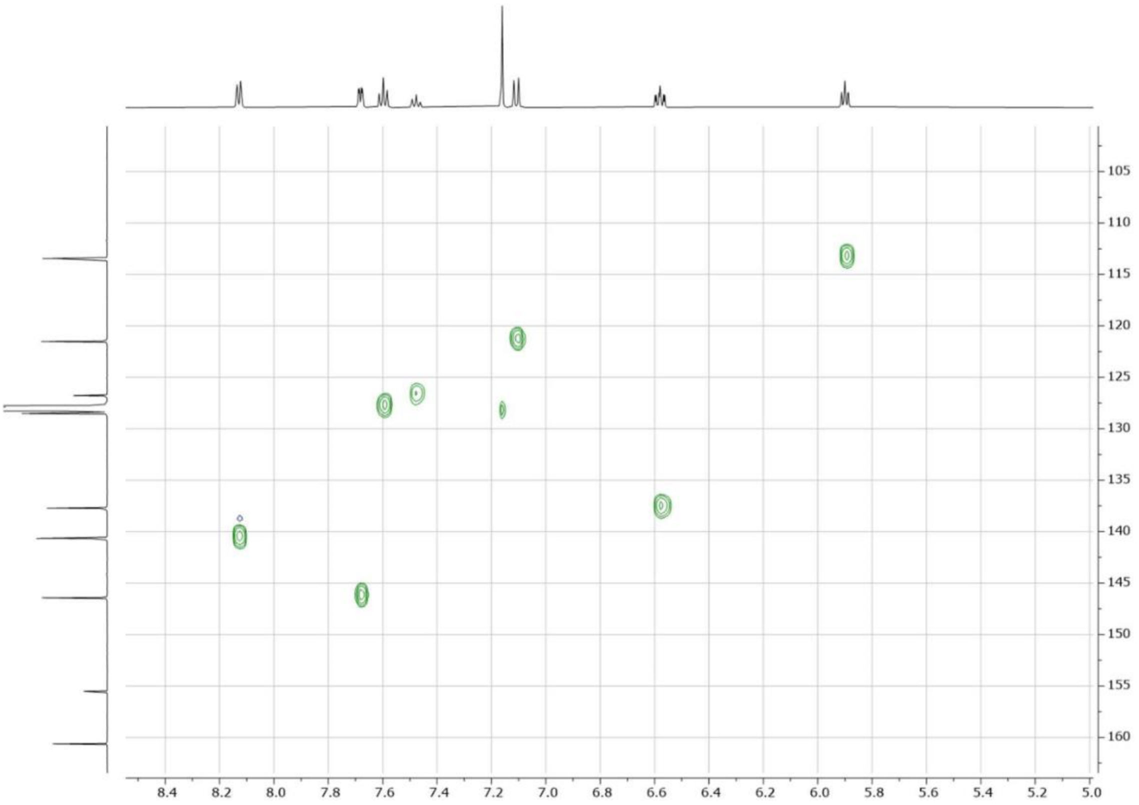

Figure S28. ^{1}H – $^{13}C\{^{1}H\}$ HSQC NMR spectrum of **2** in C_6D_6 at room temperature.

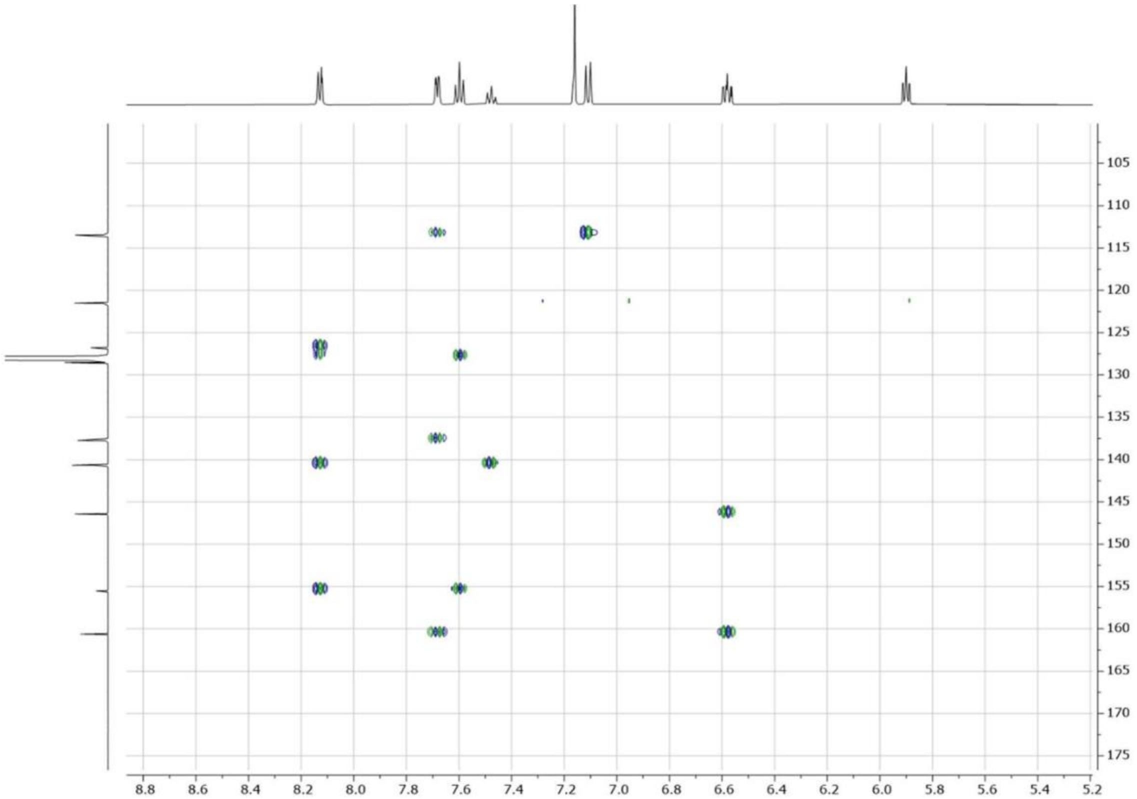

Figure S29. ^{1}H – ^{13}C{^{1}H} HMBC NMR spectrum of **2** in C_6D_6 at room temperature.

3.3 [(PMDETA)Li(μ-dpa)ZnPh$_2$] (3)

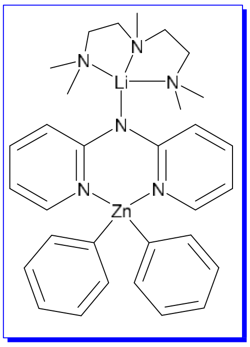

A solution of diphenylzinc (0.11 g, 0.5 mmol, 1 eq) in toluene (3 ml) was added to neat 2,2'-dipyridylamine (85 mg, 0.5 mmol, 1 eq) and stirred for 15 min at ambient temperature. In another Schlenk tube, PhLi (47 mg, 0.5 mmol, 1eq) was suspended in 5 mL toluene and PMDETA (0.11 mL, 0.5 mmol, 1 eq) was added affording a yellow solution which was then added to toluene solution of **2** and stirred for another 2 h at room temperature affording an off-white suspension. The product was isolated by filtration (184 mg, 65 %). Crystals suitable for analysis by X-ray diffraction were grown by slow mixing of toluene solutions of the separate organometallic reagents by diffusion without stirring at room temperature. Anal. Calc. for $C_{31}H_{41}LiN_6Zn$: C, 65.32; H, 7.25; N, 14.74; found: C, 64.40; H, 7.14; N, 14.72.

^{1}H NMR (500 MHz, THF-d_8, 298 K): δ 7.92 (m, 2 H, CH_{dpa}), 7.55 (d, *J*=6.7 Hz, 4 H, *o*-CH_{Ph}), 7.24 (ddd, *J*=8.8, 6.8, 2.1 Hz, 2 H, CH_{dpa}), 6.94 (br s, 4 H, *m*-CH_{Ph}), 6.86 (m, 2 H, *p*-CH_{Ph}), 6.78 (m, 2H, CH_{dpa}), 6.34 (m, 2 H, CH_{dpa}), 2.58 (t, *J*=6.7 Hz, 4 H, CH_2N), 2.42 (t, *J*=6.5 Hz, 4 H, CH_2N), 2.30 (s, 3 H, N*Me*), 2.16 (s, 12H, NMe_2) ppm. ^{13}C{^{1}H} NMR (126 MHz, THF-d_8, 298 K): δ 147.5(*C*H_{dpa}); 140.4 (o-*C*H_{Ph}); 136.8(*C*H_{dpa}); 126.2(m-*C*H_{Ph}); 125.8(p-*C*H_{Ph}); 124.2(*C*H_{dpa}); 111.2(*C*H_{dpa}); 58.2(N*C*H_2); 57.4(N*C*H_2); 46.4(NMe_2); 43.7(N*Me*) ppm. ^{7}Li NMR (155.5 MHz, d_8-THF, 298 K): 1.04 (s) ppm.

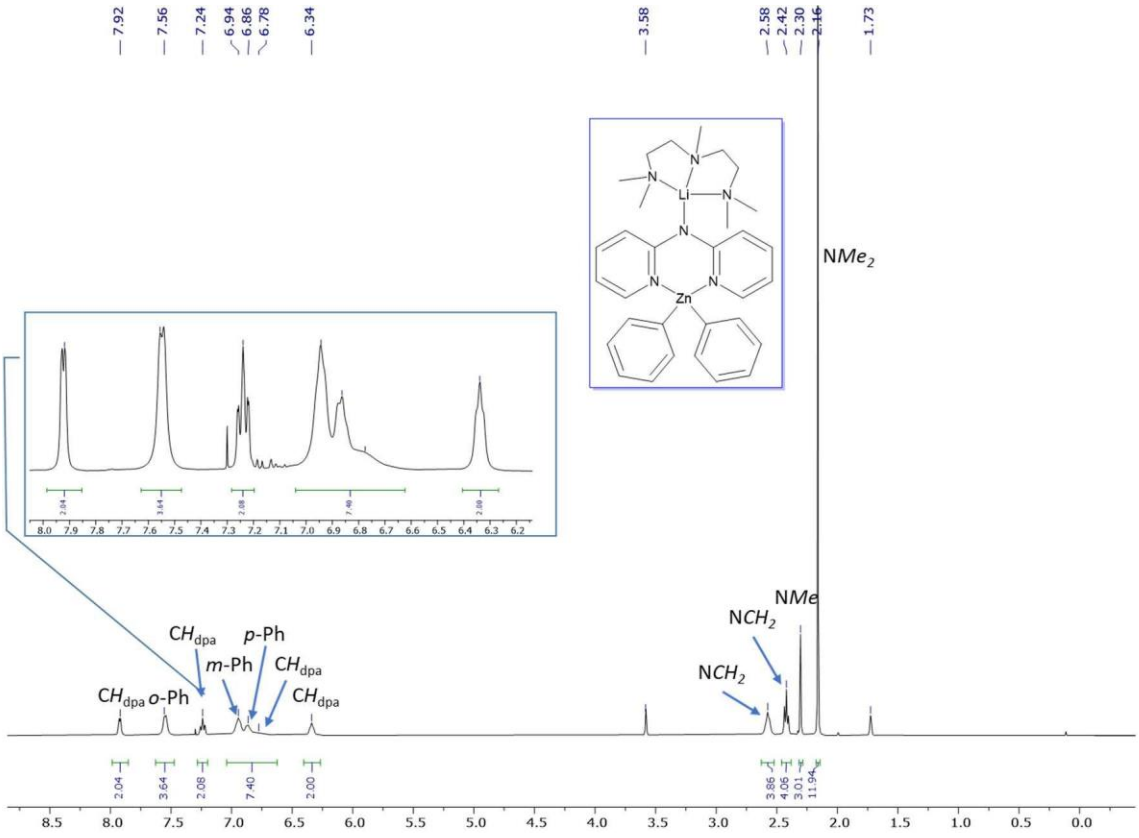

Figure S30. ^{1}H NMR spectrum of **3** in d_8-THF at room temperature.

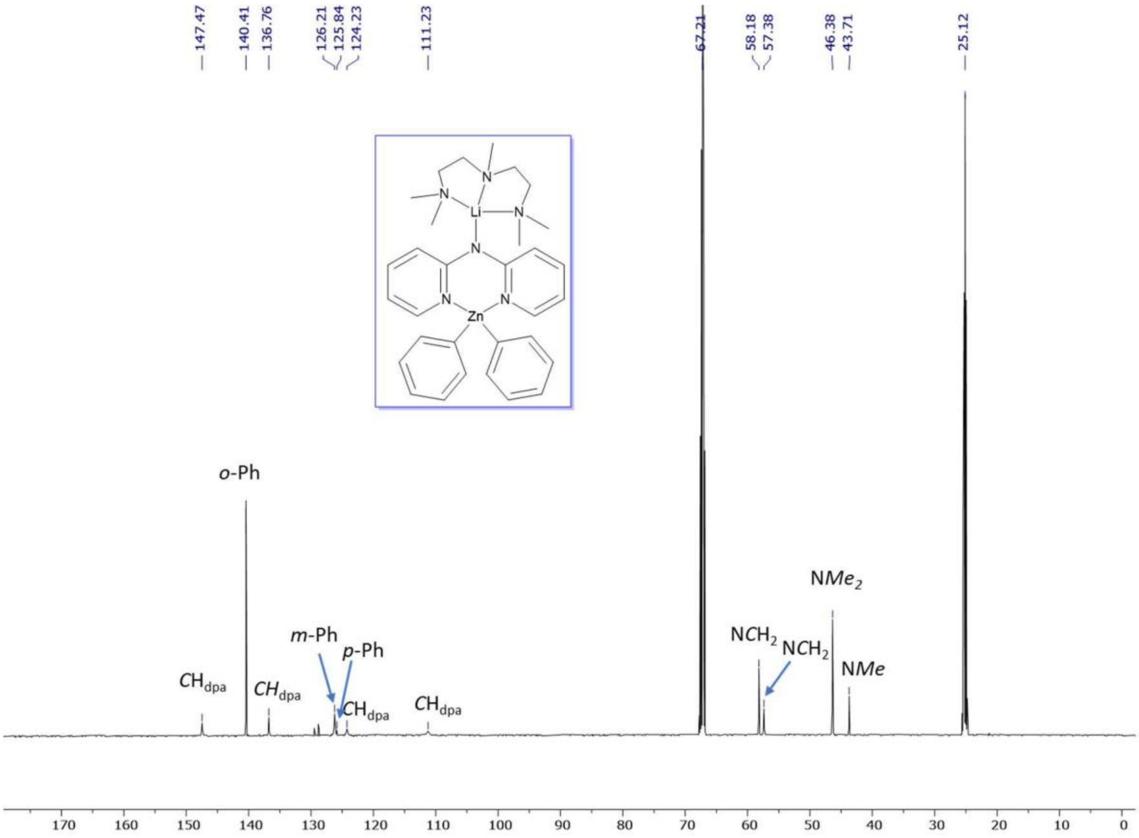

Figure S31. $^{13}C\{^{1}H\}$ NMR spectrum of **3** in d_8-THF at room temperature.

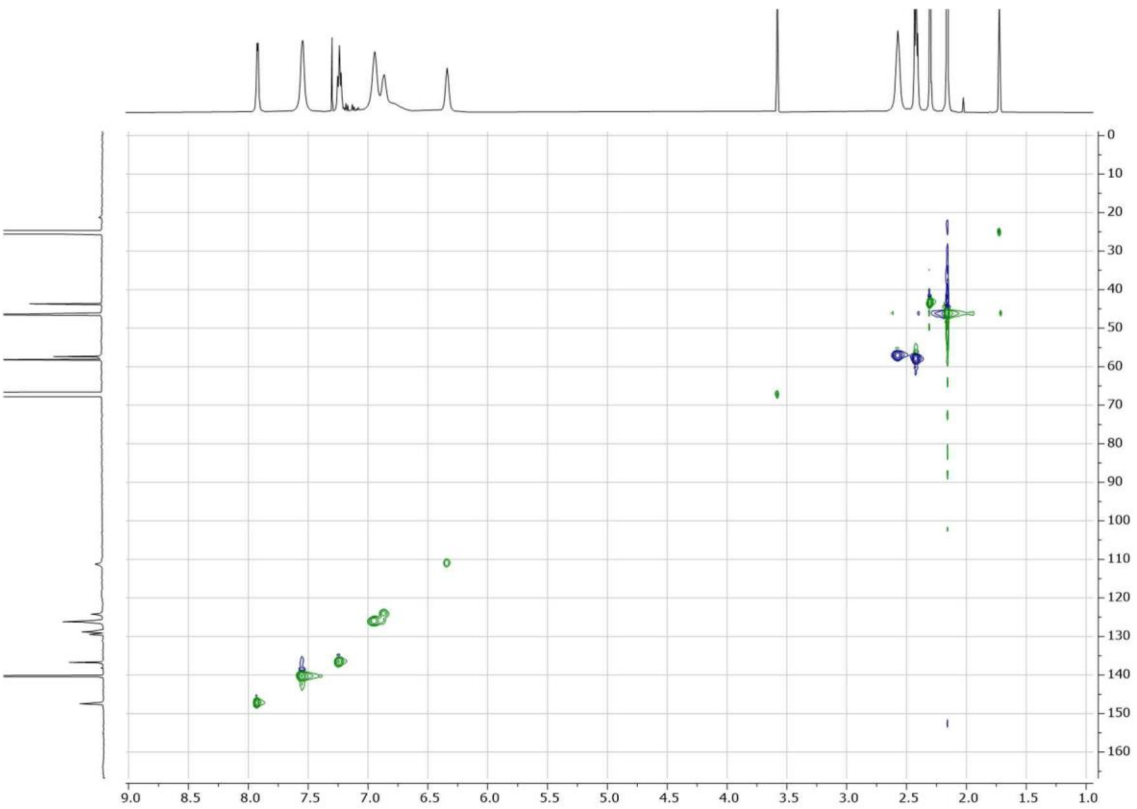

Figure S32. ^{1}H – $^{13}C\{^{1}H\}$ HSQC NMR spectrum of **3** in d_8-THF at room temperature.

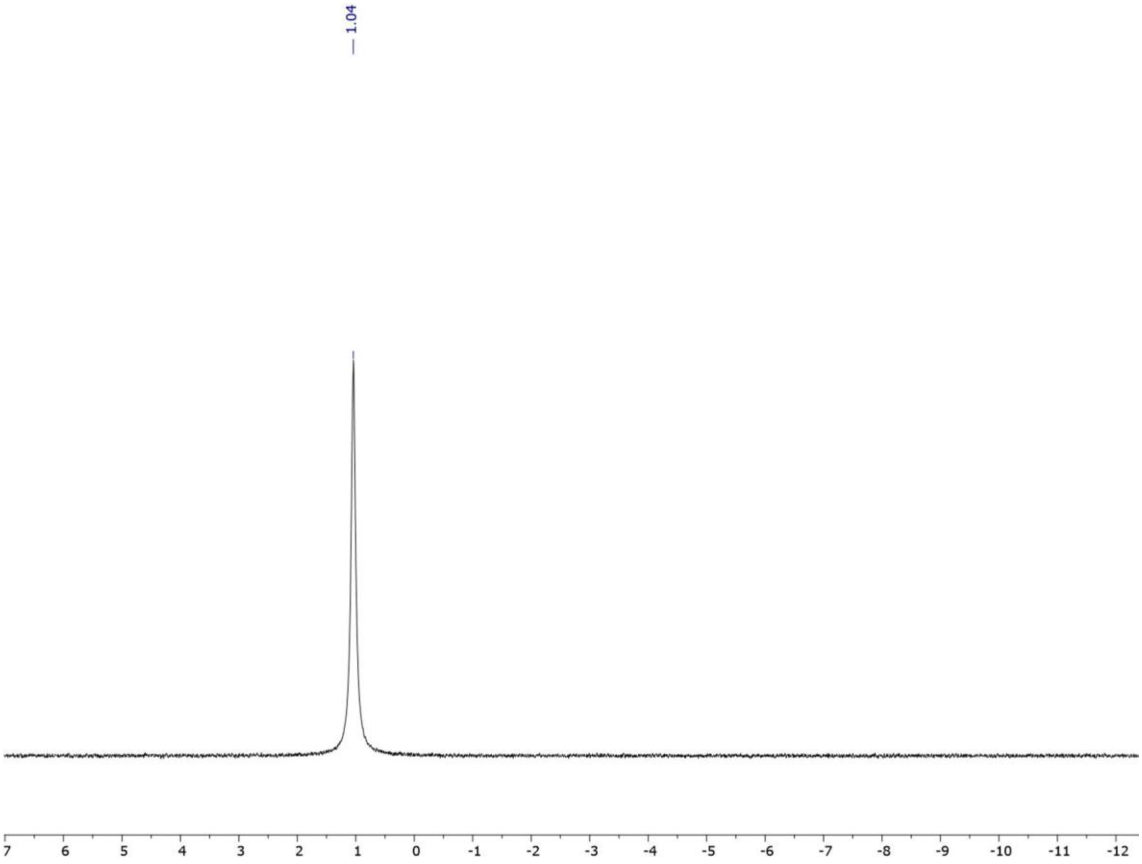

Figure S33. ^{7}Li NMR spectrum of **3** in d_8-THF at room temperature.

3.4 [(Me-dpa)·$ZnPh_2$] (4)

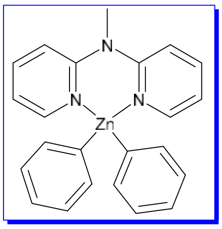

Diphenylzinc (0.11 g, 0.5 mmol, 1 eq) was loaded into a Schlenk tube and dissolved in toluene (3 mL). The solution was added to another Schlenk tube containing neat 2,2'-dipyridyl-*N*-methylamine (0.5 mmol, 94 mg, 1 eq) and stirred for 1 hour. Complex **4** was formed as a white precipitate, observable after 15 min of stirring. The solvent was exchanged *in vacuo* for 1,2-difluorobenzene (5 mL) and heated to reflux affording a clear solution which upon slow cooling to room temperature deposited crystals of **1** (121 mg, 60 %). Accurate elemental analysis was not obtained despite fully homogeneous samples being very clean by multinuclear NMR spectroscopy.

^{1}H NMR (500 MHz, C_6D_6, 298 K): δ 8.17 (ddd, *J*=5.2, 2.1, 0.8 Hz, 2 H,C*H*$_{dpa}$), 8.0 (m, 4 H, *o*-C*H*$_{Ph}$), 7.48 (t, 7.4 Hz, 4 H, *m*-C*H*$_{Ph}$), 7.38 (m, 2 H, *p*-C*H*$_{Ph}$), 6.85 (ddd, *J*=8.4, 7.3, 2.0 Hz, 2 H, C*H*$_{dpa}$), 6.19 (ddd, *J*=7.4, 5.2, 0.9 Hz, 2 H, C*H*$_{dpa}$), 6.14 (d, *J*=8.5 Hz, 2 H, C*H*$_{dpa}$), 2.57 ppm (s, 3 H, N*Me*). ^{13}C{^{1}H} NMR (126 MHz, C_6D_6, 298 K): δ 160.9 (Zn-*C*$_{Ph}$), 156.3 (*C*-N$_{dpa}$) 148.0 (*C*H$_{dpa}$), 140.3 (*o*-*C*H$_{Ph}$), 138.9 (*C*H$_{dpa}$), 127.1 (*m*-*C*H$_{Ph}$), 125.5 (*p*-*C*H$_{Ph}$) 118.4 (*C*H$_{dpa}$), 113.2 (*C*H$_{dpa}$), 38.2(N*Me*) ppm.

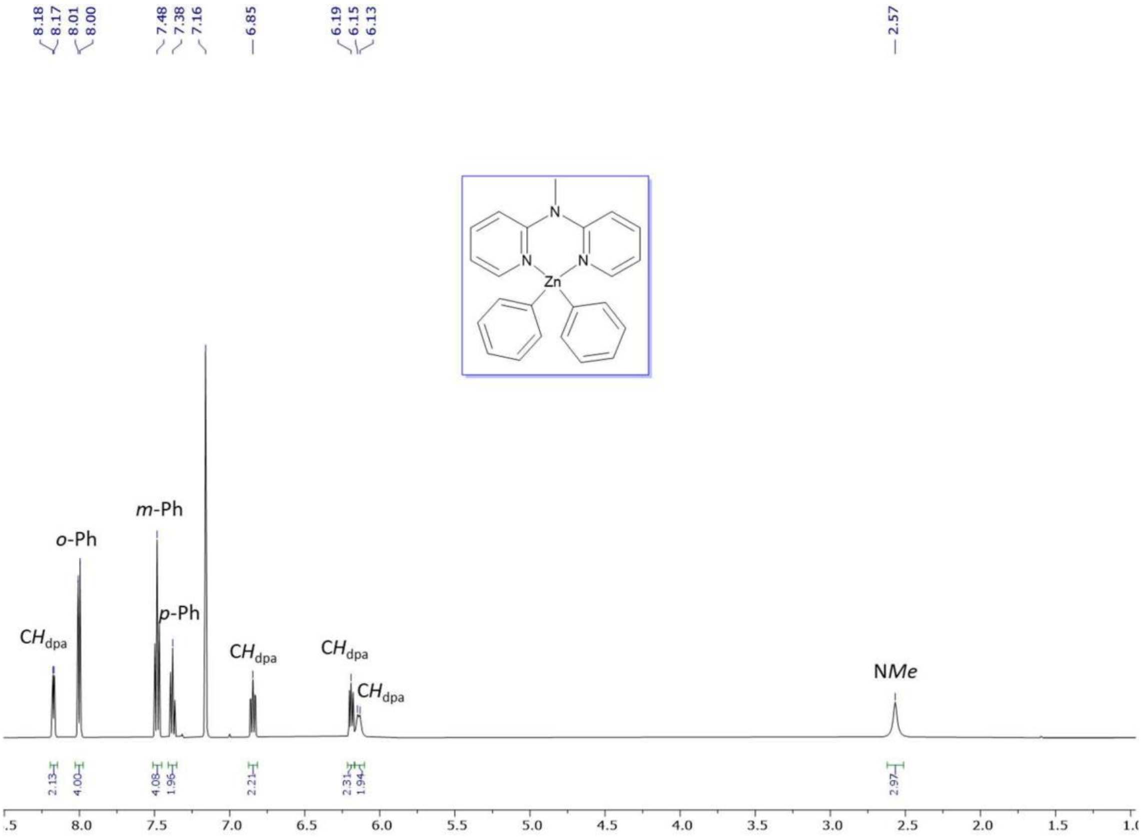

Figure S34. ^{1}H NMR spectrum of **4** in C_6D_6 at room temperature.

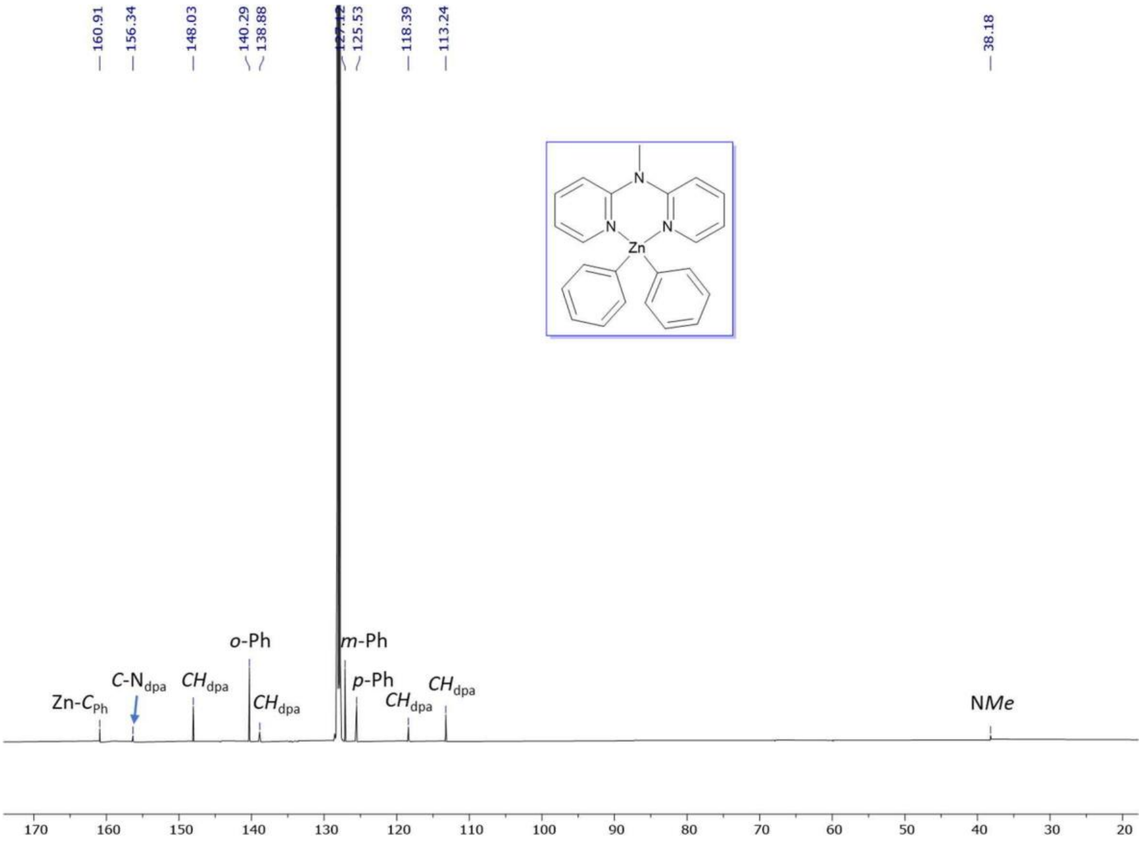

Figure S35. $^{13}C\{^{1}H\}$ NMR spectrum of **4** in C_6D_6 at room temperature.

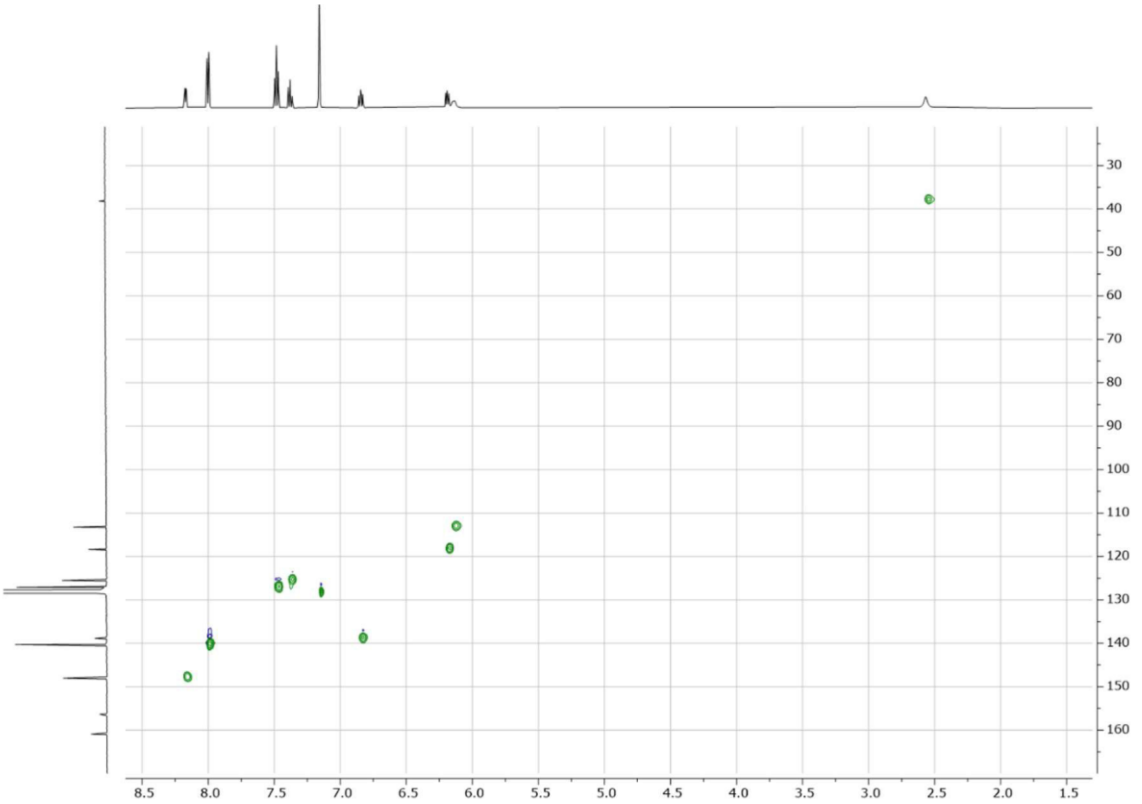

Figure S36. ^{1}H- $^{13}C\{^{1}H\}$ HSQC NMR spectrum of **4** in C_6D_6 at room temperature.

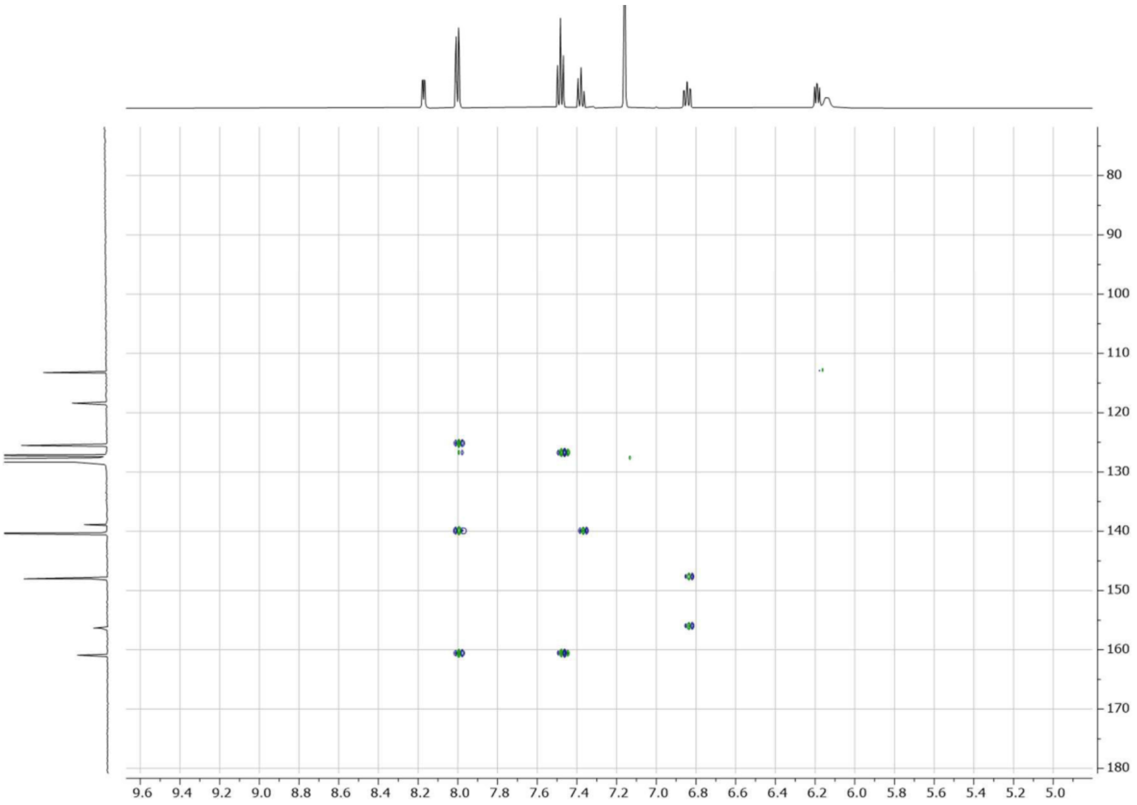

Figure S37. ^{1}H – $^{13}C\{^{1}H\}$ HMBC NMR spectrum of **4** in C_6D_6 at room temperature.

3.5 ^{1}H DOSY NMR comparison of 2 and 4

^{1}H DOSY NMR experiments were conducted in C_6D_6 using the External Calibration Curve (ECC) method at 15µM as described by Stalke.[4] Data was accumulated by linearly varying the diffusion encoding gradients over a range of 2% to 95% for 16 gradient increment values. The signal decay dimension on the pseudo-2D data was generated by Fourier transformation of the time-domain data. The diffusion profile and coefficients were established by use of the DOSY processing features of TopSpin software. Using residual proton signal of solvent as a reference standard we have been able to approximate the aggregates of complexes **2** and **4** as compact spheres (CS) and determine their mean diffusion coefficients (Table S1).

Table S1. Solution-state calculations for complexes **2** and **4** in C_6D_6 using External Calibration Curve.

Complex	Solvent	$D_{analyte}$ [x10^{-10} m^2/s]	$D_{reference}$ [x10^{-9} m^2/s]	MW_{det} [g/mol]	Structure	MW_{calc} [g/mol]	MW_{diff}
**4**	C_6D_6	9.327	2.140	428	[^{Me}dpa·$ZnPh_2$]	405	-5 %
**2**	C_6D_6	5.992	1.764	709	[$(dpaZnPh)_2$]	625	-12%
					[$(dpaZnPh)_3$]	938	32%
					[$(dpaZnPh)_2$] ⇌ [$(dpaZnPh)_3$]	782	10 %

^{1}H DOSY NMR spectrum of **4** revealed co-diffusion of the phenyl and dipyridylamine peaks, suggesting that the solid-state structure of **4** is retained in solution. A mean diffusion coefficient of D = 9.327 x 10^{-10} m^2/s was measured (Figure S39) and determined molecular weight of 428 g/mol correlates well with calculated molecular weight of monomeric complex **4** of 406 g/mol with error of -5%.
A mean diffusion coefficient of D = 5.992 x 10^{-10} m^2/s was measured for complex **2** (Figure S38) and determined molecular weight of 709 g/mol. This value does not correlate well with monomeric constitution (MW_{calc} = 313 g/mol), nor trimeric constitution (MW_{calc} = 938 g/mol, error = 32 %) found

in the solid state. The closest correlation would be with dimeric constitution (MW_{calc} = 625 g/mol, error = -12 %) or with an equilibrium state between dimeric and trimeric constitution (MW_{calc} = 782 g/mol, error = 10 %). Considering that the ECC method employed is valid for MW up to 600 g/mol, the exact structure of **2** in C_6D_6 solution is not clear but it is a strong indication that **2** exists as oligomer(s) in solution.

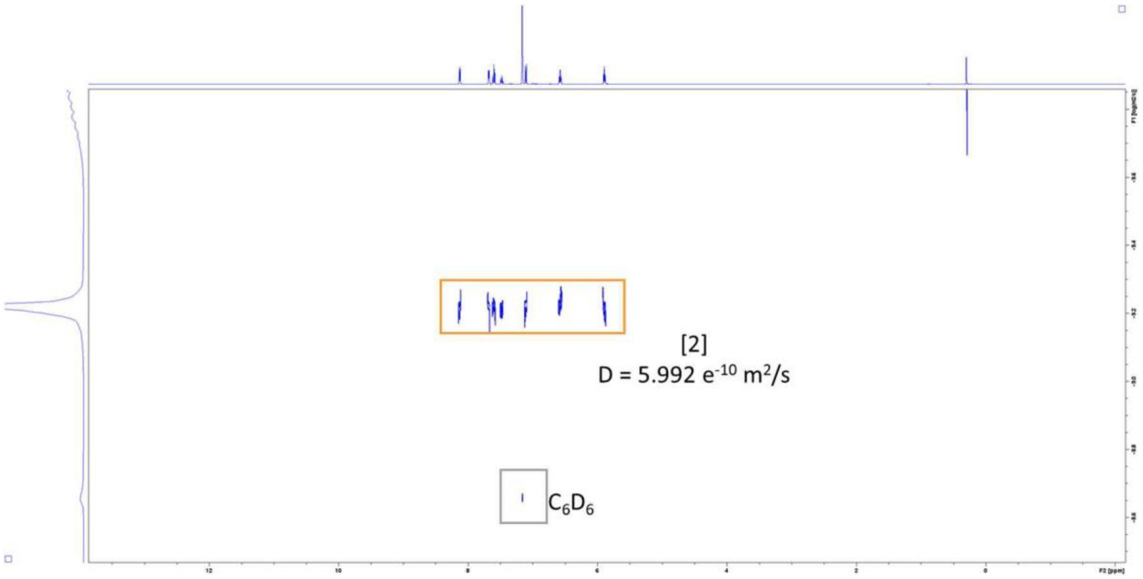

Figure S38. ^{1}H DOSY NMR spectrum of **2** in C_6D_6 at room temperature.

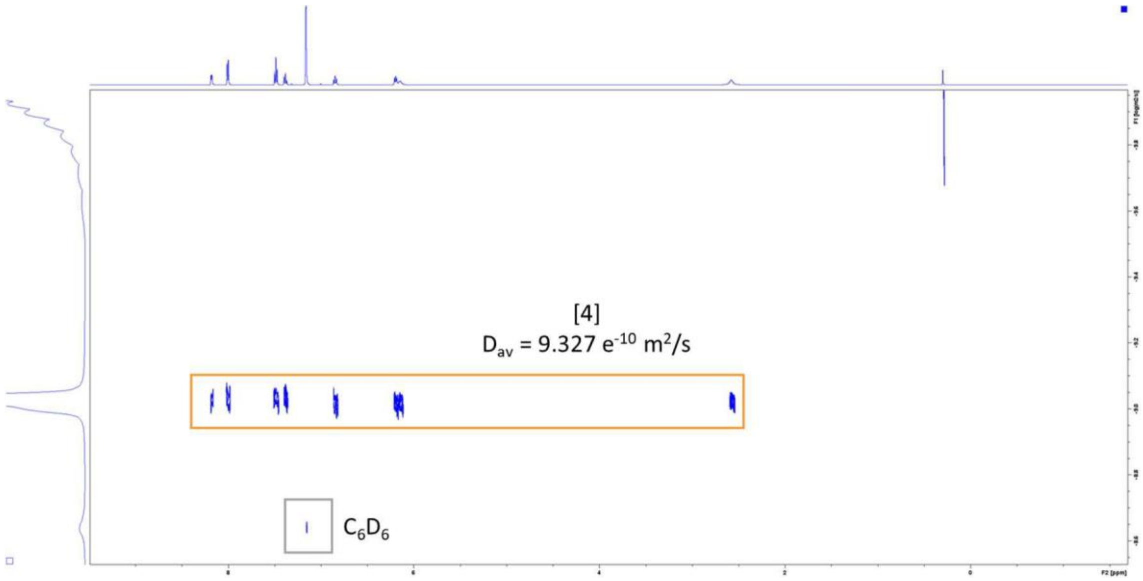

Figure S39. ^{1}H DOSY NMR spectrum of **4** in C_6D_6 at room temperature.

3.6 Conversion of 1 to 3 by addition of PMDETA in THF solution

In order to assess the propensity of PMDETA to bind to the Li-centre of [{(THF)$_3$Li(μ-dpa)ZnPh$_2$}] scaffold by displacing THF, **1** (30 mg, 4.89 x 10^{-5} mol, 1 eq) was loaded into a J Young's NMR tube and dissolved in d_8-THF (0.5 mL). To this solution, PMDETA (10.2 µL, 4.89 x 10^{-5} mol, 1 eq) was added and the reaction mixture was stirred for 2 h. A change was firstly evident in observed precipitation, indicative of formation of **3** which was previously observed to exhibit lower solubility than **1** (in THF,

while **3** is insoluble in C_6D_6). The mixture was analysed by ^{1}H and ^{7}Li NMR spectroscopies (Figure S40-S42) which showed that the starting ate **1** has changed. Furthermore, comparison of obtained spectra with spectra of pure sample of **3** is in excellent agreement.

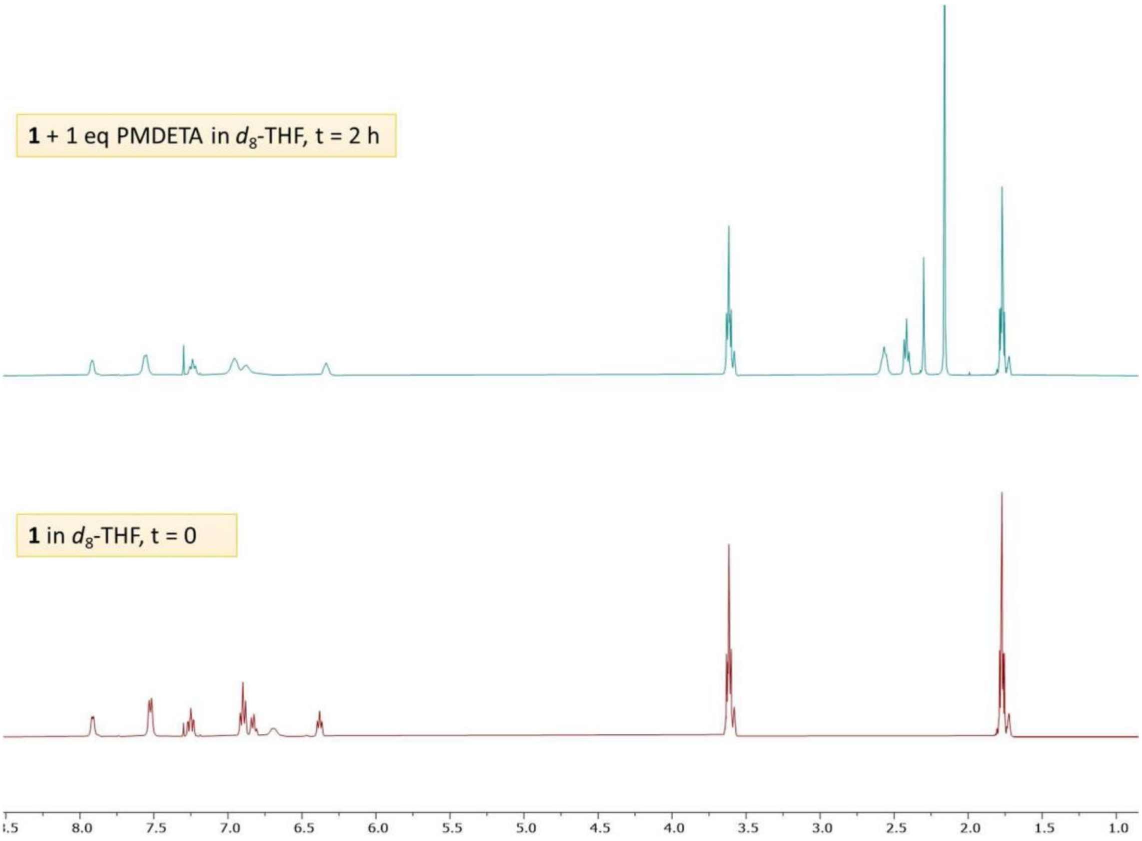

Figure S40. Stacked ^{1}H NMR spectra of **1** (bottom) and **1** with 1 eq of PMDETA (top) in d_8-THF at room temperature.

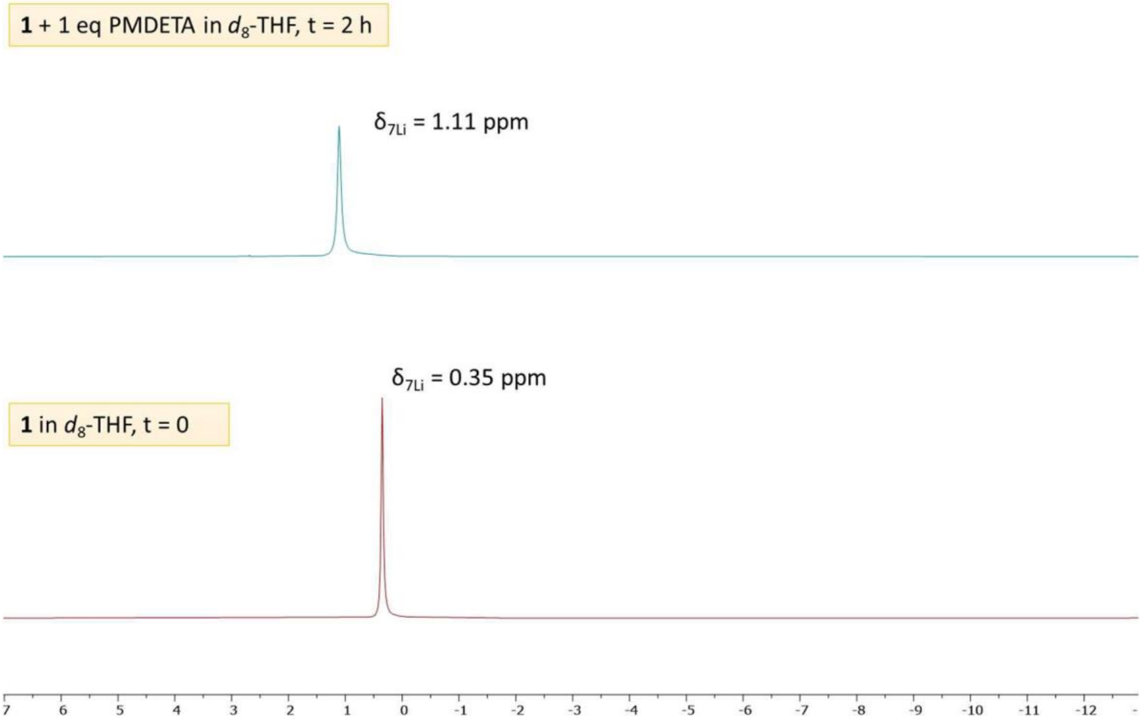

Figure S41. Stacked ^{7}Li NMR spectra of **1** (bottom) and **1** with 1 eq of PMDETA (top) in d_8-THF at room temperature.

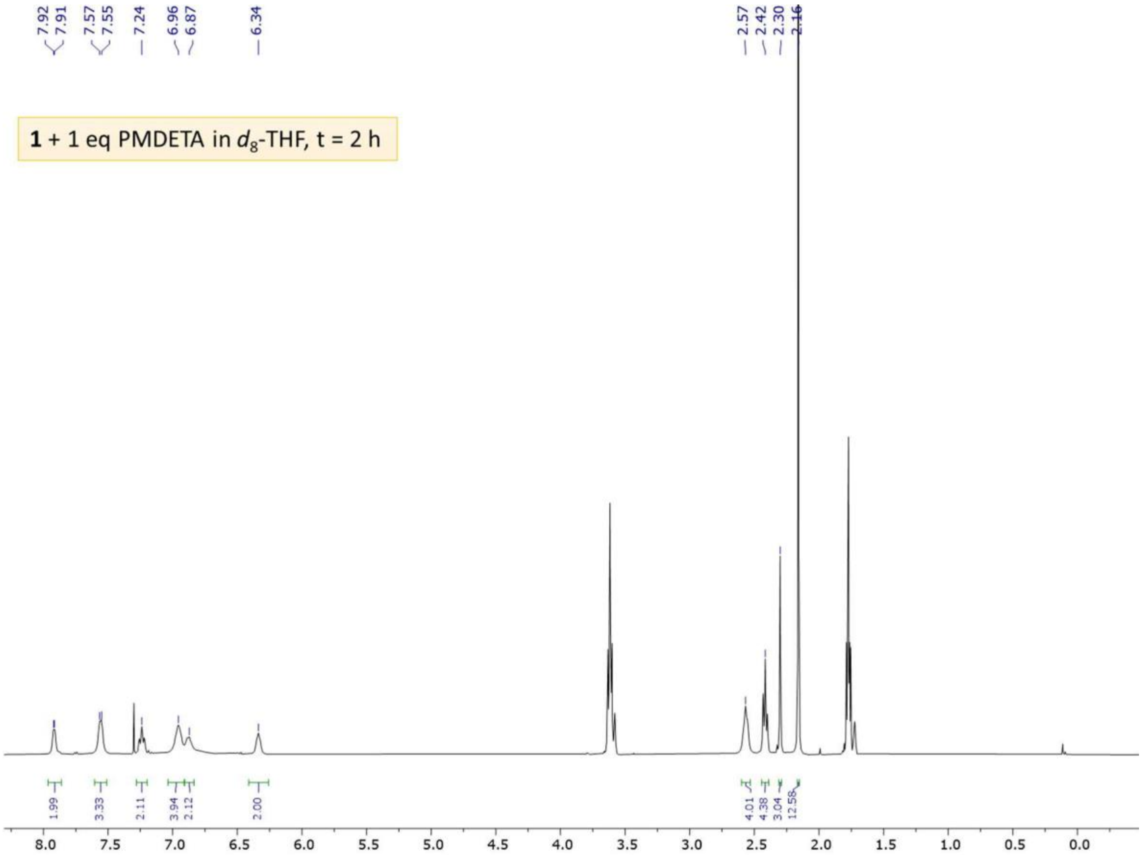

Figure S42. ^{1}H NMR spectrum of **1** with 1 eq of PMDETA in d_8-THF at room temperature.

4. Reactivity studies

4.1 Deprotonation of terminal alkyne and isolation of 5

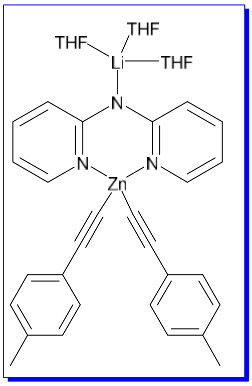

Lithium zincate **1** (30 mg, 4.89 x 10^{-5} mol, 1 eq) was loaded into a J Young's NMR tube and dissolved in d_8-THF (0.5 mL). 4-ethynyltoluene (12.4 µL, 9.79 x 10^{-5} mol, 2 eq) was added and after 2 h stirring at room temperature, the reaction mixture was analysed by ^{1}H and ^{7}Li NMR spectroscopies (Figure S43-S44) which showed formation of **5** as the major species and minor amounts of two more species containing alkynyl substituents. A sharp signal for benzene forming as a side product is also detected in the ^{1}H NMR spectrum. The product crashed out of the solution upon standing at room temperature and a crystal suitable for X-ray measurement was obtained. Poor solubility of crystalline **5** precluded subsequent recording of a meaningful ^{13}C NMR spectrum.

^{1}H NMR (400 MHz, d_8-THF, 298 K): δ 8.07 (br s, 2 H), 7.27 (br s, 2H), 7.10 (d, *J*=7.6, Hz, 4 H), 6.89 (d, *J*=7.4 Hz, 4 H), 6.64 (br s, 2H), 6.47 (br s, 2 H), 2.21 (s, 6 H, CH_3Ph). ^{7}Li NMR (155.5 MHz, d_8-THF, 298 K): 0.21 (s).

In order to compare with other available zinc species, deprotonation of two equivalents of 4-ethynyltoluene with lithium zincate **3** (PMDETA), neutral Ph_2Zn and **4** was probed. Like **1**, **3** exhibits enhanced basicity and after 2 h at room temperature the deprotonation is near complete (Figure S45). There is only trace amount of alkyne left, but due to the poor solubility of the product, the exact amount cannot be determined by integration.

Both neutral organozinc species, Ph_2Zn and **4**, showed sluggish deprotonation. For instance, alkyne deprotonation with **4** was not completed even after a week at room temperature (Figure S46). Relative ratio, based on the integration of the CH_3 resonance of 4-ethynyltoluene, shows about 80% of Zn-alkynyl species and 20% leftover unreacted alkyne even after this time.

Similarly, with Ph_2Zn only extensive heating pushed the deprotonation to completion (Figure S47). Nevertheless, we attempted this as an alternative route to obtain clean product **5**. Once diphenylzinc (22 mg, 0.1 mmol, 1 eq) completed deprotonation of 4-ethynyltoluene (25.4 µL, 0.2 mmol, 2 eq) in d_8-THF, Lidpa (17.7 mg, 0.1 mmol, 1 eq) was added and immediately white solid started crashing out. The product was isolated by filtration and analysed by ^{1}H and ^{7}Li NMR spectroscopy which showed formation of **5** as major species and minor amounts of two more species containing alkynyl substituent.

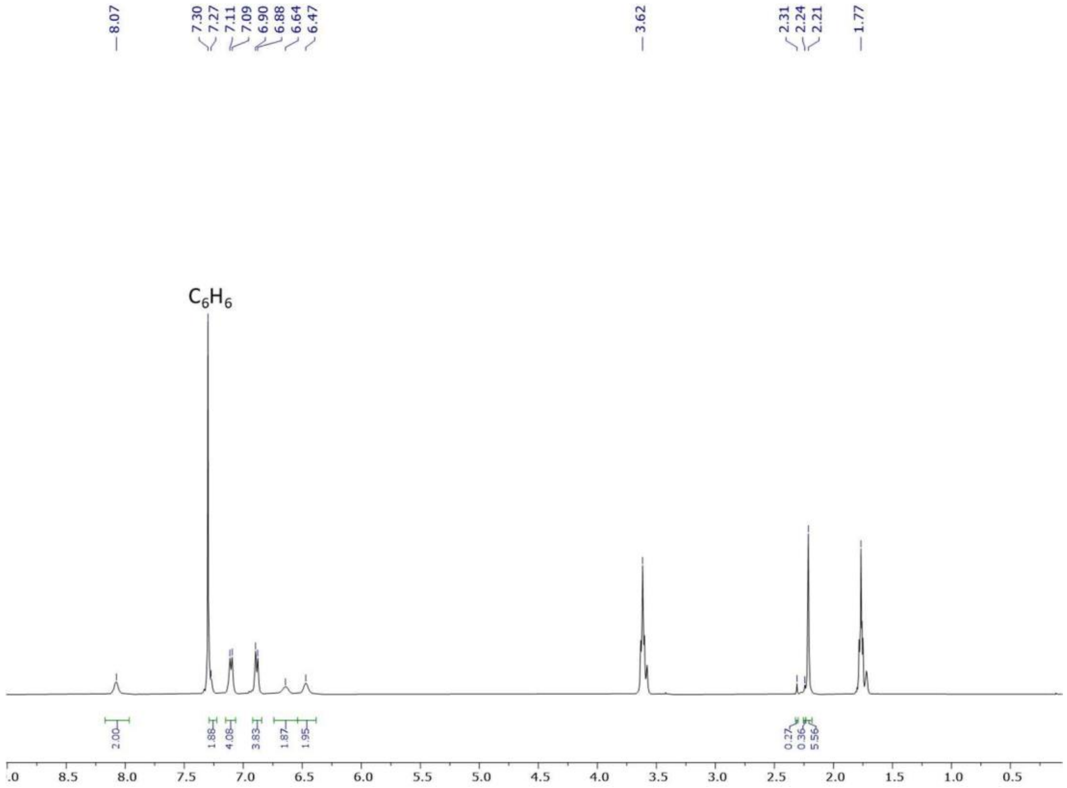

Figure S43. ^{1}H NMR spectrum of *in situ* deprotonation of 2 eq of 4-ethynyltoluene by **1** affording **5** as a major product in d_8-THF at room temperature.

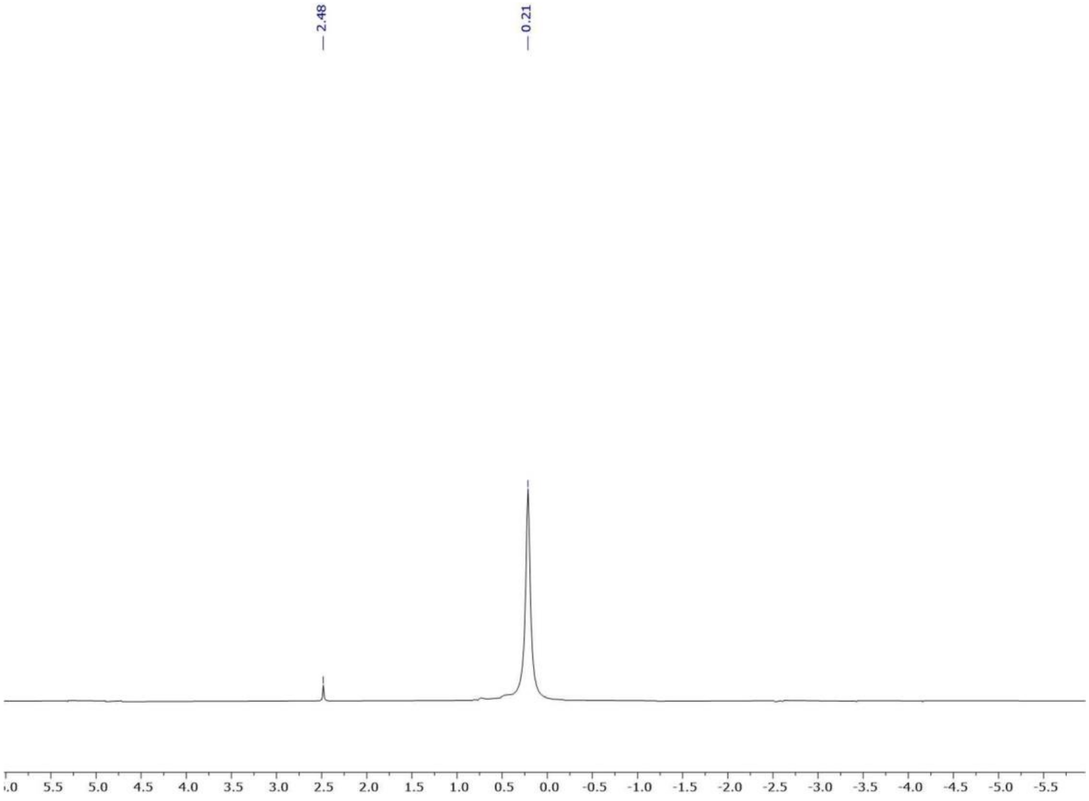

Figure S44. ^{7}Li NMR spectrum of *in situ* deprotonation of 2 eq of 4-ethynyltoluene by **1** affording **5** as a major product in d_8-THF at room temperature.

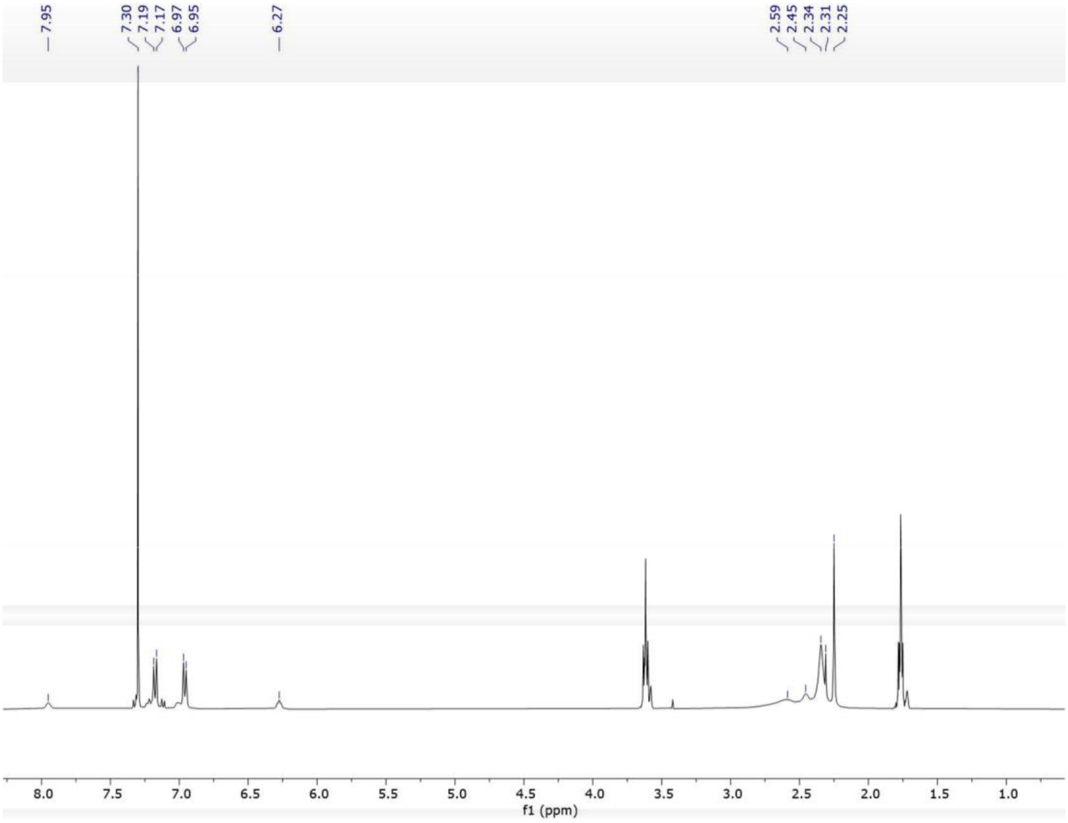

Figure S45. ^1H NMR spectrum of deprotonation of 2 eq of 4-ethynyltoluene with **3** in d_8-THF after 2 h at room temperature.

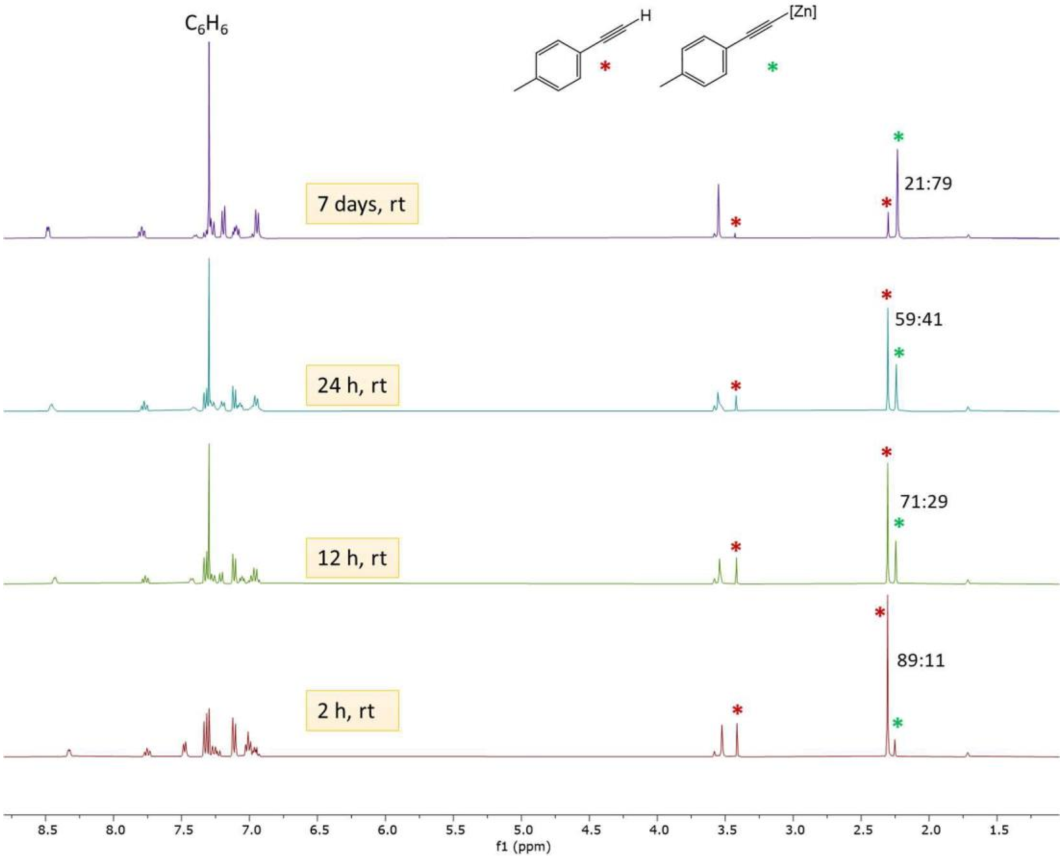

Figure S46. Stacked ^1H NMR spectra of monitoring of deprotonation of 2 eq of 4-ethynyltoluene with **4** in d_8-THF with relative ratios of free alkyne and Zn-alkynyl species determined based on the CH_3 group labelled.

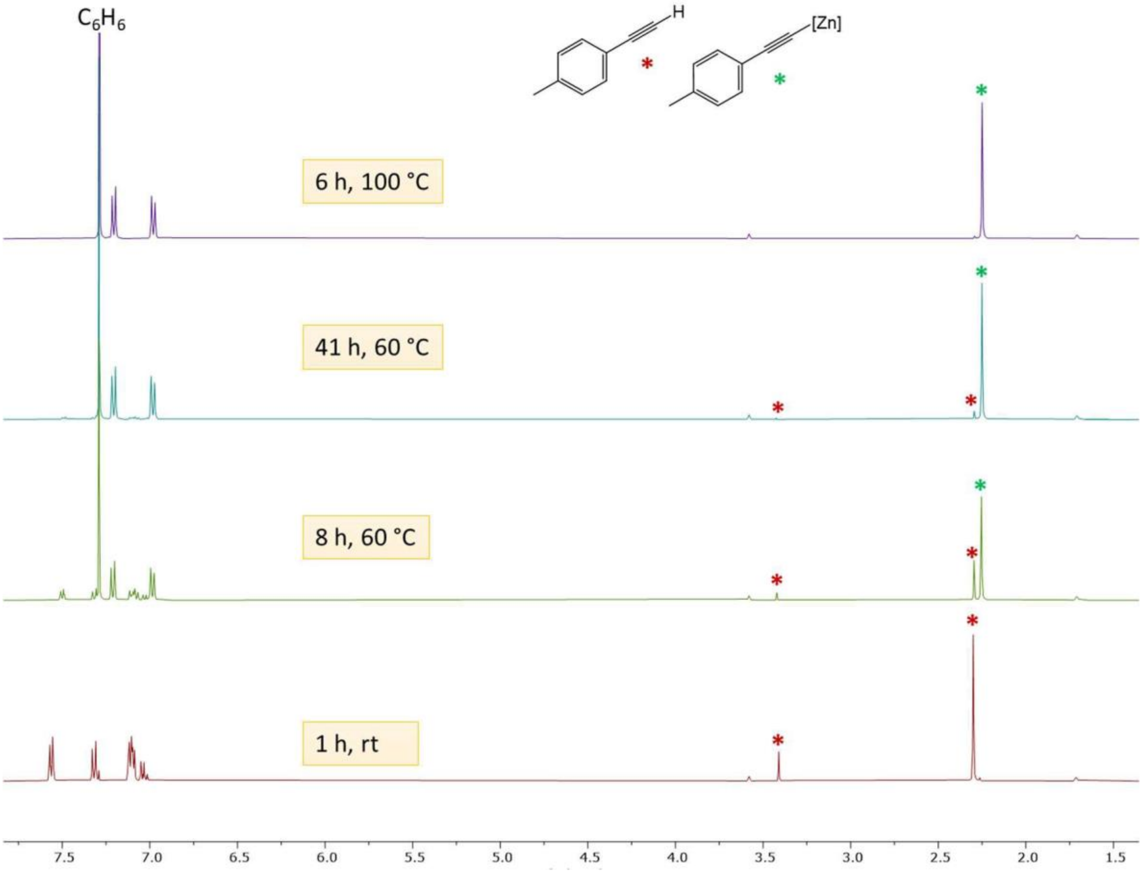

Figure S47. Stacked ^1H NMR spectra of monitoring of deprotonation of 2 eq of 4-ethynyltoluene with Ph_2Zn in d_8-THF.

4.2 Arylation of α,α,α-trifluoroacetophenone and synthesis of 6

Compound **1** (100 mg, 1.63 x 10^{-4} mol, 1 eq) was placed in a Schlenk tube and dissolved in dry benzene (2 mL). To this solution, α,α,α-trifluoroacetophenone (23 µL, 1.63 x 10^{-4} mol, 1 eq) was added and after 2 h stirring at room temperature, the reaction mixture was quenched with HCl (1M, 10 mL) and left stirring overnight. The colourless solution was diluted with water and extracted with diethyl ether (3 x 3 mL). Combined organic layers were dried over Na_2SO_4 and volatiles removed under vacuum. The obtained crude was dissolved in $CDCl_3$ (Figure S50–S51) to which CH_2Br_2 (11.4 µL, 1.63x10^{-4} mol, 1 eq) was added to determine NMR yield (71%).

^1H NMR (500 MHz, $CDCl_3$, 298 K): 2.89 (b, 1H); 7.35 – 7.38 (m, 6H); 7.49 – 7.52 (m, 4H). ^{19}F NMR (376.4 MHz, $CDCl_3$, 298 K): -74.5. Data in agreement with literature values.[5]

In order to gain understanding on organometallic species formed pre acid quench, the reaction was repeated in a J Young's NMR tube. Lithium zincate **1** (30 mg, 4.89 x 10^{-5} mol, 1 eq) was loaded into a J Young's NMR tube and dissolved in C_6D_6 (0.5 mL). α,α,α-trifluoroacetophenone (7 µL, 4.89 x 10^{-5} mol, 1 eq) was added and after 2 h stirring at room temperature, the reaction mixture was analysed by ^1H and ^{19}F NMR spectroscopy (Figure S48-S49) which showed formation of new species assigned as Li-OC(CF_3)Ph_2 and formation of complex **2**.

The identity of Li-OC(CF_3)Ph_2 was confirmed by independent preparation. Phenyllithium (16.8 mg, 0.2 mmol, 1 eq) was loaded into a J Young's NMR tube and suspended in C_6D_6 (0.5 mL). α,α,α-trifluoroacetophenone (28 µL, 0.2 mmol, 1 eq) was added and after 2 h stirring at room temperature,

the reaction mixture was analysed by ^{1}H and ^{19}F NMR spectroscopy (Figure S52-S53) which showed formation of Li-OC(CF_3)Ph_2 along with some leftover substrate.

In order to compare the reactivity of **1**, with other prepared zinc species, complexes **2**-**4** were reacted with α,α,α-trifluoroacetophenone in C_6D_6 at room temperature. Unlike in the deprotonation of alkyne, lithium zincate **3** does not display the same reactivity as lithium zincate **1** in this case. Lithium zincate **3** (30 mg, 5.26 x 10^{-5} mol, 1 eq) was loaded into a J Young's NMR tube and suspended in C_6D_6 (0.5 mL). α,α,α-trifluoroacetophenone (14.8 µL, 1.0 x 10^{-4} mol, 2 eq) was added and after 2 h stirring at room temperature, the reaction mixture was analysed by ^{1}H and ^{19}F NMR spectroscopies (Figure S54-S55) which showed only starting material present as a fluorine-containing species.

The reaction between neutral zinc reagent **2** and one equivalent of α,α,α-trifluoroacetophenone afforded no reaction even after stirring for 5 days at room temperature as evidenced by ^{1}H and ^{19}F NMR spectroscopies (Figure S56-S57). The same outcome was evident if neutral monomeric zinc reagent **4** was reacted with two equivalents of the substrate (Figure S58-S59). It should be noted that the seemingly incorrect stoichiometry in this final reaction is caused by poor solubility of **4** in C_6D_6.

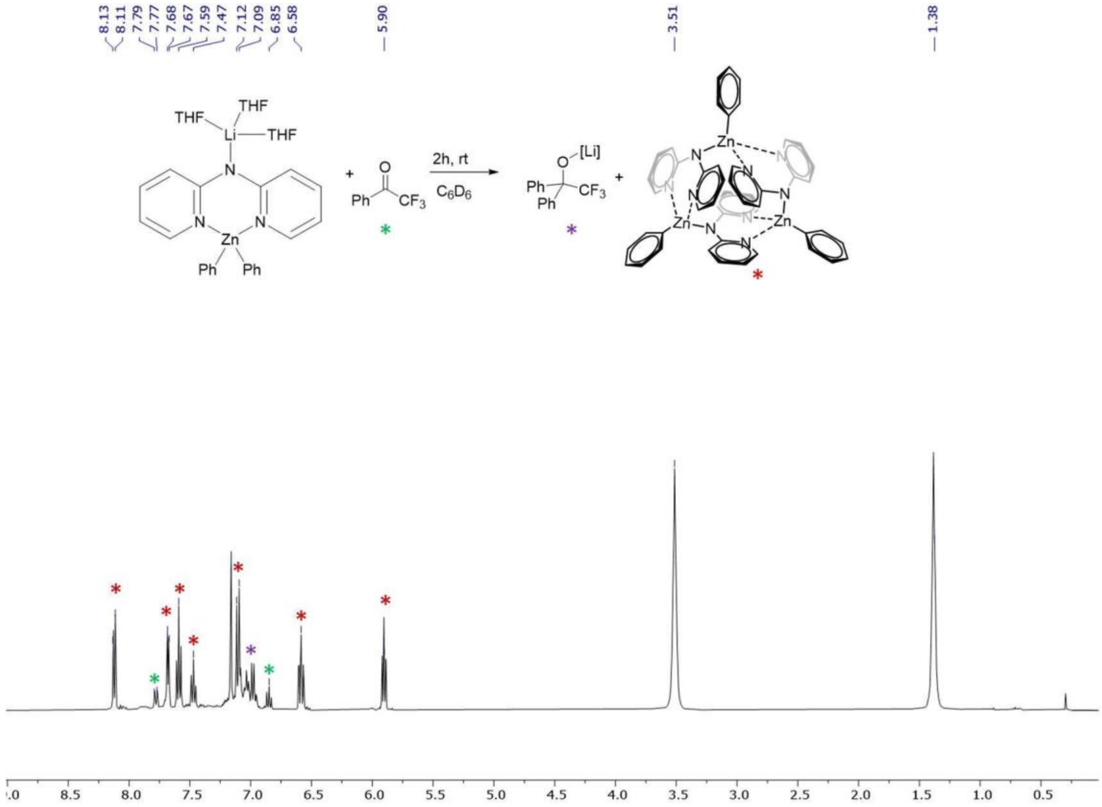

Figure S48. ^{1}H NMR spectrum of reaction mixture of **1** with 1 eq of α,α,α-trifluoroacetophenone in C_6D_6 at room temperature.

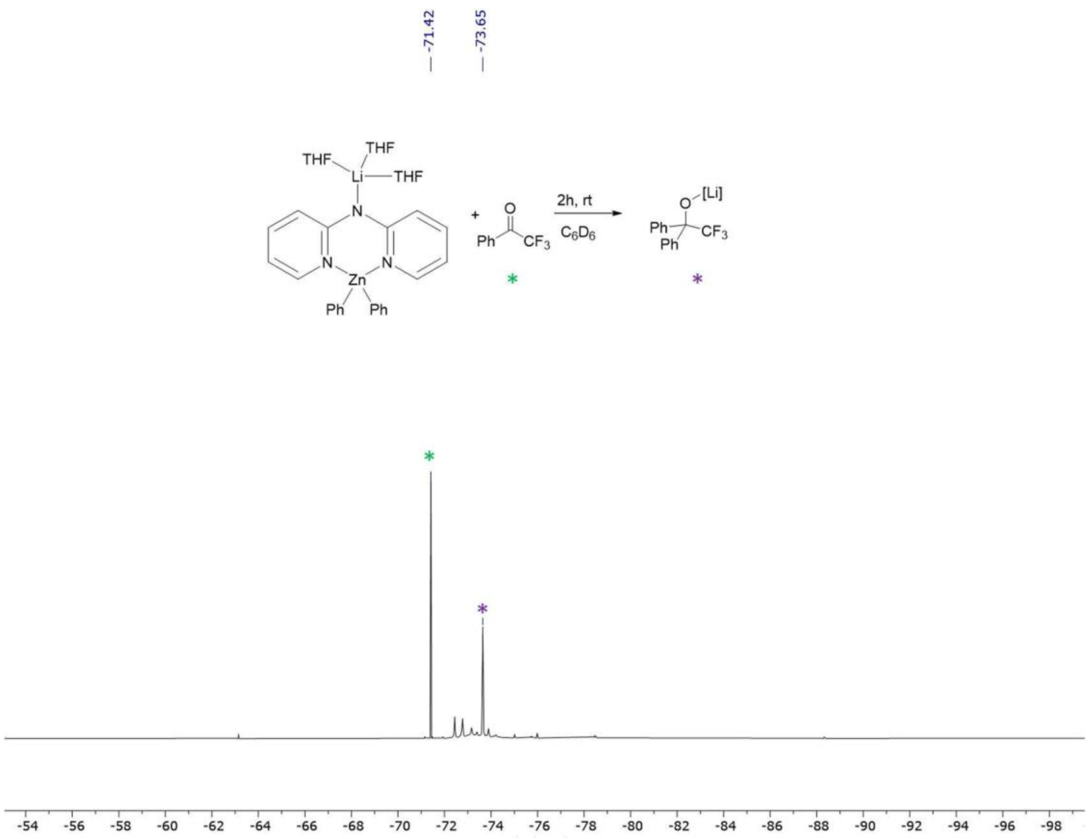

Figure S49. ^{19}F NMR spectrum of reaction mixture of **1** with 1 eq of α,α,α-trifluoroacetophenone in C_6D_6 at room temperature.

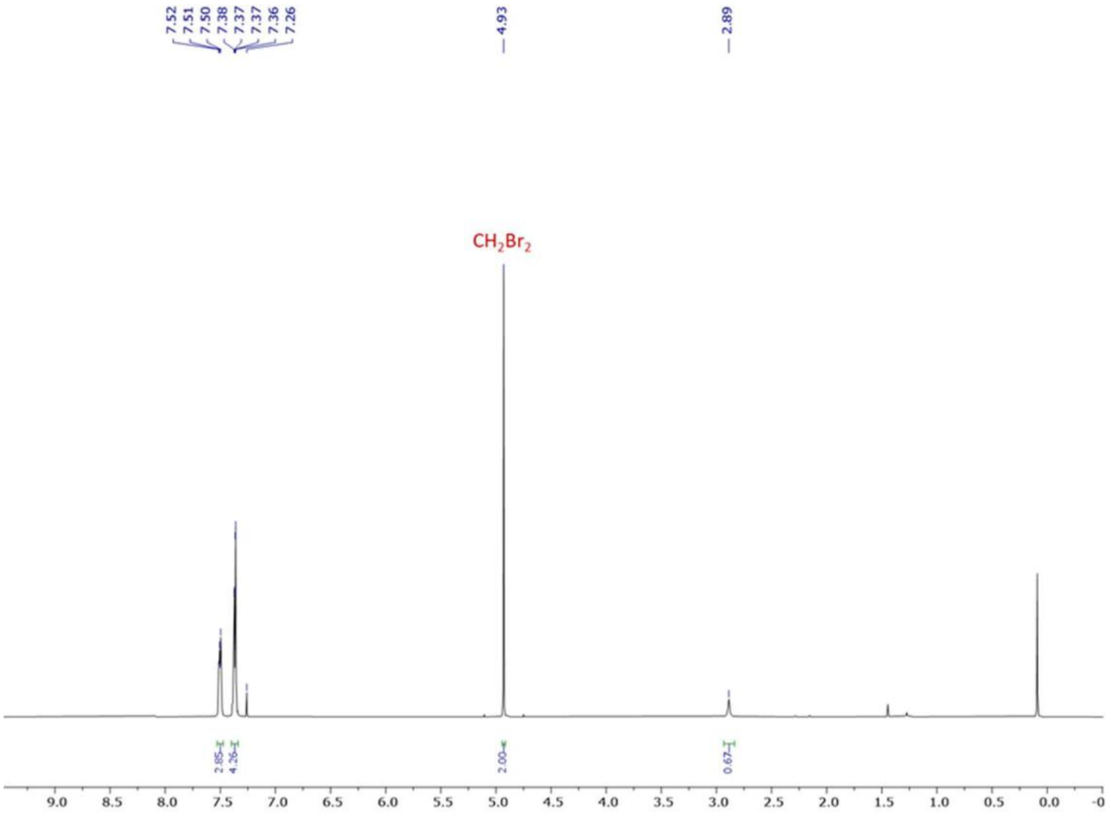

Figure S50. ^{1}H NMR spectrum of the crude product **6** (71%) using CH_2Br_2 as internal standard in $CDCl_3$ at room temperature.

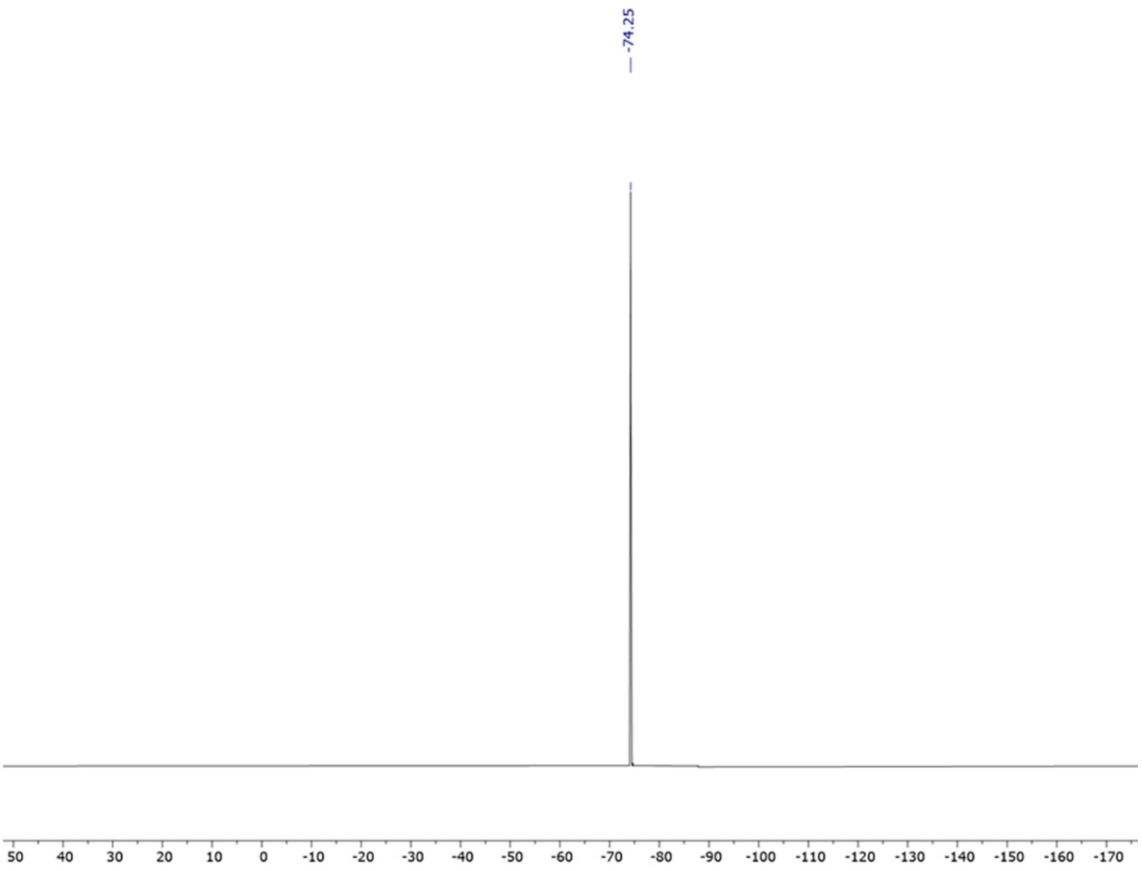

Figure S51. ^{19}F NMR spectrum of crude reaction product **6** in $CDCl_3$ at room temperature.

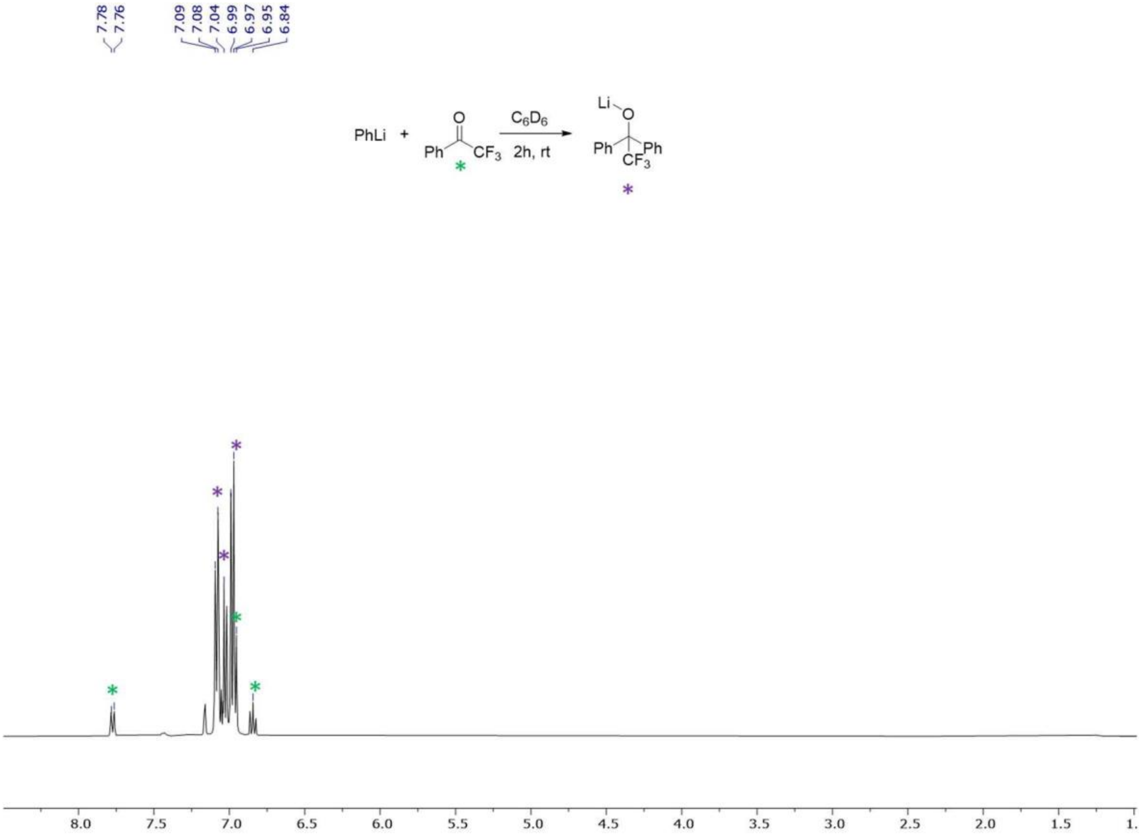

Figure S52. ^{1}H NMR spectrum of reaction mixture of PhLi with α,α,α-trifluoroacetophenone in C_6D_6 after 2h at room temperature.

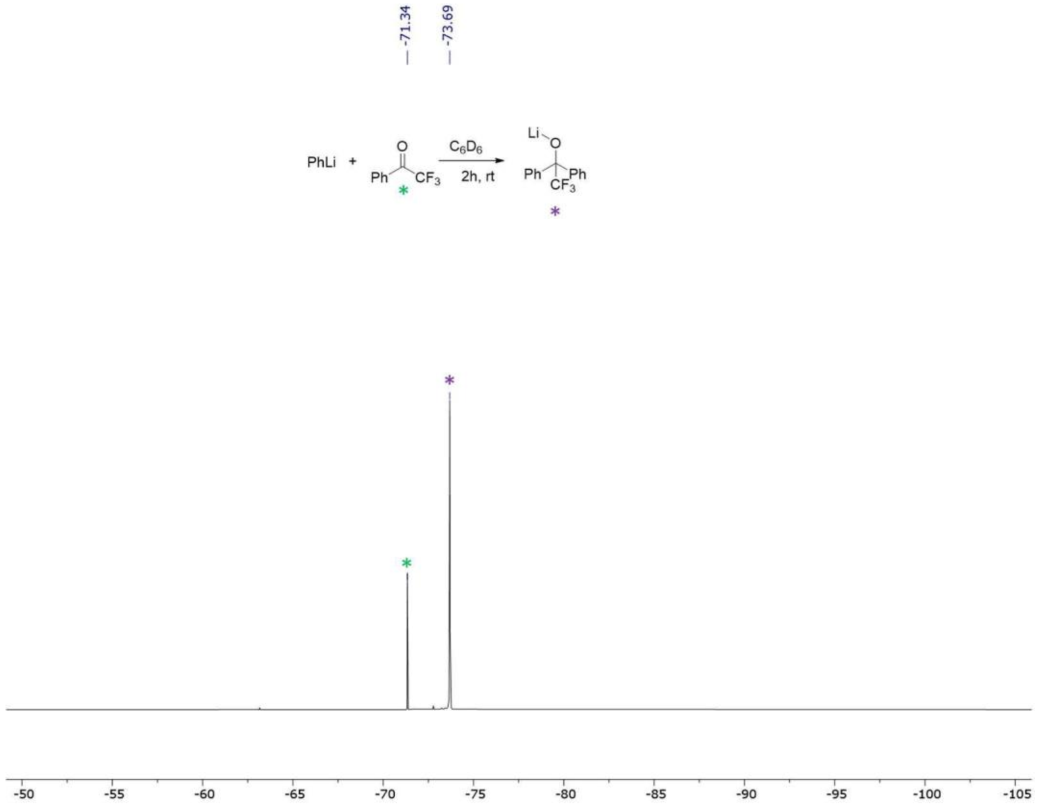

Figure S53. ^{19}F NMR spectrum of reaction mixture of PhLi with α,α,α-trifluoroacetophenone in C_6D_6 after 2h at room temperature.

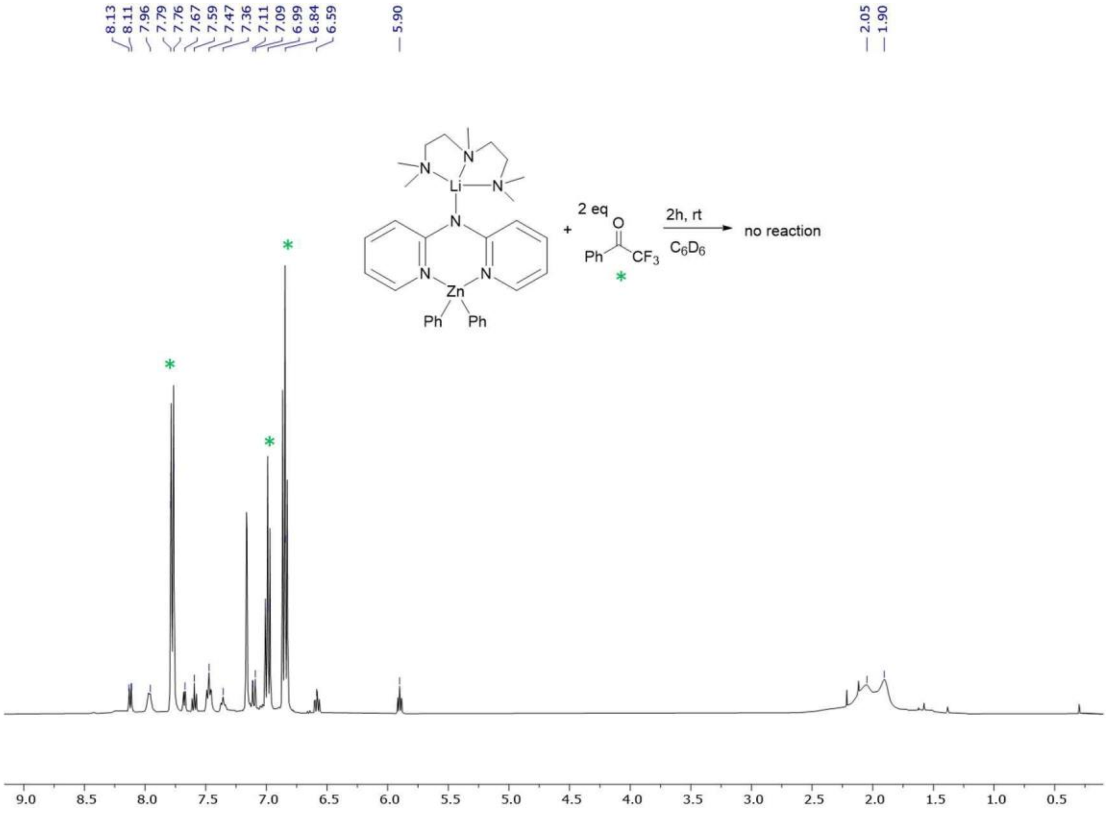

Figure S54. ^{1}H NMR spectrum of reaction mixture of **3** with 2 eq of α,α,α-trifluoroacetophenone in C_6D_6 at room temperature.

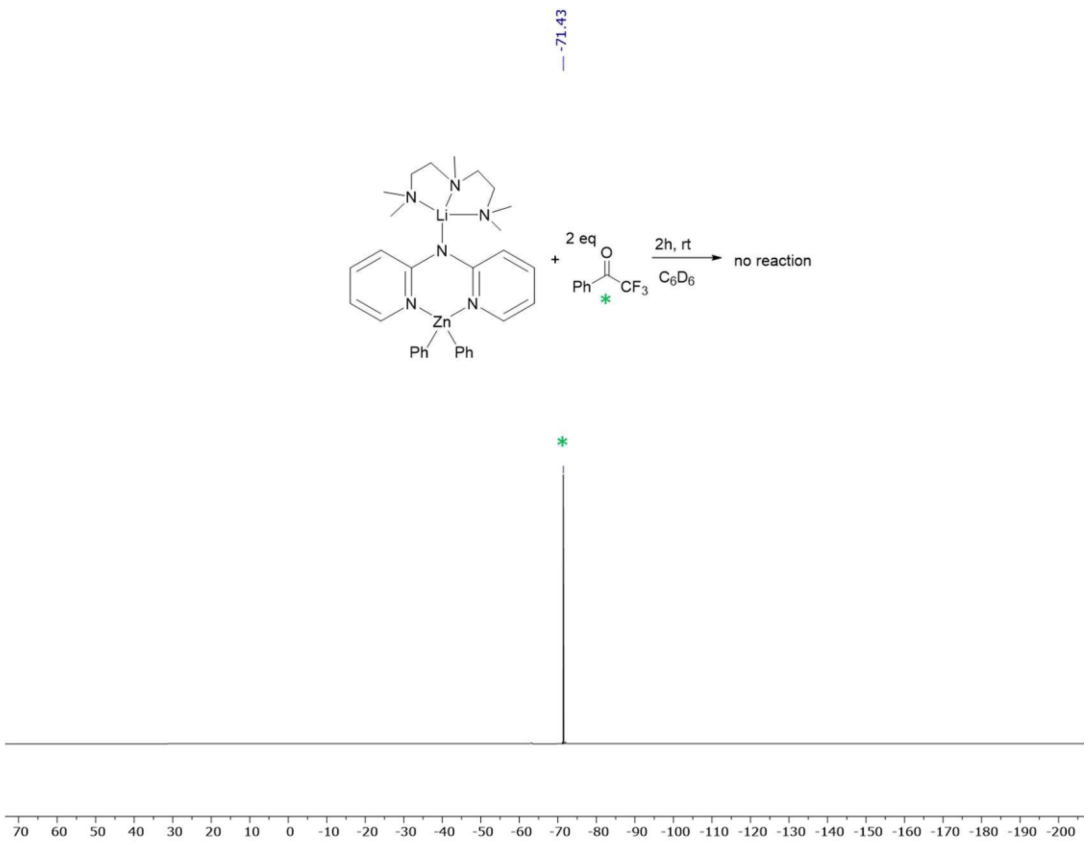

Figure S55. ^{1}H NMR spectrum of reaction of **3** with 2 eq of α,α,α-trifluoroacetophenone in C_6D_6 at room temperature.

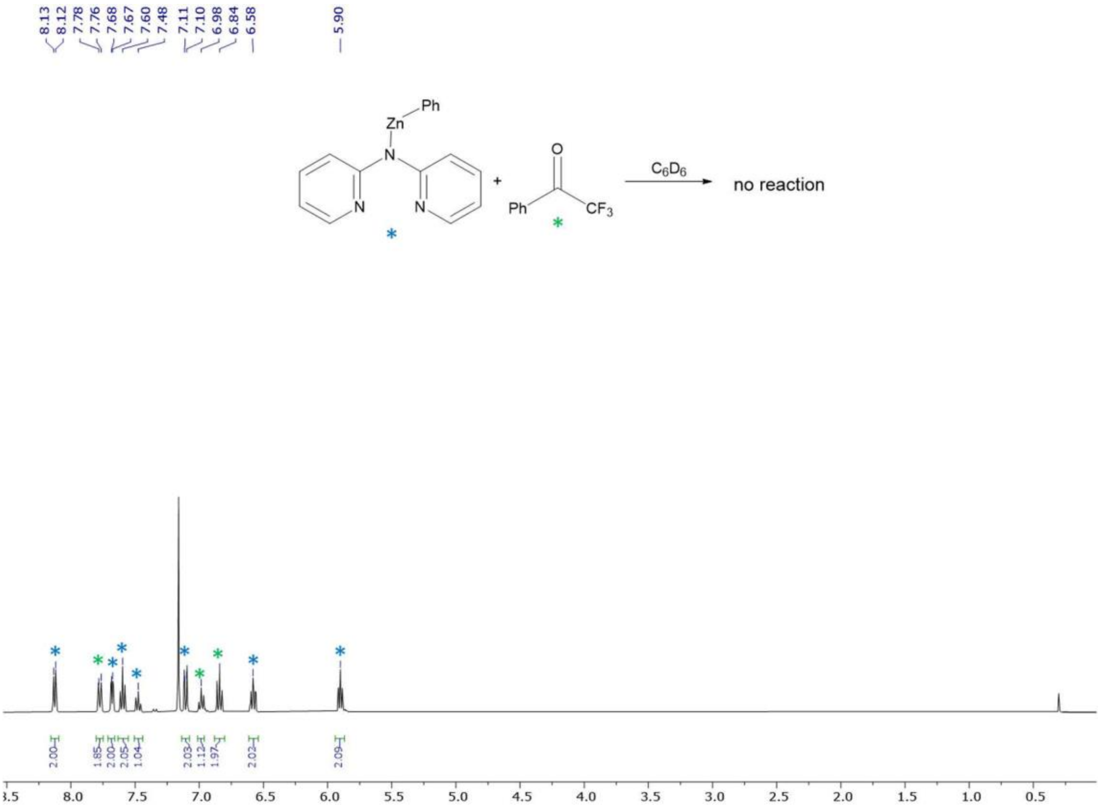

Figure S56. ^{1}H NMR spectrum of reaction of **2** with α,α,α-trifluoroacetophenone in C_6D_6 at room temperature after 5 days.

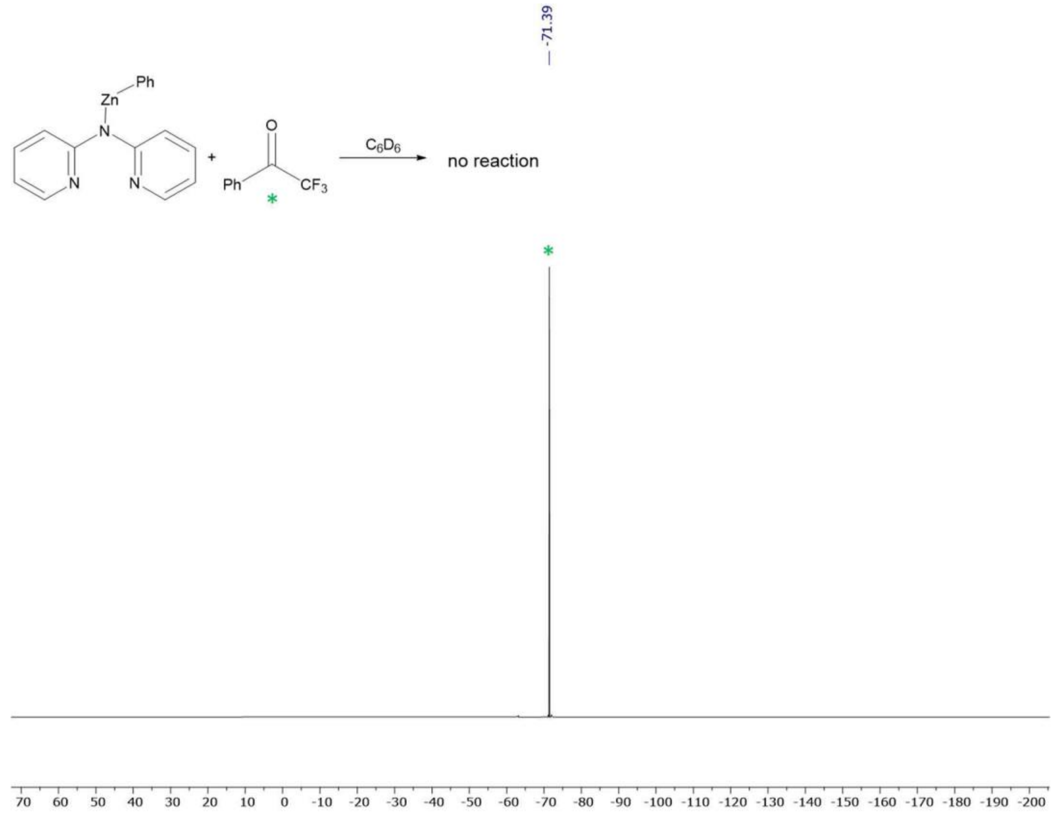

Figure S57. ^{19}F NMR spectrum of reaction of **2** with α,α,α-trifluoroacetophenone in C_6D_6 at room temperature after 5 days.

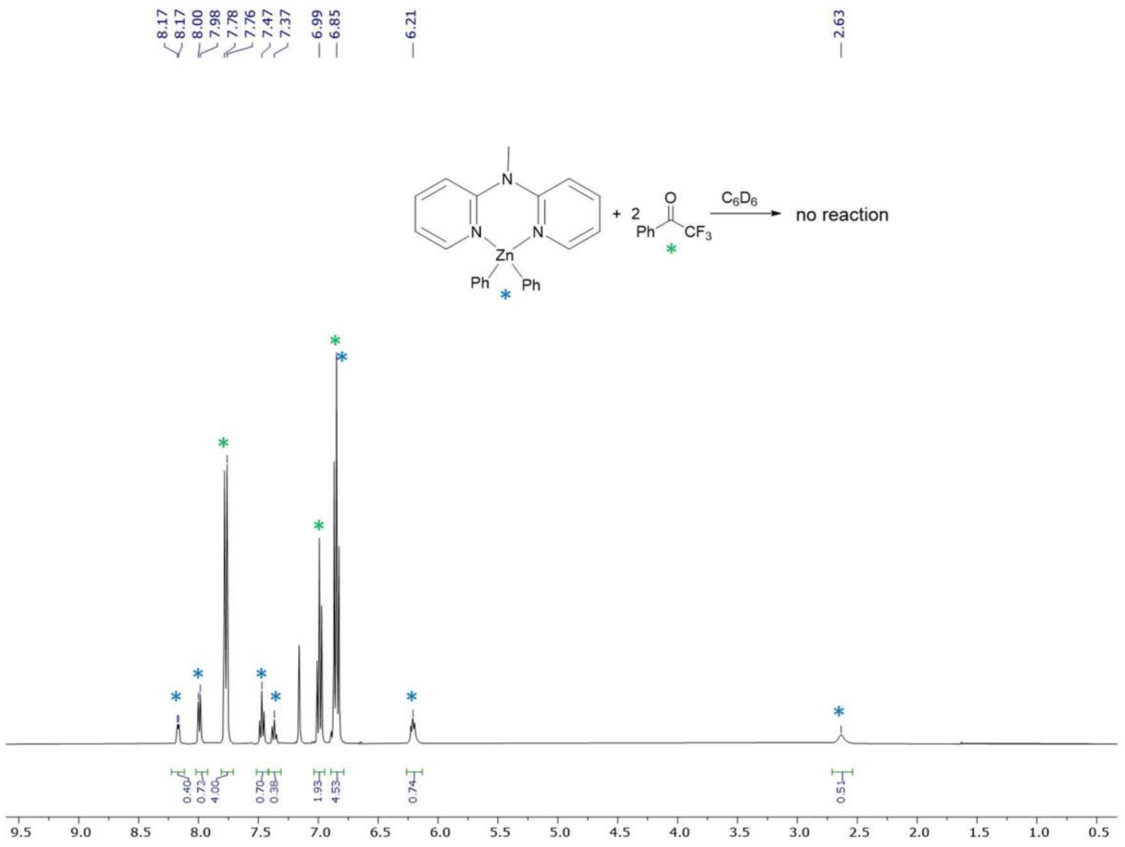

Figure S58. ^{1}H NMR spectrum of reaction of **4** with α,α,α-trifluoroacetophenone in C_6D_6 at room temperature after 5 days.

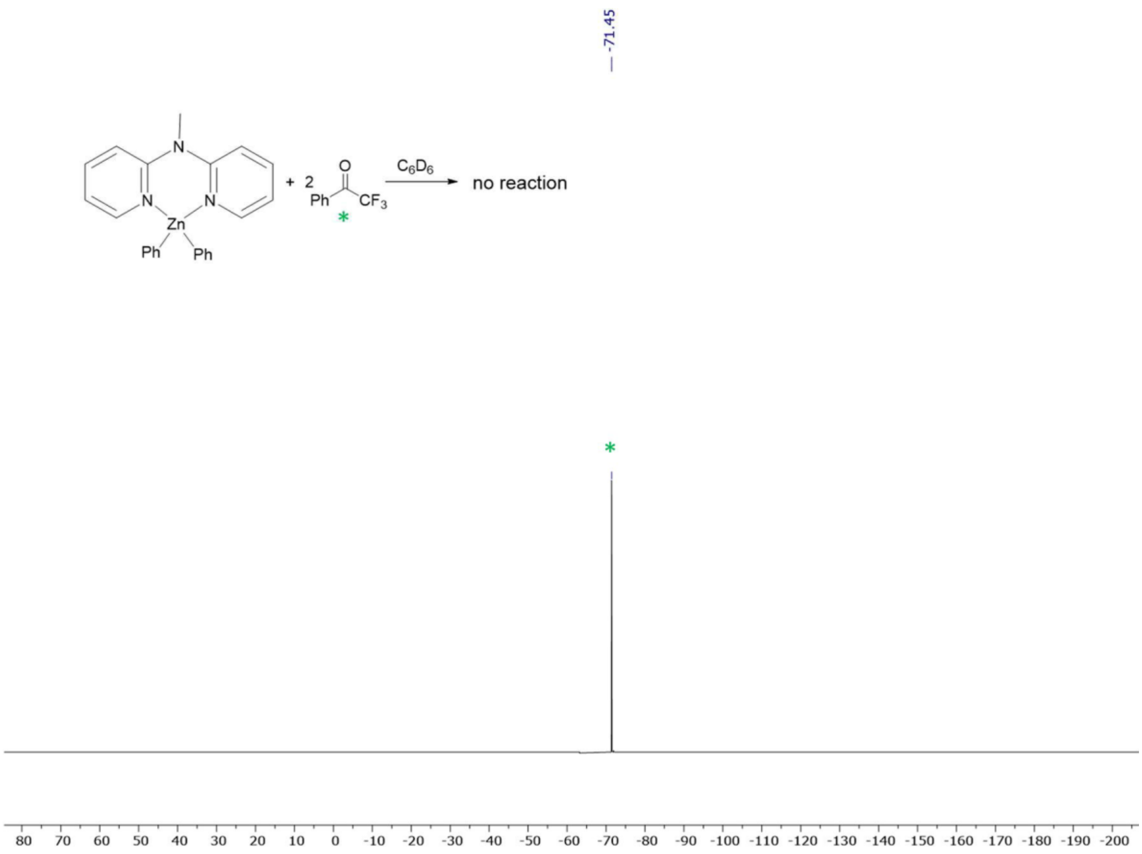

Figure S59. ^{19}F NMR spectrum of reaction of **4** with α,α,α-trifluoroacetophenone in C_6D_6 at room temperature after 5 days.

5. Reactivity studies on formation of hydride species

5.1 Reactivity of 1 towards HBPin

A J Young's NMR tube was charged with **1** (30 mg, 4.89 x 10^{-5} mol, 1 eq) followed by the addition of C_6D_6 (0.5 mL) and HBPin (7.1 µl, 4.89 x 10^{-5} mol, 1 eq). The sealed tube was spun at room temperature and checked by NMR spectroscopy after 15 min (Figure S60-S62). The same reaction was performed in d_8-toluene by adding HBPin at -40 °C (Figure S64-S65) and immediately recording the NMR spectra. Analysis of the data shows that in both instances the outcome is the same – no free HBPin is observed and it is transformed (mostly) into PhBPin, along with the formation of two Zn-H containing species (**7A** and **7B**) in approximately 2:1 ratio. Repeating the NMR analysis after 1 h or overnight revealed no visible change. ^{1}H DOSY NMR analysis showed that **7A** and **7B** have similar diffusion coefficients (Figure S63) confirming that they are both molecular zinc hydrides containing dpaZn units.

The reaction also was probed in THF as solvent, this gave significantly more complex outcomes. For example - a J Young's NMR tube was charged with **1** (30 mg, 4.89 x 10^{-5} mol, 1 eq) followed by the addition of THF (0.5 mL) and HBPin (7.1 µl, 4.89 x 10^{-5} mol, 1 eq or 14.2 µl, 9.79 x 10^{-5} mol, 2 eq). The sealed tube was spun at room temperature and checked by NMR spectroscopy after 1 h. Analysis of the data showed that no free HBpin is observed but only one resonance for Zn-H is observed. The formation of PhBPin is again dominant, but more ligand redistribution occurs (Figure S66-S67) than in aromatic solvents (i.e. benzene or toluene) and this is further increased in case of 2 eq. HBPin (Figure S68-S70). Repeating the reaction in THF with 2 eq of DBPin affords spectra (Figure S71-S74) in agreement with the ones obtained when using HBPin but with a difference in the absence of any resonances in the 4.5 – 6.5 ppm region in the ^{1}H NMR spectrum where we observed resonances

assigned to Zn-H. Furthermore, in the ^{2}H NMR spectrum, a resonance at 4.98 ppm is now observed. In ^{11}B and ^{7}Li NMR spectra, the resonances are again comparable to the HBPin counterpart.

Going back to arenes as solvent, the reaction was repeated with excess HBPin. A J Young's NMR tube was charged with **1** (30 mg, 4.89 x 10^{-5} mol, 1 eq) followed by the addition of C_6D_6 (0.5 mL), and hexamethylbenzene (8 mg, 4.89 x 10^{-5} mol, 1 eq) as internal standard. The reaction mixture was checked by ^{1}H NMR spectroscopy showing that clean starting material **1** and internal standard are present in a 1:1 ratio. Then, HBPin (14.2 µl, 9.78 x 10^{-5} mol, 2 eq) was added and immediately (5 min) analysed by ^{1}H and ^{11}B NMR spectroscopy, followed by further analysis after 15 and 30 min (Figure S77) which showed that the reaction is complete after 5 min and no change is observed within next hour. Further analysis revealed major products to be PhBPin and Zn-H species **7B**, both present in 70% against internal standard (Figure S75-S76). Multinuclear NMR spectroscopy (including 2D COSY, HSQC, HMBC and ^{1}H DOSY experiments) imply that a dpa-ZnPh(H) species is formed (Figure S78-S84).

If the excess of HBPin is increased further, to 3 eq, the same outcome is evident – two equivalents of HBPin are consumed and an equivalent is left unreacted, as evidenced in both ^{1}H and ^{11}B NMR spectra (Figure S85-S86). The reaction is again fast and no change is observed on standing overnight (Figure S87-S88).

Attempts to observe and more accurately identify the two Zn-H species observed in arene solvents by mass spectrometry were unsuccessful. The key identifiable fragment from multiple MS experiments was:

(MS-EI^{+}) found: 311.04208 [$C_{16}H_{13}N_3Zn$ = dpaZnPh], simulated 311.03954

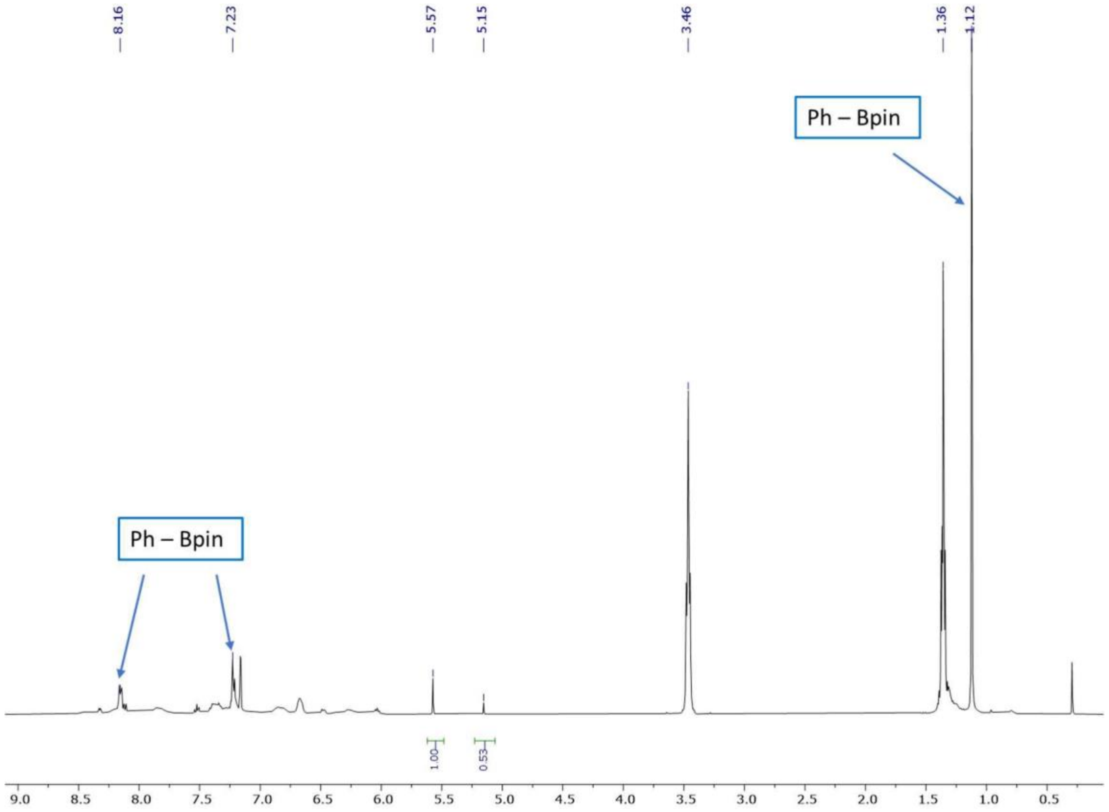

Figure S60. ^{1}H NMR spectrum of reaction of **1** with 1 eq of HBPin in C_6D_6 at room temperature.

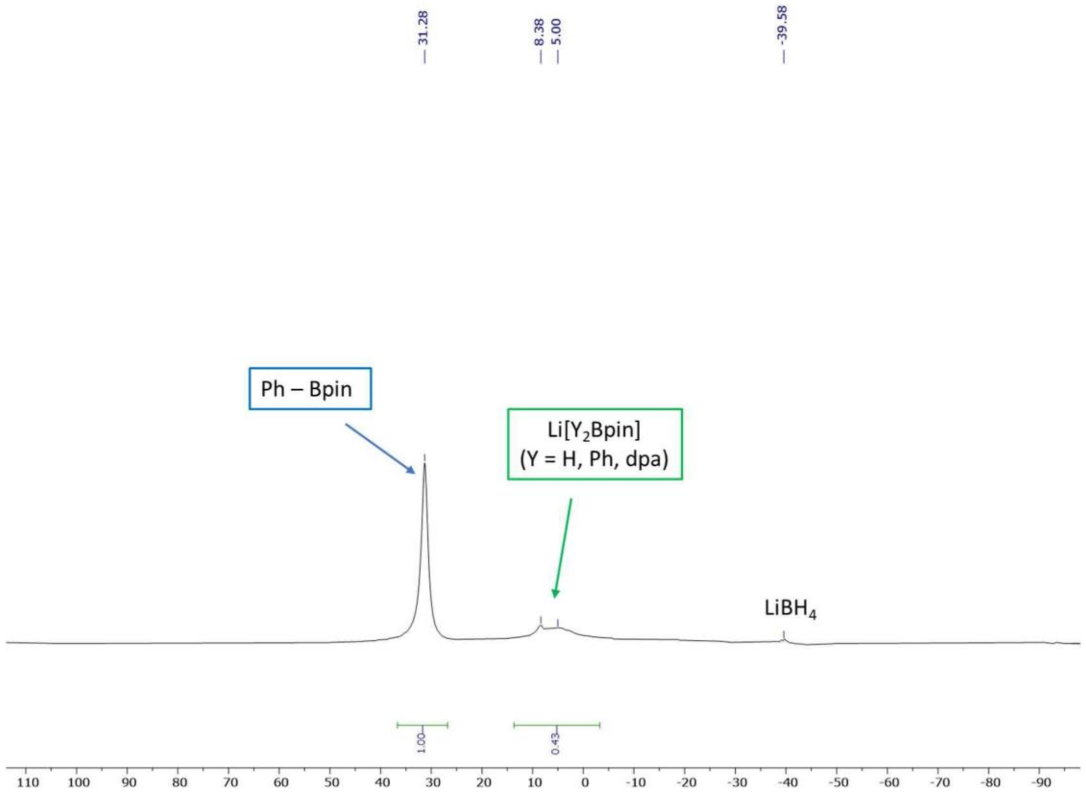

Figure S61. ^{11}B NMR spectrum of reaction of **1** with 1 eq of HBPin in C_6D_6 at room temperature.

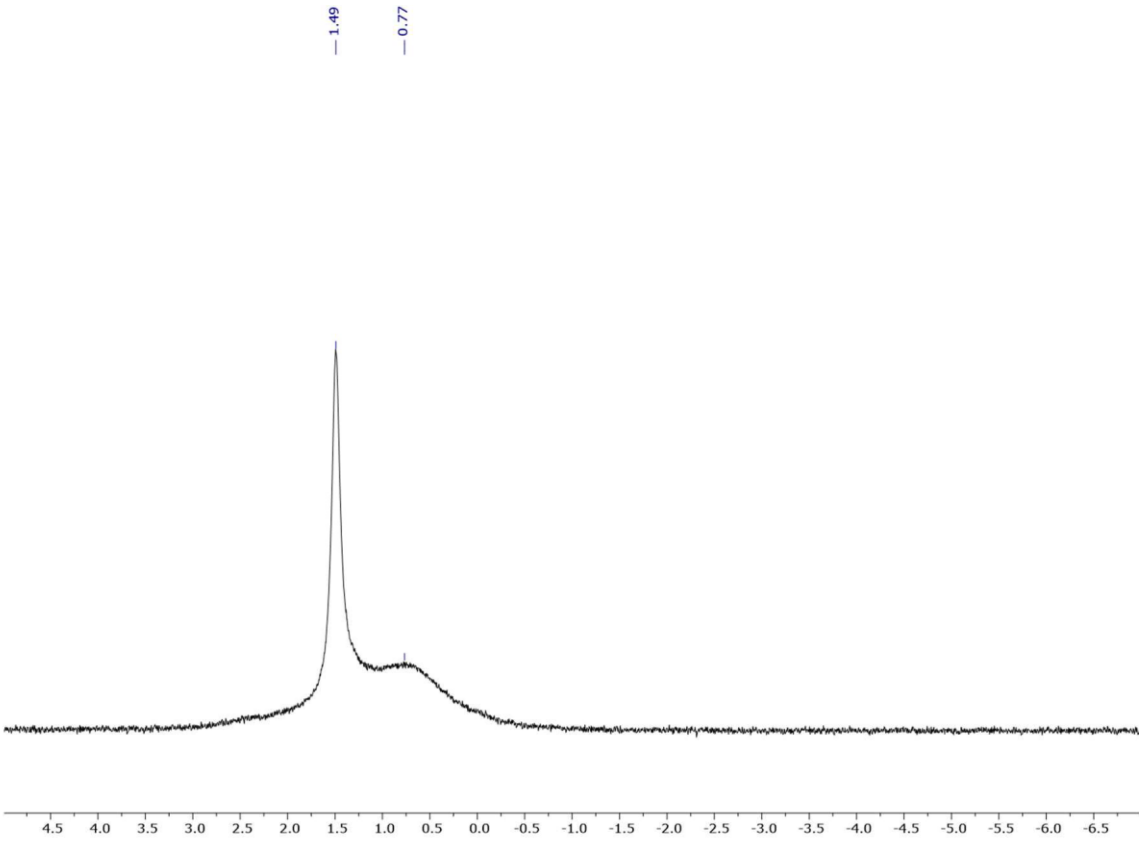

Figure S62. ^{7}Li NMR spectrum of reaction of **1** with 1 eq of HBPin in C_6D_6 at room temperature.

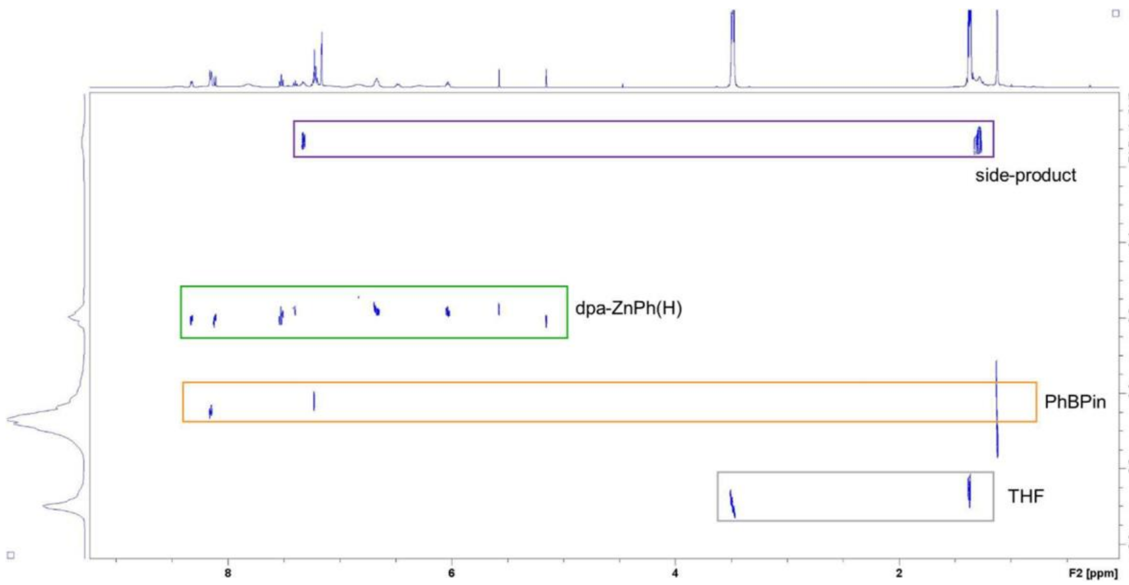

Figure S63. ^1H DOSY NMR spectrum of reaction of **1** with 1 eq of HBPin in C_6D_6 at room temperature.

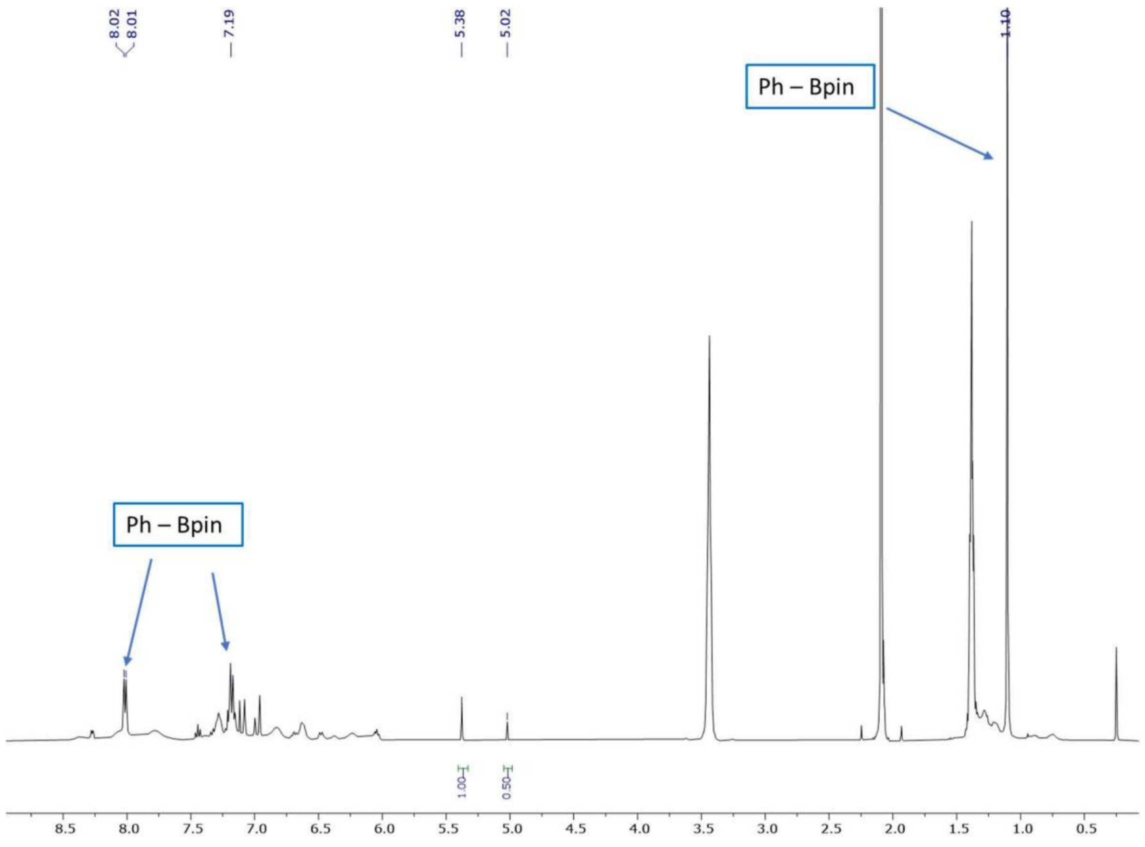

Figure S64. ^1H NMR spectrum of reaction of **1** with 1 eq of HBPin in d_8-toluene at -40 °C, recorded at room temperature.

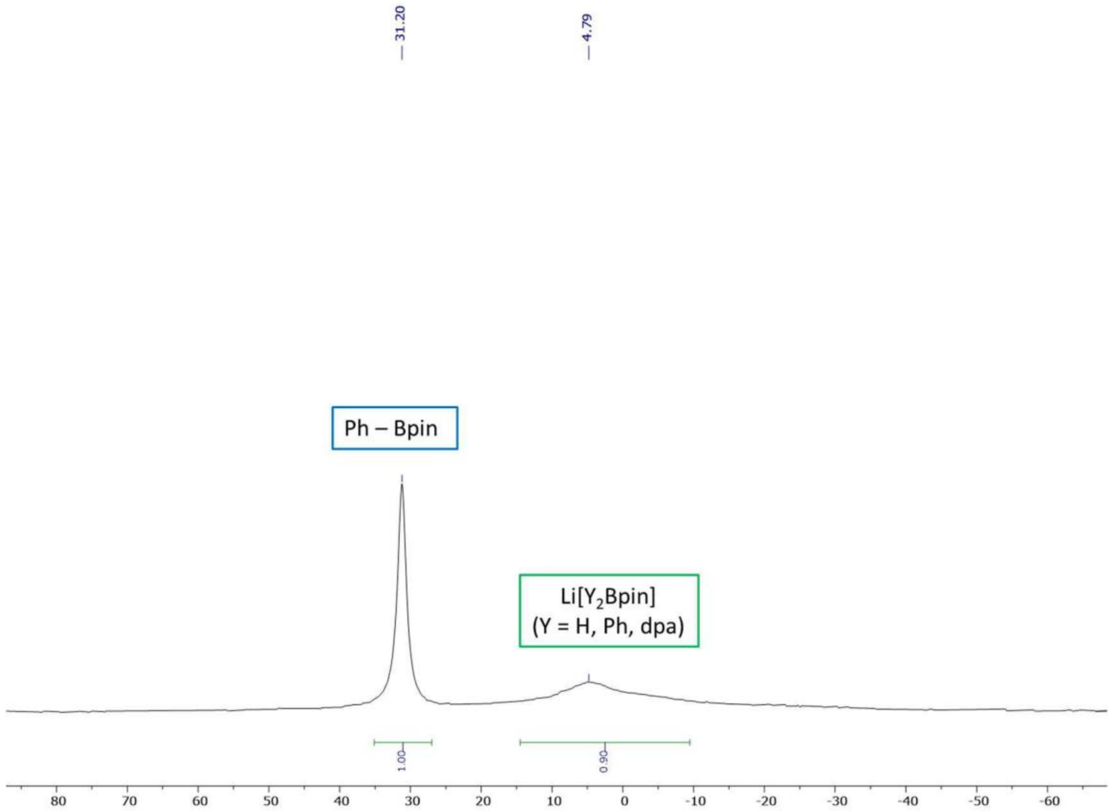

Figure S65. ^{11}B NMR spectrum of reaction of **1** with 1 eq of HBPin in d_8-toluene at -40 °C, recorded at room temperature.

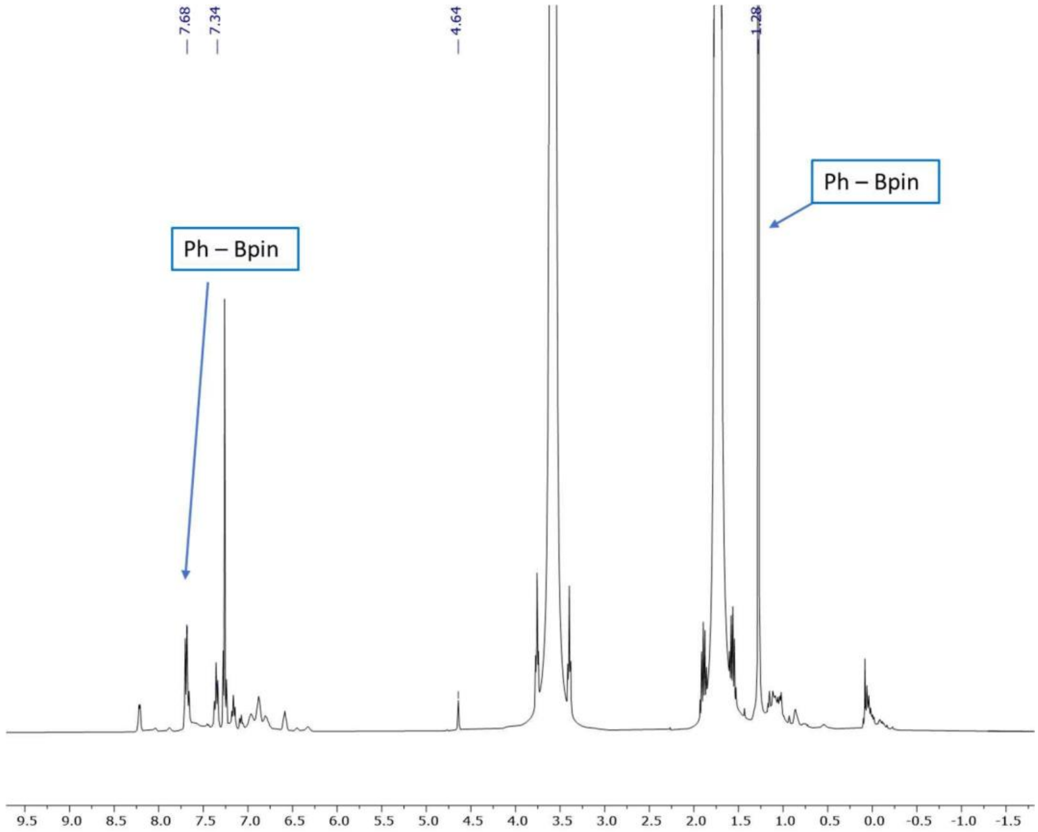

Figure S66. ^{1}H NMR spectrum of reaction of **1** with 1 eq of HBPin in THF at room temperature.

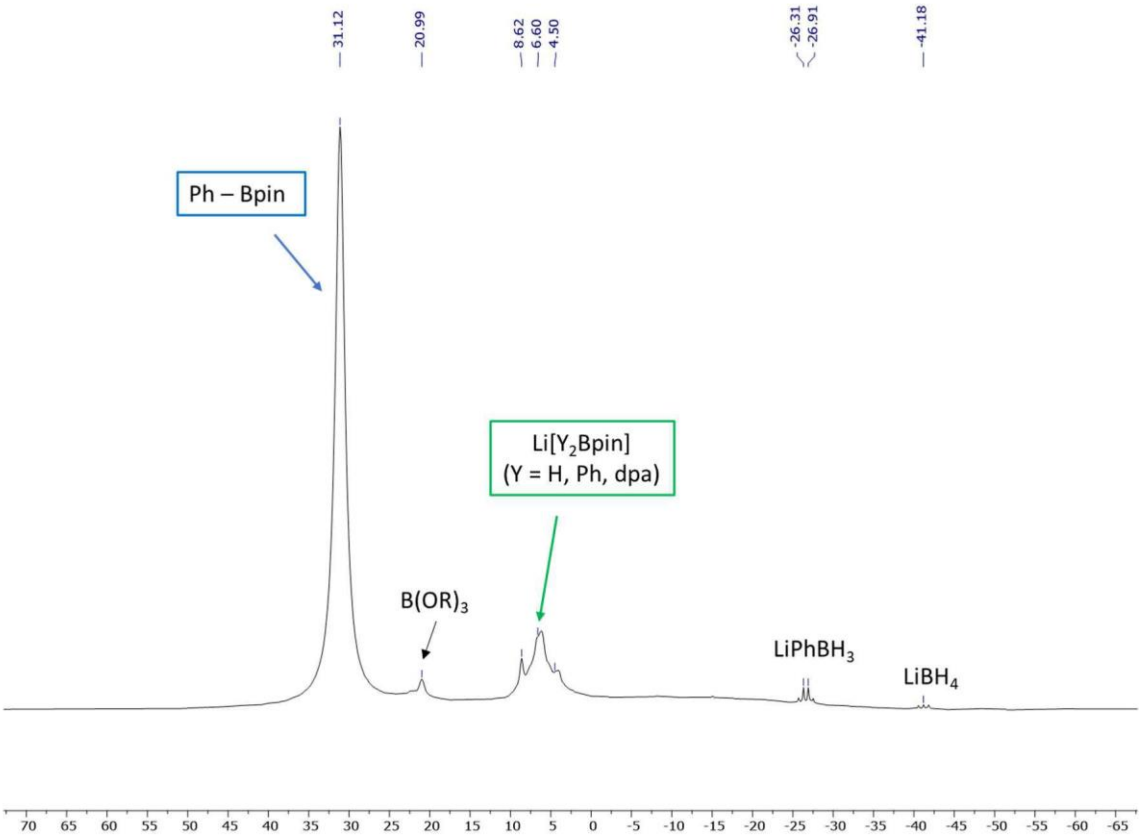

Figure S67. ^{11}B NMR spectrum of reaction of **1** with 1 eq of HBPin in THF at room temperature.

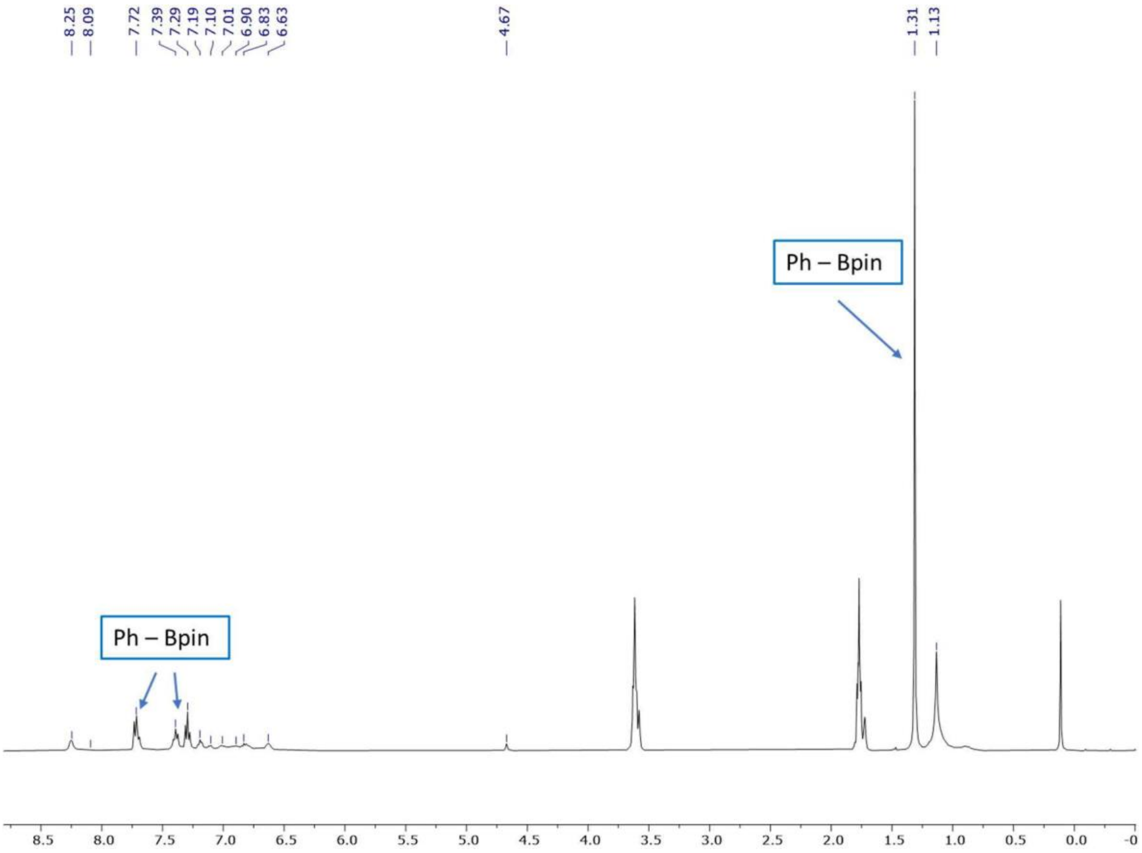

Figure S68. ^{1}H NMR spectrum of reaction of **1** with 2 eq of HBPin in d_8-THF at room temperature.

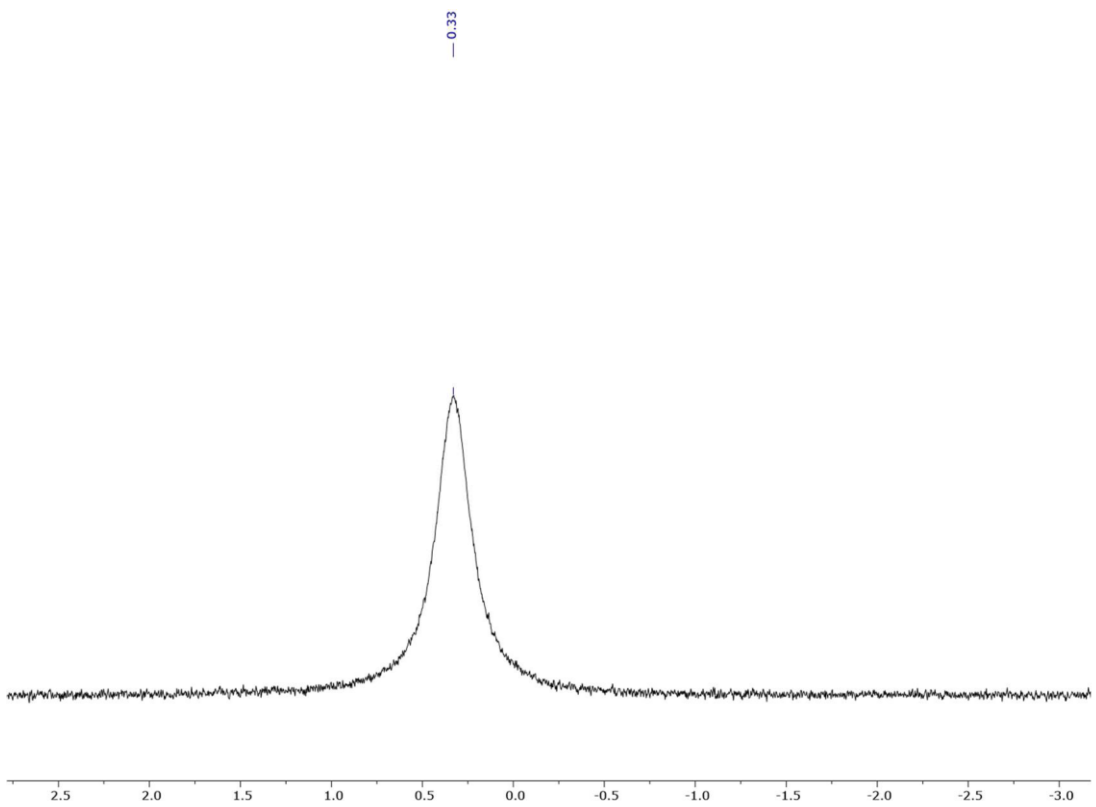

Figure S69. ^{7}Li NMR spectrum of reaction of **1** with 2 eq of HBPin in d_8-THF at room temperature.

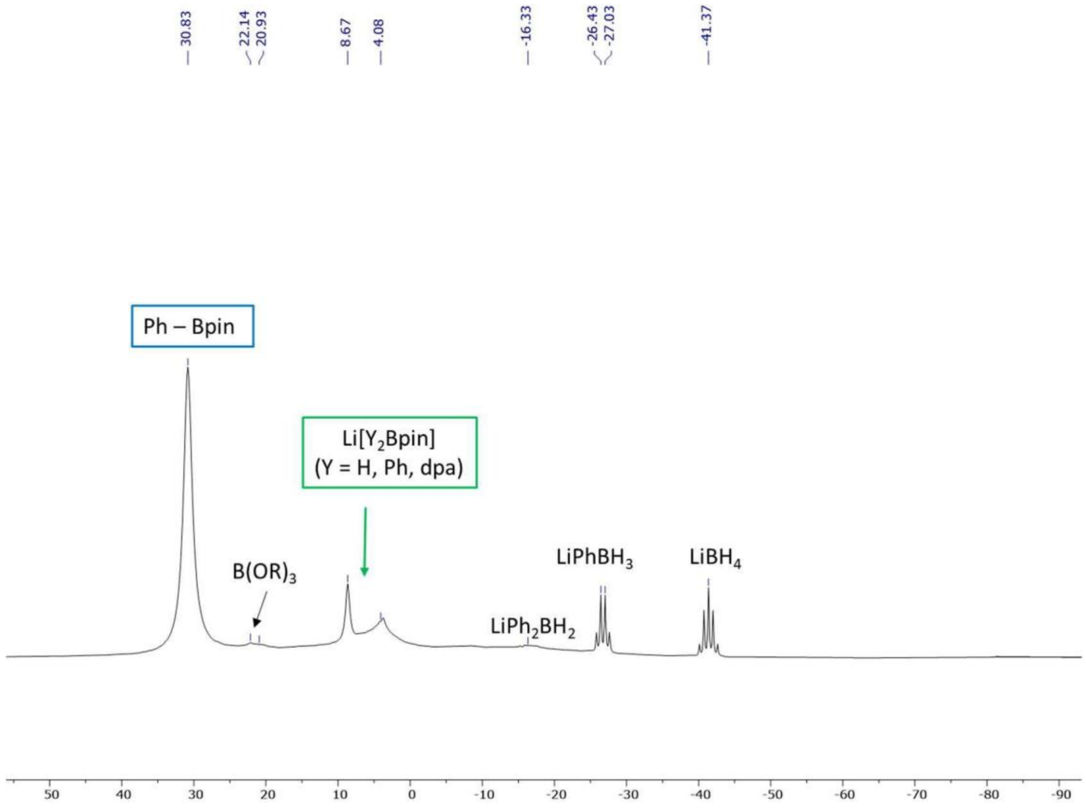

Figure S70. ^{11}B NMR spectrum of reaction of **1** with 2 eq of HBPin in d_8-THF at room temperature.

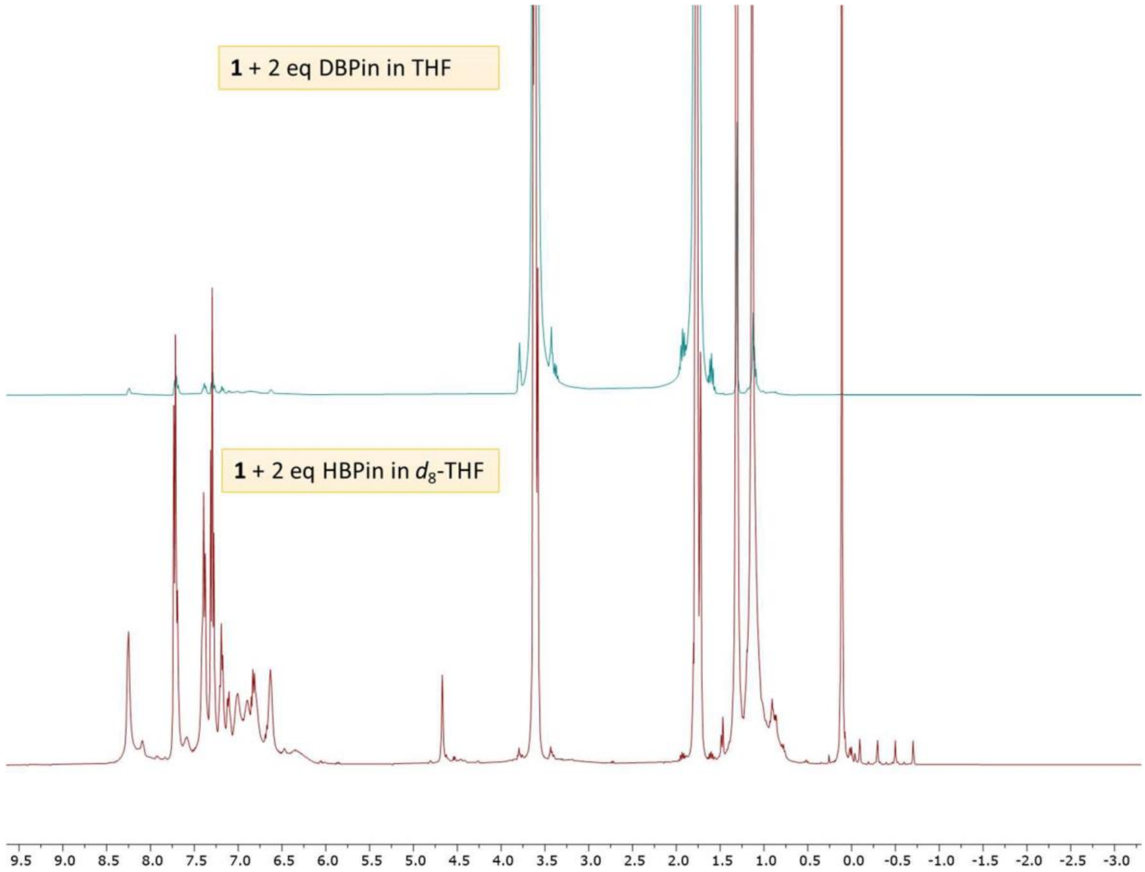

Figure S71. Stacked ^{1}H NMR spectra for comparison of reaction of **1** with 2 eq of HBPin in d_8-THF (bottom) and with 2 eq of DBPin in THF (top) at room temperature.

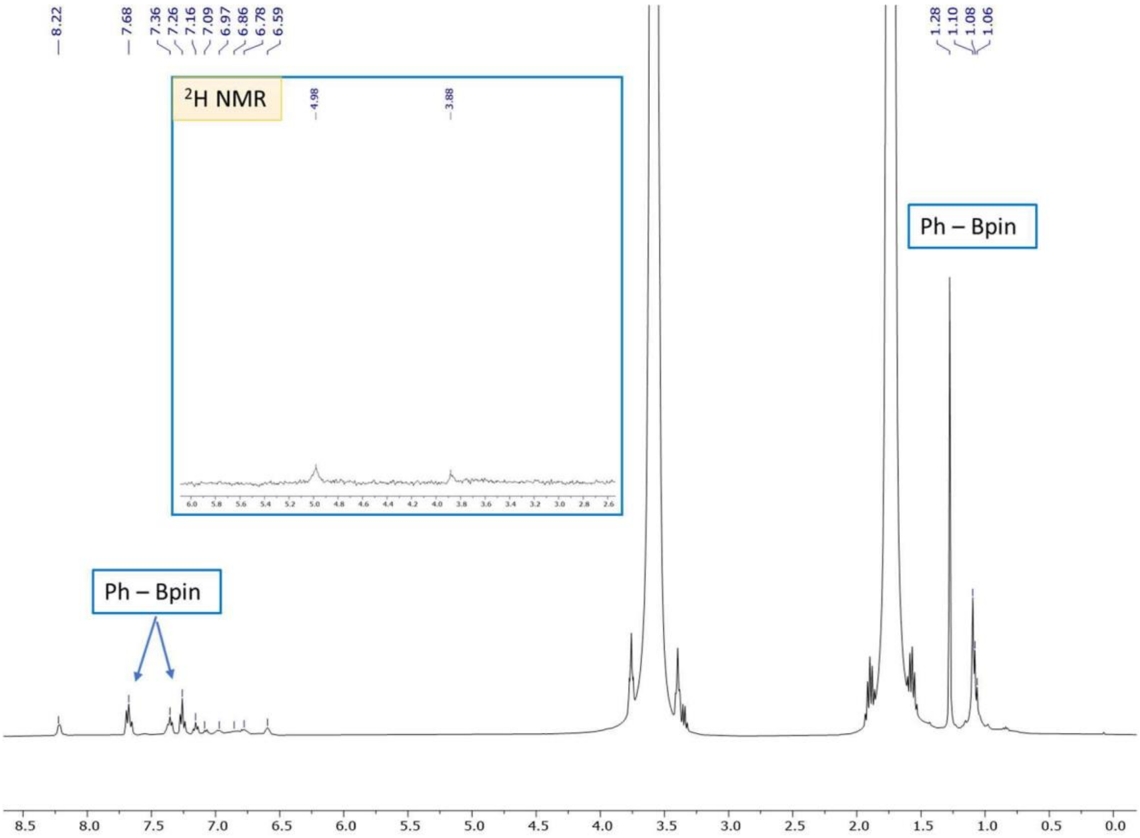

Figure S72. ^{1}H NMR spectrum of reaction of **1** with 2 eq of DBPin in d_8-THF at room temperature.

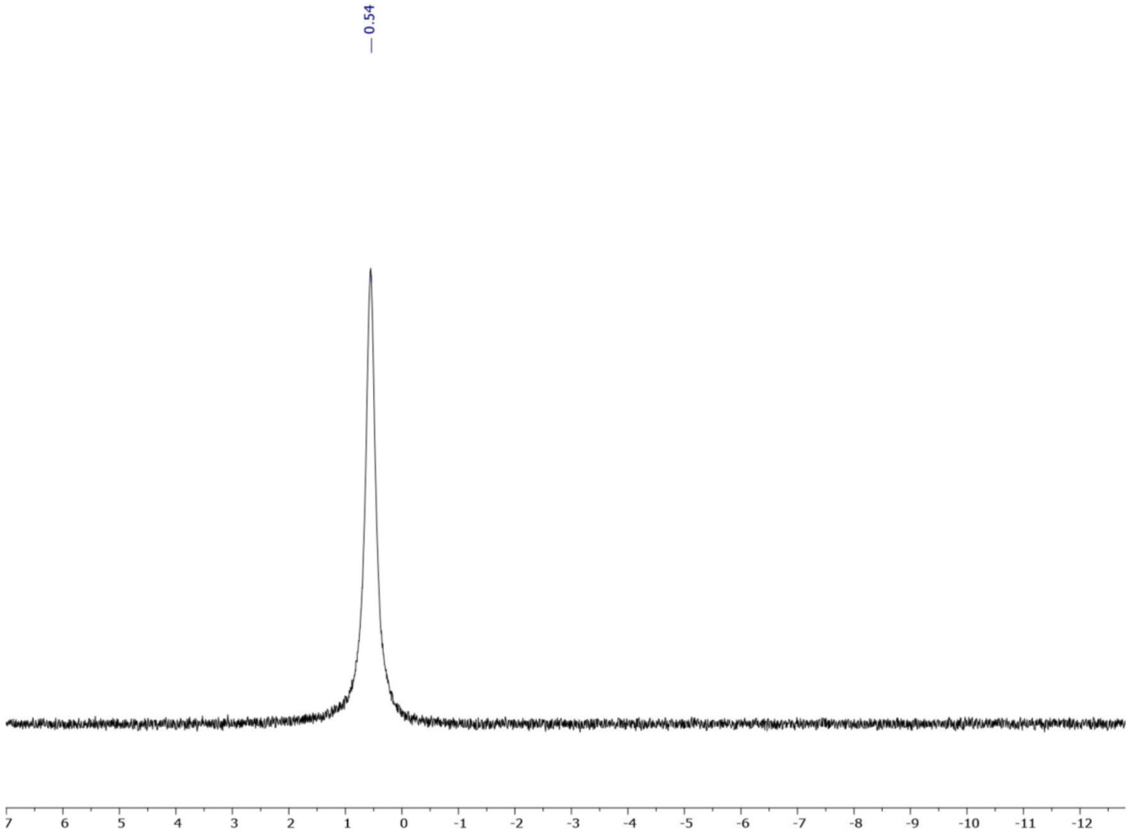

Figure S73. ^{7}Li NMR spectrum of reaction of **1** with 2 eq of DBPin in d_8-THF at room temperature.

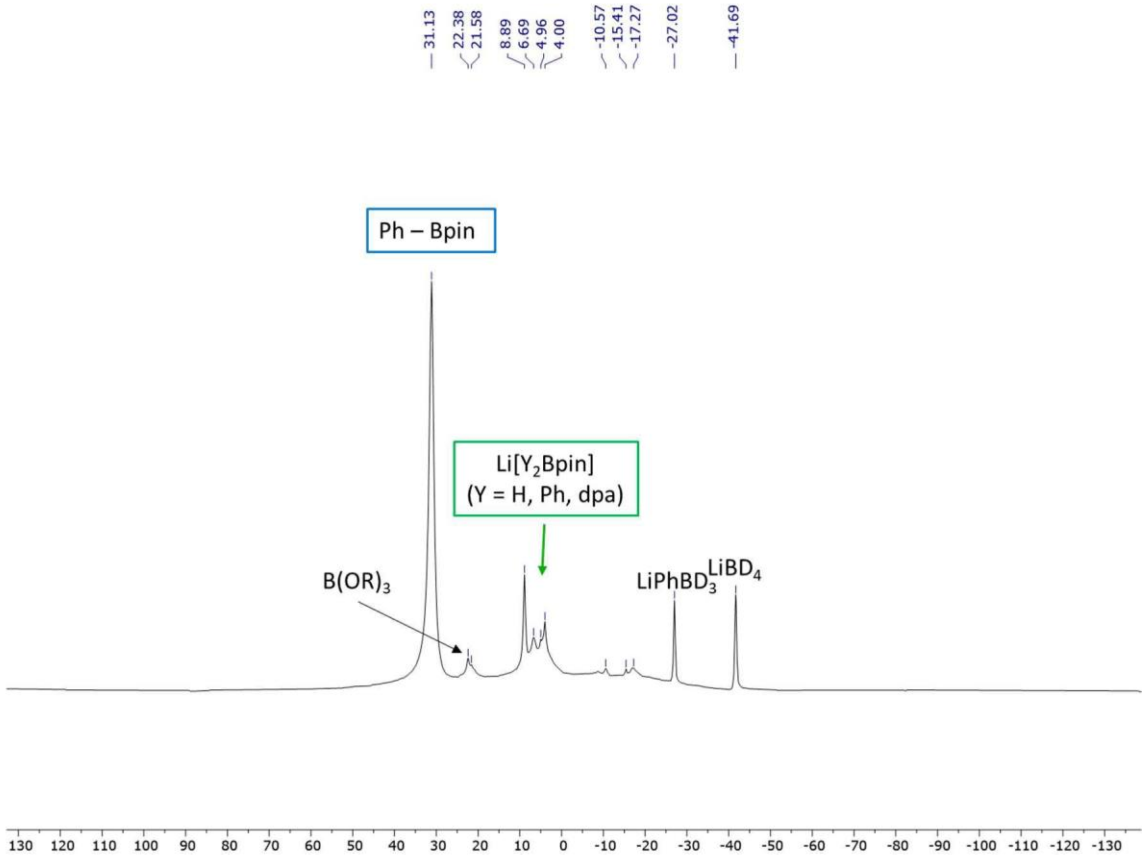

Figure S74. ^{11}B NMR spectrum of reaction of **1** with 2 eq of DBPin in d_8-THF at room temperature.

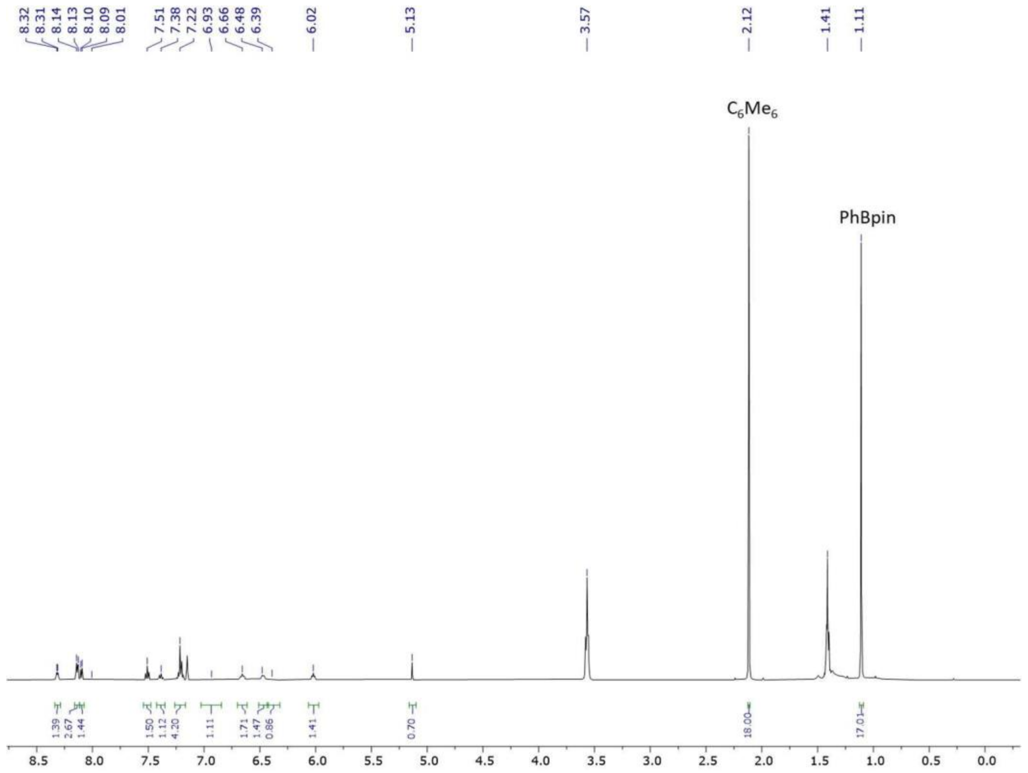

Figure S75. ^{1}H NMR spectrum of reaction of **1** with 2 eq of HBPin in C_6D_6 at room temperature with C_6Me_6 (1 eq) as internal standard showing 70% formation of PhBPin and mixed PhZn-H species.

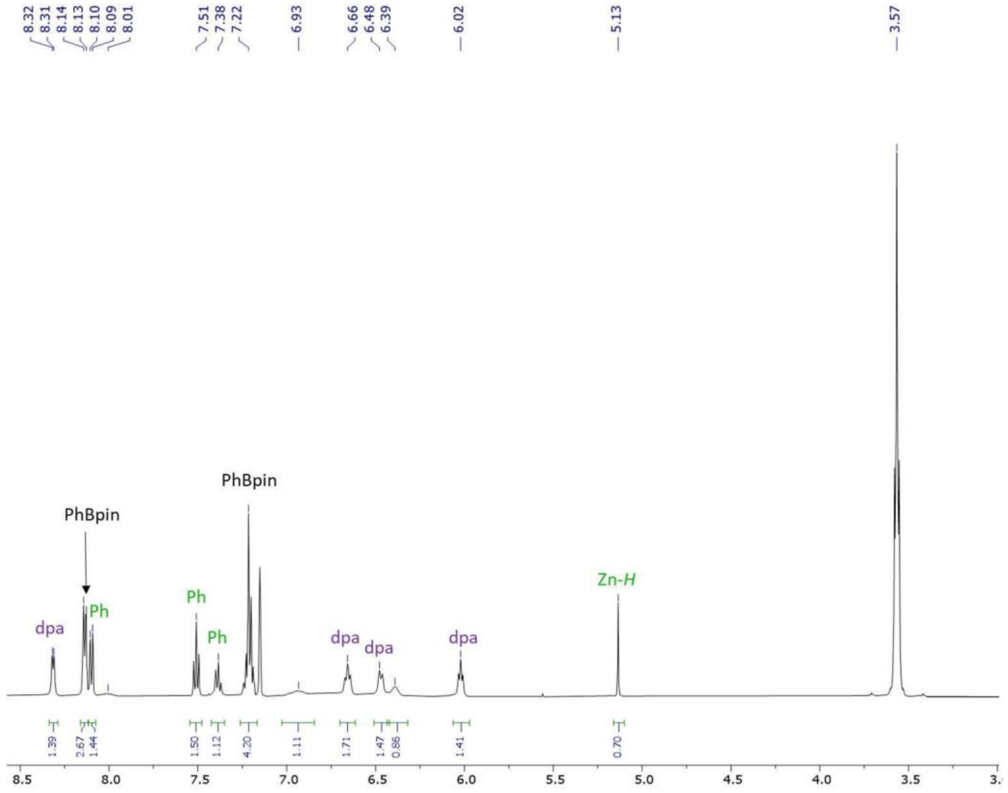

Figure S76. ^1H NMR spectrum (9-3 ppm region) of reaction of **1** with 2 eq of HBPin in C_6D_6 at room temperature with C_6Me_6 (1 eq) as internal standard showing 70% formation of PhBPin and mixed PhZn-H species.

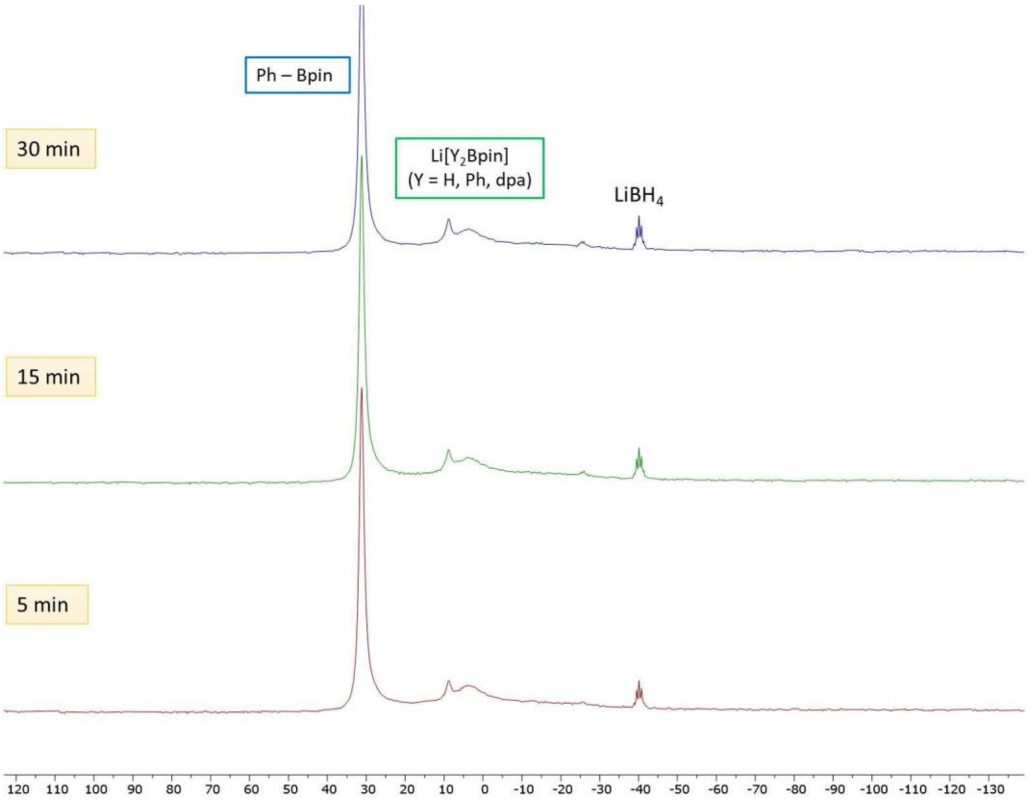

Figure S77. Stacked ^{11}B NMR spectra of reaction of **1** with 2 eq of HBPin in C_6D_6 at room temperature.

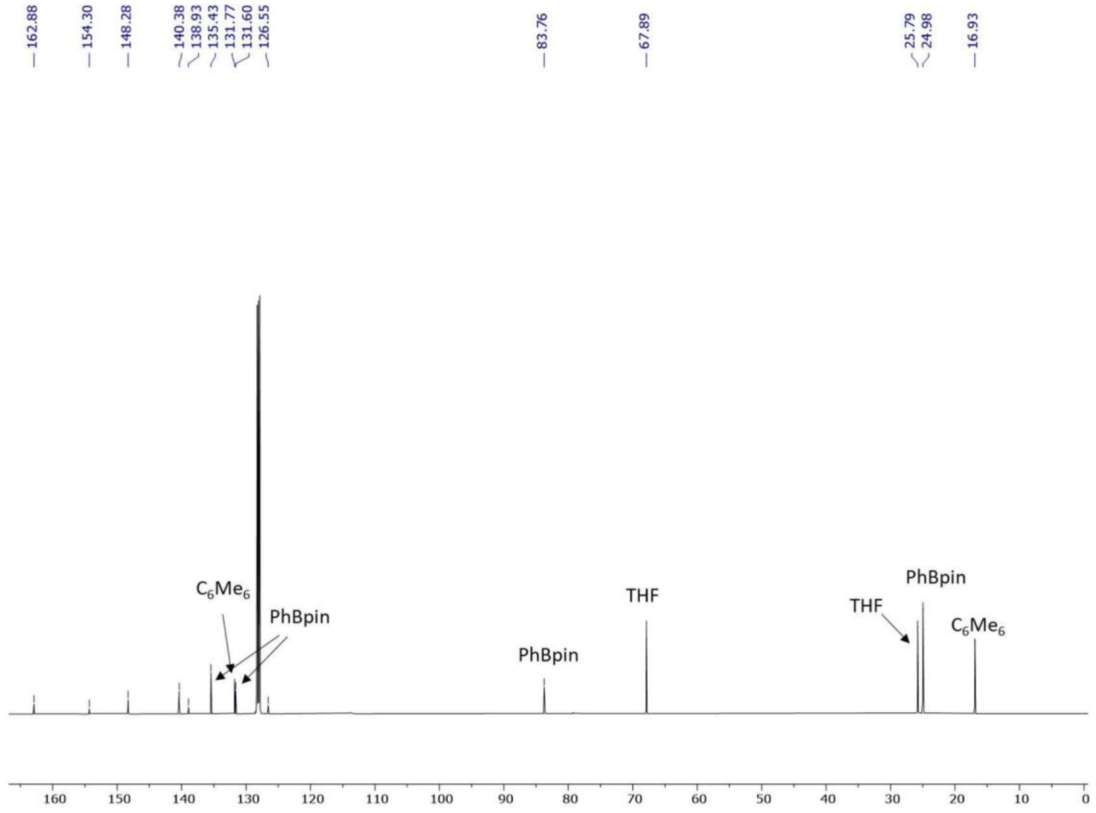

Figure S78. $^{13}C\{^1H\}$ NMR spectrum of reaction of **1** with 2 eq of HBPin in C_6D_6 at room temperature with C_6Me_6 (1 eq) as internal standard showing formation of PhBPin and mixed PhZn-H species.

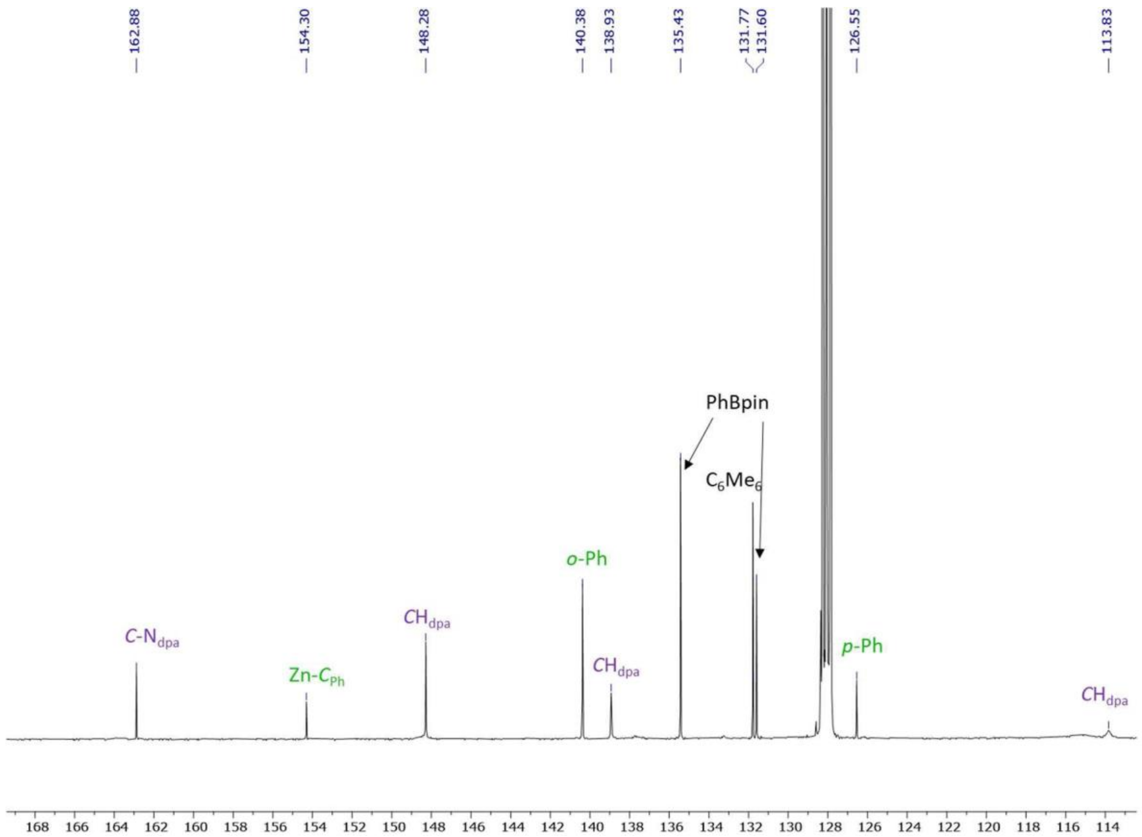

Figure S79. $^{13}C\{^1H\}$ NMR spectrum (170 -110 ppm region) of reaction of **1** with 2 eq of HBPin in C_6D_6 at room temperature with C_6Me_6 (1 eq) as internal standard showing formation of PhBPin and mixed PhZn-H species.

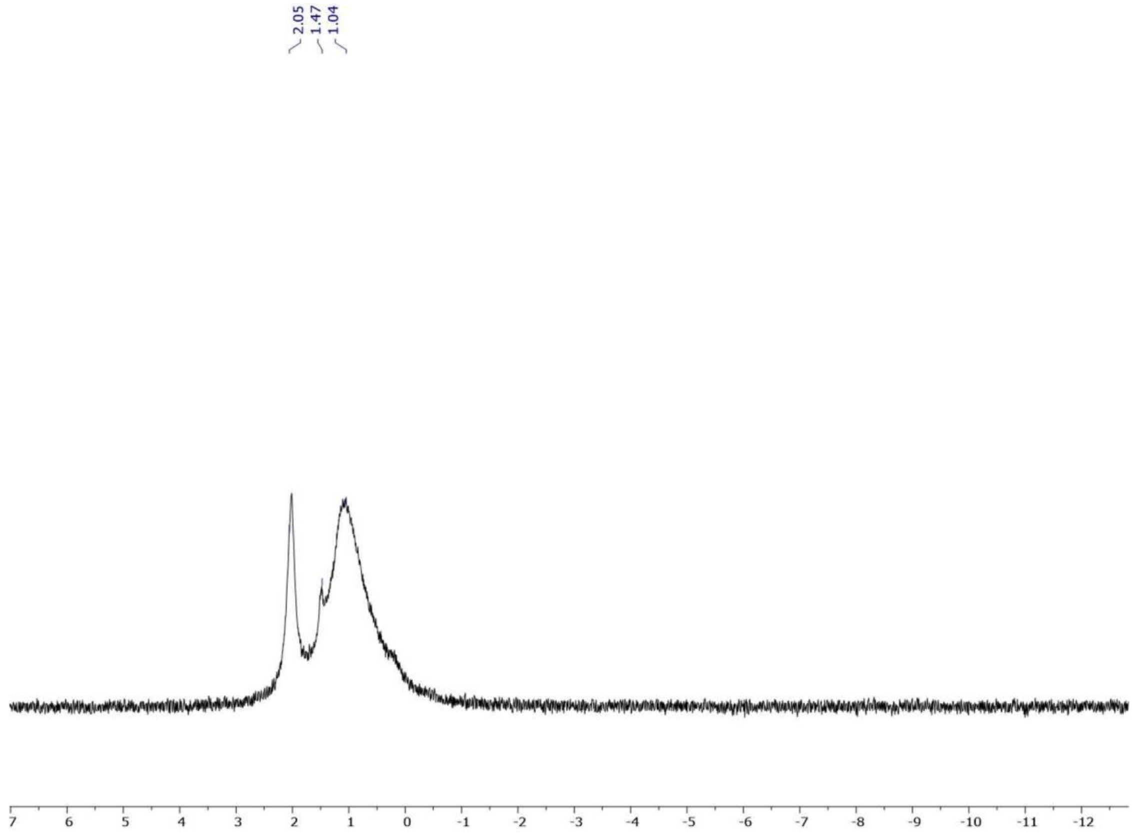

Figure S80. ^{7}Li NMR spectrum of reaction of **1** with 2 eq of HBPin in C_6D_6 at room temperature.

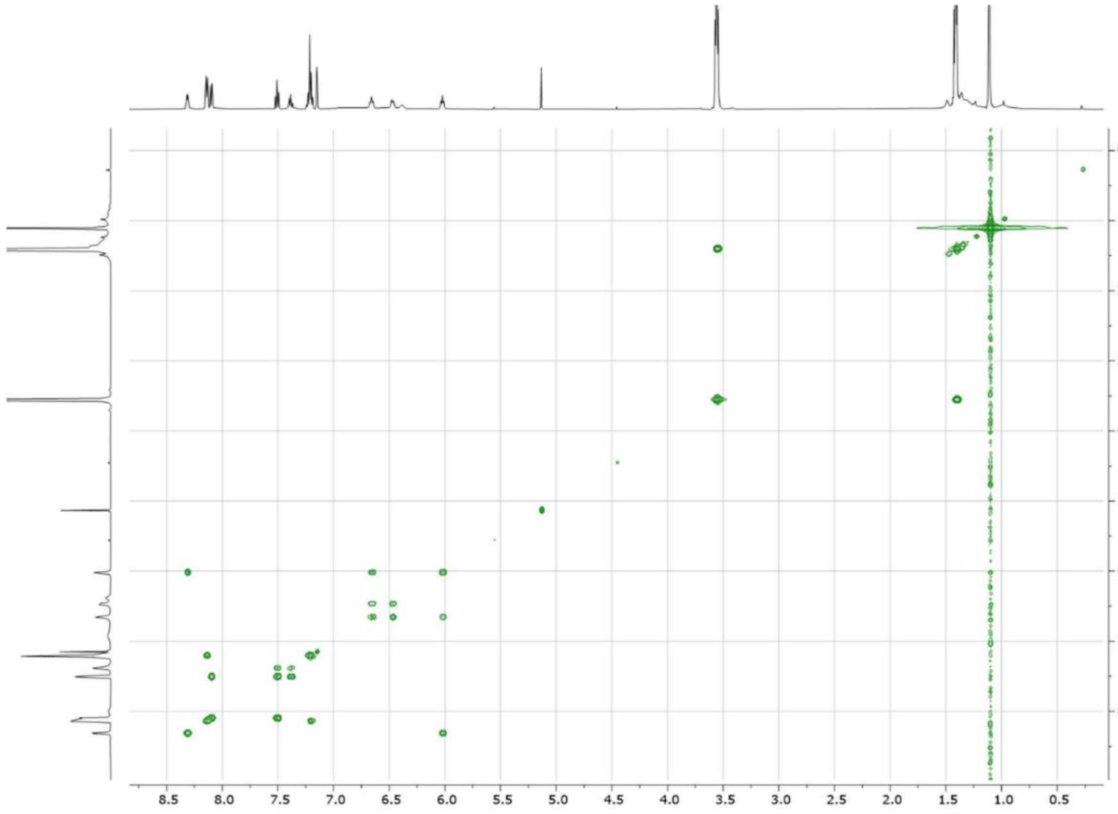

Figure 81. ^{1}H - ^{1}H COSY NMR spectrum of reaction of **1** with 2 eq of HBPin in C_6D_6 at room temperature.

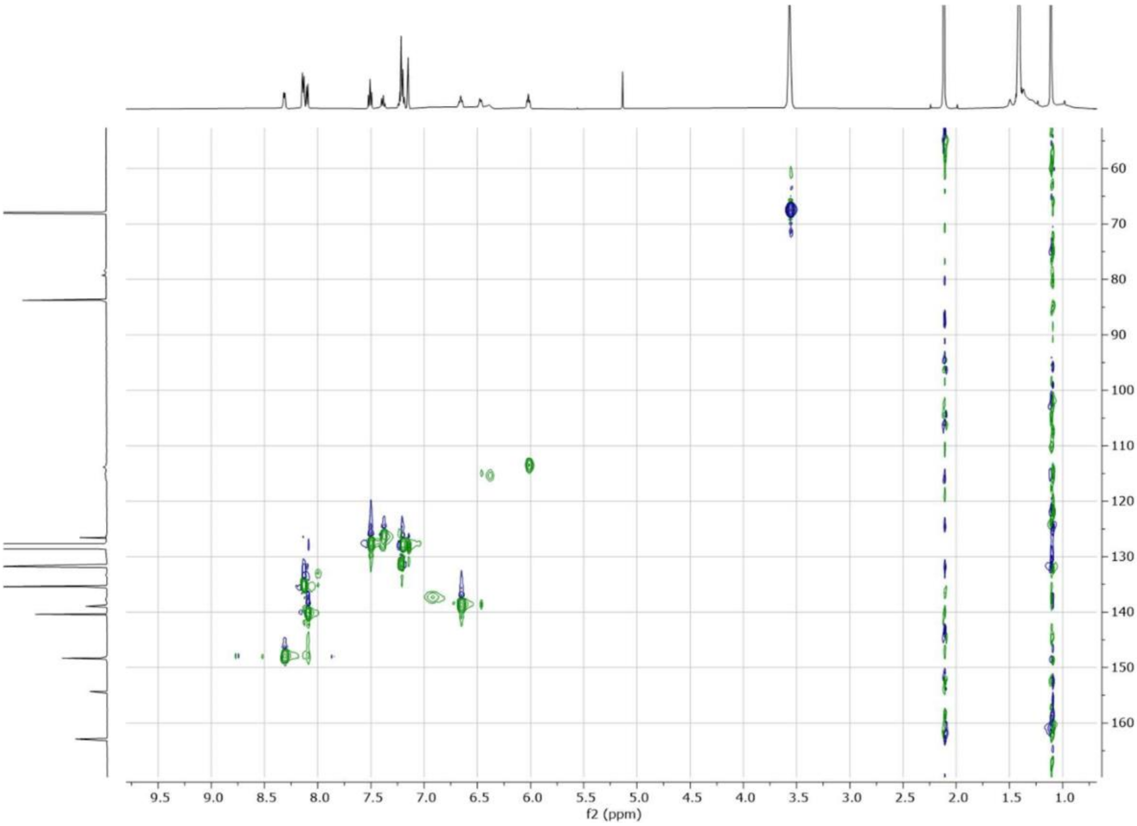

Figure S82. ^{1}H - $^{13}C\{^{1}H\}$ HSQC NMR spectrum of reaction of **1** with 2 eq of HBPin in C_6D_6 at room temperature.

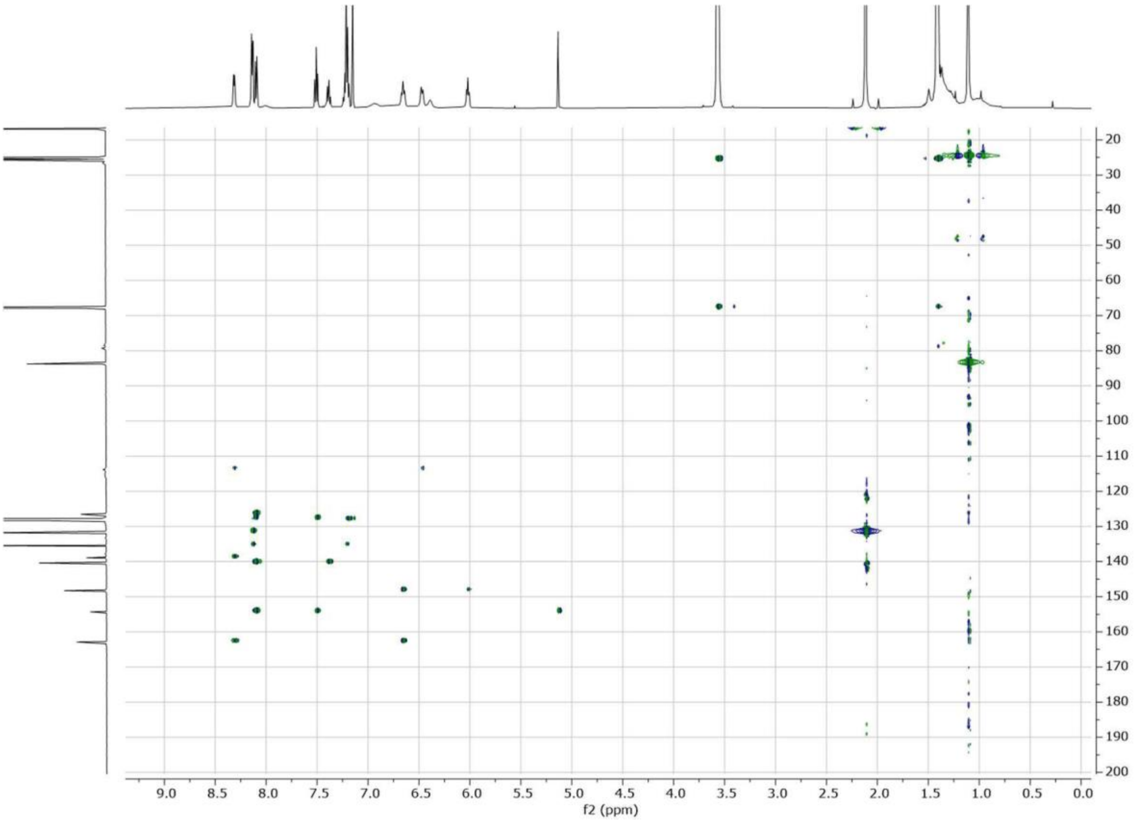

Figure S83. ^{1}H - $^{13}C\{^{1}H\}$ HMBC NMR spectrum of reaction of **1** with 2 eq of HBPin in C_6D_6 at room temperature.

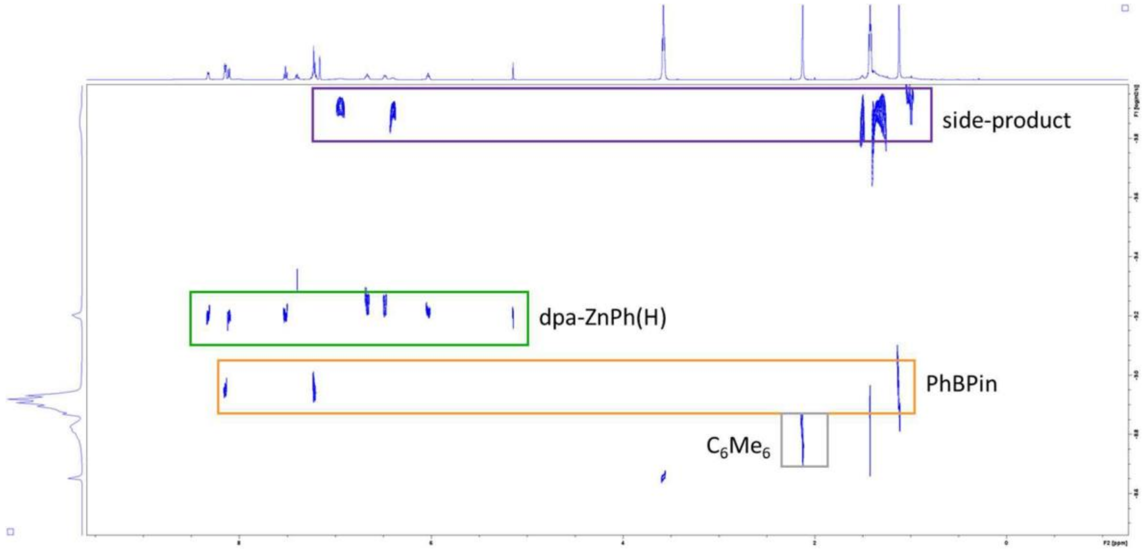

Figure S84. ^{1}H DOSY NMR spectrum of reaction of **1** with 2 eq of HBPin in C_6D_6 at room temperature.

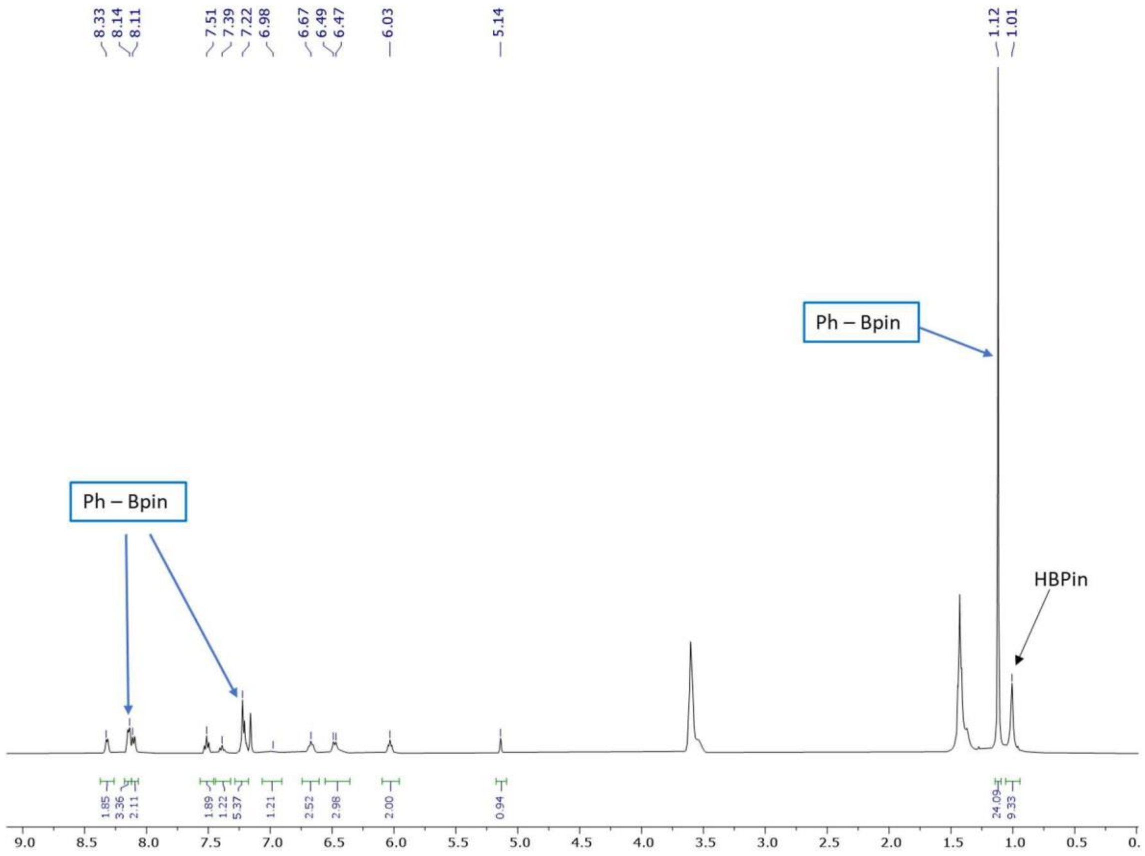

Figure S85. ^{1}H NMR spectrum of reaction of **1** with 3 eq of HBPin in C_6D_6 at room temperature.

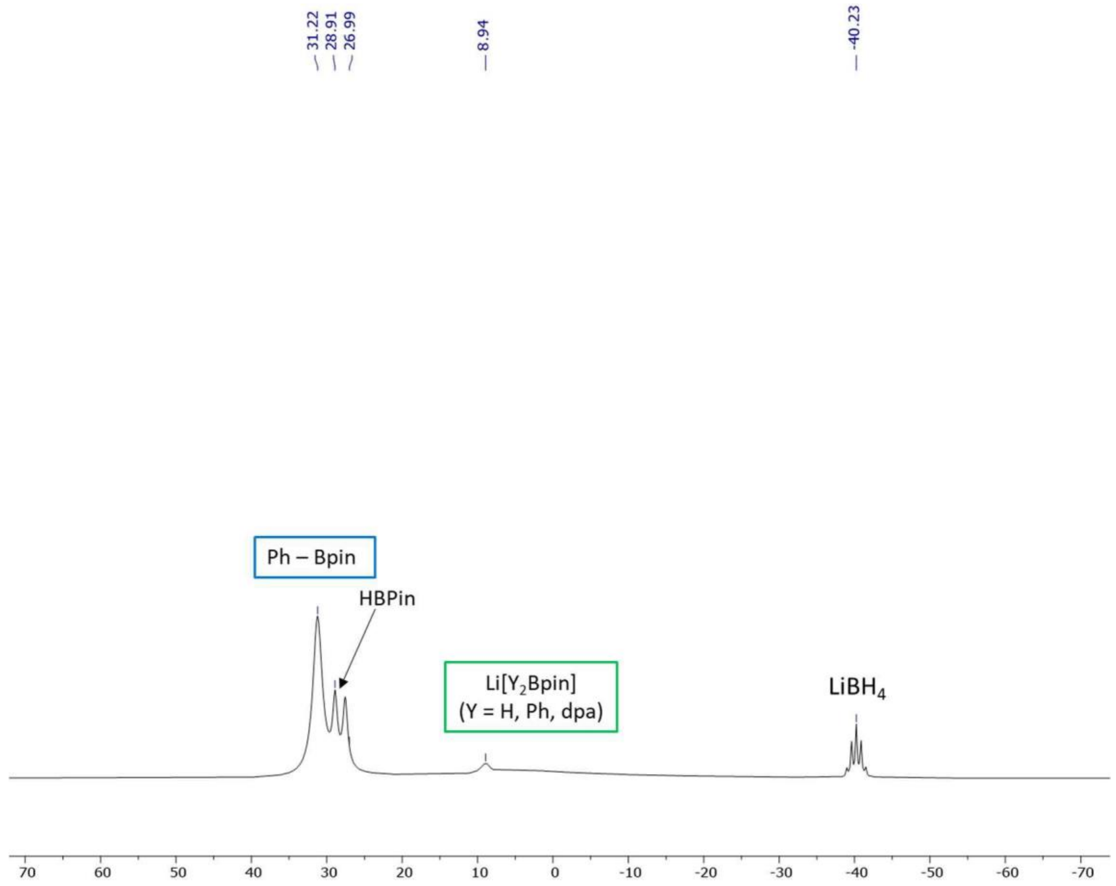

Figure S86. ^{11}B NMR spectrum of reaction of **1** with 3 eq of HBPin in C_6D_6 at room temperature.

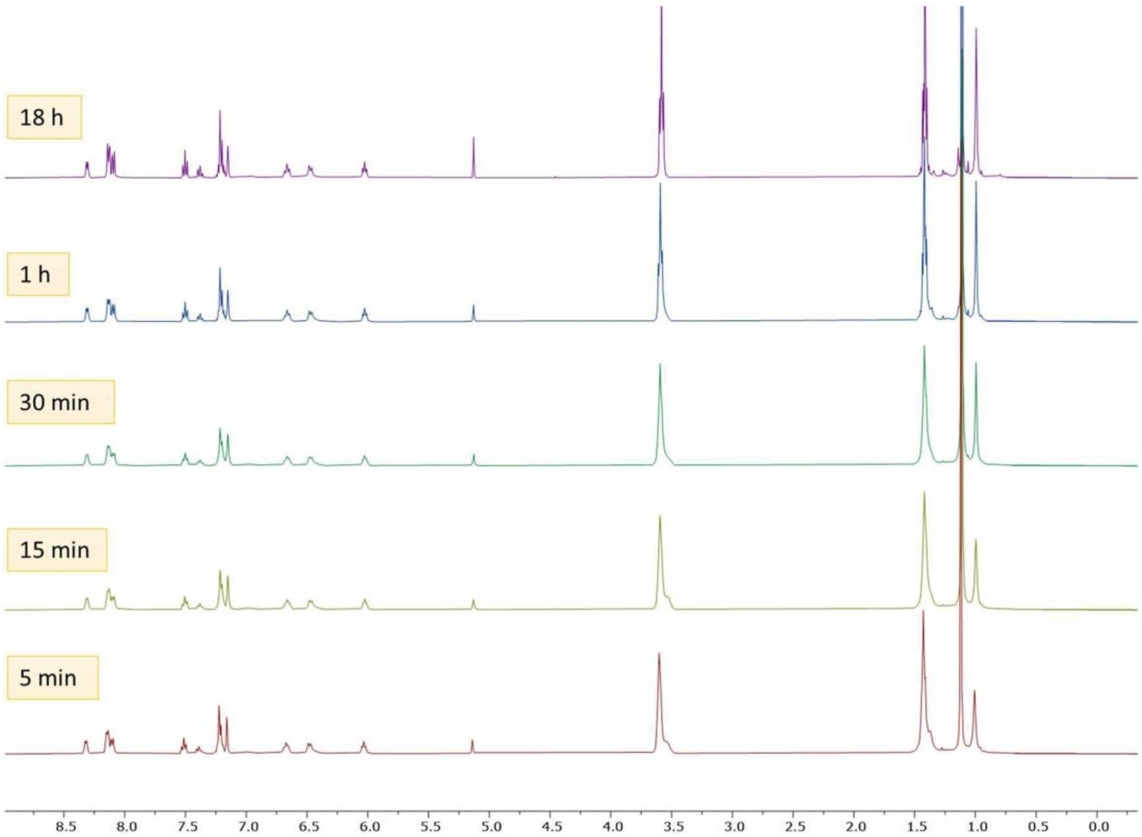

Figure S87. Stacked ^{1}H NMR spectra of reaction of **1** with 3 eq of HBPin in C_6D_6 at room temperature.

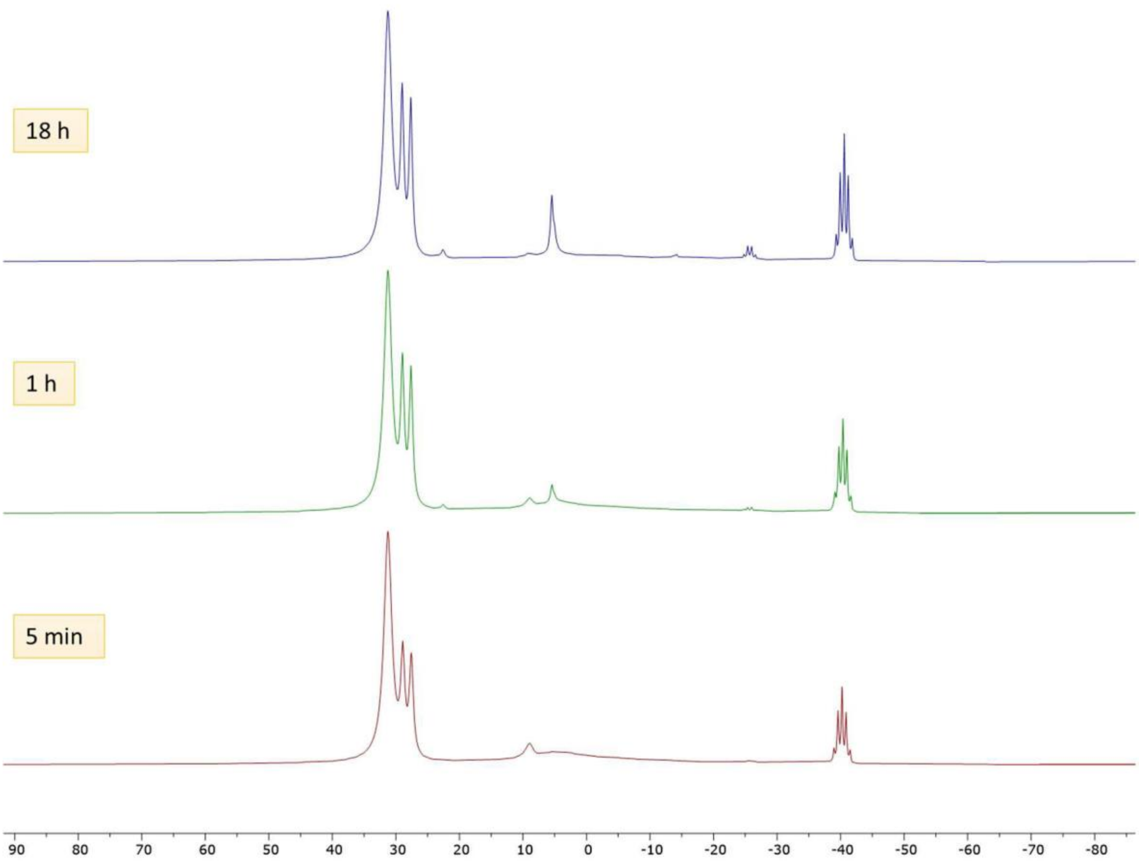

Figure S88. Stacked ^{11}B NMR spectra of reaction of **1** with 3 eq of HBPin in C_6D_6 at room temperature.

5.2 Reactivity of 1 towards HBCat

A J Young's NMR tube was charged with **1** (30 mg, 4.89 x 10^{-5} mol, 1 eq) followed by the addition of 0.5 mL of C_6D_6 and HBcat (5.2 µl, 4.89 x 10^{-5} mol, 1 eq). The sealed tube was spun at room temperature and after an hour analysed by ^{1}H and ^{11}B NMR spectroscopy (Figure S89-S90) clearly showing extensive decomposition and/or ligand redistribution, with only trace amounts of targeted Zn-H and Ph-BCat species observable.

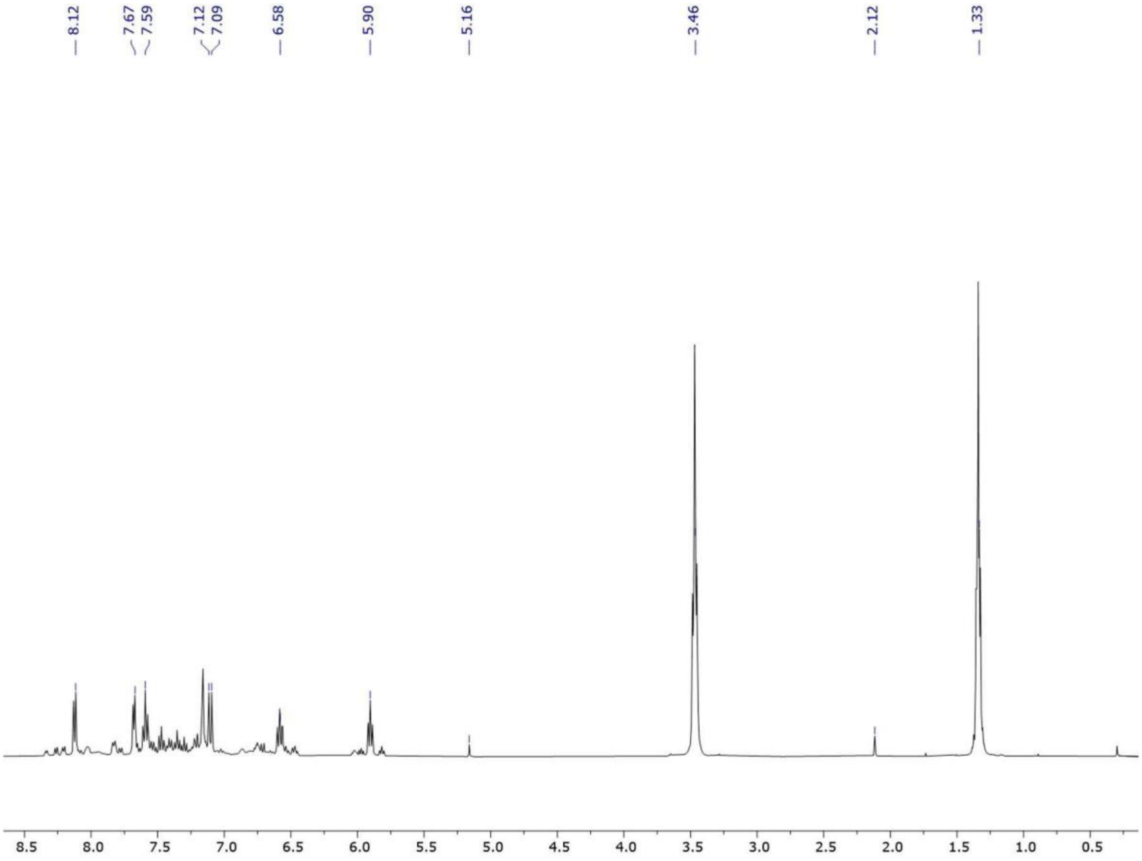

Figure S89. ^{1}H NMR spectrum of reaction of **1** with 1 eq of HBCat in C_6D_6 at room temperature.

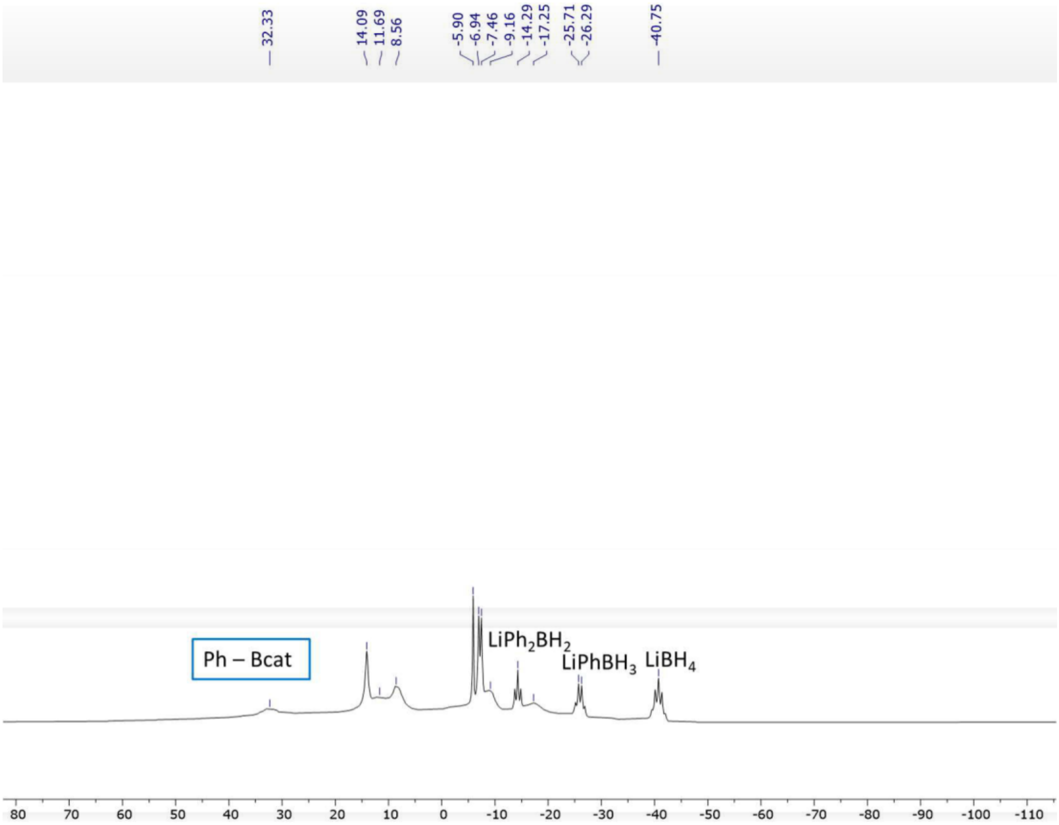

Figure S90. ^{11}B NMR spectrum of reaction of **1** with 1 eq of HBCat in C_6D_6 at room temperature.

5.3 Reactivity of 1 towards silane

A J Young's NMR tube was charged with **1** (34 mg, 5.55 x 10^{-5} mol, 1 eq) followed by the addition of 0.5 mL of C_6D_6 and Ph_3SiOH (15.3 mg, 5.55 x 10^{-5} mol, 1 eq). The sealed tube was spun at room temperature and analysed by ^1H and ^7Li NMR spectroscopy after one hour revealing complete consumption of starting materials and formation of benzene (Figure S91-S92). To this in situ formed alkoxide species, $PhSiH_3$ (7 µL, 5.55 x 10^{-5} mol, 1 eq) was added and analysed by ^1H and ^7Li NMR spectroscopy (Figure S93-S94) after one hour and 3 days later. In both cases only traces of Zn-H species are evident and majority of silane is unreacted. With prolonged reaction times (i.e. 3 days) only increased degradation is evident, rather than formation of desired product.

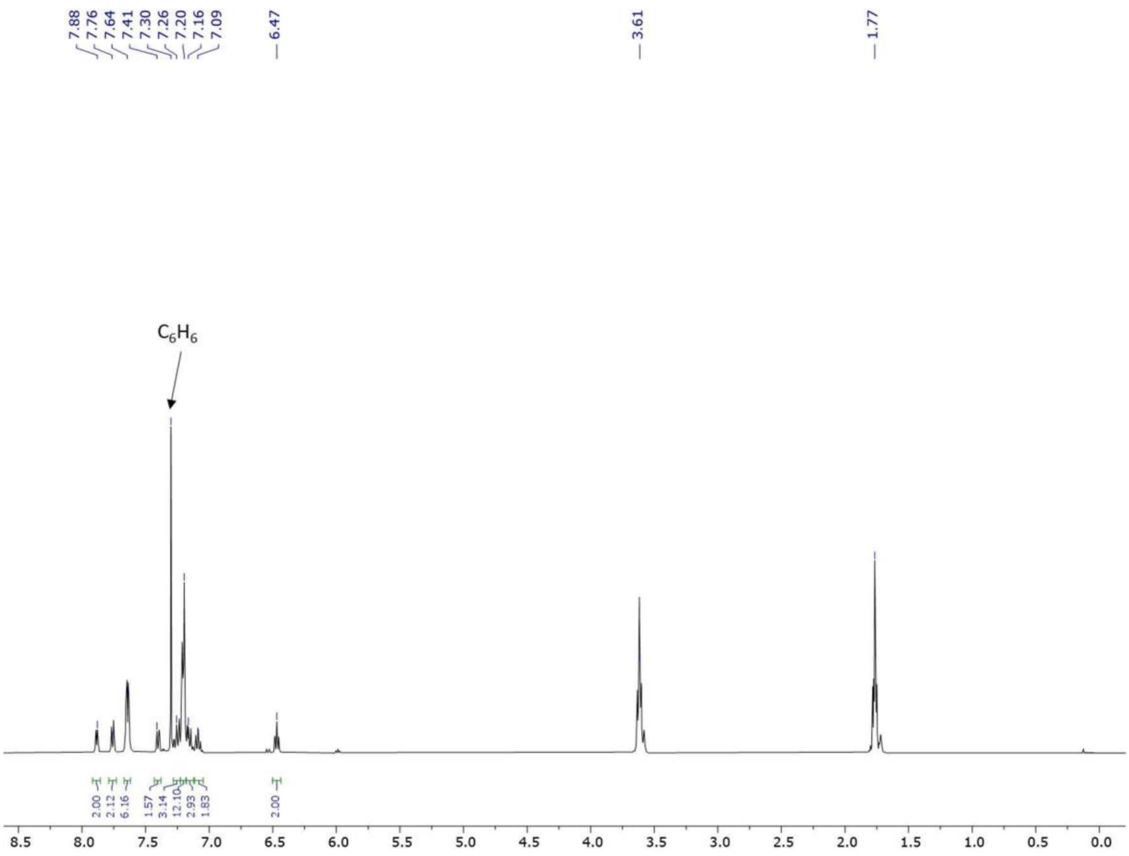

Figure S91. ^1H NMR spectrum of reaction of **1** with 1 eq of Ph_3SiOH in d_8-THF at room temperature.

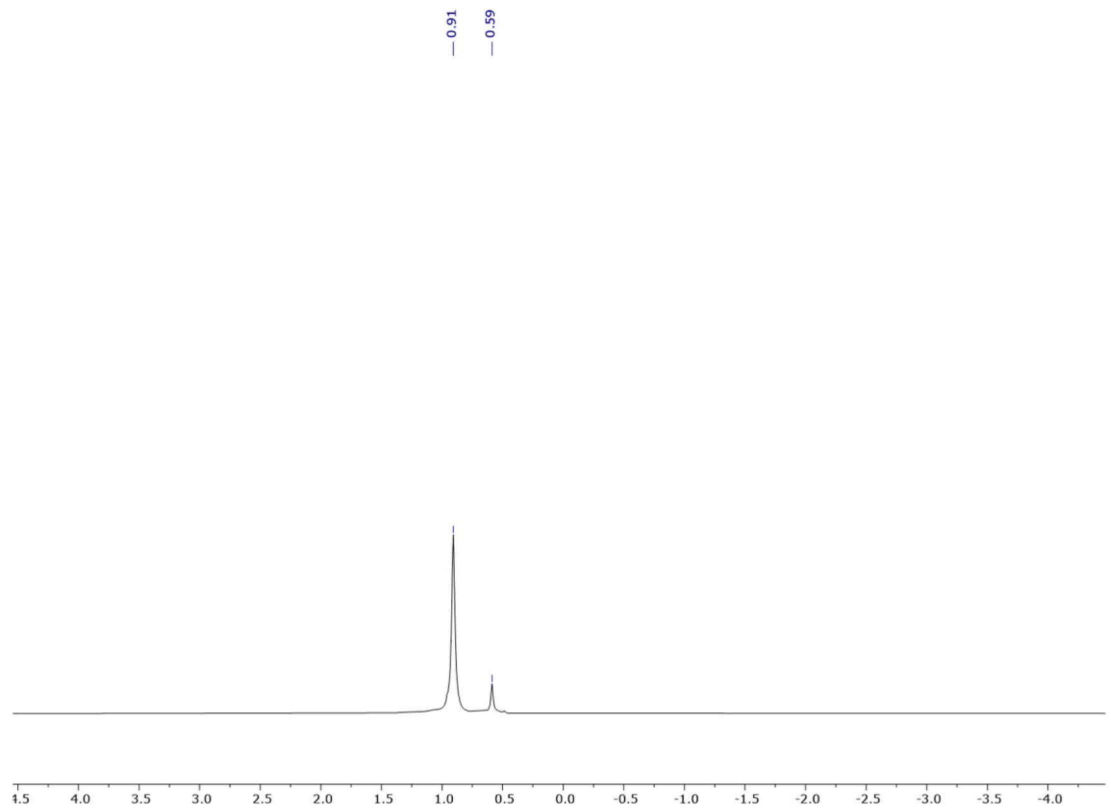

Figure S92. ^{7}Li NMR spectrum of reaction of **1** with 1 eq of Ph_3SiOH in d_8-THF at room temperature.

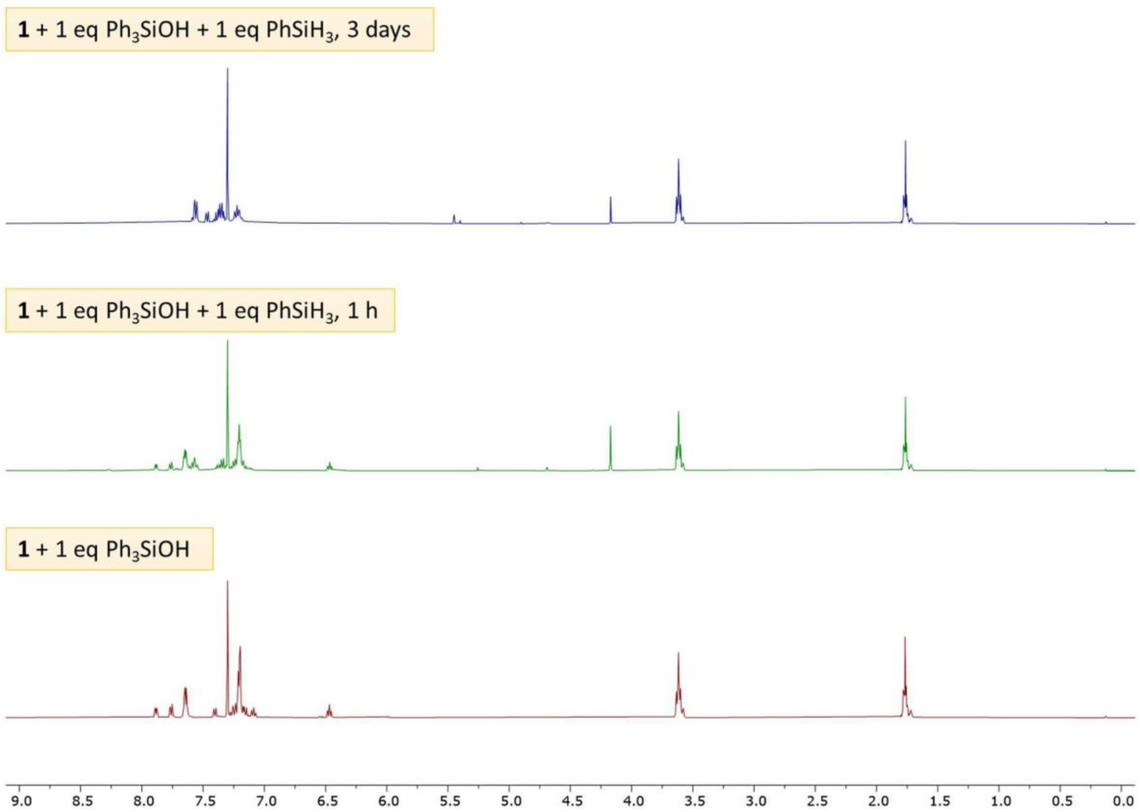

Figure S93. Stacked ^{1}H NMR spectra of reaction of **1** with 1 eq of Ph_3SiOH, followed by reaction with 1 eq of $PhSiH_3$ in d_8-THF at room temperature.

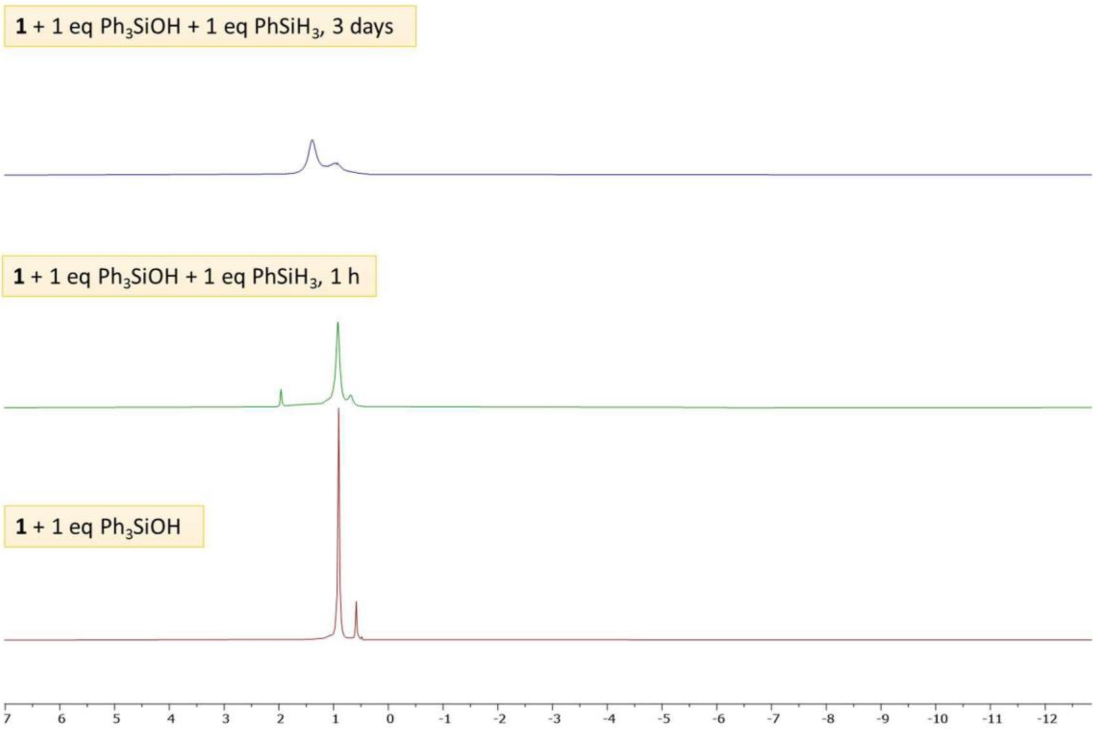

Figure S94. Stacked ^{7}Li NMR spectra of reaction of **1** with 1 eq of Ph_3SiOH, followed by reaction with 1 eq of $PhSiH_3$ in d_8-THF at room temperature.

5.4 Reactivity of 3 towards HBPin

A J Young's NMR tube was charged with **3** (30 mg, 5.26 x 10^{-5} mol, 1 eq) followed by the addition of 0.5 mL of C_6D_6 and HBpin (15.3 µl, 1.05 x 10^{-5} mol, 2 eq). The sealed tube was spun at room temperature and analysed by ^{1}H and ^{11}B NMR spectroscopy (Figure S95-S96) after 90 min revealing that majority of HBPin had not reacted. The reaction mixture was left over night to react (18 h) and again analysed. Both ^{1}H and ^{11}B NMR spectra now show almost complete consumption of HBPin (Figure S97-S98) and formation of the same Zn-H species **7B** as when lithium zincate **1** is used, albeit it is formed at a much slower rate (Figure S99-S100).

Control reaction between PMDETA and HBPin was finally performed by mixing the two reagents in C_6D_6 in a sealed J Young's NMR tube. ^{1}H and ^{11}B NMR spectroscopy (Figure S101-S102) showed no interaction between these reagents.

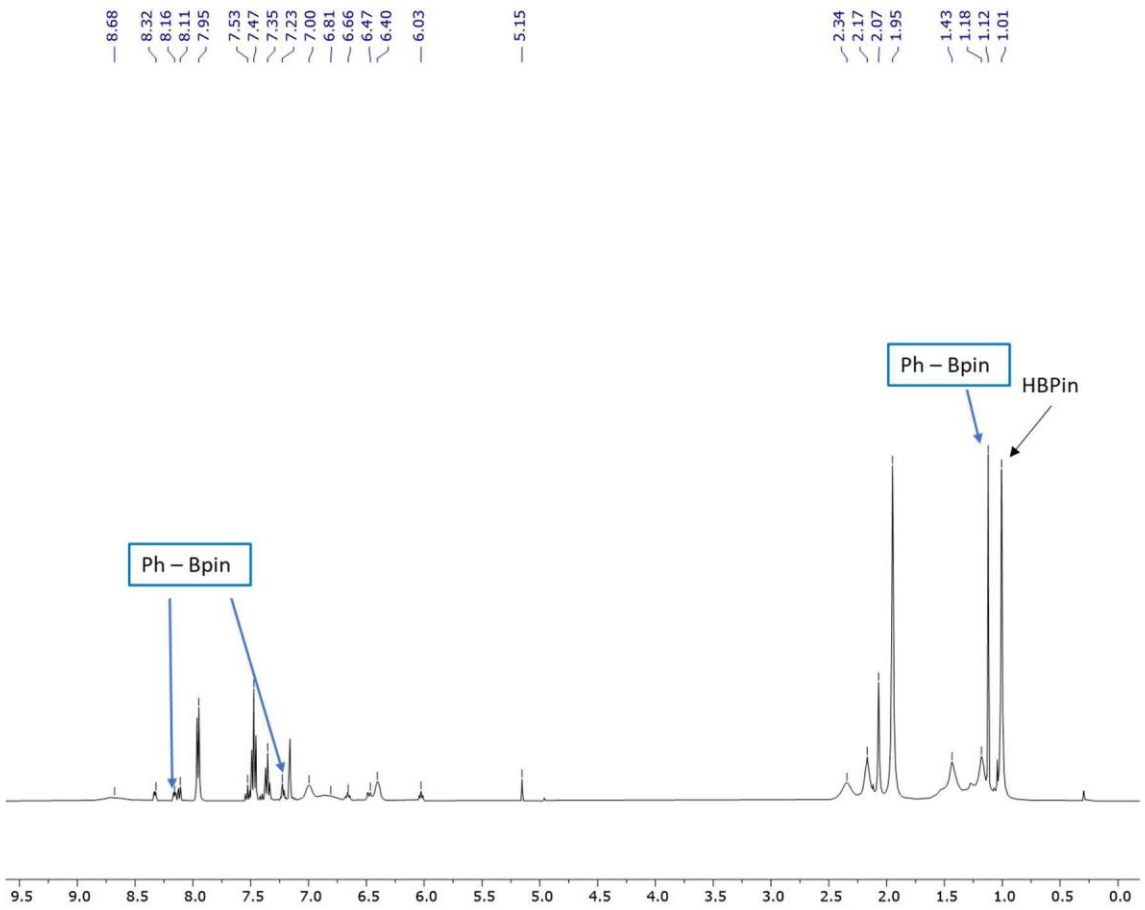

Figure S95. ^1H NMR spectrum of reaction of **3** with 2 eq of HBPin in C_6D_6 at room temperature after 90 min.

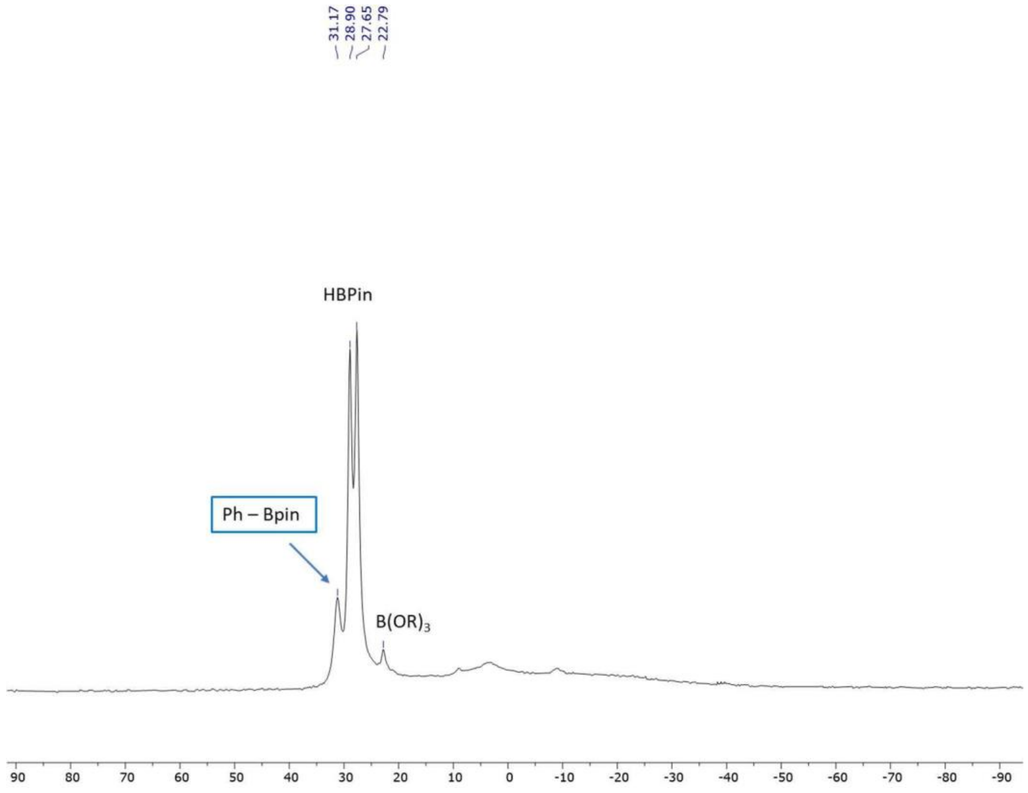

Figure S96. ^{11}B NMR spectrum of reaction of **3** with 2 eq of HBPin in C_6D_6 at room temperature after 90 min.

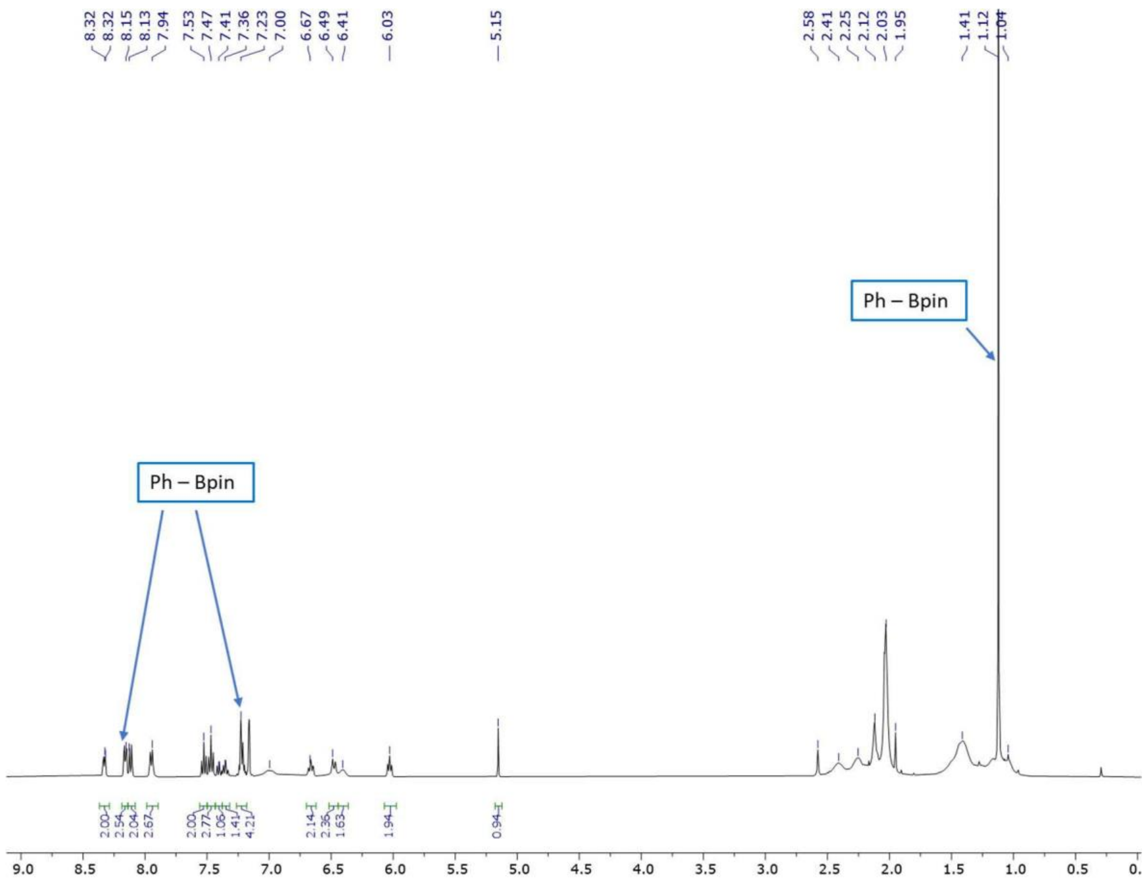

Figure S97. ^{1}H NMR spectrum of reaction of **3** with 2 eq of HBPin in C_6D_6 at room temperature after 18 h.

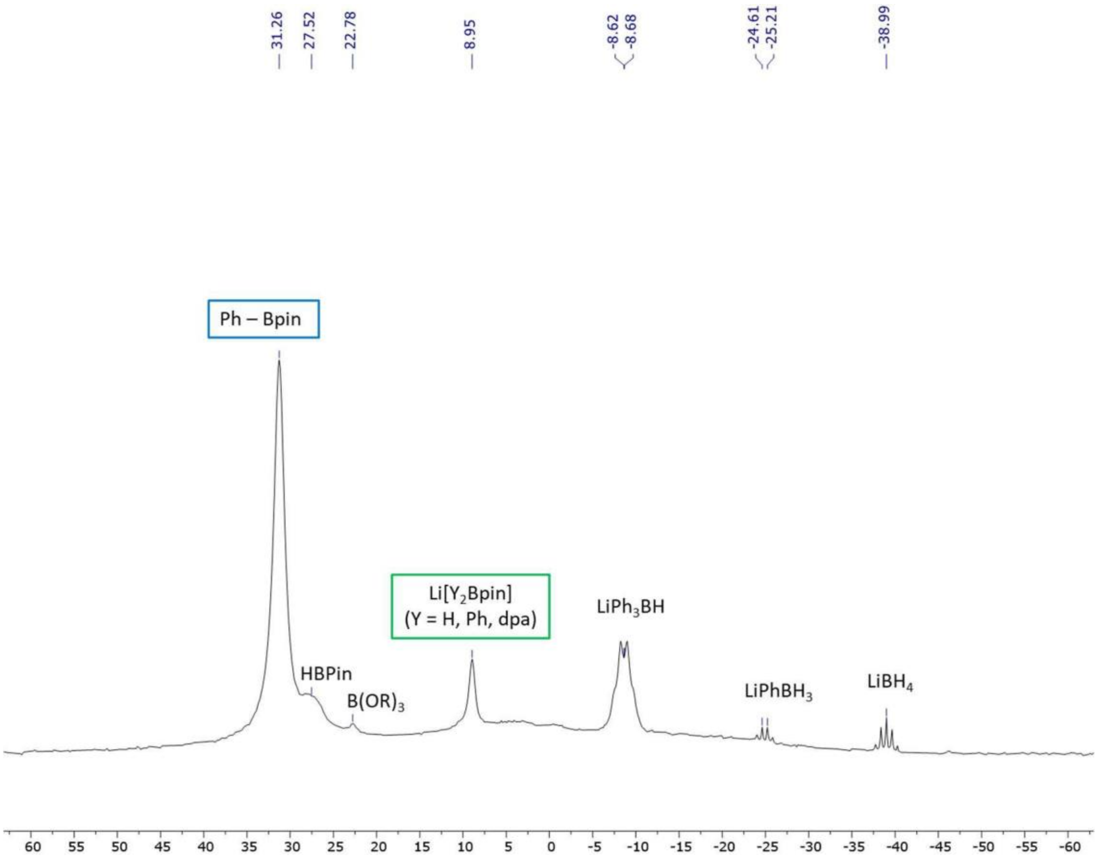

Figure S98. ^{11}B NMR spectrum of reaction of **3** with 2 eq of HBPin in C_6D_6 at room temperature after 18 h.

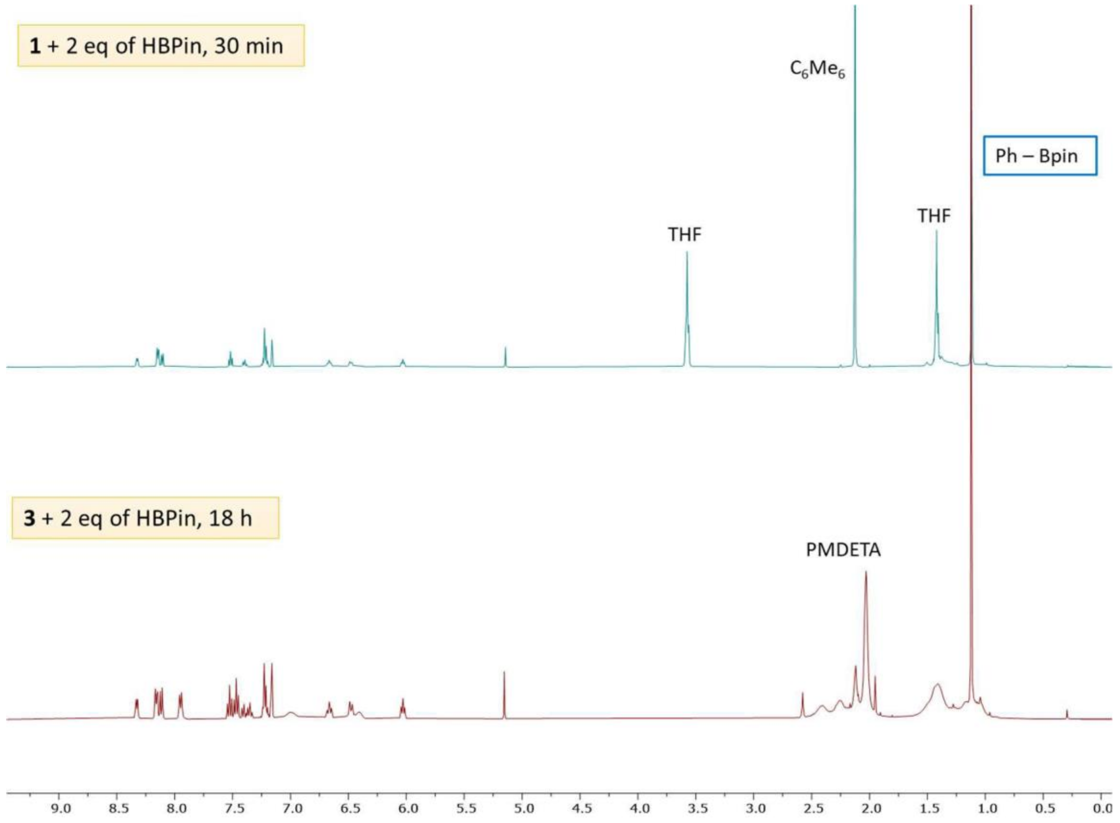

Figure S99. Stacked ^{1}H NMR spectra comparing the reactivity of lithium zincates **1** and **3** with 2 eq of HBPin in C_6D_6 at room temperature.

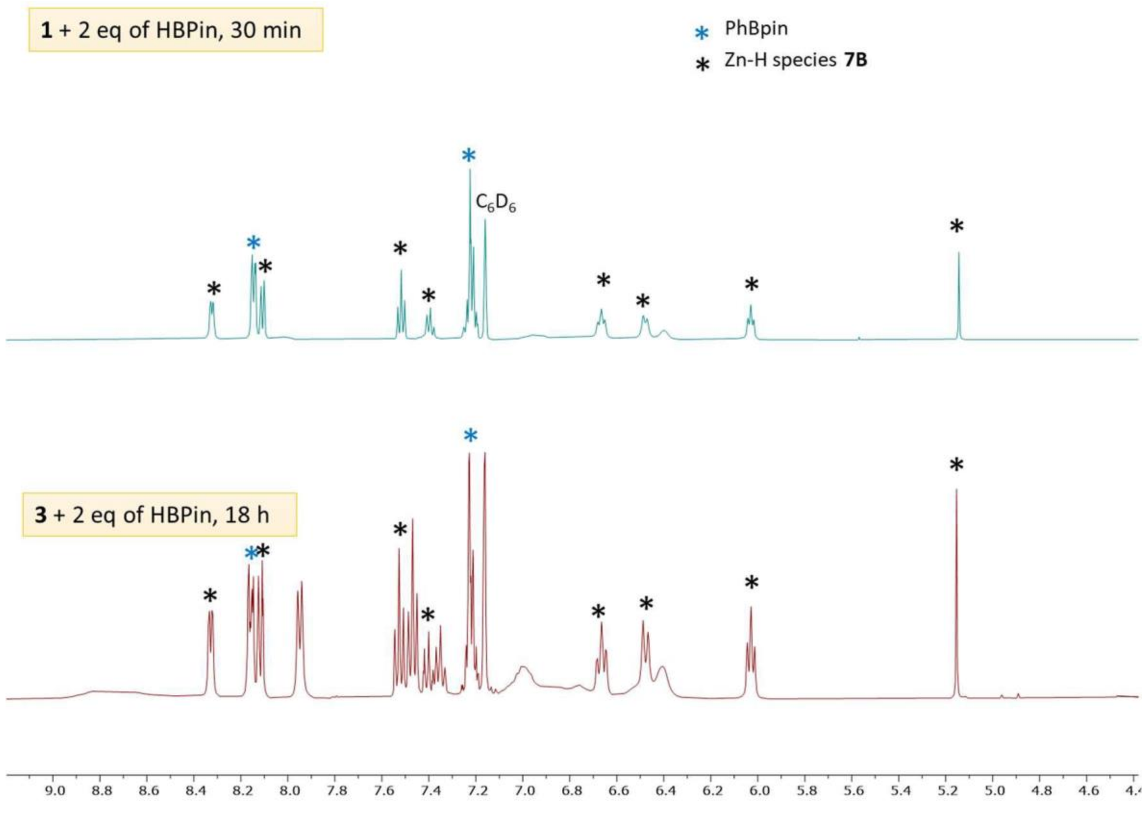

Figure S100. Stacked ^{1}H NMR spectra (9.5 – 4.5 ppm region) comparing the reactivity of **1** and **3** with 2 eq of HBPin in C_6D_6 at room temperature.

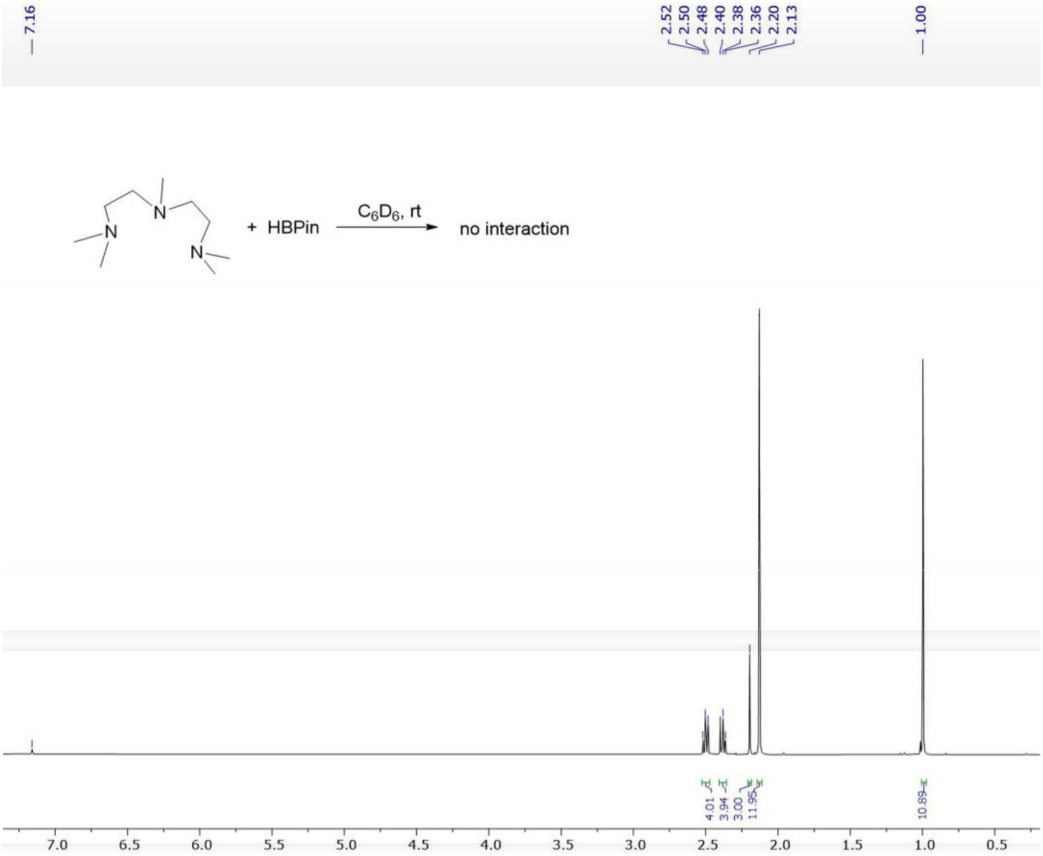

Figure 101. ^{1}H NMR spectrum of the mixture PMDETA and HBPin (1:1) in C_6D_6 at room temperature.

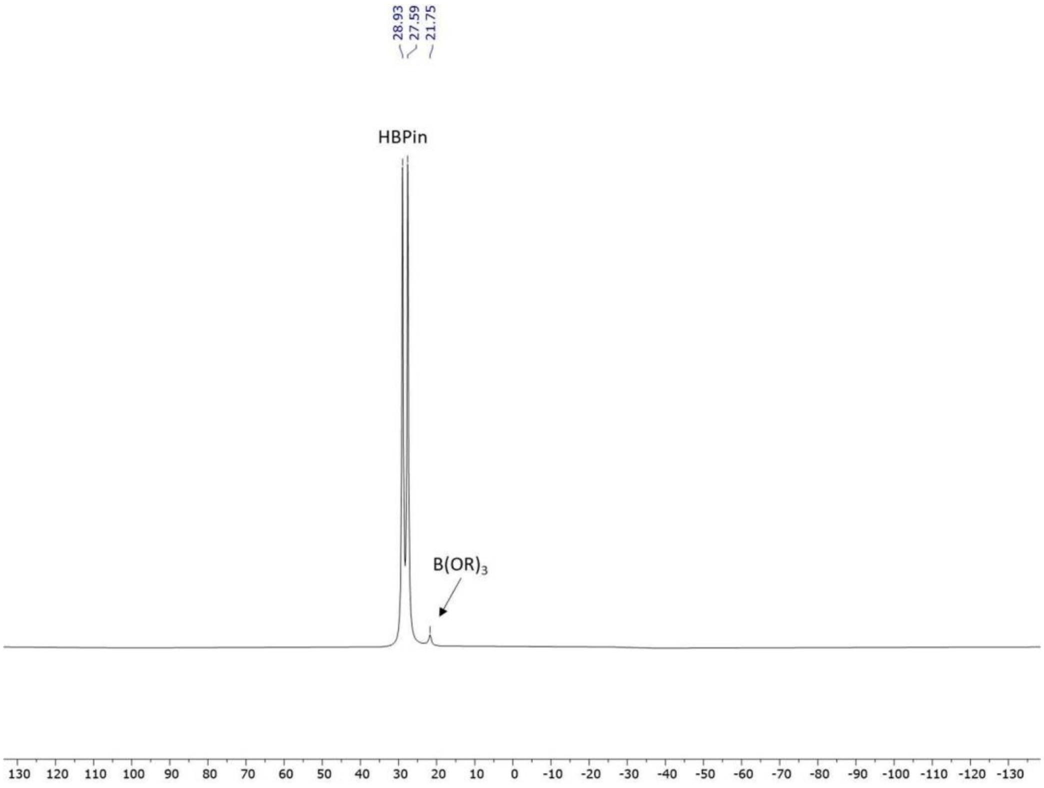

Figure S102. ^{11}B NMR spectrum of mixture PMDETA and HBPin (1:1) in C_6D_6 at room temperature.

5.5 Reactivity of 2 towards HBPin

A J Young's NMR tube was charged with **2** (30 mg, 9.64 x 10^{-5} mol, 1 eq) followed by the addition of 0.5 mL of C_6D_6 and HBpin (14 µl, 9.64 x 10^{-5} mol, 1 eq). The sealed tube was monitored over days followed by prolonged heating (Figure S103-S104) showing consumption of HBPin and formation of PhBPin. Repeating the reaction and heating the sealed tube at 80 °C for 5 h (Figure S105-S106) afforded the same outcome with evident decomposition of formed Zn-H containing species.

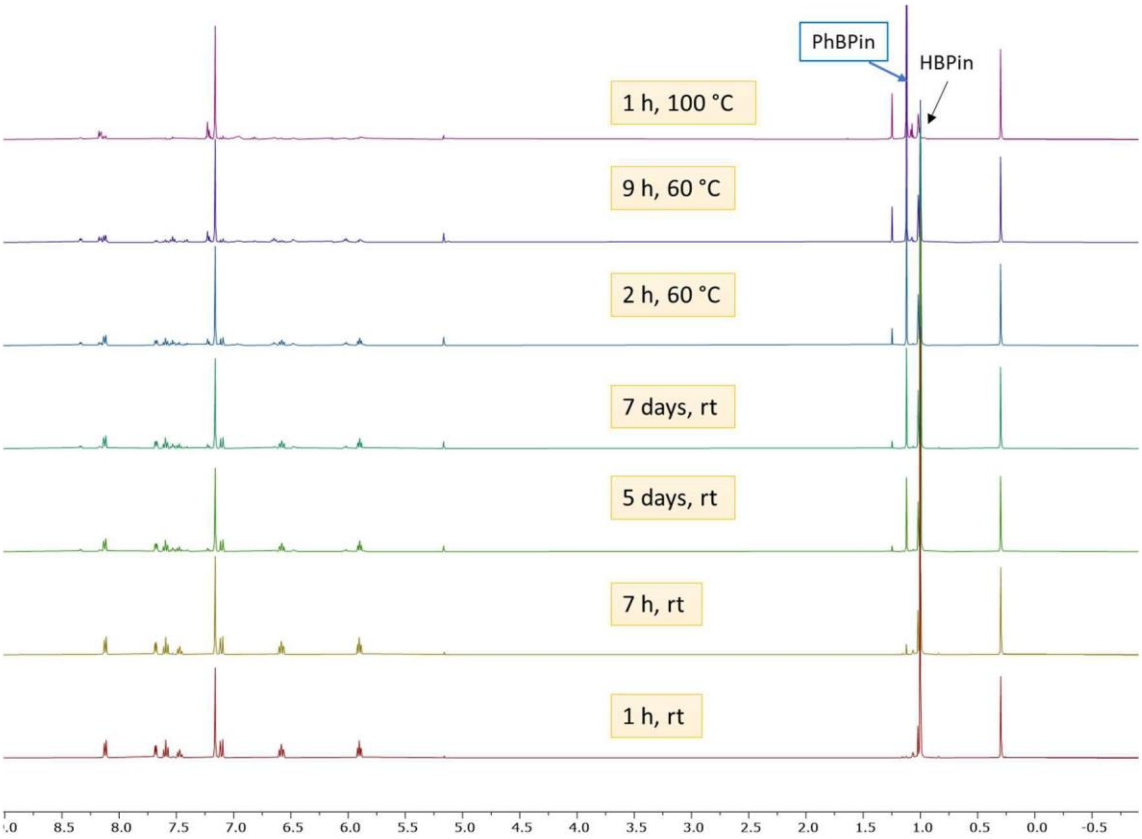

Figure S103. Stacked ^1H NMR spectra of reaction of **2** with 1 eq of HBPin in C_6D_6.

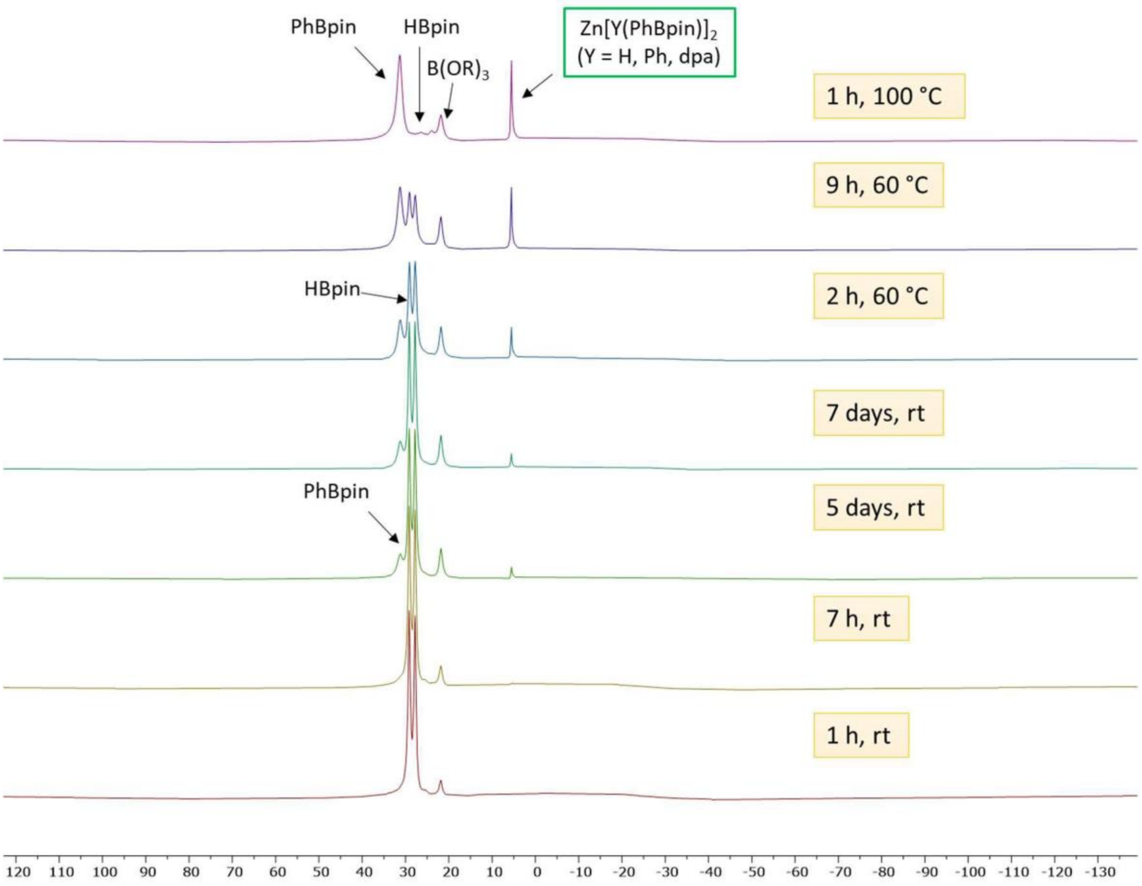

Figure S104. Stacked ^{11}B NMR spectra of reaction of **2** with 1 eq of HBPin in C_6D_6.

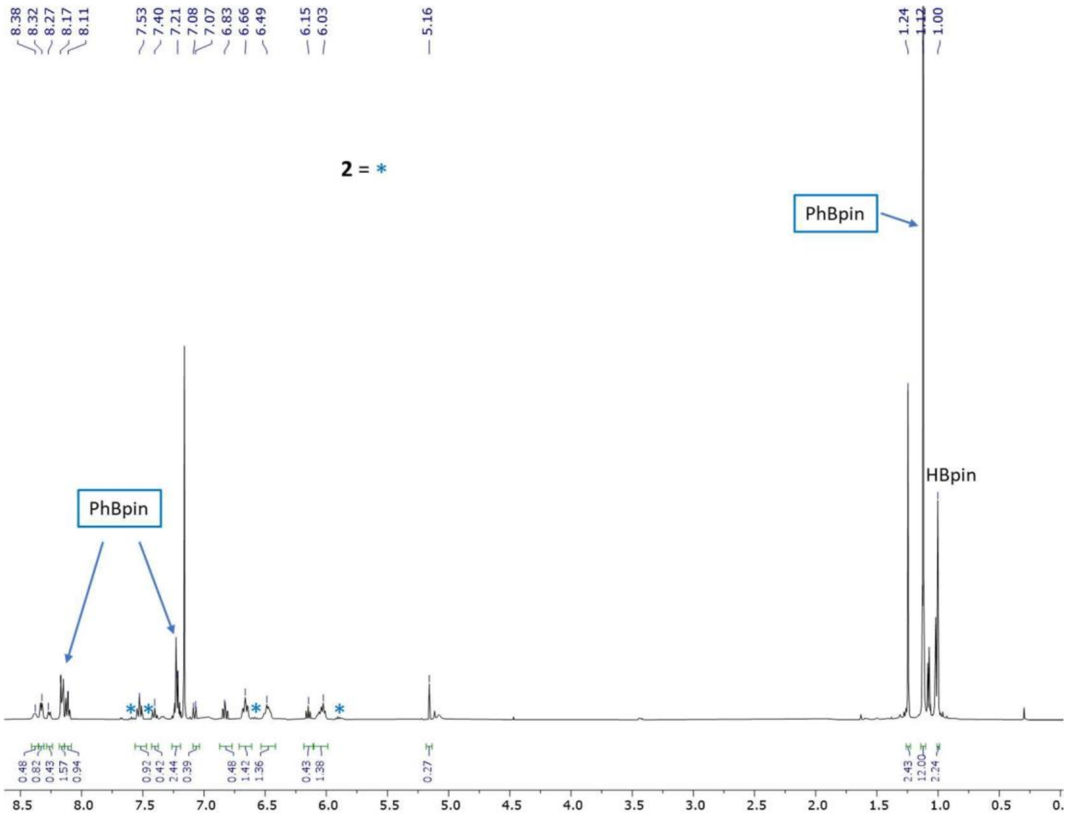

Figure S105. ^{1}H NMR spectrum of reaction of **2** with 1 eq of HBPin in C_6D_6, heating 5 h at 80 °C, recorded at room temperature.

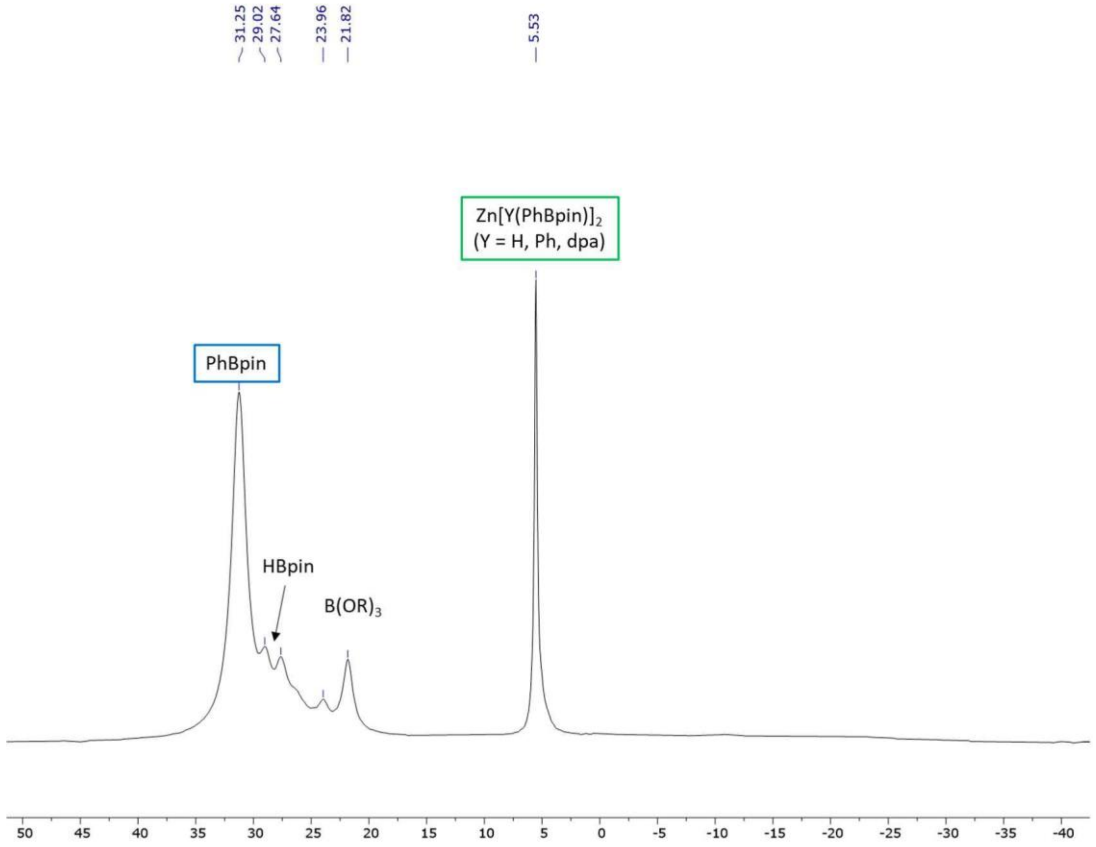

Figure S106. ^{11}B NMR spectrum of reaction of **2** with 1 eq of HBPin in C_6D_6, heating 5 h at 80 °C, recorded at room temperature.

5.6 Reactivity of 4 towards HBPin

A J Young's NMR tube was charged with **4** (20 mg, 4.94 x 10^{-5} mol, 1 eq) followed by the addition of 0.5 mL of C_6D_6 and HBpin (14.3 µl, 9.88 x 10^{-5} mol, 2 eq). The sealed tube was spun at room temperature and regularly analysed by ^1H and ^{11}B NMR spectroscopy (Figure S107-S108). In the end after 22h at room temperature only free PhBPin and free 2,2'-dipyridyl-*N*-methylamine (L) implying that L-ZnH_2 formed but decomposed (Figure S109).

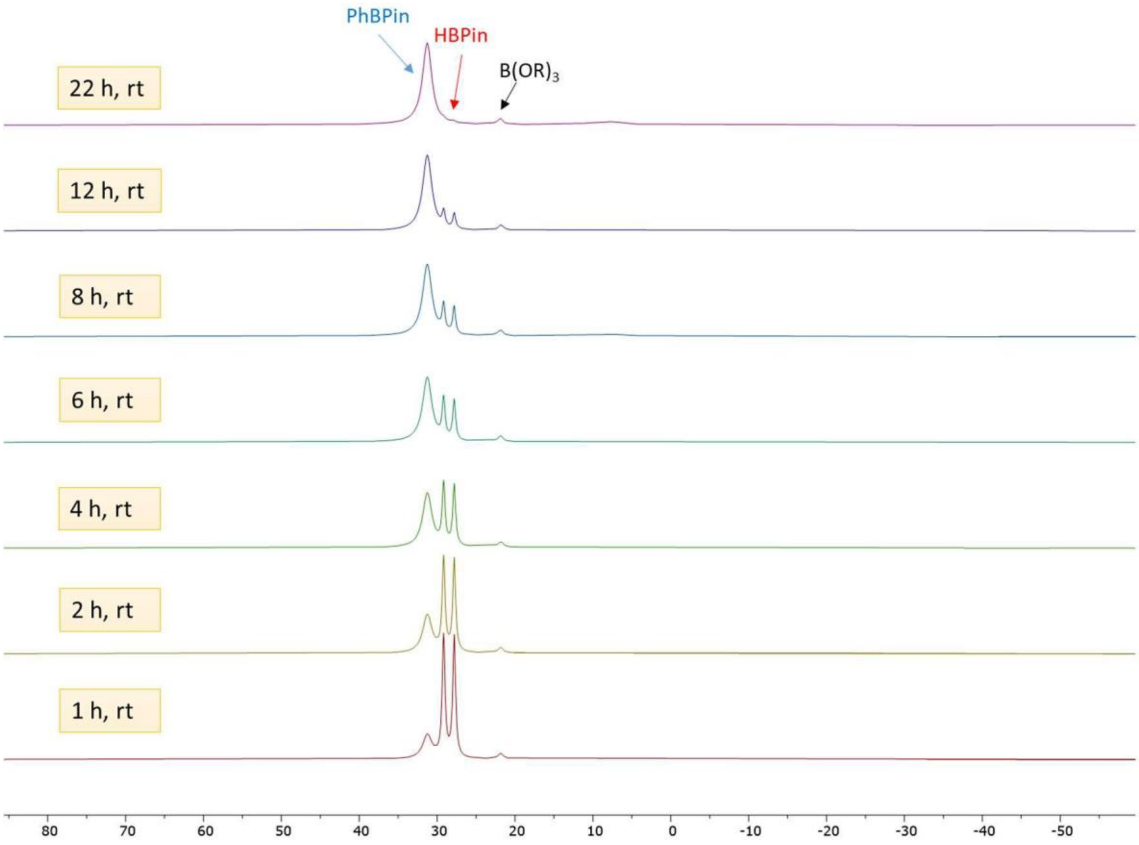

Figure S107. Stacked ^{11}B NMR spectra of reaction mixture of **4** and HBPin (1:1) in C_6D_6.

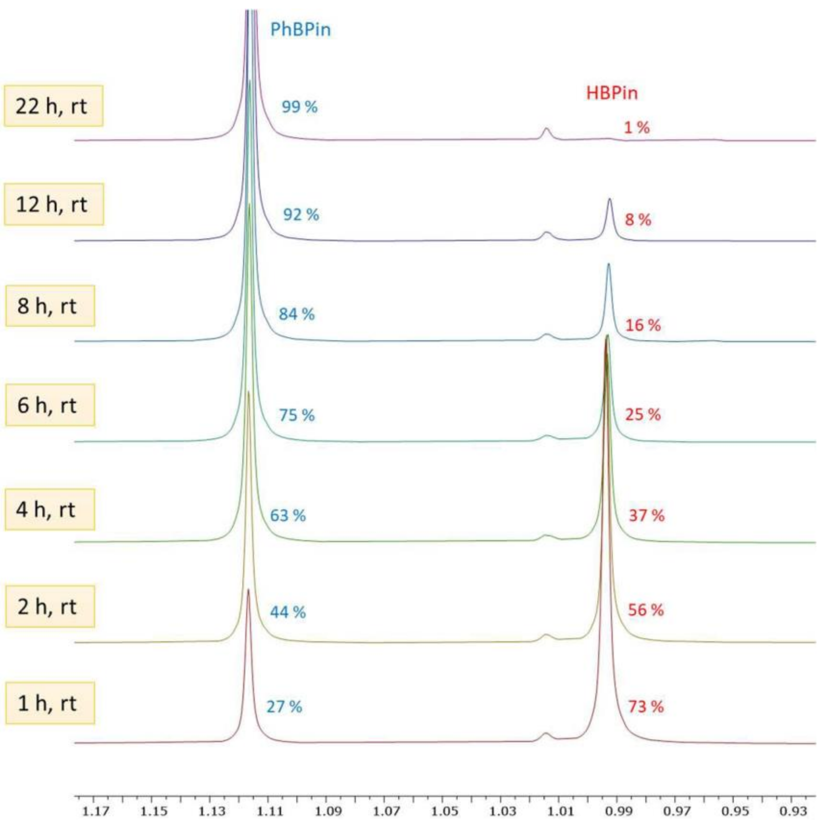

Figure S108. Stacked ^1H NMR spectra (0.9 – 1.2 ppm region) of reaction mixture of **4** and HBPin (1:1) in C_6D_6 with relative ratios of HBPin and PhBPin over time.

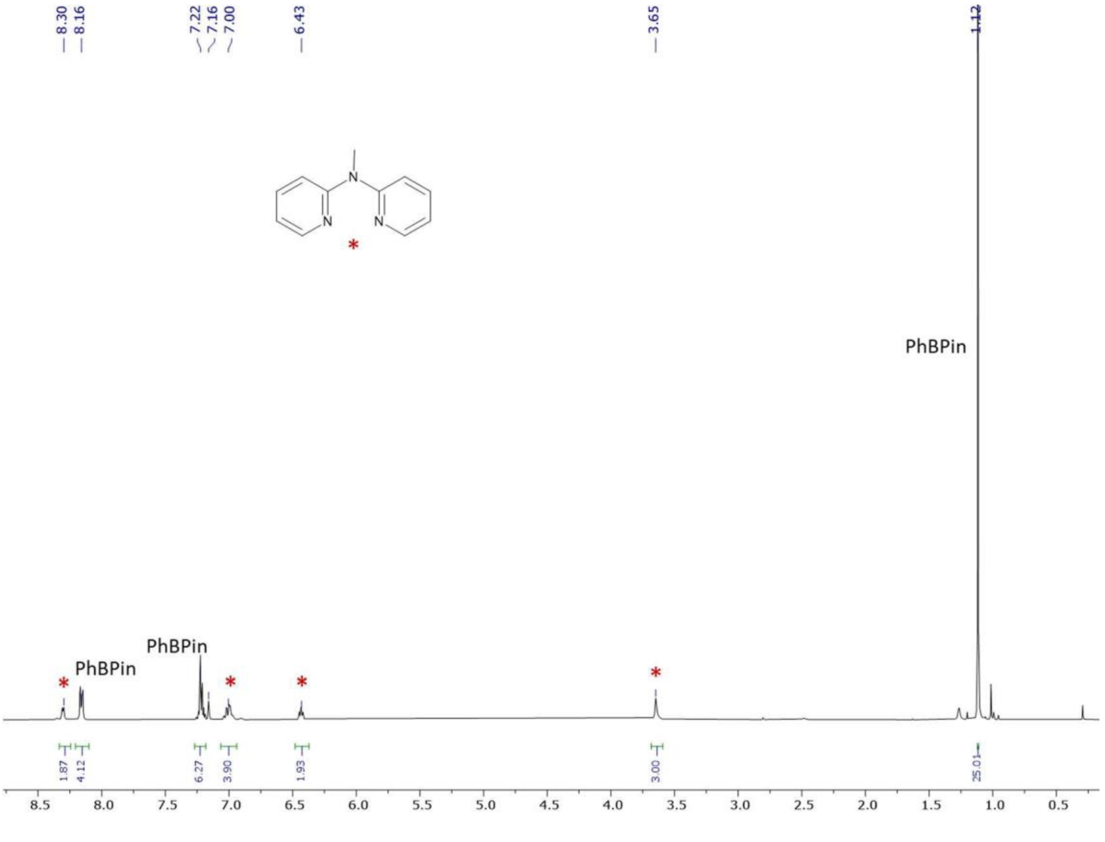

Figure S109. ^1H NMR spectrum of reaction mixture of **4** and HBPin (1:1) in C_6D_6 after 22 h at room temperature.

5.7 Reactivity of hydride species towards ketones

A J Young's NMR tube was charged with **1** (30 mg, 4.89 x 10^{-5} mol, 1 eq) followed by the addition of 0.6 mL of C_6D_6 and HBpin (7.1 µl, 4.89 x 10^{-5} mol, 1 eq). As soon as the reagents were added the mixture was analysed by ^{1}H and ^{11}B NMR spectroscopy showing the predominant formation of Zn-H species assigned as complex **7A** and PhBPin. To this mixture, α,α,α-trifluoroacetophenone (6.9 µl, 4.89 x 10^{-5} mol, 1 eq) was added and the reaction mixture was spun at room temperature for 2 h. Multinuclear NMR analysis (Figure S110-S114) showed consumption of Zn-H species **7A** and formation of predominantly M-OC(Ph)(CF_3)H, and small amounts of M-OCPh$_2$(CF_3) and hydroborated product PinBOC(Ph)(CF_3)H.[6] The formed Zn-containing species has resonances identical to complex **2**.

The reaction was repeated on a larger scale. Compound **1** (100 mg, 1.63 x 10^{-4} mol, 1 eq) was placed in a Schlenk tube and dissolved in dry benzene (1 mL) and HBPin (23.7 µL, 1.63 x 10^{-4} mol, 1 eq) was added. After 15 min, α,α,α-trifluoroacetophenone (23 µL, 1.63 x 10^{-4} mol, 1 eq) was added and after 2 h stirring at room temperature, the reaction mixture was quenched with HCl (1M, 2 mL) and left stirring overnight. The colourless solution was diluted with water and extracted with diethyl ether (3 x 2 mL). Combined organic layers were dried over Na_2SO_4 and most of the solvent was removed by N_2 bubbling, due to the volatility of product.[7] Obtained crude was dissolved in $CDCl_3$ (Figure S115–S116) to which 1,3,5-trifluorobenzene (16.7 µL, 1.63x10^{-4} mol, 1 eq) was added to determine NMR yield of 2,2,2-trifluoro-1-phenylethanol (67%) and 1,1-diphenyl-2,2,2-trifluoroethanol side-product (6 %).

^{1}H NMR (500 MHz, $CDCl_3$, 298 K): 2.89 (br, 1H); 5.04 (q, *J* = 6.8 Hz, 1H, CHCF$_3$); 7.44 (m, 3H, Ar*H*); 7.56 (m, 2H, Ar*H*). ^{19}F NMR (376.4 MHz, $CDCl_3$, 298 K): -78.2 (d, *J* = 6.8 Hz, C*F*$_3$).

The reaction was next attempted using 2 equivalents of HBPin under otherwise identical conditions. Multinuclear NMR analysis (Figure S117-S121) revealed that after initial formation of the "second" Zn-H species **7B**, it does not transfer the hydride ligand to ketone but persists over a period of 21 h. However, the hydroborated product forms in significant amount.

The potential of **7A** to transfer hydride to several other ketones was tested – specifically 4-fluorobenzophenone, benzophenone and acetophenone. Compound **1** (100 mg, 1.63 x 10^{-4} mol, 1 eq) was placed in a Schlenk tube and dissolved in dry benzene (1 mL) and HBPin (23.7 µL, 1.63 x 10^{-4} mol, 1 eq) was added. After 15 min, the selected ketone (1 eq) was added and after 2 h stirring at room temperature, the reaction mixture was quenched with HCl (1M, 2 mL) and left stirring for 3 h. The colourless solution then was diluted with water and extracted with ethyl acetate (3 x 2 mL). Combined organic layers were dried over Na_2SO_4 and the solvent was evaporated. Obtained crude was dissolved in $CDCl_3$ to which dibromomethane (11.4 µL, 1.63x10^{-4} mol, 1 eq) was added to determine NMR yield.

Bis(4-fluorophenyl)methanol (**11**, 58%). ^{1}H NMR (500 MHz, $CDCl_3$, 298 K): 2.32 (br, 1H); 5.81 (s, 1H); 7.03 (m, 4H, Ar*H*); 7.32 (m, 4H, Ar*H*). ^{19}F NMR (376.4 MHz, $CDCl_3$, 298 K): -114.7 (Figure S122 – S123).

1,1-diphenylmethanol (**12**, 64%) ^{1}H NMR (500 MHz, $CDCl_3$, 298 K): 2.34 (br, 1H); 5.85 (s, 1H); 7.32 – 7.40 (m, 10H, Ar*H*). Formation of side product triphenylmethanol (10%) was also observed (Figure S124).

1-phenylethanol (**13**, 54%). ^{1}H NMR (500 MHz, $CDCl_3$, 298 K): 1.53 (d, *J* = 6.5 Hz, 3H), 1.95 (br, 1H); 4.92 (q, *J* = 6.5 Hz, 1H); 7.27 – 7.32 (m, 1H, Ar*H*); 7.35 – 7.42 (m, 4H, Ar*H*). Unreacted acetophenone (45%) was also observed (Figure S125).

Control reactions showed that HBPin on its own hydroborates α,α,α-trifluoroacetophenone only in trace amounts even after 3 days (Figure S126-S128), and the complex Lidpa·HBPin is unreactive (Figure S129-S131).

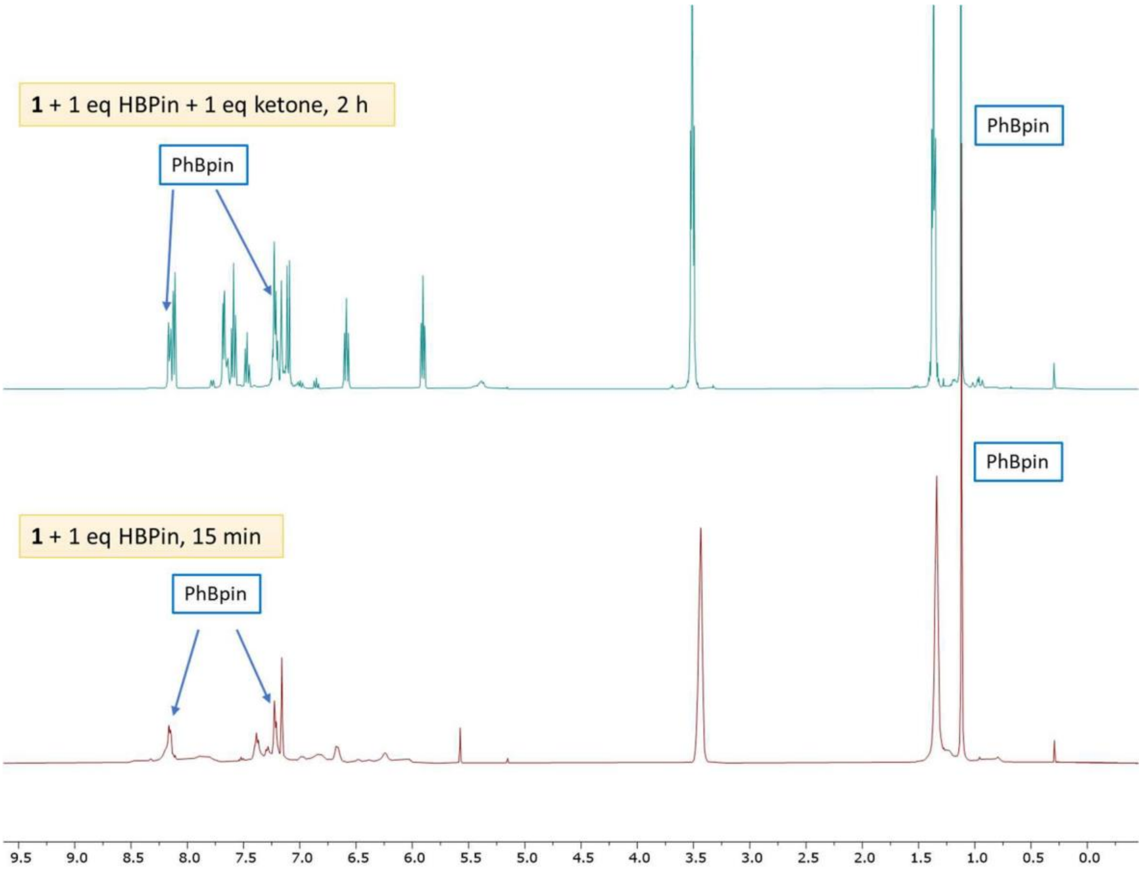

Figure S110. Stacked ^{1}H NMR spectra of reaction mixture of **1** and HBPin (1:1) in C_6D_6 and α,α,α-trifluoroacetophenone at room temperature.

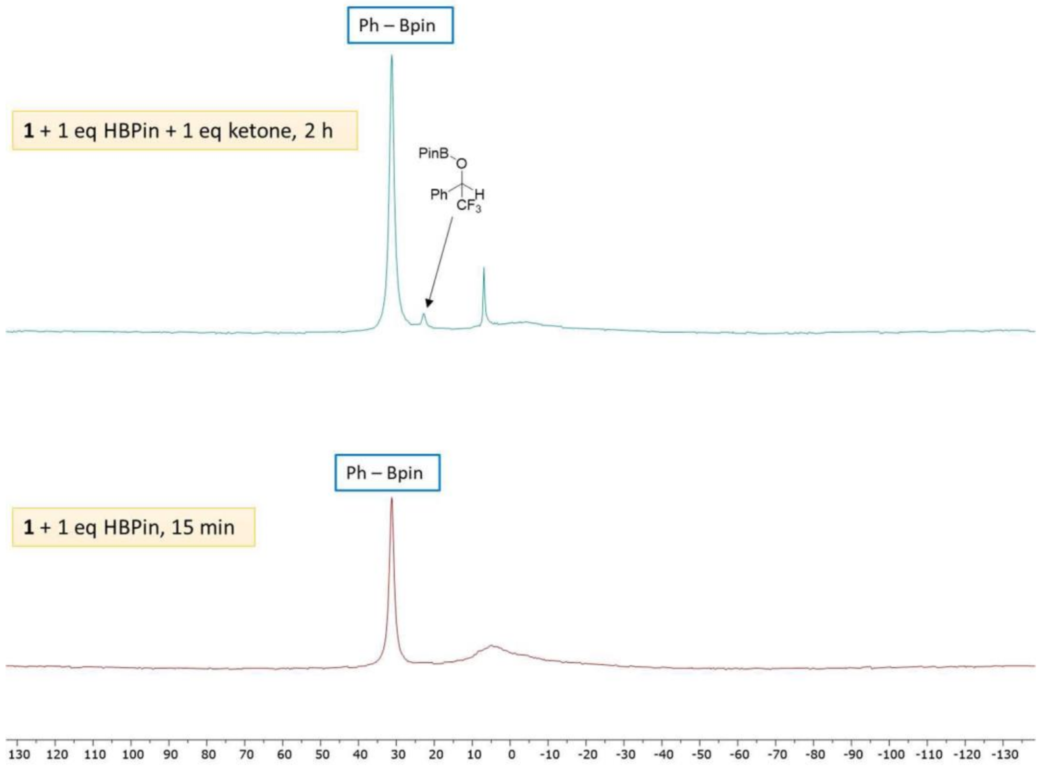

Figure S111. Stacked ^{11}B NMR spectra of reaction mixture of **1** and HBPin (1:1) in C_6D_6 and α,α,α-trifluoroacetophenone at room temperature.

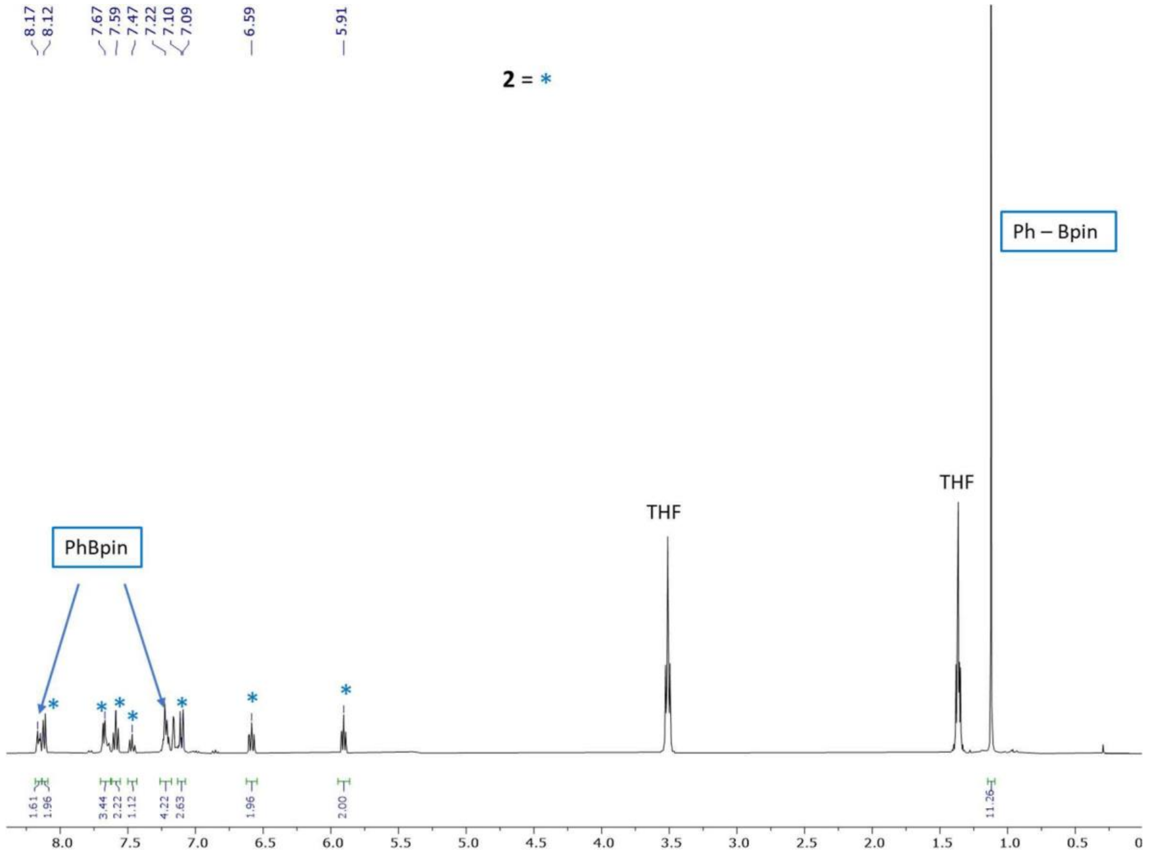

Figure S112. ^{1}H NMR spectrum of reaction mixture of **1** and HBPin (1:1) in C_6D_6 and α,α,α-trifluoroacetophenone at room temperature.

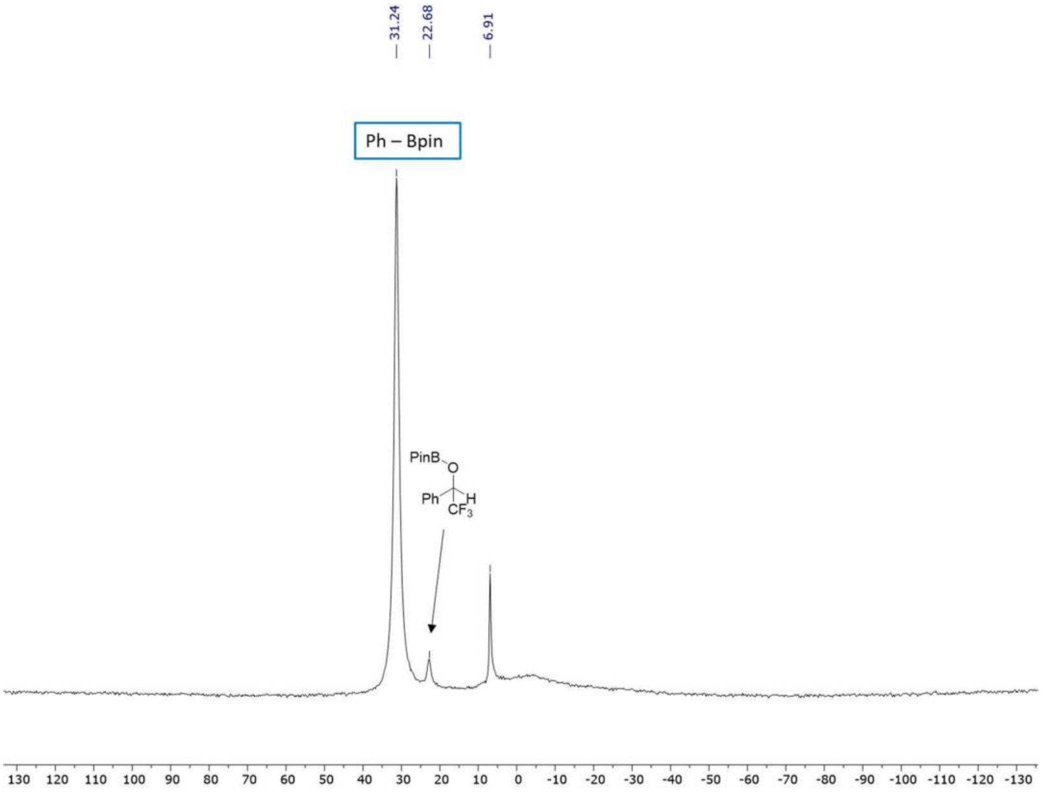

Figure S113. ^{11}B NMR spectrum of reaction mixture of **1** and HBPin (1:1) in C_6D_6 and α,α,α-trifluoroacetophenone at room temperature.

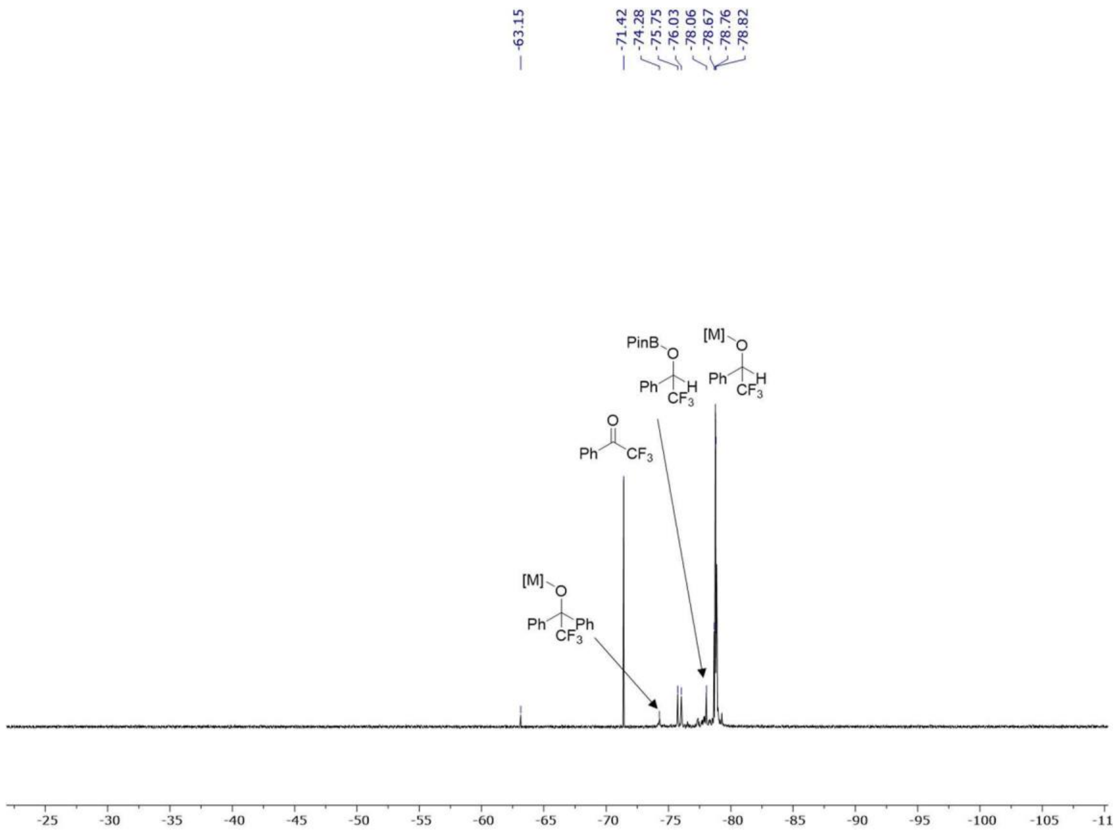

Figure S114. ^{19}F NMR spectrum of reaction mixture of **1** and HBPin (1:1) in C_6D_6 and α,α,α-trifluoroacetophenone at room temperature.

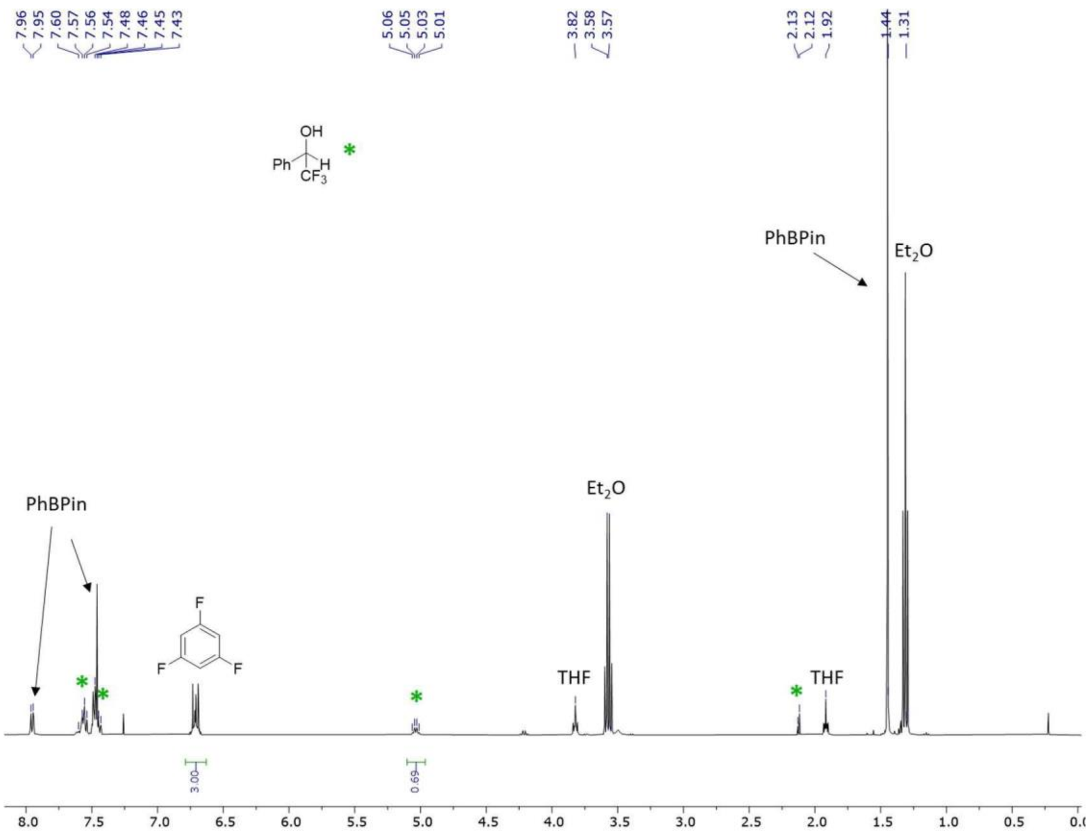

Figure S115. ^{1}H NMR spectrum of hydrolysed crude reaction mixture in $CDCl_3$ and with 1,3,5-trifluorobenzene (1 eq) as internal standard at room temperature to determine yield.

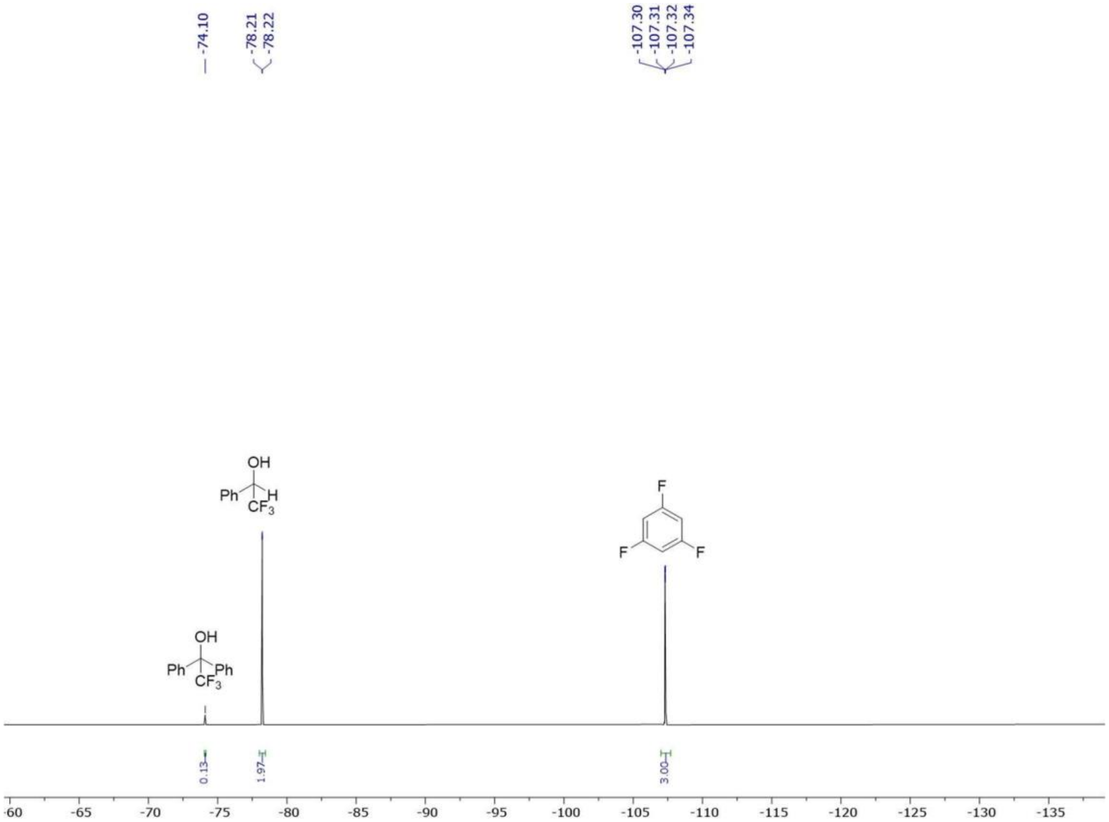

Figure S116. ^{19}F NMR spectrum of hydrolysed crude reaction mixture in $CDCl_3$ and with 1,3,5-trifluorobenzene (1 eq) as internal standard at room temperature to determine yield.

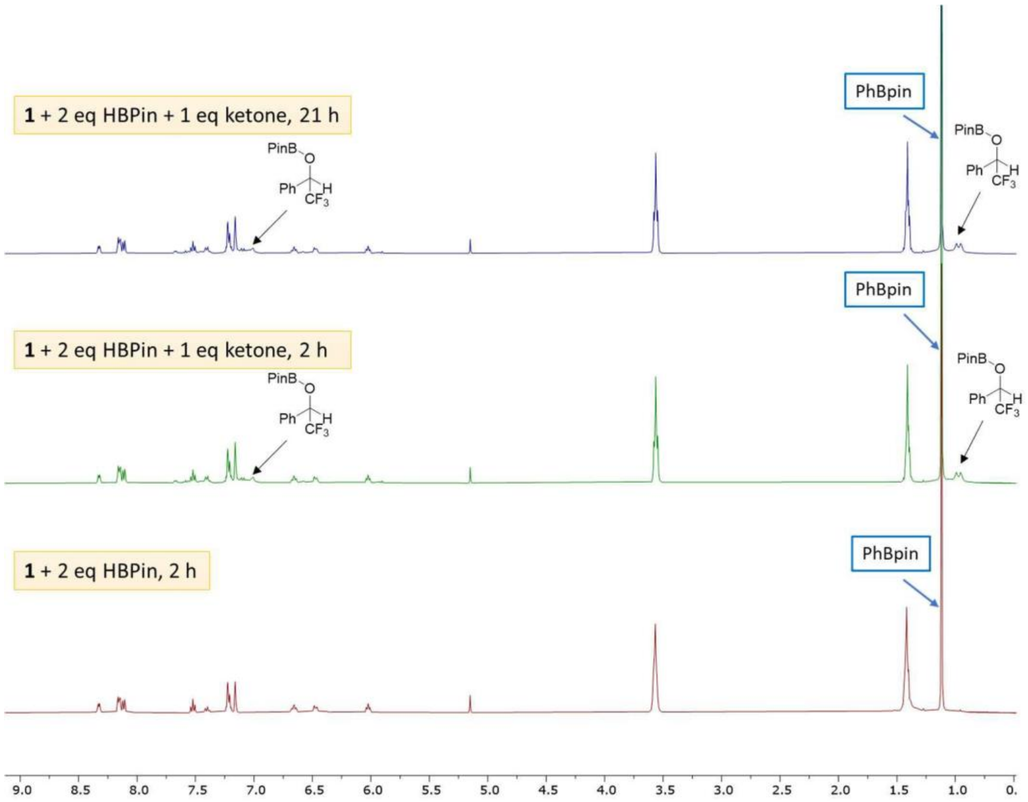

Figure S117. Stacked ^{1}H NMR spectra of reaction mixture of **1** and HBPin (1:2) in C_6D_6 and α,α,α-trifluoroacetophenone at room temperature.

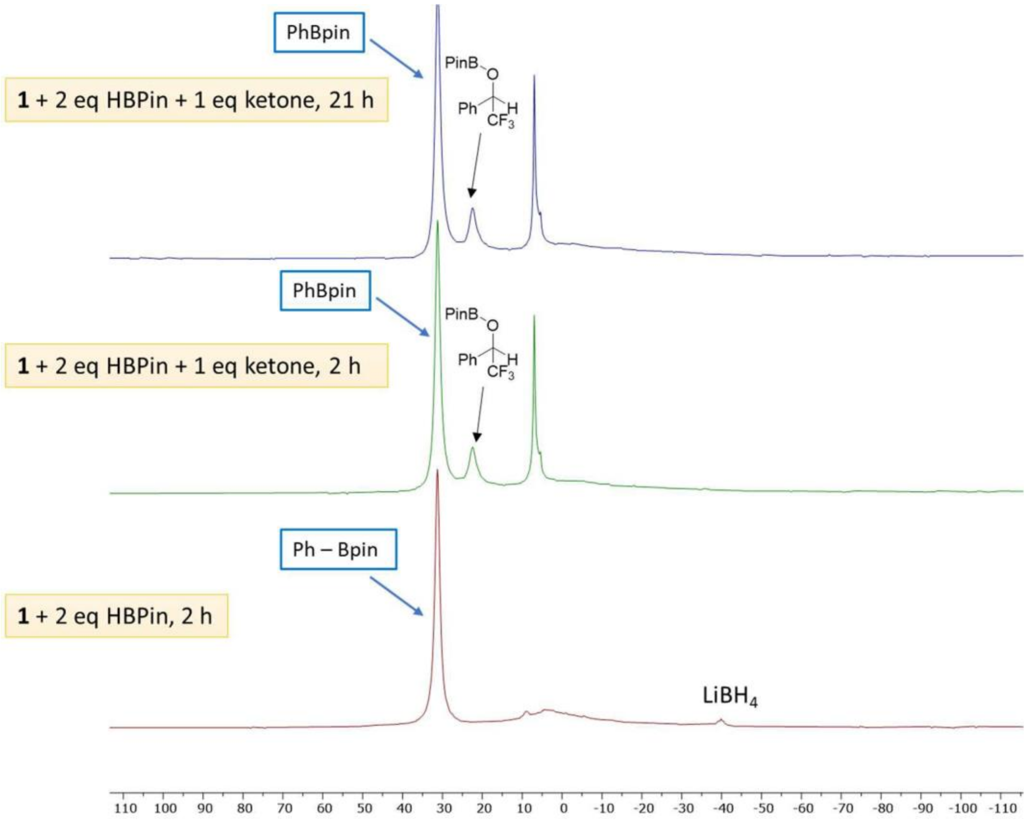

Figure S118. Stacked ^{11}B NMR spectra of reaction mixture of **1** and HBPin (1:2) in C_6D_6 and α,α,α-trifluoroacetophenone at room temperature.

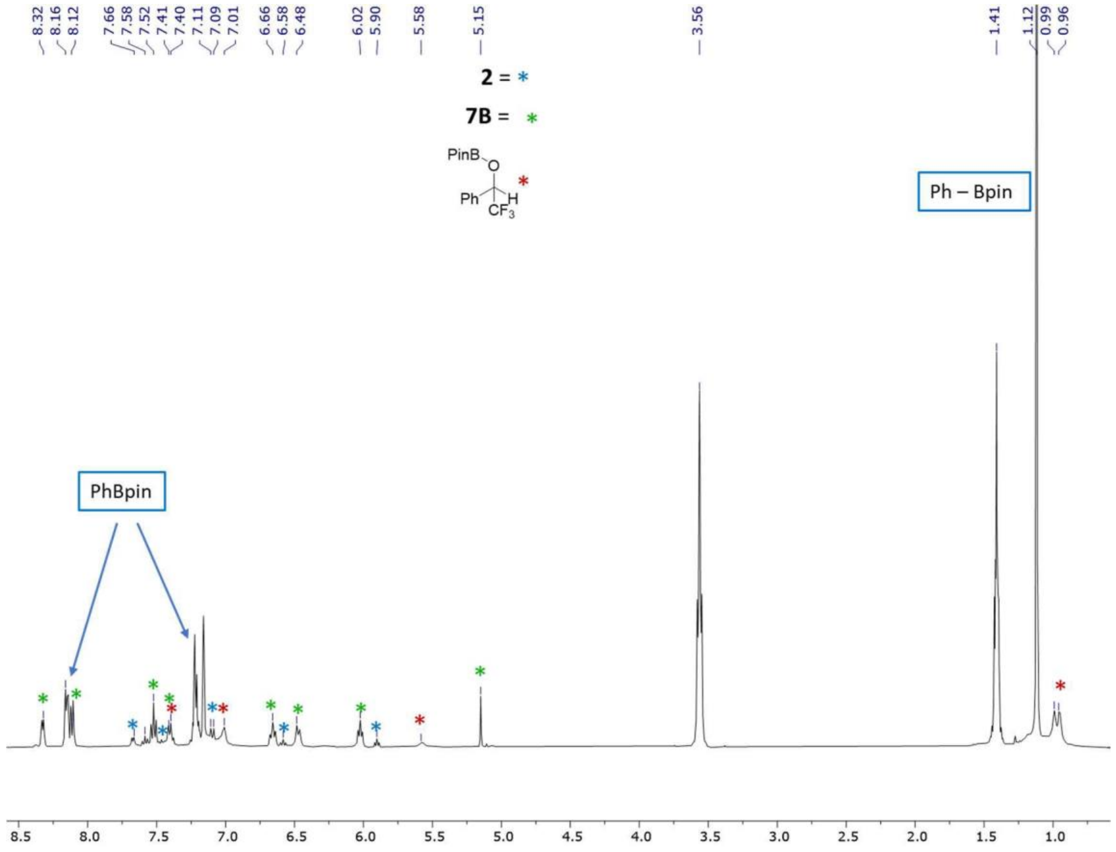

Figure S119. ^{1}H NMR spectrum of reaction mixture of **1** and HBPin (1:2) in C_6D_6 and 1 eq of α,α,α-trifluoroacetophenone at room temperature after 21 h.

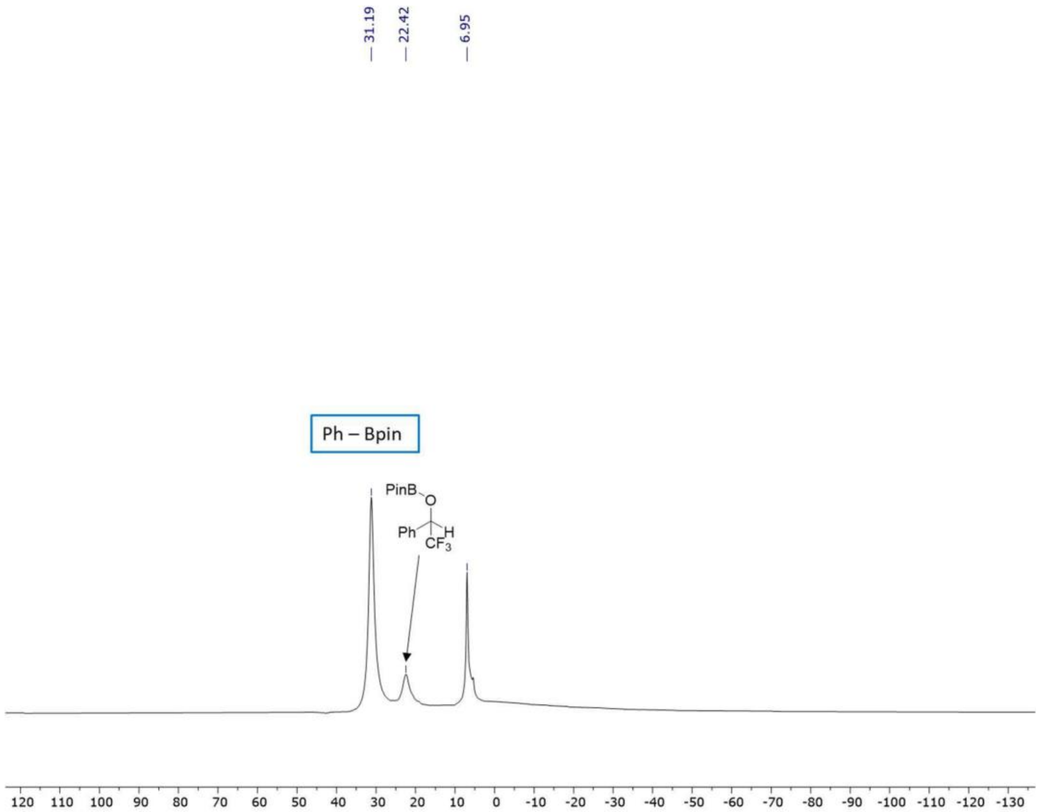

Figure S120. ^{11}B NMR spectrum of reaction mixture of **1** and HBPin (1:2) in C_6D_6 and 1 eq of α,α,α-trifluoroacetophenone at room temperature after 21 h.

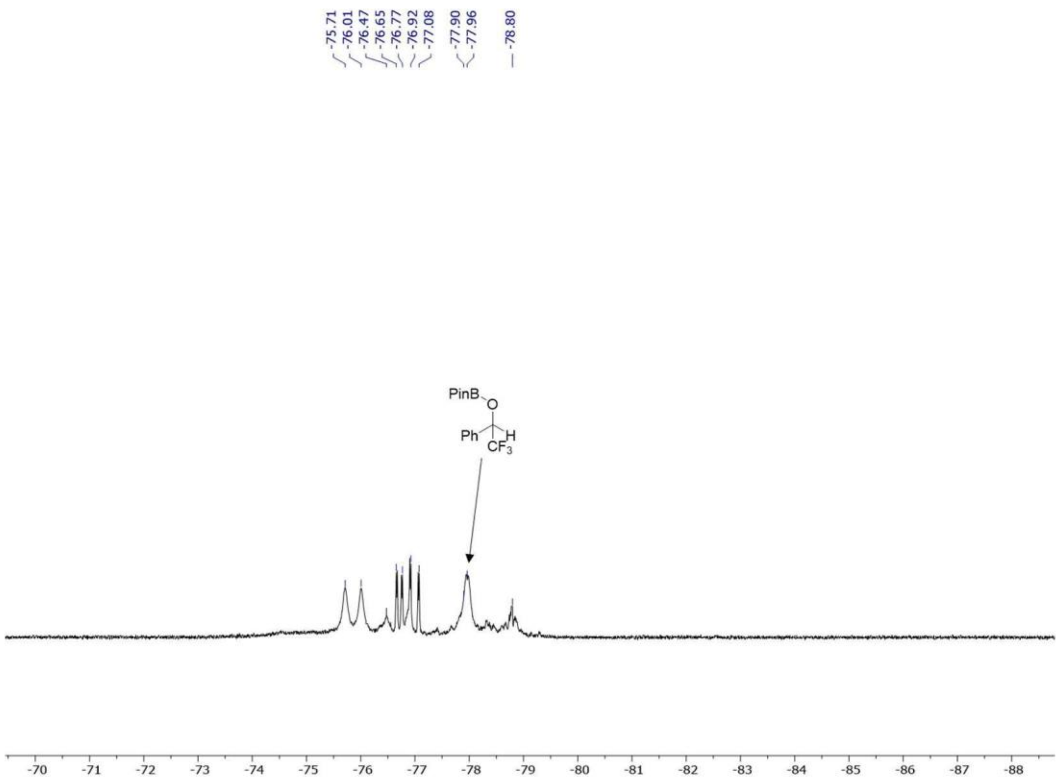

Figure S121. ^{19}F NMR spectrum of reaction mixture of **1** and HBPin (1:2) in C_6D_6 and 1 eq of α,α,α-trifluoroacetophenone at room temperature.

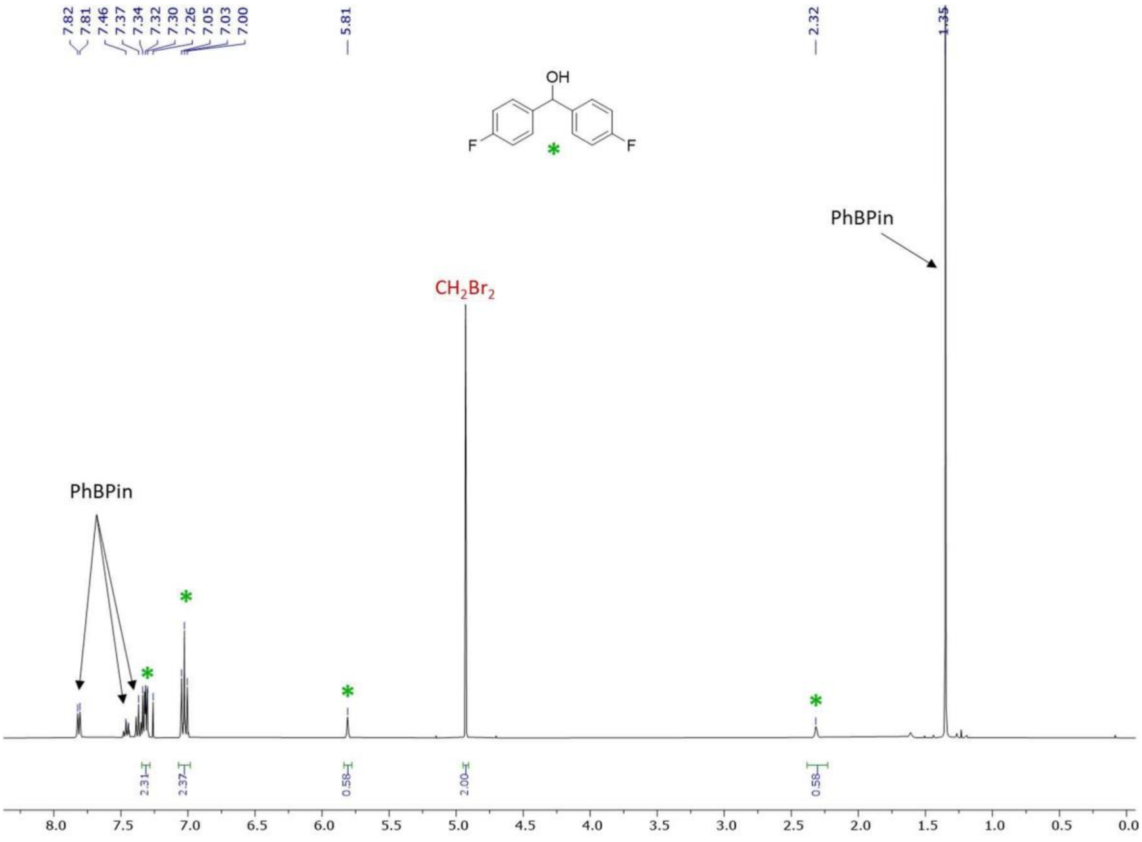

Figure S122. ^{1}H NMR spectrum of the crude product **11** (58%) using CH_2Br_2 (1 eq) as internal standard in $CDCl_3$ at room temperature.

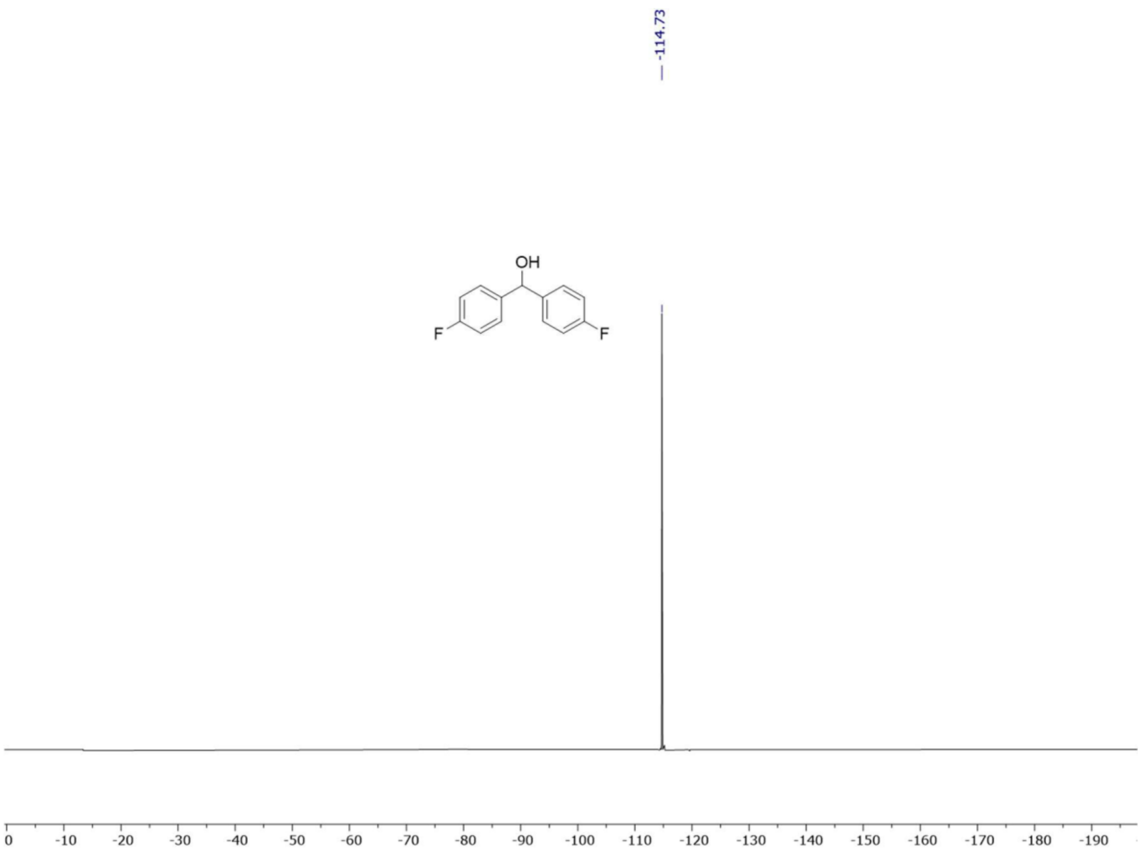

Figure S123. ^{19}F NMR spectrum of the crude product **11** (58%) in $CDCl_3$ at room temperature.

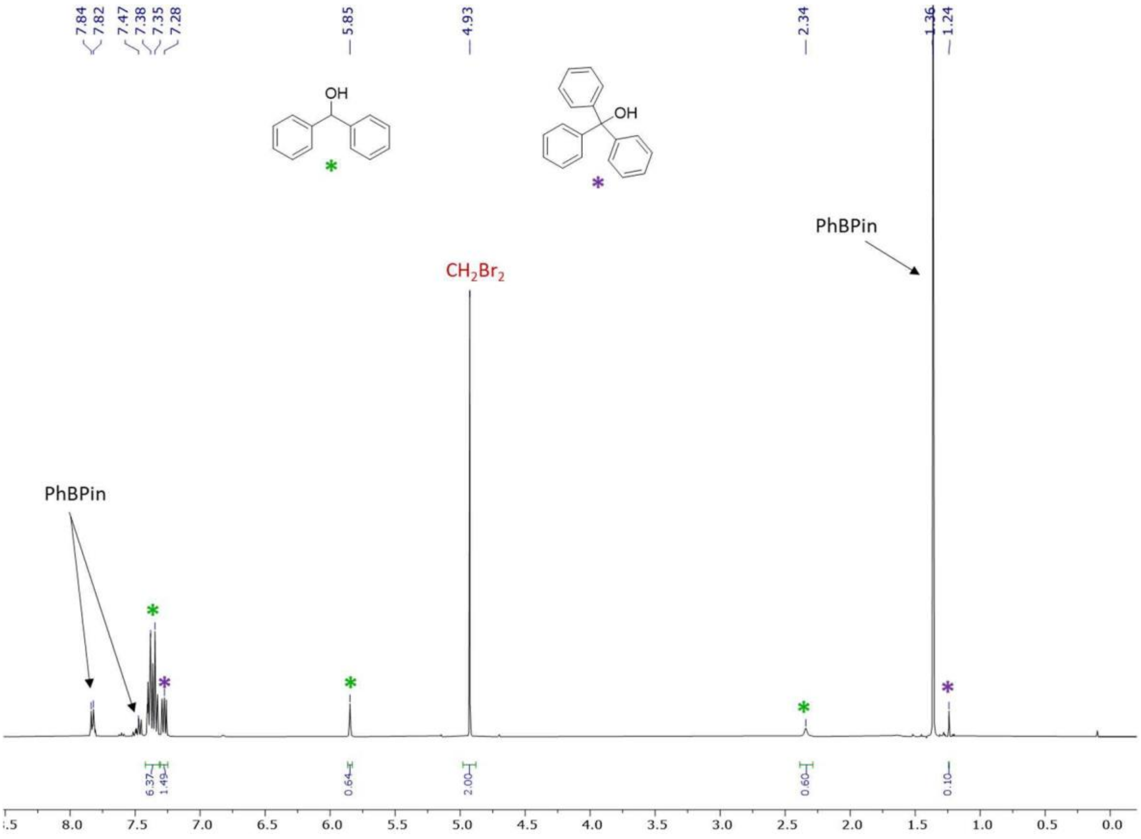

Figure S124. ^{1}H NMR spectrum of the crude product **12** (64%) and side-product triphenylmethanol (10%) using CH_2Br_2 (1 eq) as internal standard in $CDCl_3$ at room temperature.

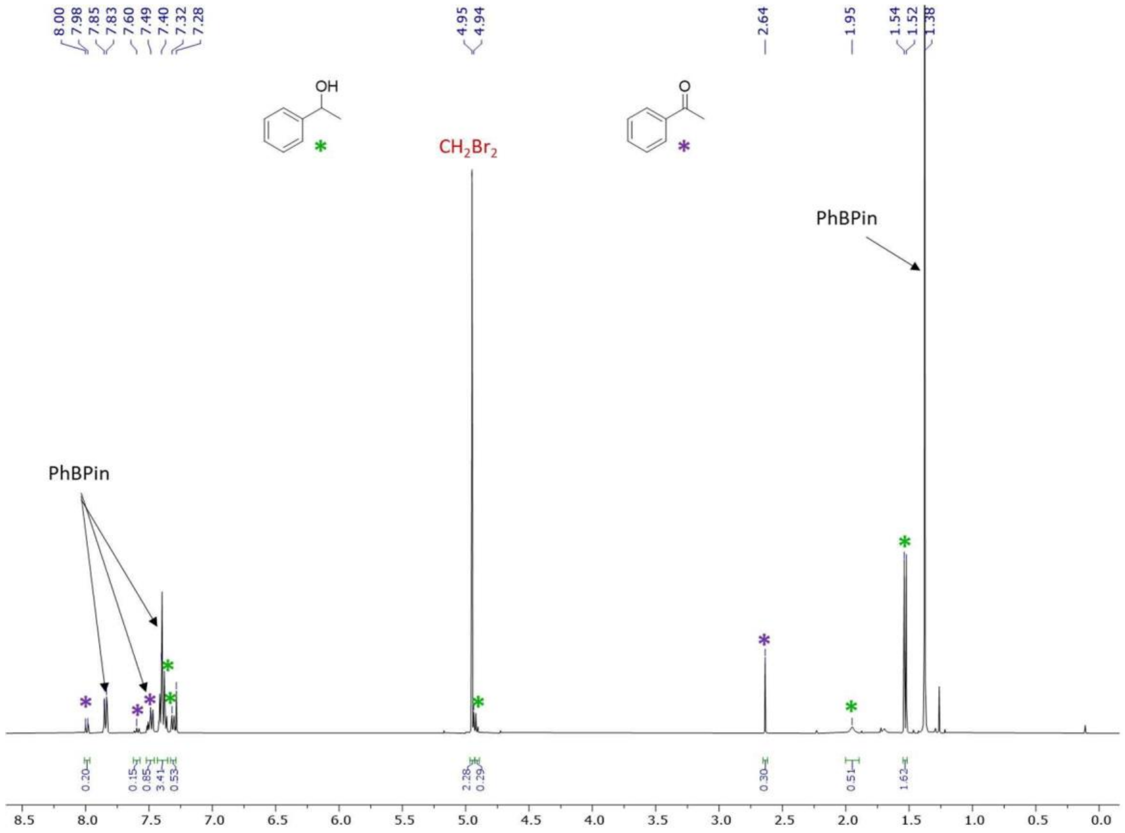

Figure S125. ^1H NMR spectrum of the crude product **13** (54%) and left-over starting material (45%) using CH_2Br_2 (1 eq) as internal standard in $CDCl_3$ at room temperature.

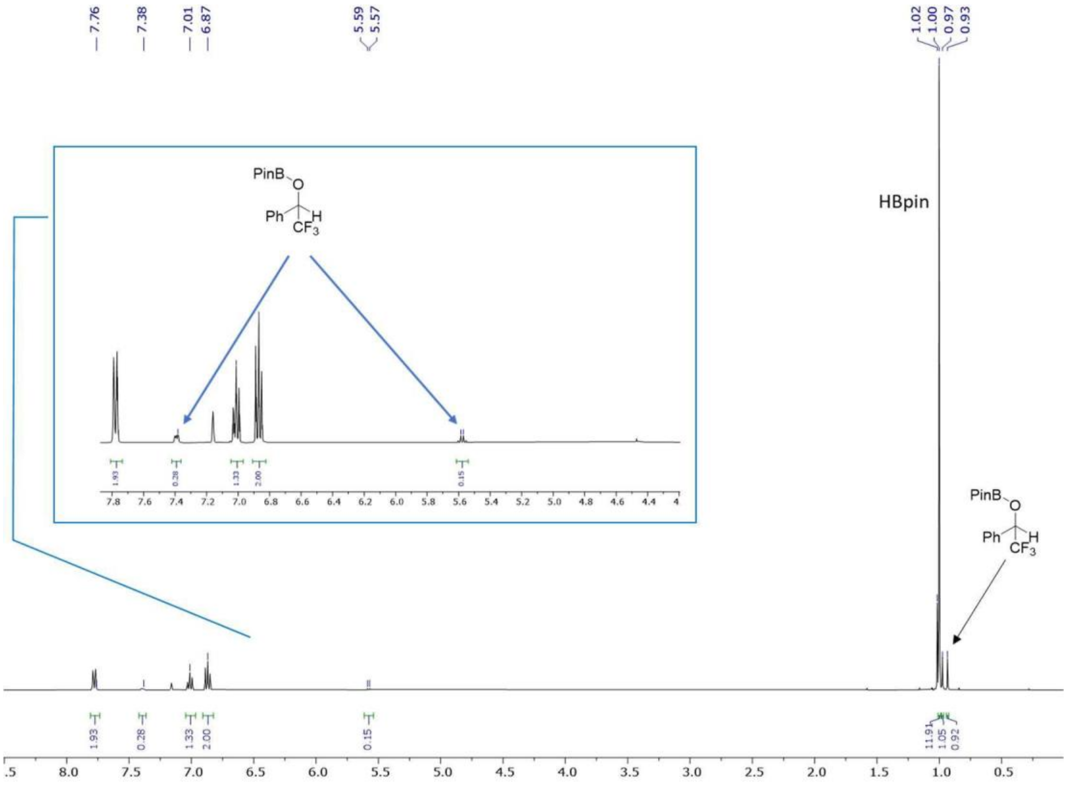

Figure S126. ^1H NMR spectrum of reaction of HBPin and α,α,α-trifluoroacetophenone (1:1) in C_6D_6 after 3 days at room temperature.

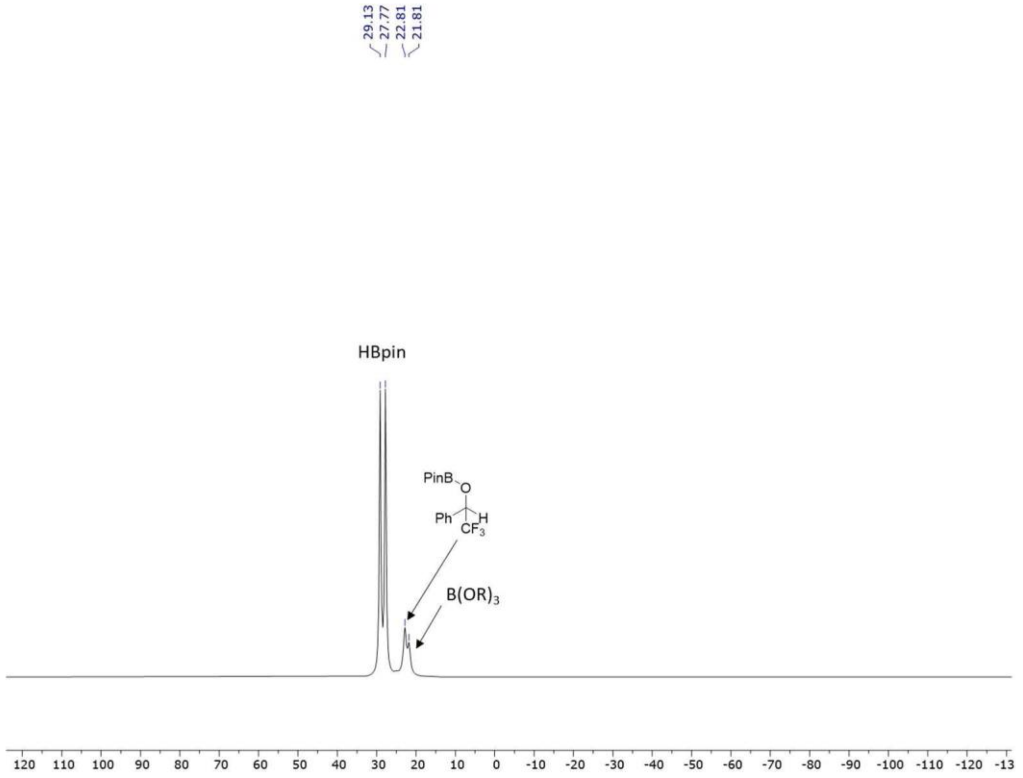

Figure S127. ^{11}B NMR spectrum of reaction of HBPin and α,α,α-trifluoroacetophenone (1:1) in C_6D_6 after 3 days at room temperature.

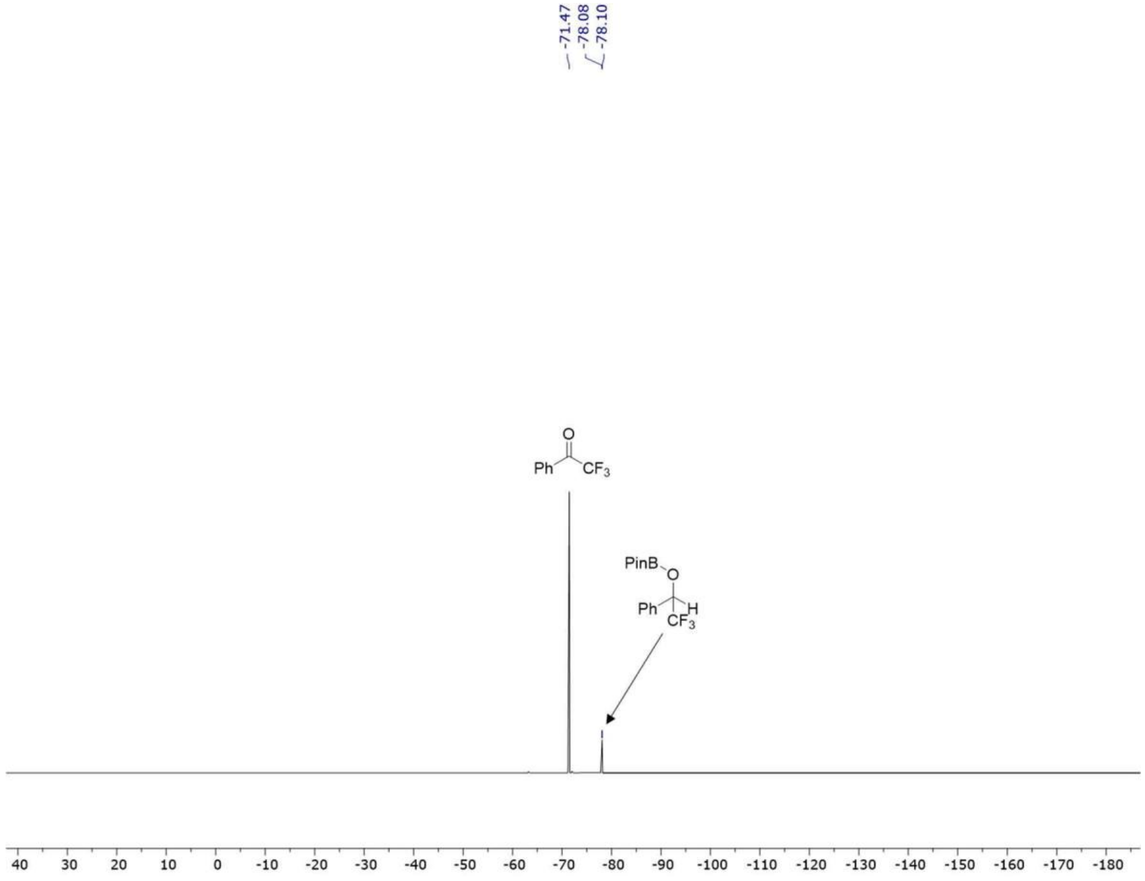

Figure S128. ^{19}F NMR spectrum of reaction of HBPin and α,α,α-trifluoroacetophenone (1:1) in C_6D_6 after 3 days at room temperature.

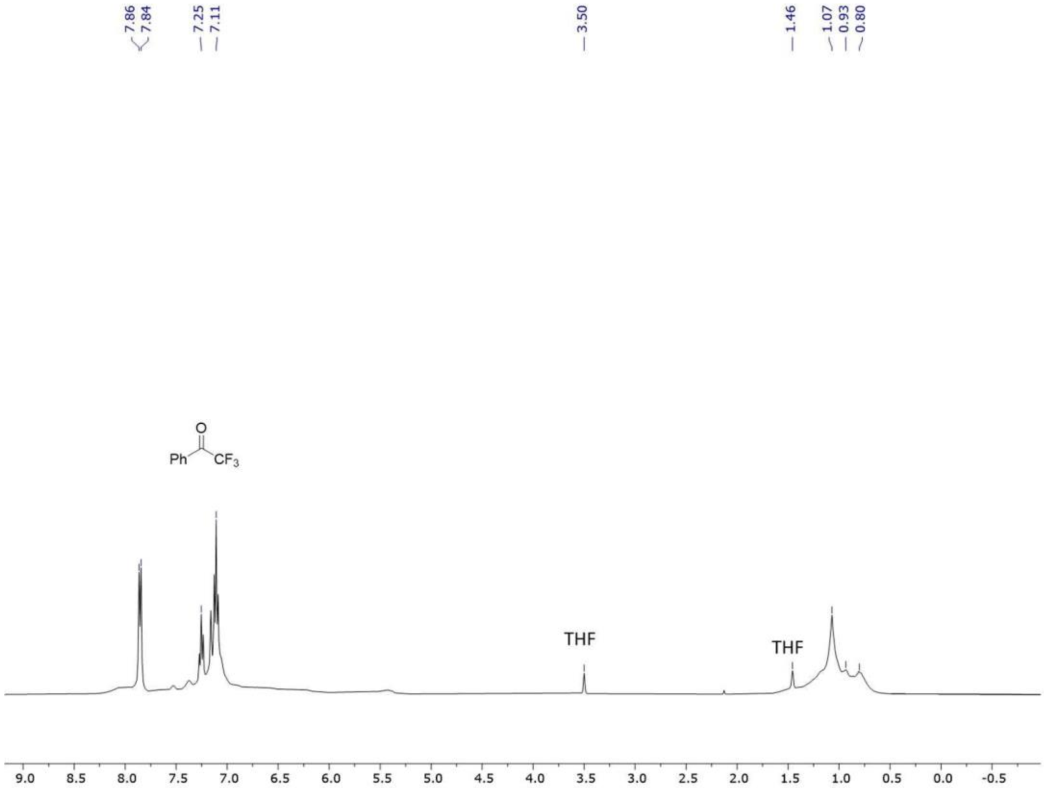

Figure S129. ^{1}H NMR spectrum of reaction of HBPin·Lidpa adduct with α,α,α-trifluoroacetophenone (1:1) in C_6D_6 / THF after 18 h at room temperature.

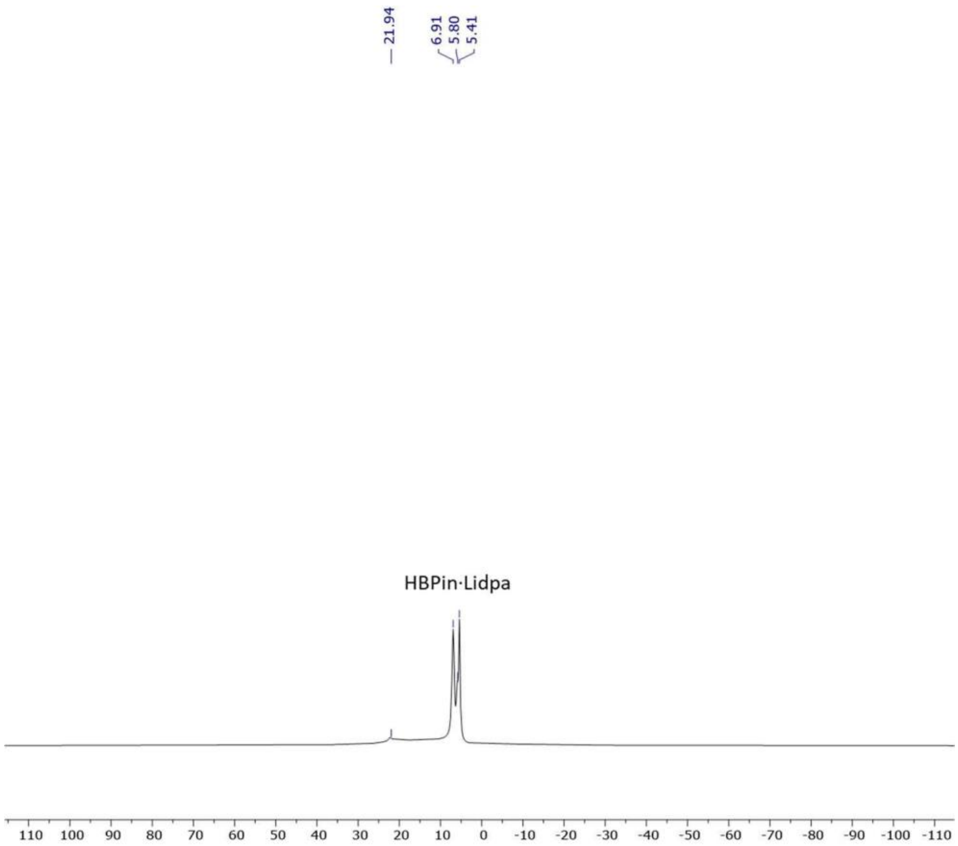

Figure S130. ^{11}B NMR spectrum of reaction of HBPin·Lidpa adduct with α,α,α-trifluoroacetophenone (1:1) in C_6D_6 / THF after 18 h at room temperature.

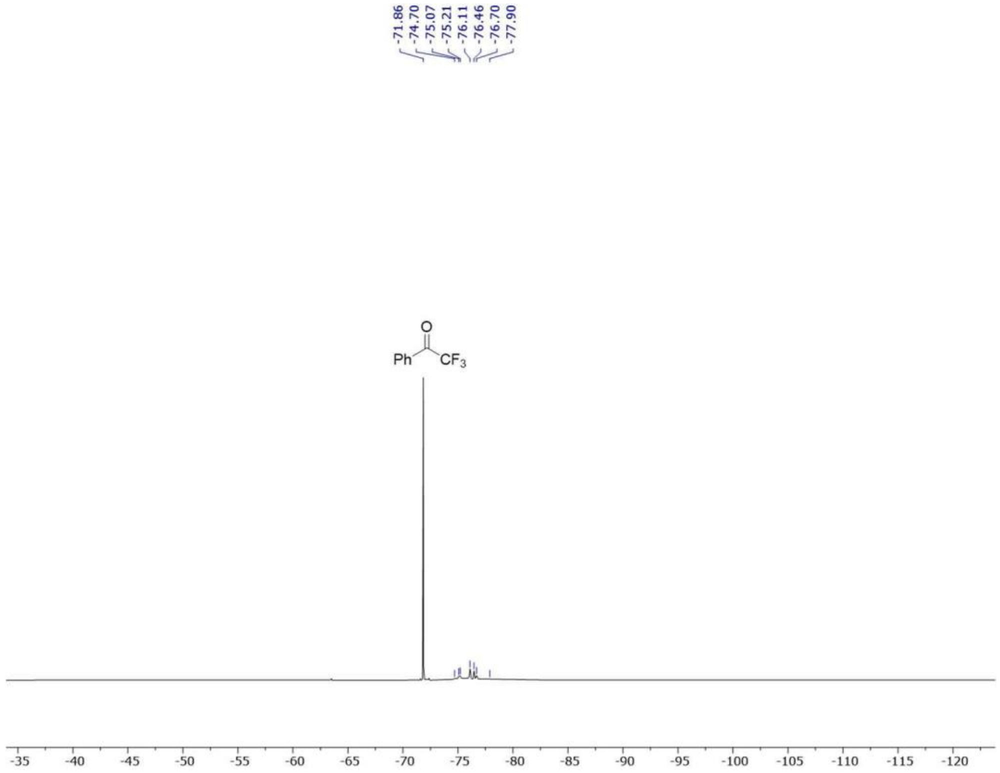

Figure S131. ^{19}F NMR spectrum of reaction of HBPin·Lidpa adduct with α,α,α-trifluoroacetophenone (1:1) in C_6D_6 / THF after 18 h at room temperature.

6. X-Ray Crystallography

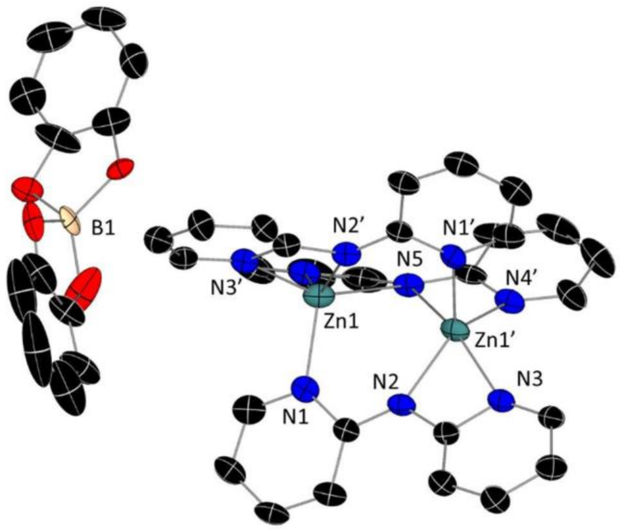

Figure S132. ORTEP representation of $[Zn_2dpa_3][BCat_2]$ (**9**) with ellipsoids at 50% probability level (symmetry operator: 1-x; 1-y; z). Hydrogen atoms, disorder components and free catechol omitted for clarity.

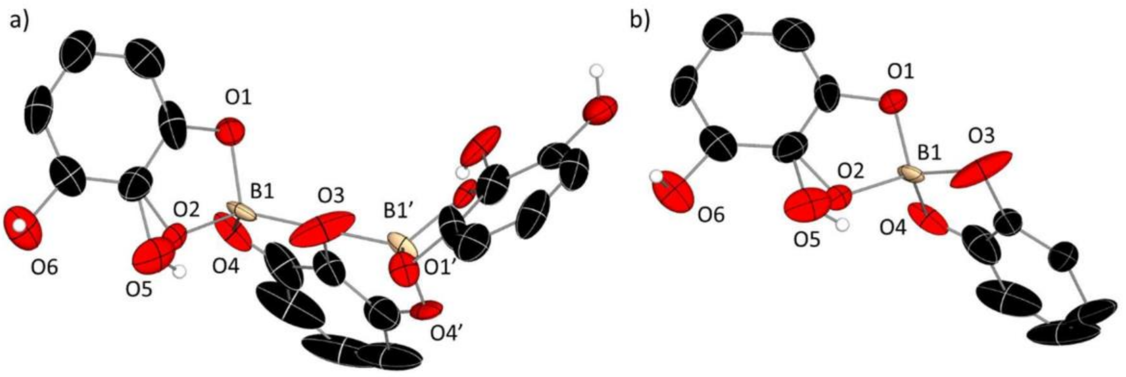

Figure S133. ORTEP representation of anionic part of complex **9** with thermal ellipsoids at 50% probability level: a) Full anion with disorder component and molecule of catechol overlapping (symmetry operator: 2-x; 1-y; z); b) Anion with disorder component omitted, but with shown molecule of catechol overlapping.

Crystallographic data for compounds **1**, **2**, **4** and **9** were recorded on a Rigaku Oxford Diffraction Excalibur diffractometer, at 120 K with Mo Kα radiation (λ = 0.71073 Å) equipped with Eos CCD detector. Crystallographic data for compound **5** were recorded on a Rigaku Oxford diffraction SuperNova diffractometer, at 120 K with Cu Kα radiation (λ = 1.54178 Å). Crystallographic data for compound **3** were recorded on a Bruker D8 VENTURE diffractometer, at 100 K with Mo Kα radiation (λ = 0.71073 Å). Crystallographic data for compound **8** were recorded at Diamond Light Source beam line I-19 EH1,[8] at 100 K with synchrotron radiation (λ = 0.68890 Å).

For data sets **1**, **2**, **4**, **5** and **9** the CrysAlisPro[9] software package was used for data collection, cell refinement and data reduction. The CrysAlisPro software package was used for empirical absorption corrections, which were applied using spherical harmonics, implemented in SCALE3 ABSPACK scaling algorithm. For data set **3** Bruker APEX3 software package was used for data collection, the applications SAINT[10] and SADABS[11] were used for the data reduction and absorption corrections of the data, respectively. For data set **8** Xia3/DIALS software package was used for data collection, the applications SAINT[10] and SADABS[11] were used for the data reduction and absorption corrections of the data, respectively. All further data processing was undertaken within the Olex2 software package.[12] The molecular structures of all compounds were solved with the ShelXT[13] structure solution program using Intrinsic Phasing and refined with the ShelXL[14-16] refinement package using Least Squares minimisation. Non-hydrogen atoms were refined anisotropically. Hydrogen atoms were all located in a difference map and repositioned geometrically.

Complex **2** was positionally/rotationally disordered over two positions, so was split into two parts and refined with an occupancy ratio of 0.95:0.05. Split same function in Olex2 was used to rotate the whole molecule fitting the Zn centres on the residual Q peaks. The anisotropic refinement of minor component resulted in an unstable refinement and was instead refined isotropically, with fixed U(iso) values for C and N-atoms, while Zn was freely refined.

The $\{BCat_2\}^-$ anion of complex **9** was modelled as disordered over a symmetry element, exchanging places with a neutral catechol molecule to retain charge balance. Restraints on ellipsoids and geometry were used where appropriate and are given in the embedded res file. Other ellipsoids were not split, despite elongation, as this brought no improvement in refinement and indeed the ellipsoids take care of disorder across the symmetry element.

Selected crystallographic data are presented in Table S2 and S3 and full details in cif format can be obtained free of charge from the Cambridge Crystallographic Data Centre via www.ccdc.cam.uk/data_request/cif.

Table S2. Selected crystallographic data for compounds **1** - **3**.

	**1**	**2**	**3**
CCDC No	2101029	2101030	2101033
Empirical formula	$C_{34}H_{42}LiN_3O_3Zn$	$C_{57}H_{39}D_9N_9Zn_3$	$C_{31}H_{41}LiN_6Zn$
Formula Weight	613.01	1064.21	570.01
Temperature (K)	120.00(11)	120.00(13)	100.0
Radiation	0.71073	0.71073	0.71073
Crystal system	Monoclinic	Monoclinic	Monoclinic
Space group	$P\ 2_1/c$	P 2/c	C 2/c
a (Å)	11.3465(5)	20.7441(4)	29.6035(15)
b (Å)	15.5769(6)	14.2785(2)	10.2616(5)
c (Å)	18.7091(9)	17.5264(3)	20.0024(10)
α(°)	90	90	90
β (°)	106.863(5)	106.317(2)	91.660(2)
γ (°)	90	90	90
Cell volume (Å^3)	3164.5(3)	4982.13(15)	6073.8(5)
Z	4	4	8
□calc (gcm^{-3})	1.287	1.419	1.247
μ (mm^{-1})	0.813	1.481	0.838
F (000)	1296.0	2172.0	2416.0
Crystal size/ mm^3	0.323 x 0.237 x 0.065	0.324 x 0.201 x 0.162	0.291 x 0.107 x 0.054
2θ range for data collection/°	6.686 to 61.448	6.668 to 58.858	4.652 to 66.392
Index ranges	$-15 \le h \le 16$; $-21 \le k \le 21$; $-26 \le l \le 26$	$-26 \le h \le 27$; $-19 \le k \le 19$; $-21 \le l \le 24$	$-45 \le h \le 45$; $-15 \le k \le 15$; $-30 \le l \le 30$
Reflections collected	78720	107408	266524
Independent reflections	9075 [R_{int} = 0.0605; R_{sigma} = 0.0433]	12534 [R_{int} = 0.0515; R_{sigma} = 0.0385]	11613 [R_{int} = 0.0386; R_{sigma} = 0.0150]
Data/restraints/parameters	9075/15/379	12534/183/699	11613/0/357
Goodnes-of-fit-on F^2 (*GOF*)	1.015	1.045	1.023
Final *R* indices [I>2σ(I)]	R_1 = 0.0440; wR_2 = 0.0961	R_1 = 0.0395; wR_2 = 0.0814	R_1 = 0.0307; wR_2 = 0.0808
R indices (all data)	R_1 = 0.0775; wR_2 = 0.1105	R_1 = 0.0586; wR_2 = 0.0879	R_1 = 0.0400; wR_2 = 0.0869
Largest diff. peak and hole (e Å^{-3})	0.54 / -0.40	0.61 / -0.41	0.43 / -0.58

Table S3. Selected crystallographic data for compounds **4**, **5**, **8** and **9**.

	**4**	**5**	**8**	**9**
CCDC No	2101031	2101027	2101028	2101032
Empirical formula	$C_{23}H_{21}N_3Zn$	$C_{40}H_{46}LiN_3O_3Zn$	$C_{26}H_{32}B_2Li_2N_6O_2$	$C_{48}H_{38}BN_9O_6Zn_2$
Formula Weight	404.08	689.11	496.07	978.42
Temperature (K)	120.01(13)	120.00(10)	100.0	120.01(13)
Radiation	0.71073	1.54178	0.68890	0.71073
Crystal system	Monoclinic	Monoclinic	Orthorhombic	Orthorhombic
Space group	$P\ 2_1/c$	$P\ 2_1$	$P\ 2_12_12_1$	$P\ 2_12_12$
a (Å)	9.8368(2)	8.88420(10)	10.756(3)	14.0270(8)

b (Å)	14.6402(2)	14.3691(2)	15.008(4)	17.4139(7)
c (Å)	14.2478(2)	14.33240(10)	17.229(5)	8.5193(3)
α(°)	90	90	90	90
β (°)	105.724(2)	99.2620(10)	90	90
γ (°)	90	90	90	90
Cell volume (Å^3)	1975.08(6)	1805.79(4)	2781.2(13)	2080.97(16)
Z	4	2	4	2
□calc (gcm^{-3})	1.361	1.267	1.185	1.561
μ (mm^{-1})	1.255	1.257	0.070	1.218
F (000)	840.0	728.0	1048.0	1004.0
Crystal size/ mm^3	0.352 x 0.258 x 0.099	0.485 x 0.098 x 0.076	0.045 x 0.008 x 0.008	0.305 x 0.169 x 0.124
2θ range for data collection/°	6.562 to 58.83	8.772 to 152.412	3.488 to 36.488	6.692 to 50.688
Index ranges	-13 ≤ h ≤ 13; -20 ≤ k ≤ 19; -18 ≤ l ≤ 19	-11 ≤ h ≤ 11; -16 ≤ k ≤ 18; -18 ≤ l ≤ 17	-9 ≤ h ≤ 9; -13 ≤ k ≤ 13; -15 ≤ l ≤ 15	-16 ≤ h ≤ 16; -20 ≤ k ≤ 19; -10 ≤ l ≤ 10
Reflections collected	43196	54236	18741	19725
Independent reflections	4951 [R_{int} = 0.0329; R_{sigma} = 0.0223]	7357 [R_{int} = 0.0697; R_{sigma} = 0.0382]	2181 [R_{int} = 0.0950; R_{sigma} = 0.0692]	3800 [R_{int} = 0.0629; R_{sigma} = 0.0572]
Data/restraints/parameters	4951/0/245	7357/1/454	2181/147/359	3800/161/329
Goodnes-of-fit-on F^2 (*GOF*)	1.083	1.114	1.086	1.090
Final *R* indices [I>2σ(I)]	R_1 = 0.0312; wR_2 = 0.0669	R_1 = 0.0494; wR_2 = 0.1347	R_1 = 0.0584; wR_2 = 0.1251	R_1 = 0.0537; wR_2 = 0.1225
R indices (all data)	R_1 = 0.0397; wR_2 = 0.0698	R_1 = 0.0505; wR_2 = 0.1359	R_1 = 0.0882; wR_2 = 0.1425	R_1 = 0.0698; wR_2 = 0.1306
Largest diff. peak and hole (e Å^{-3})	0.37 / -0.30	0.51 / -0.37	0.18/ -0.18	0.93 / -0.48
Flack parameter		-0.07(3)	-1.3(10)	0.000(9)

7. Computational details

All calculations were performed using the Gaussian09 series of programs.[17] Geometries were optimized with the DFT method using M06-2X functional[18] with a LANL2DZ basis set for Zn and 6-311G(d,p) basis set for the rest of the atoms. All geometry optimizations were full, with no restrictions. All stationary points were characterized as minima by vibrational analysis. Solvent effects of tetrahydrofuran were introduced using the self-consistent field approach, by means of the integral equation formalism polarizable continuum model (IEFPCM).[19] Full Cartesian coordinates for the optimised geometries are included in the .xyz file.

8. References

1. Concealed cyclotrimeric polymorph of lithium 2,2,6,6-tetramethylpiperidide unconcealed: X-ray crystallographic and NMR spectroscopic studies. E. Hevia, A. R. Kennedy, R. E. Mulvey, D. L. Ramsay, S. D. Robertson, Chem. Eur. J. 2013, 19, 14069 – 14075.
2. A structural and computational study of synthetically important alkali-metal/tetramethylpiperidide (TMP) amine solvates. D. R. Armstrong, D. V. Graham, A. R. Kennedy, R. E. Mulvey, C. T. O'Hara. *Chem. Eur. J.* **2008**, *14*, 8025 – 8034.

3. The synthesis and application of novel Ni(II) N-alkyldipyridylaldiminato complexes as selective ethylene oligomerisation catalysts. A. J. Swarts, S. F. Mapolie, *Dalton Trans.* **2014**, *43*, 9892 – 9900.
4. Accurate molecular weight determination of small molecules via DOSY-NMR by using external calibration curves with normalized diffusion coefficients. R. Neufeld, D. Stalke, *Chem. Sci.* **2015**, *6*, 3354 – 3364.
5. Alkoxide- and Hydroxide-Induced Nucleophilic Trifluoromethylation Using Tifluoromethyl Sulfone or Sulfoxide, G. K. S. Prakash, J. Hu, G. A. Olah, *Org. Lett.* **2003**, *5*, 3253 – 3256.
6. An Unsymmetric Imino-phosphanamidinate Ligand and its Y(III) Complex: Synthesis, Characterization, and Catalytic Hydroboration of Carbonyl Compounds, S. Anga, J. Acharya, V. Chandrasekhar, *J. Org. Chem.* **2021**, *86*, 2224 – 2234.
7. Trifluoromethylation of Ketones and Aldehydes with Bu_3SnCF_3, I. A. Sanhueza, K. J. Bonney, M. C. Nielsen, F. Schoenebeck, *J. Org. Chem.* **2013**, *78*, 7749 – 7753.
8. Remote access revolution: chemical crystallographers enter a new era at Diamond Light Source beamline I19. N. T. Johnson, P. G. Waddell, W. Clegg, M. R. Probert, *Crystals* **2017**, *7*, 360 – 368.
9. CrysAlisPro, Agil. Technol. Version 1.1 71.35.19 (release 27-10-2011 CrysAlis171.NET) (compiled Oct 27 2011,150211).
10. Bruker, *Bruker AXS*, Inc. SAINT, Version 7.68A; Bruker AXS: Madison WI 2009
11. G. M. Sheldrick, SADABS, Version 2008/2; University of Göttingen: Germany, 2003.
12. OLEX2: a complete structure solution, refinement and analysis program. O. V. Dolomanov, L. J. Bourhis, R. J. Gildea, J. A. K. Howard, H. Puschmann, *J. Appl. Cryst.* **2009**, *42*, 339 – 341.
13. *SUPERFLIP*- a computer program for the solution of crystal structures by charge flipping in arbitrary dimensions. L. Palatinus, G. Chapuis, *J. Appl. Cryst.* **2007**, *40*, 786 – 790.
14. Symmetry determination following structure solution in P1. L. Palatinus, A. Van der Lee, *J. Appl. Cryst.* **2008**, *41*, 975 – 984.
15. EDMA: a computer program for topological analysis of discrete electron densities. L. Palatinus, S. J. Prathapa, S. Van Smaalen, *J. Appl. Cryst.* **2012**, *45*, 575 – 580.
16. A short history of SHELX. G. M. Sheldrick, *Acta Cryst.* **2008**, *A64*, 112 – 122.
17. Gaussian 09, Revision C1, Frisch, M. J.; Trucks, G. W.; Schlegel, H. B.; Scuseria, G. E.; Robb, M. A.; Cheeseman, J. R.; Scalmani, G.; Barone, V.; Mennucci, B.; Petersson, G. A.; Nakatsuji, H.; Caricato, M.; Li, X.; Hratchian, H. P.; Izmaylov, A. F.; Bloino, J.; Zheng, G.; Sonnenberg, J. L.; Hada, M.; Ehara, M.; Toyota, K.; Fukuda, R.; Hasegawa, J.; Ishida, M.; Nakajima, T.; Honda, Y.; Kitao, O.; Nakai, H.; Vreven, T.; Montgomery, Jr., J. A.; Peralta, J. E.; Ogliaro, F.; Bearpark, M.; Heyd, J. J.; Brothers, E.; Kudin, K. N.; Staroverov, V. N.; Kobayashi, R.; Normand, J.; Raghavachari, K.; Rendell, A.; Burant, J. C.; Iyengar, S. S.; Tomasi, J.; Cossi, M.; Rega, N.; Millam, J. M.; Klene, M.; Knox, J. E.; Cross, J. B.; Bakken, V.; Adamo, C.; Jaramillo, J.; Gomperts, R.; Stratmann, R. E.; Yazyev, O.; Austin, A. J.; Cammi, R.; Pomelli, C.; Ochterski, J. W.; Martin, R. L.; Morokuma, K.; Zakrzewski, V. G.; Voth, G. A.; Salvador, P.; Dannenberg, J. J.; Dapprich, S.; Daniels, A. D.; Farkas, Ö.; Foresman, J. B.; Ortiz, J. V.; Cioslowski, J.; Fox, D. J. Gaussian, Inc., Wallingford CT, **2009**.
18. The Mo6 suite of density functionals for main group thermochemistry, thermochemical kinetics, noncovalent interactions, excited states, and transition elements: two new functionals and systematic testing of four Mo6-class functionals and 12 other functionals. Zhao Y.; Truhlar D. G., *Theor. Chem. Acc.* **2008**, *120*, 215-241.

19. Evaluation of Solvent Effects in Isotropic and Anisotropic Dielectrics and in Ionic Solutions with a Unified Integral Equation Method: Theoretical Bases, Computational Implementation, and Numerical Applications. Mennucci B.; Cancès E.; Tomasi J., *J. Phys. Chem. B* **1997**, *101*, 10506-10517.
